# Donor Strengths Determination of Pnictogen and Chalcogen Ligands by the Huynh Electronic Parameter and Its Correlation to Sigma Hammett Constants

**DOI:** 10.1002/chem.201902795

**Published:** 2019-09-30

**Authors:** Qiaoqiao Teng, Ping Siang Ng, Jia Nuo Leung, Han Vinh Huynh

**Affiliations:** ^1^ Department of Chemistry National University of Singapore 3 Science Drive 3 Singapore 117543 Singapore; ^2^ Current address: School of Petrochemical Engineering Changzhou University Changzhou 213164 P. R. China

**Keywords:** donor strength, electronic parameters, palladium, pnictogens, substituent effects

## Abstract

The suitability and accuracy of the Huynh electronic parameter (HEP) was further tested to reveal remote substituent effects in pyridines, which are located five or six bonds away from the reporter probe. These values show an excellent correlation to Hammett *σ*‐constants of the respective substituents with coefficients of *R* ^2^=0.9856 (*σ*_m_) and *R* ^2^=0.9857 (*σ*_p_). Based on this observation, a methodology for the re‐evaluation of certain Hammett constants with larger uncertainties has been proposed and demonstrated. Moreover, the scope of HEP was extended to various neutral pnictogen and chalcogen donors during which “*transphobia* effects” were revealed for mixed NHC complexes containing phosphites, arsine and stibine for the first time.

## Graphical abstract

**Remote substituent effects** can be revealed by Huynh electronic parameters and an excellent correlation to Hammett constants was observed. In addition, the electronic properties of selected pnictogen and chalcogen ligands were determined on a unified scale.

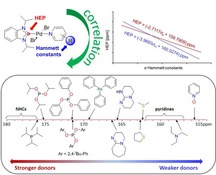

## Introduction

**Figure 1:**
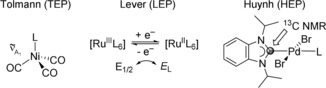
Selected experimental electronic parameters.

The properties of metal complexes are determined by the nature of the metal center and the stereoelectronic signatures imposed by the ligands. Often the steric bulk of a particular ligand can be easily estimated by its Lewis structure drawing, while its electronic properties are more difficult to judge. This is especially so, when the ligand has multiple substituents of different inductive and mesomeric effects, which could enhance or oppose each other. In order to compare the donating abilities of ligands experimentally, a few electronic parameters have been developed (Figure 1).

The ligand electrochemical parameter *E*_L_ (LEP) has been introduced by Lever based on redox potentials of for example, Ru^II/III^ metal complexes.^1^ The *E*_L_ values of a large number of Werner‐type ligands have been tabulated, which reflect their relative capacity to stabilize a metal in a certain oxidation state. Ligands with smaller *E*_L_ values can therefore stabilize the Ru^III^ state in the Ru^II/III^ couple better than those with larger *E*_L_ values. Although *E*_L_ values are not a direct measure for the donating ability of a ligand, they are nevertheless related to its donating power.^2^ The drawback is that only complexes with reversible redox chemistry can be considered for the determination of *E*_L_ values. The requirement for less common electrochemical setups and the exclusion of non‐innocent ligands is a further limitation.

In organometallic chemistry, the most commonly used parameter is the so‐called Tolman electronic parameter (TEP) developed in 1970, which compares the A_1_ carbonyl IR stretching frequencies of [Ni(CO)_3_L] complexes, in which L denotes the ligand of interest.^3^ This methodology evaluates the amount of π‐backdonation from the nickel(0) center to the carbonyl ligands, which is in turn influenced by the donor/acceptor power of the ligand L. For phosphines, for which σ‐donor and π‐acceptor strengths are approximately inversely proportional, TEP has become a valuable tool of assessment. For ligands that do not show such behavior, interpretation of TEPs is less straightforward, since deconvolution into donor and acceptor contributions is not possible. Another disadvantage of TEP and its Rh/Ir variants^4^ is the requirement for highly toxic materials, that is, [Ni(CO)_4_] or carbon monoxide. The largest drawback, however, is that the majority of Werner‐type ligands cannot be systematically probed using carbonyl‐based methods. For example, amines and pyridines do not react with [Ni(CO)_4_].

We have introduced a new electronic parameter, which addresses these shortcomings and allows direct comparison of Werner‐type and organometallic ligands on a unified scale without the use of highly toxic materials.^5^ Our parameter, that is, Huynh electronic parameter (HEP), evaluates the influence of a particular *trans*‐ligand L on the ^13^C_carbene_ NMR shift of the ^*i*^Pr_2_‐bimy reporter ligand (i.e. HEP signal) in square‐planar complexes of the type *trans*‐[PdBr_2_(^*i*^Pr_2_‐bimy)L]. It was found that a weaker donor would lead to a relative upfield shift, whereas a stronger donor would lead to a downfield shift of the HEP signal. Different types of NHCs,^6^ phosphines, isocyanides, and N‐donors have been evaluated.^7^, ^8^, ^9^, ^10^ Furthermore, much smaller electronic differences among similar ligands can be resolved, providing useful information for the fine‐tuning of complexes. To test its suitability further, we herein report a systematic evaluation of remote substituent effects by using *meta*‐ and *para*‐substituted pyridines and correlated the HEP results to the respective *σ*_p_ and *σ*_m_ Hammett constants.^11^ Subsequently, the HEP scale was extended to various neutral pnictogen and chalcogen ligands such as phosphites, arsines, stilbenes, etc. to provide insights into their electronic differences.

## Results and Discussion

### HEPs of substituted pyridines and correlation to *σ‐*Hammett constants

One advantage of the HEP is the ease of complex probe preparation. Thus, simple mixing of the dimeric complex [PdBr_2_(^*i*^Pr_2_‐bimy)]_2_ (**I**)^12^ with two equivalents of the substituted pyridines in dichloromethane at ambient temperature already results in rapid reactions. In all cases, the initial orange suspensions clear into bright‐yellow solutions (Table 1). To ensure completion, the mixtures were allowed to stir for 2 h. Overall, 26 new *trans*‐[PdBr_2_(^*i*^Pr_2_‐bimy)(Py‐R)] complexes were prepared with 13 *meta*‐ (**2**–**14**) and 13 *para*‐substituted pyridines (**15**–**27**) and compared to the previously reported parent complex *trans*‐[PdBr_2_(^*i*^Pr_2_‐bimy)(Py)] (**1**).^13^, ^14^

**Table 1:** Synthesis of *trans*‐[PdBr_2_(^*i*^Pr_2_‐bimy)(Py‐R)] complexes **2**–**27**, in which Py‐R=*meta* or *para*‐substituted pyridines, and their characteristic ^1^H NMR signals [ppm], HEP values [ppm],^[a]^ and Hammett *σ‐*constants in CDCl_3_.

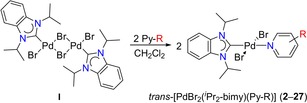				
R‐group	δNCH	δPyH_2,6_	HEP	*σ*_m/p_
3‐CN (**2**)	6.25	9.53, 9.43	157.3_3_	0.56
3‐NO_2_ (**3**)	6.28	10.10, 9.55	157.3_8_	0.71
3‐CHO (**4**)	6.31	9.67, 9.43	158.5_4_	0.35
3‐Br (**5**)	6.29	9.26, 9.11	158.6_3_	0.39
3‐I (**6**)	6.29	9.36, 9.12	158.6_8_	0.35
3‐Cl (**7**)	6.30	9.18, 9.07	158.7_2_	0.37
3‐F (**8**)	6.30	9.12, 9.02	158.7_3_	0.34
3‐CO_2_H (**9**)	6.33	9.81, 9.37	159.0_4_	0.37
3‐OH (**10**)	6.32	8.63, 8.54	159.6_0_	0.12
3‐Ph (**11**)	6.37	9.39, 9.12	159.8_0_	0.06
3‐Et (**12**)	6.35	8.94, 8.92	160.3_3_	−0.07
3‐Me (**13**)	6.34	8.91, 8.89	160.4_1_	−0.07
3‐NH_2_ (**14**)	6.33	8.51, 8.44	160.4_7_	−0.16
4‐CN (**15**)	6.25	9.42	157.7_9_	0.66
4‐CF_3_ (**16**)	6.29	9.40	158.3_8_	0.54
4‐CHO (**17**)	6.30	9.43	158.7_6_	0.42
4‐Cl (**18**)	6.29	9.08	158.9_8_	0.23
4‐CO_2_H (**19**)	6.31	9.36	159.0_0_	0.45
4‐Br (**20**)	6.29	8.94	159.0_3_	0.23
4‐I (**21**)	6.28	8.79	159.2_1_	0.18
4‐Ph (**22**)	6.38	9.16	160.1_6_	−0.01
4‐OMe (**23**)	6.33	8.92	160.3_9_	−0.27
4‐Me (**24**)	6.33	8.93	160.4_3_	−0.17
4‐Et (**25**)	6.34	8.96	160.5_8_	−0.15
4‐NH_2_ (**26**)	6.34	8.61	161.3_5_	−0.66
4‐NMe_2_ (**27**)	6.36	8.61	161.9_7_	−0.83

[a] HEP values are given with the second decimal in subscript for comparison. Detailed discussion on standard deviations of HEP can be found in References [5], [7] and [8].

All complexes are air‐ and moisture‐stable and were characterized by ^1^H and ^13^C NMR spectroscopies, ESI mass spectrometry and elemental analysis. Single crystals for six complexes were also obtained as representatives by slow evaporation of their solutions, and X‐ray diffraction analyses confirm their desired compositions (vide infra). The ^1^H NMR spectra of all compounds show the presence of the ^*i*^Pr_2_‐bimy and the pyridine derivative in an expected 1:1 ratio. Upon coordination, the *ortho*‐proton signals of the pyridine derivatives shift downfield by 0.4–0.6 ppm (i.e. δPyH_2,6_ in Table 1). Another striking feature is the downfield shift of 1.04–1.17 ppm observed for the isopropyl C−H protons (i.e. δNCH in Table 1) of the complexes in comparison to those in the 1,3‐diisopropyl‐benzimidazolium bromide salt at 5.21 ppm. These suggest the presence of anagostic C−H⋅⋅⋅Pd interactions, which are quite common for d^8^ metal complexes with ^*i*^Pr_2_‐bimy ligands.^15^ Similar spectral observations were also made for the related compounds with the parent pyridine and bridging bipyridine ligands.^13^

More important for our purpose are the ^13^C NMR chemical shifts of the ^*i*^Pr_2_‐bimy reporter ligand, that is, HEP values. The good solubility of the complexes allows an easy detection of these signals, and they are summarized in Table 1. In general, electron releasing substituents lead to small downfield shifts, while electron‐withdrawing ones induced an upfield trend with respect to the parent unsubstituted pyridine complex (R=H), which has an HEP value of 160.0_1_ ppm.^8^ Notably, remote changes five (*meta*) or six (*para*) bonds away can be differentiated by the ^*i*^Pr_2_‐bimy reporter signal in line with chemical intuition. In this respect, it is worth mentioning that a standard deviation of SD=0.01 ppm can be estimated by the full‐width‐at‐half‐maximum (fwhm≈0.02 ppm) for typical ^13^C NMR signals.^5^, ^7^, ^8^

In organic chemistry, electronic substituent effects have been quantified by the Hammett *σ*‐constants, which have been defined using *meta* and *para* substituted benzoic acids, while no constants were considered for *ortho* derivatives due to steric interferences.^16^ They are typically based on conductance measurements of ionization constants of the appropriately substituted benzoic acids [Eq. (1)], in which *K*_H_ is the ionization constant for benzoic acid in water at 25 °C, and *K*_X_ is the ionization constant for a *meta‐* or *para‐*substituted benzoic acid.^11^, ^16^

(1) $$\sigma \;\left(X\right)=\log K{}_{X}-\log K{}_{H}$$

Electron‐withdrawing substituents would increase the acidity giving rise to positive values of Hammett *σ‐*constants and vice versa. In addition, these constants have also been shown to reflect the electron densities of substituted pyridines.^17^ Hence, the HEP values obtained for complexes **2**–**27** were correlated to the well‐accepted and widely used Hammett constants in order to further evaluate our methodology in terms of accuracy (Table 2).

**Figure 2:**
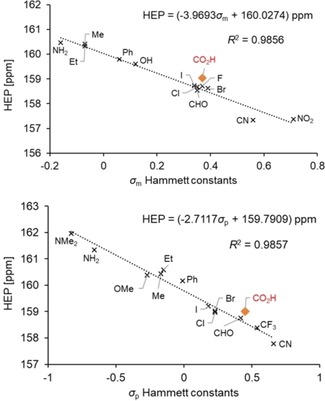
Graphs and equations for the regressions of (i) HEP vs. *σ*_m_ Hammett constants (upper) and (ii) HEP vs. *σ*_p_ Hammett constants (lower).

Notably, an excellent correlation was already observed using all data with linear regression coefficients of *R* ^2^=0.9773 and *R* ^2^=0.9816 for the *meta* and *para* series, respectively. In an early paper, McDaniel and Brown highlighted that there were considerable variations in the *σ*‐values for the 3‐ and 4‐CO_2_H substituents (0.37 and 0.45) with large estimated limits of uncertainty of around 0.1.^18^ Based on this statement, the correlation study was repeated with the exclusion of the carboxylic acid group, which led to further improvements of the coefficients to *R* ^2^=0.9856 (*meta*) and *R* ^2^=0.9857 (*para*). The linear regression graphs and equations are depicted in Figure 2.

The resulting equations allow interconversion of HEP into Hammett *σ* constants or vice versa [e.g., Eqs. (2), (3)]. For example, using the reported HEP value for 3‐ethynylpyridine (3‐Py‐C_2_H) of 159.2 ppm^8^ and Equation 2, we can predict a *σ*_m_ value of 0.20 for the ethynyl substituent, which is very close to the reported value of 0.21.^11^

(2) $$\sigma {}_{m}=\left(\mathrm{HEP}-160.0433\right)/-4.1235$$

(3) $$\sigma {}_{p}=\left(\mathrm{HEP}-159.7855\right)/-2.7171$$

**Figure 3:**
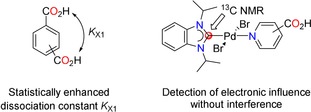
Determination of the Hammett constant and HEP for the carboxylic acid substituent.

Since Hammett *σ*‐values are determined from acidity constants of benzoic acids, it is actually understandable why the uncertainties for the carboxylic acid substituent are rather large. The increased acidities of isophthalic (p*K*_a1_=3.70) and terephthalic acid (p*K*_a1_=3.54) compared to that of the parent benzoic acid (p*K*_a_=4.19) cannot be solely attributed to the electron‐withdrawing nature of the CO_2_H group. Due to the presence of two identical acid functions in these molecules, the proton dissociation is statistically enhanced. Thus, the electronic influence of the CO_2_H group is likely to be overestimated resulting in larger *σ*‐values. On the other hand, the HEP values of the pyridine carboxylic acid complexes **9** and **19** reflect the intrinsic electronic property of the molecules without such interferences (Figure 3). Application of Equations 2 and 3 with these HEP values gives lower calculated constants of *σ*_m_*=0.25 and *σ*_p_*=0.29.

**Scheme 1:**
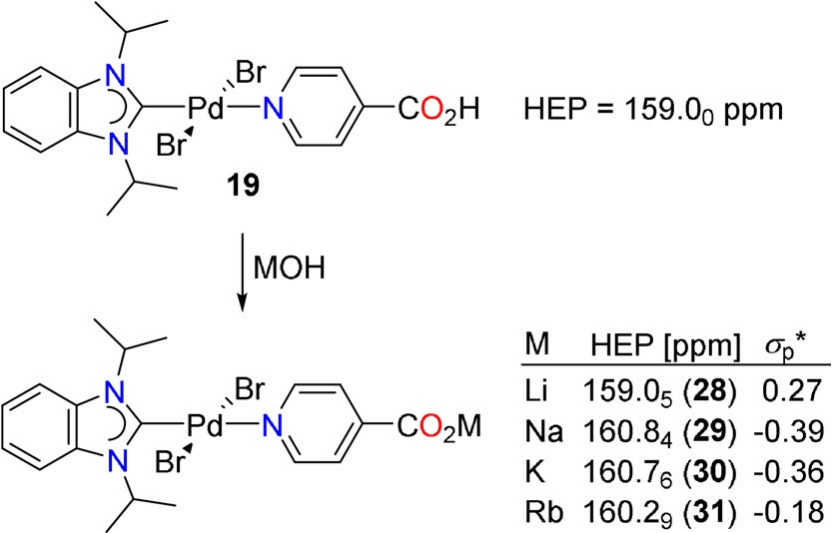
Synthesis of HEP probes with different alkali pyridinecarboxylate ligands.

McDaniel and Brown also noted that ionic substituents have Hammett constants with larger uncertainties.^18^ An example is the carboxylate group (CO_2_^−^)_,_ which has been assigned a zero value for *σ*_p_ without any consideration of cation effects.^18^ However, we suppose that the electronic influence of this group cannot be cation‐independent. To test this notion, we subjected the complex *trans*‐[PdBr_2_(^*i*^Pr_2_‐bimy)(Py‐4‐CO_2_H)] (**19**) to common alkali metal hydroxides to obtain HEP probes of pyridyl‐4‐carboxylates with different alkali metal ions (Scheme 1).

Upon deprotonation, an increased donating ability is expected for the resulting pyridinecarboxylate ligand, which is indeed confirmed by the larger HEP values. As anticipated, different cations induce a different response, and the downfield shifts are smallest for Li^+^ and largest for Na^+^ and K^+^. This observation implies that the negative charge is best stabilized by the lithium cation, which is in line with its largest charge/size ratio leading to the best hard/hard interactions with the carboxylate group. The HEP value peaks with sodium and gradually decrease with increasing size of the cation. The difference between potassium and rubidium is larger as the latter is known to form compounds with higher coordination numbers. Scheme 1 also depicts the calculated *σ*_p_* constants for the different alkali metal carboxylates using Equations 2 and 3.

**Figure 4:**
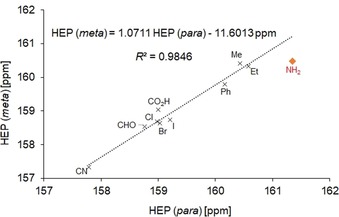
Regression graphs and equation of HEP (*meta*) vs. HEP (*para*).

With the availability of HEP data summarized in Table 1, it is lastly also of interest to study if the HEP value of a *meta*‐substituted pyridine could be predicted by that of its *para*‐analogue and vice versa. In order to test such a possibility, HEPs of *meta*‐ and *para*‐substituted pyridines bearing identical substituents were plotted against each other where available. With inclusion of all ten substituents, a very good linear regression coefficient of *R* ^2^=0.9701 was obtained. Exclusion of the NH_2_ group as an outlier led to a further improvement to *R* ^2^=0.9846 (Figure 4).

Based on this encouraging result, it appears that substituents in *meta*‐ and *para*‐position exhibit a similar (almost linear) effect on the HEP, potentially allowing for predictions from each other. This could be particularly powerful for groups with primarily inductive effects, while those with dominant mesomeric contributions have to be treated with more caution. The latter is understandable as mesomeric effects have a drastically different impact in *meta*‐ compared to *para*‐position.

### Determining the HEPs of selected pnictogen donors

Having established an excellent correlation of HEP with Hammett constants, we focused on the extension of our method to previously unaccounted ligands. In particular, triethylamine (NEt_3_), 1,5,7‐triazabicyclo[4.4.0]dec‐5‐ene (TBD) and 1,8‐diazabicyclo[5.4.0]undec‐7‐ene (DBU) are widely used organic bases, but they can also stabilize active metal species by coordination. It is therefore surprising that their donating abilities have not been explicitly compared yet. Hence, the respective HEP probes were routinely prepared by exposure of [PdBr_2_(^*i*^Pr_2_‐bimy)]_2_ (**I**)^12^ to the free amine bases. The yellow products *trans*‐[PdBr_2_(^*i*^Pr_2_‐bimy)(TBD)] (**32**) and *trans*‐[PdBr_2_(^*i*^Pr_2_‐bimy)(DBU)] (**33**) formed rapidly, while prolonged stirring with an excess of triethylamine was required to fully convert complex **I** into *trans*‐[PdBr_2_(^*i*^Pr_2_‐bimy)(NEt_3_)] (**34**). TBD is a bicyclic guanidine containing an imine and a secondary amine function that are available for coordination. However, the ligand shows selective coordination of the imine nitrogen to metal centers such as Pd, Cu, Ag, Fe and Li,^19^, ^20^ which is also confirmed by single crystal X‐ray diffraction of TBD complex **32** (vide infra). Coordination via the imine function is also expected for the DBU ligand. The three base adducts **32**–**34** have good solubilities in most organic solvents, and no *cis*‐isomers were detected. In the ^13^C NMR spectra, the three carbene carbon atoms resonate in the order of **34** (NEt_3_, 157.9_6_ ppm)<**32** (TBD, 165.7_4_ ppm)<**33** (DBU, 166.3_2_ ppm, Table 2).^21^ This trend indicates that the aliphatic amine NEt_3_ is a significantly weaker donor compared to the unsaturated imines, which is in agreement with a greater availability of the lone pair of *sp* ^2^‐hybridized imine donors. The weaker donating effect of NEt_3_ comparable to the weakest pyridines (Table 1) also explains its sluggish reaction with dimer **I**. In addition, DBU is a stronger ligand than TBD, which is reasonable by considering the additional −*I* effect from the third nitrogen atom in the TBD ligand.

**Table 2:** Selected NMR spectroscopic data of complexes **32**–**34** and *trans*‐**36**–**39** measured in CDCl_3_.

Complex	Ligand	^1^H_Me_ [ppm]	HEP [ppm]	^2^ *J*(C,P) [Hz]
**32**	TBD	1.74	165.7_4_	–
**33**	DBU	1.77	166.3_2_	–
**34**	NEt_3_	1.74	157.9_6_	–
*trans*‐**36**	P(O^*i*^Pr)_3_	1.77	175.2_5_	287.1
*trans*‐**37**	P(OPh)_3_	1.51	171.6_5_	289.8
*trans*‐**38**	P(O‐2,4‐^*t*^Bu‐Ph)_3_	1.59	171.4_8_	289.6
*trans*‐**39**	AsPh_3_	1.79	169.1_7_	–

In addition to nitrogen donors, HEP has also been used to evaluate different types of phosphines.^8^, ^22^, ^23^ It is thus of interest to extend the scope to other pnictogen donors such as phosphites, arsines and stibines. This study was initiated with P(OMe)_3_, P(O^*i*^Pr)_3_, P(OPh)_3_ and P(O‐2,4‐^*t*^Bu‐Ph)_3_, which are diverse in terms of stereoelectronic properties. Instant color changes from orange to pale yellow were observed for most reaction mixtures indicative of a generally strong donating ability of these ligands and the fast formation of mixed NHC/phosphite complexes, which are surprisingly rare.^24^, ^25^ Similar to their phosphine counterparts, electronically driven *trans–cis* isomerizations were observed. The thermodynamically preferred *cis*‐arrangement in these complexes is due to the so‐called “*transphobia* effect” of phosphorus donors.^12^, ^26^, ^27^ This term was introduced to describe the general difficulty of placing a phosphorus *trans* to a carbon donor.^28^ The isomerization is fastest for the P(OMe)_3_ and P(O^*i*^Pr)_3_ complexes containing the smallest phosphites. Hence, only the *cis*‐[PdBr_2_(^*i*^Pr_2_‐bimy){P(OMe)_3_}] (*cis*‐**35**) and *cis*‐[PdBr_2_(^*i*^Pr_2_‐bimy){P(O^*i*^Pr)_3_}] (*cis*‐**36**) complexes were isolated in the standard procedures. The process is slower for P(OPh)_3_ complex, and signals for both *trans*/*cis*‐[PdBr_2_(^*i*^Pr_2_‐bimy){P(OPh)_3_}] (*trans*‐/*cis*‐**37**) complexes were captured in the NMR spectra. The complex *trans*‐[PdBr_2_(^*i*^Pr_2_‐bimy){P(O‐2,4‐^*t*^Bu‐Ph)_3_}] (*trans*‐**38**) resists isomerization due to the enhanced steric bulk, and a HEP value of 171.4_8_ ppm was detected for the P(O‐2,4‐^*t*^Bu‐Ph)_3_ ligand without problems (Table 2).

Since *trans*‐configured complex probes are required for the HEP method, direct NMR scale reactions were carried out to capture the signals of the initially formed *trans*‐isomers for the less bulky phosphites before complete isomerization. Upon mixing the reactants in NMR tubes, the samples were immediately measured after addition of CDCl_3_. Indeed, only one set of signals due to the *trans*‐[PdBr_2_(^*i*^Pr_2_‐bimy){P(O^*i*^Pr)_3_}] (*trans*‐**36**) or *trans*‐[PdBr_2_(^*i*^Pr_2_‐bimy){P(OPh)_3_}] (*trans*‐**37**) complexes was observed in the respective ^1^H NMR spectra. ^31^P and ^13^C NMR data were measured immediately thereafter. This approach was successful for complex *trans*‐**37**, and a HEP of 171.6_5_ ppm was obtained for the P(OPh)_3_ ligand. However, signals due to both *trans*‐ and *cis*‐isomers were detected in the ^13^C NMR spectrum of **36**. To resolve the carbene signal of *trans*‐**36** in a shorter time, the NMR reaction was repeated with ^13^C2‐labeled complex **I**,^5^ which allowed detection of the HEP signal for P(O^*i*^Pr)_3_ at 175.2_5_ ppm with a single scan. Unfortunately, the signals of *trans*‐**35** complex could not be captured even within such a short time due to its very rapid *trans–cis* isomerization. Overall, the donating ability of the phosphites decreases in the order P(O^*i*^Pr)_3_≫P(OPh)_3_>P(O‐2,4‐^*t*^Bu‐Ph)_3_ (Table 2). Generally, they are also weaker donors compared to phosphines with the same substituents. No HEP value was obtained for P(OMe)_3_ due to its strong *transphobia*. However, it is intuitive to place it in between P(O^*i*^Pr)_3_ and P(OPh)_3_, which is reflected in the trends of the available *cis* complexes (Table 3).

**Table 3:** Selected NMR spectroscopic data of complexes *cis*‐**35**–**37**, *cis*‐**39** and *cis*‐**40** measured in CDCl_3_.

Complex	Ligand	^1^H_Me_ [ppm]	HEP [ppm]	^2^ *J*(C,P) [Hz]
*cis*‐**35**	P(OMe)_3_	1.74, 1.66	170.7_9_	21.4
*cis*‐**36**	P(O^*i*^Pr)_3_	1.71, 1.69	171.9_8_	22.9
*cis*‐**37**	P(OPh)_3_	1.57, 1.14	169.2_7_	22.9
*cis*‐**39**	AsPh_3_	1.66, 0.89	169.3_4_	–
*cis*‐**40**	SbPh_3_	1.66, 1.04	167.4_6_	–

Similar *trans*–*cis* isomerizations were also observed for mixed NHC/AsPh_3_ and NHC/SbPh_3_ complexes. The process is particularly rapid for the antimony compound, and only *cis*‐[PdBr_2_(^*i*^Pr_2_‐bimy)(SbPh_3_)] (*cis*‐**40**) could be detected by all means. Complex *trans*‐[PdBr_2_(^*i*^Pr_2_‐bimy)(AsPh_3_)] (**39**), on the other hand, isomerizes slower and could be fully characterized by direct NMR reaction, giving a HEP value of 169.1_7_ ppm. This arsine is therefore a weaker donor compared to phosphines and phosphites. Comparison of the *cis*‐**39** and *cis*‐**40** could indicate that the stibine is a weaker donor than the arsine (Table 3), which is in agreement with TEP studies.^29^

After having obtained the carbene signals of the *trans*‐configured pnictogen complexes **36**–**39**, the isomerization processes were purposely monitored by ^1^H NMR spectroscopy in CDCl_3_. Differentiation of *trans*‐ versus *cis*‐configured products can be best achieved by analyzing the ^1^H NMR signals of methyl groups in the ^*i*^Pr_2_‐bimy ligand. For the symmetrical *trans*‐isomers, only one doublet is observed, while non‐equivalence in the *cis*‐isomers leads to two doublets of equal integration. In addition, the phosphite complexes can be distinguished by the ^31^P coupled carbene signals. The *trans*‐isomers exhibit ^2^ *J*(C,P) coupling constants of >280 Hz (Table 2), while <23 Hz are observed for *cis*‐isomers (Table 3).

The P(O^*i*^Pr)_3_ complex *trans*‐**36** fully converted to *cis*‐**36** within two hours. For the P(OPh)_3_ complex **37**, only 17 % was converted to the *cis*‐isomer after eight days. However, the ^1^H NMR spectrum of the product mixture obtained from the initial lab‐scale reaction (30 min stirring in CH_2_Cl_2_) shows a *trans*–*cis* conversion of 50 %. Besides better mixing, the different solvent would be the key factor in influencing the isomerization rate. The increased polarity of CH_2_Cl_2_ compared to CDCl_3_ should favor the formation of the *cis*‐isomer with a larger dipole moment. Hence, by repeating the reaction in the even more polar organic solvent MeOH, *cis*‐[PdBr_2_(^*i*^Pr_2_‐bimy){P(OPh)_3_}] (*cis*‐**37**) was exclusively obtained after 10 hours of stirring at ambient temperature. The P(O‐2,4‐^*t*^Bu‐Ph)_3_ complex *trans*‐**38** did not change at all after ten days. Heating the NMR sample in an oil bath (65 °C) for several days led to decomposition to palladium black, but no *cis*‐isomer was detected. The isomerization of the AsPh_3_ complex **39** seems to reach equilibrium after six days giving rise to a *trans*/*cis* mixture with a ratio of 1:3.5. Due to *transphobia*, only single crystals of the *cis*‐adducts could be obtained (vide infra).

### Determining the HEPs of selected neutral chalcogen donors

Neutral chalcogen donors form weaker bonds with metal centers compared to their pnictogen counterparts. Their weak donor capability makes them suitable candidates for the design of hemilabile hybrid ligands for catalysis. For example, complexes of thioether‐functionalized NHC ligands have shown promising catalytic activities and an interesting structural dynamics.^30^, ^31^, ^32^, ^33^, ^34^ However, less attention has been paid to the measurement of their electron donating abilities compared to those of the other ligands. Herein, the electron donating ability of two common thioethers, that is, dimethylsulfide (DMS) and tetrahydrothiophene (THT) are assessed. We were also interested in studying the coordination chemistry and electron donating properties of analogous neutral oxygen donors. Unfortunately, dialkyl ethers are even weaker donors and were not able to cleave the dimeric complex **I**. Pyridine *N*‐oxide (PNO) as a formally neutral oxygen donor was included instead. The increased dipole moment due to charge separation leads to a stronger nucleophilic property of the oxygen atom, which may allow the cleavage reaction to proceed. The two thioethers smoothly cleaved complex **I**, affording complexes *trans*‐[PdBr_2_(^*i*^Pr_2_‐bimy)(DMS)] (**41**) and *trans*‐[PdBr_2_(^*i*^Pr_2_‐bimy)(THT)] (**42**). However, an excess of PNO was required to afford the desired complex *trans*‐[PdBr_2_(^*i*^Pr_2_‐bimy)(PNO)] (**43**). The two thioethers are expected to have very similar electron donating properties. However, ^13^C NMR spectroscopy could still resolve the small difference as indicated by the HEP values of complexes **41** and **42** at 163.4_6_ and 163.6_3_ ppm, respectively. The cyclic THT ligand was found to be slightly stronger donating than the DMS ligand. The PNO complex **43** shows the most upfield HEP of 155.7_4_ ppm indicating a weak donor property of the PNO ligand, which explains the difficulty met in the preparation of this complex.

**Figure 5:**
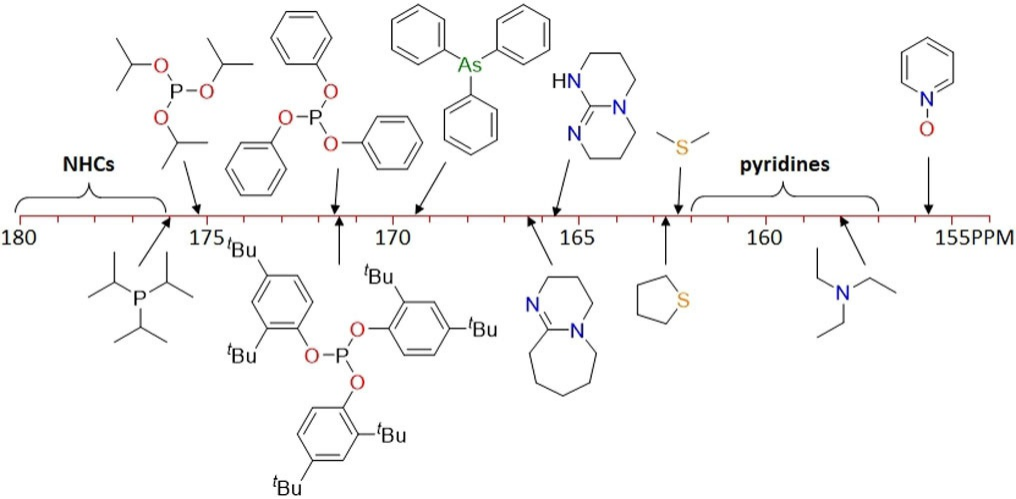
The donor strengths of selected organometallic and Werner‐type ligands on the HEP scale.

Overall, the ligands investigated in this work cover a HEP range of 20 ppm (Figure 5). The phosphites are among the strongest donors, but they are still weaker than their direct phosphine counterparts,^8^ which in turn are inferior to common NHCs in terms of electron donation.^7^ The HEP value of triphenylarsine is smaller than all P‐donors, but significantly larger than all N‐donors. The latter can be differentiated into *sp* ^2^‐ and *sp* ^3^‐hybridized N‐donor atoms and aromaticity of the heterocycle. Generally, *sp* ^2^‐hybridized N‐donors are stronger due to the less hindered lone pair, and aliphatic imines such as DBU and the guanidine TBD are stronger than aromatic pyridines. We also note that the donating ability of pyridines can be fine‐tuned over a range of 5 ppm. Thioethers are found in between aliphatic imines and pyridines, and pyridine‐*N*‐oxide is the weakest donor on the HEP scale thus far.

### Solid‐state molecular structures

Single crystals of complexes **4**, **9**, **15**⋅CH_2_Cl_2_, **19**⋅2 CH_2_Cl_2_, **26**⋅0.5 CHCl_3_, **27**⋅0.5 CHCl_3_⋅0.5 CH_2_Cl_2_, **32**, *cis*‐**35**⋅CH_2_Cl_2_, *cis*‐**36**⋅CHCl_3_, *cis*‐**37**⋅2 CHCl_3_, *cis*‐**39**, *cis*‐**40**, **42** and **43**⋅CHCl_3_ were obtained by slow evaporation of their solutions (see Supporting Information), and X‐ray diffraction reveals square‐planar geometries expected for Pd^II^ complexes. In all cases, the ^*i*^Pr_2_‐bimy ligand is perpendicular to the [PdCBr_2_L] coordination plane.

**Figure 6:**
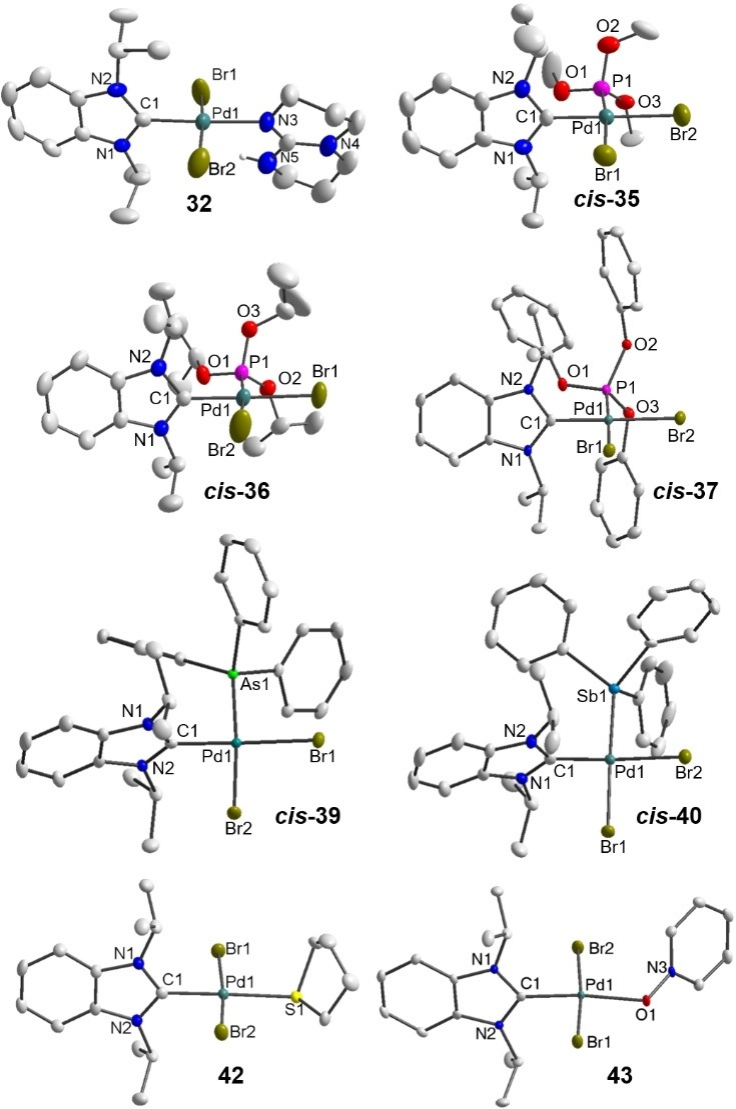
Molecular structures of **32**, *cis*‐**35**⋅CH_2_Cl_2_, *cis*‐**36**⋅CHCl_3_, *cis*‐**37**⋅2CHCl_3_, *cis*‐**39**, *cis*‐**40**, **42** and **43**⋅CHCl_3_ showing 50 % probability ellipsoids; hydrogen atoms and solvent molecules are omitted for clarity. Selected bond length [Å] and bond angles [°]: **32**, Pd1−C1 1.967(4), Pd1−Br1 2.4380(7), Pd1−Br2 2.4341(7), Pd1−N3 2.075(4); C1‐Pd1‐Br1 86.3(1), C1‐Pd1‐Br2 89.2(1), Br1‐Pd1‐N3 93.4(1), Br2‐Pd1‐N3 91.2(1); PdCBr_2_N/NHC dihedral angle *θ* 88.1°. *cis*‐**35**, Pd1−C1 1.980(7), Pd1−Br1 2.4914(9), Pd1−Br2 2.472(1), Pd1−P1 2.204(2); C1‐Pd1‐Br1 84.8(2), C1‐Pd1‐P1 89.7(2), Br1‐Pd1‐Br2 94.21(3), Br2‐Pd1‐P1 91.30(6); *θ* 89.6°. *cis*‐**36**, Pd1−C1 1.988(3), Pd1−Br1 2.4853(4), Pd1−Br2 2.4779(4), Pd1−P1 2.2133(8); C1‐Pd1‐Br2 85.90(8), C1‐Pd1‐P1 90.41(8), Br1‐Pd1‐Br2 92.22(2), Br1‐Pd1‐P1 91.33(2); *θ* 83.8°. *cis*‐**37**, Pd1−C1 1.992(4), Pd1−Br1 2.4751(5), Pd1−Br2 2.4767(5), Pd1−P1 2.204(1); C1‐Pd1‐Br1 88.1(1), C1‐Pd1‐P1 90.4(1), Br1‐Pd1‐Br2 94.64(2), Br2‐Pd1‐P1 86.88(3); *θ* 82.8°. *cis*‐**39**, Pd1−C1 1.970(2), Pd1−Br1 2.4818(3), Pd1−Br2 2.4624(3), Pd1−As1 2.3568(3); C1‐Pd1‐Br2 85.44(6), C1‐Pd1‐As1 90.40(6), Br1‐Pd1‐Br2 93.92(1), Br1‐Pd1‐As1 90.09(1); *θ* 88.4°. *cis*‐**40**, Pd1−C1 1.971(3), Pd1−Br1 2.4699(4), Pd1−Br2 2.4771(4), Pd1−Sb1 2.4967(3); C1‐Pd1‐Br1 86.44(9), C1‐Pd1‐Sb1 95.66(9), Br1‐Pd1‐Br2 95.55(1), Br2‐Pd1‐Sb1 82.49(1); *θ* 84.2°. **42**, Pd1−C1 1.969(3), Pd1−Br1 2.4286(4), Pd1−Br2 2.4342(4), Pd1−S1 2.3784(7); C1‐Pd1‐Br1 86.85(8), C1‐Pd1‐Br2 87.63(8), Br1‐Pd1‐S1 87.13(2), Br2‐Pd1‐S1 98.76(2); *θ* 87.1°. **43**, Pd1−C1 1.935(2), Pd1−Br1 2.4213(3), Pd1−Br2 2.4201(3), Pd1−O1 2.113(2); C1‐Pd1‐Br1 88.42(6), C1‐Pd1‐Br2 86.46(6), Br1‐Pd1‐O1 88.86(4), Br2‐Pd1‐O1 96.39(4); *θ* 88.2°.

The structures of the pyridine adducts **4**, **9**, **15**, **19**, **26** and **27** closely resemble those of previously published analogues^13^ and do not require additional comments (Figure S1). The remaining complexes, on the other hand, are rare examples and their molecular structures are depicted in Figure 6.

Compound **32** is the first mixed NHC/guanidine complex. The N‐heterocycle is also oriented perpendicular to the coordination plane and coordinates via the imine instead of the two amine moieties, confirming once again the superior donating ability of *sp* ^2^‐N‐donors over *sp* ^3^‐hybridized counterparts. As common for N‐ligands, a *trans* arrangement is observed making them ideal candidates for HEP analyses.

In contrast, only *cis*‐isomers of the phosphite adducts **35**–**37** crystallized as a consequence of rapid *trans*–*cis* isomerization due to *transphobia*. Previously reported mixed NHC/phosphite complexes all have a *trans* arrangement, since they contain the very bulky NHCs IPr and SIPr.^25^ Compared to these, the averaged Pd–C separation of 1.987 Å in our *cis* complexes is significantly shorter indicating stronger bonds. They are also identical within error margin. The *cis*‐configuration also leads to shorter and stronger Pd−P bonds with an average length of 2.207 Å.

Similar observations were made for the arsine and stibine complexes *cis*‐**39** and *cis*‐**40**, which are the first structurally characterized *cis*‐isomers. The Pd−C bonds of 1.970(2) and 1.971(3) are identical and equal to that observed for the *cis*‐[PdBr_2_(^*i*^Pr_2_‐bimy)(PPh_3_)]^12^ complex within 3*σ*. On the other hand, bonds to palladium steadily elongate going from phosphorus {2.2624(8) Å}^12^ to arsenic {2.3568(3) Å} to antimony {2.4967(3) Å}, which is due to the increasing atom size going down the group. All these bonds are significantly shorter and stronger than those observed in related *trans* complexes.^35^

Complexes **42** and **43** are also the first of their kind. The thioether and *N*‐oxide donors adopt preferably a *trans* orientation with respect to the NHC. The Pd–C distance for the thioether complex of 1.969(3) Å is substantially longer than that in the pyridine‐*N*‐oxide complex Pd1‐C1 1.935(2) Å, which is reflective of their different donor strengths. The latter is equal to that observed for complex formed with acetonitrile,^12^ which is also a very weak donor.

## Conclusions

The study of 26 complex probes of the type *trans*‐[PdBr_2_(^*i*^Pr_2_‐bimy)(Py‐R)] using the Huynh electronic parameter (HEP) have revealed remote substituent effects in *meta*‐ and *para*‐substituted pyridines five and six bonds away from the NMR probe. Moreover, the influence of these substituents on the ^13^C_carbene_ NMR signal of the ^*i*^Pr_2_‐bimy reporter ligand shows an excellent correlation to their respective sigma Hammett constants (*σ*_m_ and *σ*_p_), providing further support for the suitability of HEP as an efficient and accurate electronic parameter. The resulting regression equations could also be used for the re‐evaluation of certain Hammett constants that carry larger uncertainties. This has been demonstrated for the CO_2_H and CO_2_M (M=alkali metal) substituents. In addition HEPs of *meta*‐ and *para*‐substituted pyridines bearing the same substituent also showed an excellent linear correlation. In principle, this allows for HEP predictions of *para*‐substituted pyridines from that of the respective *meta*‐isomer and vice versa. Finally, attempts were made to extend the library of HEP values to various ligands that hitherto have not been covered yet. In particular, these include phosphites, arsines, stibines, thioethers and N‐oxides. The mixed NHC/ligand complexes of the pnictogens show an interesting and electronically‐driven *trans*–*cis* isomerization. The so‐called “*transphobia*” effect previously described for phosphorous ligands have thus been extended to include arsenic and antimony compounds. In addition to reporting new types of complexes, this work also expands the toolbox for chemists and provides valuable electronic data for the proper comparison of different ligand classes on a unified scale.

## Supporting Information

Supporting Information for this article is given via a link at the end of the document. The Supporting Information contains a detailed description of the synthetic work, the characterization of all analytes as well as selected crystallographic data.

## Conflict of interest

The authors declare no conflict of interest.

## Supporting information

As a service to our authors and readers, this journal provides supporting information supplied by the authors. Such materials are peer reviewed and may be re‐organized for online delivery, but are not copy‐edited or typeset. Technical support issues arising from supporting information (other than missing files) should be addressed to the authors.

Supplementary
