# Supplementary material for: Donor Strengths Determination of Pnictogen and Chalcogen Ligands by the Huynh Electronic Parameter and Its Correlation to Sigma Hammett Constants
Source: Chemistry. 2019 Sep 30;25(61):13956–63. doi: 10.1002/chem.201902795 (PMC6900057; doi:10.1002/chem.201902795)
Supplement: Supplementary file 1 — Supplementary [file CHEM-25-13956-s001.pdf]

CHEMISTRY
A European Journal

Supporting Information

Donor Strengths Determination of Pnictogen and Chalcogen Ligands by the Huynh Electronic Parameter and Its Correlation to Sigma Hammett Constants

Qiaoqiao Teng,[a, b] Ping Siang Ng,[a] Jia Nuo Leung,[a] and Han Vinh Huynh*[a]

chem_201902795_sm_miscellaneous_information.pdf

Table of Contents

Experimental Section 2

Molecular Structures of **4**, **9**, **15**, **19**, **26** and **27** 21

Selected X-ray Crystallographic data 22

NMR Spectra 25

Experimental Section:

General procedure for preparation of *trans*-[$PdBr_2(^iPr_2$-bimy)(Py-R)] complexes.

A mixture of complex **I** (47 mg, 0.05 mmol) and the appropriate pyridine (0.10 mmol) was suspended in dichloromethane (4-15 mL) and stirred for 2 h at ambient temperature. The volatiles were removed under reduced pressure, and the residue washed with a small amount of diethyl ether to give the products as yellow solids.

***trans*-Dibromido(3-cyanopyridine)(1,3-diisopropylbenzimidazolin-2-ylidene)palladium(II) (2).**

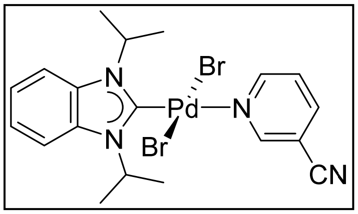

Yield: 60 mg (0.10 mmol, >99%). ^{1}H NMR (300 MHz, $CDCl_3$): δ 9.53 (d, 1 H, py−H), 9.43 (dd, 1 H, py−H), 8.07 (m, 1 H, py−H), 7.59 (dd, 2 H, Ar−H), 7.53 (m, 1 H, py−H), 7.23 (dd, 2 H, Ar−H), 6.25 (m, ^3J(H,H) = 7.1 Hz, 2 H, NCH), 1.77 (d, ^3J(H,H) = 7.1 Hz, 12 H, CH_3). $^{13}C\{^1H\}$ NMR (75 MHz, $CDCl_3$): δ 157.3$_3$ (HEP), 156.7, 156.0, 141.7, 134.0, 125.4, 123.1, 115.6, 113.3, 111.7 (Ar−C & CN), 55.3 (NCH), 21.1 (CH_3). Anal. Calc. for $C_{19}H_{22}Br_2N_4Pd$: C, 39.85; H, 3.87; N, 9.78. Found: C, 40.02; H, 3.87; N, 10.06. MS (ESI): *m/z* = 499 [M – $C_6H_4N_2$ + OMe]$^-$.

***trans*-Dibromido(1,3-diisopropylbenzimidazolin-2-ylidene)(3-nitropyridine)-palladium(II) (3).**

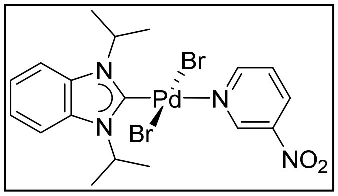

Yield: 58 mg (0.10 mmol, 98%). ^{1}H NMR (500 MHz, $CDCl_3$): δ 10.10 (d, 1 H, py−H), 9.55 (dd, 1 H, py−H), 8.62 (m, 1 H, py−H), 7.61 (m, 3 H, py−H and Ar−H), 7.24 (dd, 2 H, Ar−H), 6.28 (m, ^3J(H,H) = 7.1 Hz, 2 H, NCH, 1.80 (d, ^3J(H,H) = 7.1 Hz, 12 H, CH_3). $^{13}C\{^1H\}$ NMR (126 MHz, $CDCl_3$): δ 158.3 (s, py−NCN), 157.3$_8$ (HEP), 149.4, 145.2, 134.1, 133.6, 125.3, 123.2, 113.4 (Ar-C), 55.5 (NCH), 21.3 (CH_3). Anal. Calc. for $C_{18}H_{22}Br_2N_4O_2Pd$: C, 36.48; H, 3.74; N, 9.45. Found: C, 36.81; H, 3.71; N, 9.66. MS (ESI): *m/z* = 571 [M – Br + CH_3COCH_3]$^+$.

trans-Dibromido(1,3-diisopropylbenzimidazolin-2-ylidene)(3-pyridinecarboxaldehyde)-palladium(II) (4).

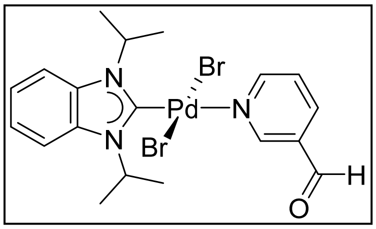

Slow evaporation of a chloroform solution of the crude product afforded the pure product as yellow crystals. Yield: 60 mg (0.10 mmol, >99%). ^{1}H NMR (300 MHz, $CDCl_3$): δ 10.14 (s, 1 H, CHO), 9.67 (d, 1 H, py−H), 9.43 (dd, 1 H, py−H), 8.29 (m, 1 H, py−H), 7.60 (dd, 2 H, Ar−H), 7.56 (m, 1 H, py−H), 7.23 (dd, 2 H, Ar−H), 6.31 (m, ^3J(H,H) = 7.1 Hz, 2 H, NCH, 1.80 (d, ^3J(H,H) = 7.1 Hz, 12 H, CH_3). $^{13}C\{^1H\}$ NMR (75 MHz, $CDCl_3$): δ 189.5 (s, CHO), 158.5$_4$ (HEP), 158.1, 156.1, 137.6, 134.2, 132.9, 125.7, 123.1, 113.4 (Ar-C), 55.4 (NCH), 21.3 (CH_3). Anal. Calc. for $C_{19}H_{23}Br_2N_3OPd$: C, 39.64; H, 4.03; N, 7.30. Found: C, 39.18; H, 3.84; N, 7.32. MS (ESI): *m/z* = 509 [M – 2 Br + 2OMe + MeOH]$^+$.

trans-Dibromido(1,3-diisopropylbenzimidazolin-2-ylidene)(3-bromopyridine)palladium(II) (5).

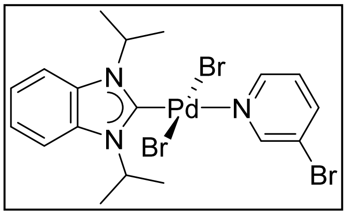

Yield: 60 mg (0.10 mmol, 96%). ^{1}H NMR (500 MHz, $CDCl_3$): δ 9.26 (d, 1 H, Py–H), 9.11 (m, 1 H, Py–H), 7.93–7.91 (m, 1 H, Py–H), 7.59 (dd, 2 H, Ar–H), 7.28–7.25 (m, 1 H, Py–H), 7.22 (dd, 2 H, Ar–H), 6.29 (m, ^3J(H,H) = 7.0 Hz, 2 H, NCH), 1.79 (d, ^3J(H,H) = 7.0 Hz, 12 H, CH_3). $^{13}C\{^1H\}$ NMR (126 MHz, $CDCl_3$): 158.6$_3$ (HEP), 154.4, 151.7, 141.5, 134.1, 125.9, 123.0, 121.3, 113.3 (Ar–C), 55.3 (NCH), 21.2 (CH_3). Anal. Calcd for $C_{18}H_{22}Br_3N_3Pd$: C, 34.51; H, 3.54; N, 6.71. Found: C, 34.75; H, 3.35; N, 6.75. MS (ESI): *m/z* 469 [M – Py + H]$^+$.

trans-Dibromido(1,3-diisopropylbenzimidazolin-2-ylidene)(3-iodopyridine)palladium(II) (6).

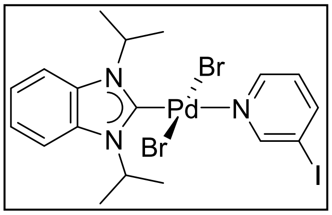

Yield: 45 mg (0.07 mmol, 67%). ^{1}H NMR (500 MHz, $CDCl_3$): δ 9.36 (s, 1 H, Py–H), 9.12 (d, 1 H, Py–H), 8.09 (d, 1 H, Py–H), 7.59 (dd, 2 H, Ar–H), 7.22 (dd, 2 H, Ar–H), 7.16–7.13 (m, 1 H, Py–H), 6.29 (m, ^3J(H,H) = 7.0 Hz, 2 H, NCH), 1.79 (d, ^3J(H,H) = 7.0 Hz, 12 H, CH_3). $^{13}C\{^1H\}$ NMR (126 MHz, $CDCl_3$): 159.0 (Ar–C), 158.6$_8$ (HEP), 152.0, 147.0, 134.1, 126.3, 123.0, 113.3, 92.9 (Ar–C), 55.3 (NCH), 21.2 (CH_3). Anal. Calcd for $C_{18}H_{22}Br_2IN_3Pd$: C, 32.10; H, 3.29; N, 6.24. Found: C, 32.23; H, 3.21; N, 6.27. MS (ESI): *m/z* 469 [M – Py + H]$^+$.

***trans*-Dibromido(1,3-diisopropylbenzimidazolin-2-ylidene)(3-chloropyridine)palladium(II) (7).**

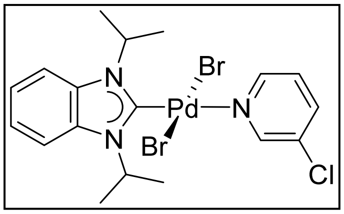

Yield: 58 mg (0.10 mmol, >99%). ^{1}H NMR (500 MHz, $CDCl_3$): δ 9.18 (d, 1 H, Py–H), 9.07 (d, 1 H, Py–H), 7.77–7.76 (m, 1 H, Py–H), 7.59 (dd, 2 H, Ar–H), 7.33–7.30 (m, 1 H, Py–H), 7.22 (dd, 2 H, Ar–H), 6.30 (m, ^{3}J(H,H) = 7.0 Hz, 2 H, NCH), 1.79 (d, ^{3}J(H,H) = 7.0 Hz, 12 H, CH_3). $^{13}C\{^{1}H\}$ NMR (126 MHz, $CDCl_3$): δ 158.7$_2$ (HEP), 152.4, 151.4, 138.6, 134.1, 133.3, 125.5, 123.0, 113.3 (Ar–C), 55.3 (NCH), 21.2 (CH_3). Anal. Calcd for $C_{18}H_{22}Br_2ClN_3Pd$: C, 37.14; H, 3.81; N, 7.22. Found: C, 37.31; H, 3.80; N, 7.22. MS (ESI): *m/z* 469 [M – Py + H]$^+$.

***trans*-Dibromido(1,3-diisopropylbenzimidazolin-2-ylidene)(3-fluoropyridine)palladium(II), (8).**

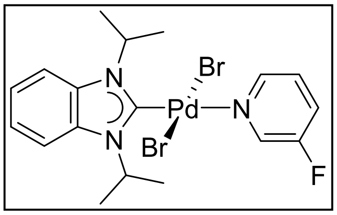

Yield: 55 mg (0.10 mmol, 97%). ^{1}H NMR (500 MHz, $CDCl_3$): δ 9.12 (t, 1 H, Py–H), 9.02 (d, 1 H, Py–H), 7.58 (dd, 2 H, Ar–H), 7.55–7.52 (m, 1 H, Py–H), 7.39–7.35 (m, 1 H, Py–H), 7.22 (dd, 2 H, Ar–H), 6.30 (m, ^{3}J(H,H) = 7.0 Hz, 2 H, NCH), 1.79 (d, ^{3}J(H,H) = 7.0 Hz, 12 H, CH_3). $^{13}C\{^{1}H\}$ NMR (126 MHz, $CDCl_3$): 159.7 (d, ^{1}J(C,F) = 253.9 Hz, Py–C), 158.7$_3$ (HEP), 149.6 (d, ^{3}J(C,F) = 4.6 Hz, Py–C), 142.5 (d, ^{2}J(C,F) = 30.2 Hz, Py–C), 134.1 (Ar–C), 125.8 (d, ^{2}J(C,F) = 12.8 Hz), 125.7, 123.0, 113.3 (Ar–C), 55.3 (NCH), 21.2 (CH_3). $^{19}F\{^{1}H\}$ NMR (282 MHz, $CDCl_3$): –46.3 (F). Anal. Calcd for $C_{18}H_{22}Br_2FN_3Pd$: C, 38.22; H, 3.92; N, 7.43. Found: C, 38.36; H, 3.67; N, 7.38. MS (ESI): *m/z* 469 [M – Py + H]$^+$.

***trans*-Dibromido(1,3-diisopropylbenzimidazolin-2-ylidene)(3-pyridinecarboxylic acid)palladium(II) (9).**

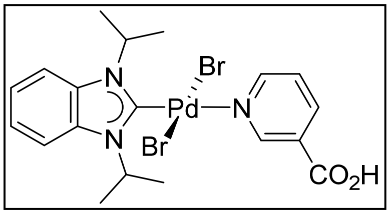

Slow evaporation of a pentane/dichloromethane solution of the crude product afforded the pure product as yellow crystals. Yield: 60 mg (0.10 mmol, >99%). ^{1}H NMR (300 MHz, $CDCl_3$): δ 9.81 (s, 1 H, py−H), 9.37 (d, 1 H, py−H), 8.45 (d, 1 H, py−H), 7.60 (dd, 2 H, Ar−H), 7.51 (m, 1 H, py−H), 7.22 (dd, 2 H, Ar−H), 6.33 (m, ^{3}J(H,H) = 7.1 Hz, 2 H, NCH), 1.80 (d, ^{3}J(H,H) = 7.1 Hz, 12 H, CH_3). The COOH signal could not be detected. $^{13}C\{^{1}H\}$ NMR

(75 MHz, $CDCl_3$): δ 168.5, (s, COOH), 159.0$_4$ (HEP), 157.3, 155.2, 140.0, 134.2, 127.3, 125.0, 123.0, 113.3 (Ar-C), 55.4 (NCH), 21.3 (CH_3). Anal. Calc. for $C_{19}H_{23}Br_2N_3O_2Pd$: C, 38.57; H, 3.92; N, 7.10. Found: C, 38.67; H, 3.97; N, 6.95. MS (ESI): *m/z* = 590 $[M - H]^-$.

***trans*-Dibromido(1,3-diisopropylbenzimidazolin-2-ylidene)(3-hydroxopyridine)-palladium(II) (10).**

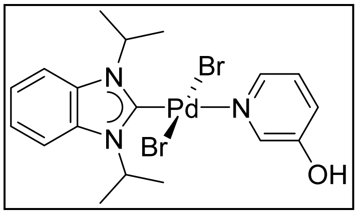

Yield: 58 mg (0.10 mmol, >99%). ^{1}H NMR (500 MHz, $CDCl_3$): δ 8.63 (d, 1 H, Py–H), 8.54 (d, 1 H, Py–H), 7.59 (dd, 2 H, Ar–H), 7.23 (dd, 2 H, Ar–H), 7.18–7.15 (m, 1 H, Py–H), 7.09–7.06 (m, 1 H, Py–H), 6.32 (m, ^3J(H,H) = 7.0 Hz, 2 H, NCH), 1.80 (d, ^3J(H,H) = 7.0 Hz, 12 H, CH_3). $^{13}C\{^1H\}$ NMR (126 MHz, $CDCl_3$): δ 159.6$_0$ (HEP), 153.6, 145.1, 141.9, 134.2, 125.27, 125.25, 123.0, 113.3 (Ar–C), 55.3 (NCH), 21.3 (CH_3). Anal. Calcd for $C_{18}H_{23}Br_2N_3OPd$: C, 38.36; H, 4.11; N, 7.46. Found: C, 38.52; H, 3.90; N, 7.37. MS (ESI): *m/z* 525 $[M - Br + CH_3CN]^+$.

***trans*-Dibromido(1,3-diisopropylbenzimidazolin-2-ylidene)(3-phenylpyridine)palladium(II) (11).**

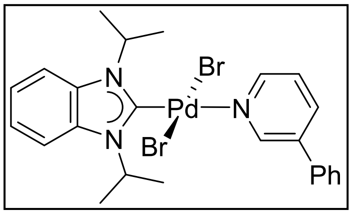

Yield: 62 mg (0.10 mmol, 99%). ^{1}H NMR (500 MHz, $CDCl_3$): δ 9.39 (d, 1 H, Py–H), 9.12 (dd, 1 H, Py–H), 7.95 (d, 1 H, Py–H), 7.62–7.59 (m, 4 H, Ar–H & Py–H), 7.49 (t, 2 H, Ar–H), 7.44–7.41 (m, 2 H, Ar–H), 7.21 (dd, 2 H, Ar–H), 6.37 (m, ^3J(H,H) = 7.0 Hz, 2 H, NCH), 1.81 (d, ^3J(H,H) = 7.0 Hz, 12 H, CH_3). $^{13}C\{^1H\}$ NMR (126 MHz, $CDCl_3$): δ 159.8$_0$ (HEP), 152.0, 151.7, 138.4, 137.1, 136.8, 134.1, 129.8, 129.3, 127.9, 124.9, 122.9, 113.2 (Ar–C), 55.2 (NCH), 21.2 (CH_3). Anal. Calcd for $C_{24}H_{27}Br_2N_3Pd$: C, 46.22; H, 4.36; N, 6.74. Found: C, 45.99; H, 4.02; N, 6.60. MS (ESI): *m/z* 697 $[M + CH_3CN + MeOH + H]^+$.

***trans*-Dibromido(3-ethylpyridine)(1,3-diisopropylbenzimidazolin-2-ylidene)palladium(II) (12).**

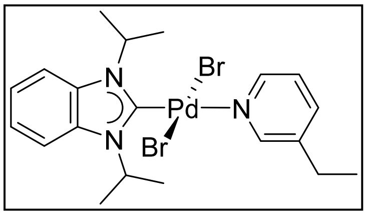

Yield: 60 mg (0.10 mmol, >99%). ^{1}H NMR (300 MHz, $CDCl_3$): δ 8.94−8.92 (m, 2 H, py−H), 7.61−7.55 (m, 3 H, py−H and Ar−H), 7.28−7.25 (m, 1 H, py−H), 7.22−7.19 (dd, 2 H, Ar−H), 6.35 (m, ^{3}J(H,H) = 7.1 Hz, 2 H, NCH), 2.69 (q, ^{3}J(H,H) = 7.6 Hz, 2 H, CH_2CH_3), 1.79 (d, ^{3}J(H,H) = 7.1 Hz, 12 H, CH_3), 1.27 (t, ^{3}J(H,H) = 7.6 Hz, 3 H, CH_2CH_3). ^{13}C{^{1}H} NMR (75 MHz, $CDCl_3$): δ 160.3_3 (HEP), 152.9, 150.7, 141.1, 137.9, 134.1, 124.7, 122.9, 113.2 (Ar−C), 55.2 (NCH), 26.7 (CH_2CH_3), 21.2 (CH_3), 15.7 (CH_2CH_3). Anal. Calc. for $C_{20}H_{27}Br_2N_3Pd$: C, 41.73; H, 4.73; N, 7.30. Found: C, 41.86; H, 4.77; N, 7.43. MS (ESI): *m/z* = 603 [M – Br + $C_7H_9N]^+$.

***trans*-Dibromido(1,3-diisopropylbenzimidazolin-2-ylidene)(3-methylpyridine)palladium(II) (13).**

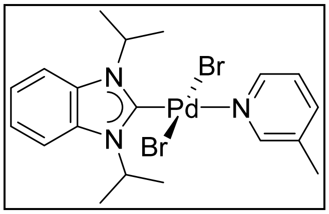

Yield: 60 mg (0.10 mmol, >99%). ^{1}H NMR (300 MHz, $CDCl_3$): δ 8.91–8.89 (m, 2 H, py−H), 7.60–7.57 (m, 3 H, py−H and Ar−H), 7.23–7.19 (m, 3 H, py−H and Ar−H), 6.34 (m, ^{3}J(H,H) = 7.1 Hz, 2 H, NCH), 2.37 (s, 3 H, py-CH_3), 1.79 (d, ^{3}J(H,H) = 7.1 Hz, 12 H, CH_3). ^{13}C{^{1}H} NMR (75 MHz, $CDCl_3$): δ 160.4_1 (HEP), 153.4, 150.5, 139.1, 135.2, 134.1, 124.6, 122.9, 113.3 (Ar−C), 55.2 (NCH), 21.3 (CH_3), 19.1 (py-CH_3). Anal. Calc. for $C_{19}H_{25}Br_2N_3Pd$: C, 40.63; H, 4.49; N, 7.48. Found: C, 40.43; H, 4.59; N, 7.49. MS (ESI): *m/z* = 575 [M – Br + $C_6H_7N]^+$.

***trans*-Dibromido(3-aminopyridine)(1,3-diisopropylbenzimidazolin-2-ylidene)palladium(II) (14).**

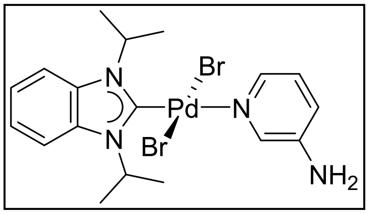

Yield: 60 mg (0.10 mmol, >99%). ^{1}H NMR (300 MHz, $CDCl_3$): δ 8.51 (d, 1 H, py−H), 8.44 (dd, 1 H, py−H), 7.57 (dd, 2 H, Ar−H), 7.20 (dd, 2 H, Ar−H), 7.04 (d, 1 H, py−H), 7.00 (dd, 1 H, py−H), 6.33 (m, ^{3}J(H,H) = 7.1 Hz, 2 H, NCH), 3.86 (br, 2 H, NH_2), 1.78 (d, ^{3}J(H,H) = 7.1 Hz, 12 H, CH_3). ^{13}C{^{1}H} NMR (75 MHz, $CDCl_3$): δ 160.4_7 (HEP), 143.9, 143.1, 140.5, 134.1, 125.1, 123.6, 122.9, 113.2 (Ar−C), 55.2 (NCH), 21.3 (CH_3). Anal. Calc. for

$C_{18}H_{24}Br_2N_4Pd$: C, 38.42; H, 4.30; N, 9.96. Found: C, 40.06; H, 4.65; N, 11.14. MS (ESI): *m/z* = 577 [M – Br + $C_5H_6N_2$]$^+$.

trans-Dibromido(4-cyanopyridine)(1,3-diisopropylbenzimidazolin-2-ylidene)palladium(II) (15).

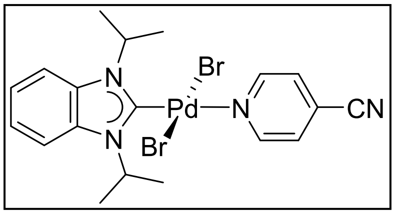

Slow evaporation of a pentane/dichloromethane solution of the crude product afforded the pure product as yellow crystals. Yield: 60 mg (0.10 mmol, >99%). ^{1}H NMR (300 MHz, $CDCl_3$): δ 9.43–9.40 (m, 2 H, py−H), 7.64–7.58 (m, 4 H, py−H and Ar−H), 7.26–7.22 (dd, 2 H, Ar−H), 6.25 (m, ^3J(H,H) = 7.1 Hz, 2 H, NCH), 1.78 (d, ^3J(H,H) = 7.1 Hz, 12 H, CH_3). $^{13}C\{^1H\}$ NMR (75 MHz, $CDCl_3$): δ 157.7$_9$ (HEP), 154.5, 151.0, 134.1, 126.9, 126.2 123.1, 113.4 (Ar−C & CN), 55.4 (NCH), 21.2 (CH_3). Anal. Calc. for $C_{19}H_{22}Br_2N_4Pd$: C, 39.85; H, 3.87; N, 9.78. Found: C, 39.55; H, 3.54; N, 9.38. MS (ESI): *m/z* = 499 [M – $C_6H_4N_2$ + OMe]$^-$.

trans-Dibromido(1,3-diisopropylbenzimidazolin-2-ylidene)(4-trifluoromethylpyridine)-palladium(II) (16).

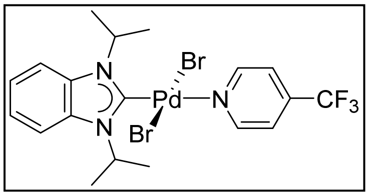

Yield: 60 mg (0.10 mmol, 98%). ^{1}H NMR (500 MHz, $CDCl_3$): δ 9.40 (d, 2 H, py−H), 7.60 (m, 4 H, Ar−H and py−H), 7.22 (dd, 2 H, Ar−H), 6.29 (m, ^3J(H,H) = 7.1 Hz, 2 H, NCH), 1.79 (d, ^3J(H,H) = 7.1 Hz, 12 H, CH_3). $^{13}C\{^1H\}$ NMR (126 MHz, $CDCl_3$): δ 158.3$_8$ (HEP), 154.6, 134.1, 123.0 (Ar–C), 121.1 (q, $^3J(^{13}C,^{19}F)$ = 3.4 Hz), 113.3 (Ar-C), 55.3 (NCH), 21.2 (CH_3) ($\mathit{C}F_3$ and C-CF_3 peaks not observed). $^{19}F\{^1H\}$ NMR (376 MHz, $CDCl_3$): –65.16. Anal. Calc. for $C_{19}H_{22}Br_2F_3N_3Pd$: C, 37.07; H, 3.60; N, 6.83. Found: C, 35.70; H, 3.52; N, 6.60. MS (ESI): *m/z* = 549 [M – $C_6H_4F_3N$ + Br]$^-$.

trans-Dibromido(1,3-diisopropylbenzimidazolin-2-ylidene)(4-pyridinecarboxaldehyde)-palladium(II) (17).

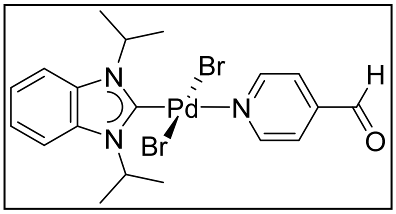

Yield: 60 mg (0.10 mmol, >99%). ^{1}H NMR (300 MHz, $CDCl_3$): δ 10.11 (s, 1 H, CHO), 9.43 (m, 2 H, py−H), 7.77 (m, 2 H, py−H), 7.59 (dd, 2 H, Ar−H), 7.22 (dd, 2 H, Ar−H), 6.30 (m, ^3J(H,H) = 7.1 Hz, 2 H, NCH), 1.79 (d, ^3J(H,H) = 7.1 Hz, 12 H, CH_3). $^{13}C\{^1H\}$

NMR (75 MHz, $CDCl_3$): δ 190.7 (CHO), 158.7$_6$ (HEP), 155.0, 142.7, 134.1, 123.6, 123.0, 113.3 (Ar–C), 55.3 (NCH), 21.2 (CH_3). Anal. Calc. for $C_{19}H_{23}Br_2N_3OPd$: C, 39.64; H, 4.03; N, 7.30. Found: C, 39.90; H, 3.54; N, 7.35. MS (ESI): *m/z* = 592 $[M + OH]^-$.

***trans*-Dibromido(1,3-diisopropylbenzimidazolin-2-ylidene)(4-chloropyridine)-palladium(II) (18)**.

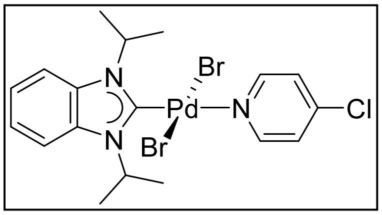

Yield: 47 mg (0.08 mmol, 80%). ^1H NMR (500 MHz, $CDCl_3$): δ 9.08 (d, 2 H, Py–H), 7.58 (dd, 2 H, Ar–H), 7.36 (d, 2 H, Py–H), 7.21 (dd, 2 H, Ar–H), 6.29 (m, ^3J(H,H) = 7.0 Hz, 2 H, NCH), 1.78 (d, ^3J(H,H) = 7.0 Hz, 12 H, CH_3). $^{13}C\{^1H\}$ NMR (126 MHz, $CDCl_3$): δ 158.9$_8$ (HEP), 154.1, 153.4, 147.2, 134.1, 125.6, 122.9, 113.2 (Ar–C), 55.2 (NCH), 21.2 (CH_3). Anal. Calcd for $C_{18}H_{22}Br_2ClN_3Pd$: C, 37.14; H, 3.81; N, 7.22. Found: C, 37.21; H, 3.72; N, 7.14. MS (ESI): *m/z* 469 $[M – Py + H]^+$.

***trans*-Dibromido(1,3-diisopropylbenzimidazolin-2-ylidene)(4-pyridinecarboxylic acid)-palladium(II) (19).**

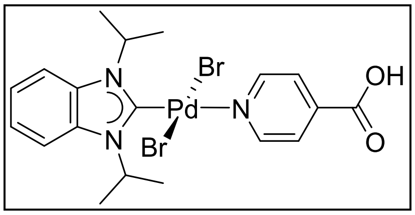

Slow evaporation of a dichloromethane solution of the crude product afforded the pure product as yellow crystals. Yield: 60 mg (0.10 mmol, >99%). ^1H NMR (300 MHz, $CDCl_3$): δ 9.36 (d, 2 H, py−H), 7.95 (d, 2 H, py−H), 7.60 (dd, 2 H, Ar−H), 7.23 (dd, 2 H, Ar−H), 6.31 (m, ^3J(H,H) = 7.1 Hz, 2 H, NCH), 1.80 (d, ^3J(H,H) = 7.1 Hz, 12 H, CH_3). The COOH signal could not be detected. $^{13}C\{^1H\}$ NMR (75 MHz, $CDCl_3$): δ 168.2 (COOH), 159.0$_0$ (HEP), 154.4, 138.9, 134.2, 124.8, 123.0, 113.3 (Ar−C), 55.3 (NCH), 21.3 (CH_3). Anal. Calc. for $C_{19}H_{23}Br_2N_3O_2Pd$: C, 38.57; H, 3.92; N, 7.10. Found: C, 38.63; H, 3.85; N, 7.04. MS (ESI): *m/z* = 635 $[M – Br + C_6H_5NO_2]^+$.

***trans*-Dibromido(1,3-diisopropylbenzimidazolin-2-ylidene)(4-bromopyridine)palladium(II) (20).**

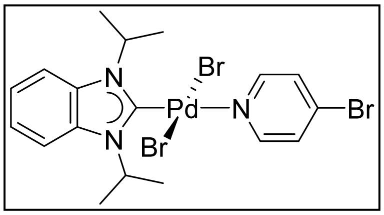

Yield: 56 mg (0.09 mmol, 89%). ^{1}H NMR (500 MHz, $CDCl_3$): δ 8.94 (d, 2 H, Py–H), 7.58 (dd, 2 H, Ar–H), 7.53 (d, 2 H, Py–H), 7.21 (dd, 2 H, Ar–H), 6.29 (m, ^{3}J(H,H) = 7.0 Hz, 2 H, NCH), 1.78 (d, ^{3}J(H,H) = 7.0 Hz, 12 H, CH_3). $^{13}C\{^{1}H\}$ NMR (126 MHz, $CDCl_3$): δ 159.0$_3$ (HEP), 153.9, 136.1, 134.1, 128.7, 123.0, 113.3 (Ar–C), 55.2 (NCH), 21.2 (CH_3). Anal. Calcd for $C_{18}H_{22}Br_3N_3Pd$: C, 34.51; H, 3.54; N, 6.71. Found: C, 34.52; H, 3.59; N, 6.27 (the best result obtained). MS (ESI): *m/z* 469 $[M - Py + H]^+$.

***trans*-Dibromido(1,3-diisopropylbenzimidazolin-2-ylidene)(4-iodopyridine)palladium(II) (21).**

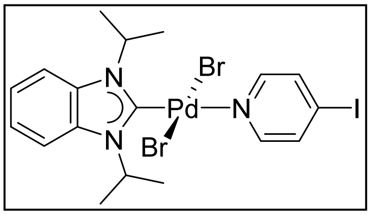

Yield: 47 mg (0.07 mmol, 70%). ^{1}H NMR (500 MHz, $CDCl_3$): δ 8.79 (d, 2 H, Py–H), 7.73 (d, 2 H, Py–H), 7.58 (dd, 2 H, Ar–H), 7.21 (dd, 2 H, Ar–H), 6.28 (m, ^{3}J(H,H) = 7.0 Hz, 2 H, NCH), 1.78 (d, ^{3}J(H,H) = 7.0 Hz, 12 H, CH_3). $^{13}C\{^{1}H\}$ NMR (126 MHz, $CDCl_3$): δ 159.2$_1$ (HEP), 153.3, 134.7, 134.1, 123.0, 113.3, 109.0 (Ar–C), 55.2 (NCH), 21.2 (CH_3). Anal. Calcd for $C_{18}H_{22}Br_2IN_3Pd$: C, 32.10; H, 3.29; N, 6.24. Found: C, 32.48; H, 3.25; N, 6.14. MS (ESI): *m/z* 469 $[M - Py + H]^+$.

***trans*-Dibromido(1,3-diisopropylbenzimidazolin-2-ylidene)(4-phenylpyridine)palladium(II) (22).**

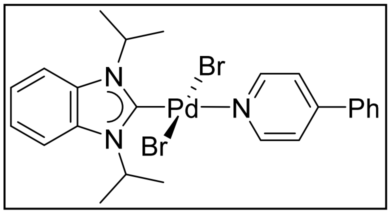

Yield: 54 mg (0.09 mmol, 87%). ^{1}H NMR (500 MHz, $CDCl_3$): δ 9.16 (d, 2 H, Py–H), 7.63–7.60 (m, 4 H, Ar–H), 7.56–7.55 (d, 2 H, Py–H), 7.49–7.48 (m, 3 H, Ar–H), 7.22 (br–s, 2 H, Ar–H), 6.38 (m, ^{3}J(H,H) = 7.0 Hz, 2 H, NCH), 1.81 (d, ^{3}J(H,H) = 7.0 Hz, 12 H, CH_3). $^{13}C\{^{1}H\}$ NMR (126 MHz, $CDCl_3$): δ 160.1$_6$ (HEP), 153.4, 150.8, 137.3, 134.1, 130.5, 129.9, 127.7, 122.9, 122.8, 113.2 (Ar–C), 55.2 (NCH), 21.2 (CH_3). Anal. Calcd for $C_{24}H_{27}Br_2N_3Pd$:

C, 46.22; H, 4.36; N, 6.74. Found: C, 46.60; H, 3.99; N, 6.72. MS (ESI): *m/z* 697 [M + CH_3CN + MeOH + H]$^+$.

trans-Dibromido(1,3-diisopropylbenzimidazolin-2-ylidene)(4-methoxypyridine)palladium-(II) (23).

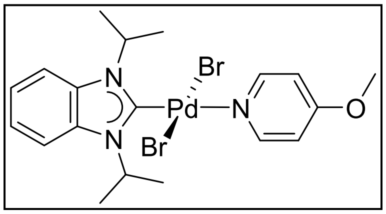

Yield: 60 mg (0.10 mmol, >99%). ^{1}H NMR (300 MHz, $CDCl_3$): δ 8.92 (m, 2 H, py−H), 7.58 (dd, 2 H, Ar−H), 7.20 (dd, 2 H, Ar−H), 6.82 (dd, 2 H, py−H), 6.33 (m, ^3J(H,H) = 7.1 Hz, 2 H, NCH), 3.86 (s, 3 H, OCH_3), 1.78 (d, ^3J(H,H) = 7.1 Hz, 12 H, CH_3). $^{13}C\{^1H\}$ NMR (75 MHz, $CDCl_3$): δ 167.3 (Ar−*C*OCH_3), 160.3$_9$ (HEP), 154.4, 134.1, 122.8, 113.2, 111.2 (Ar−C), 56.4 (OCH_3), 55.1 (NCH), 21.2 (CH_3). Anal. Calc. for $C_{19}H_{25}Br_2N_3OPd$: C, 39.51; H, 4.36; N, 7.27. Found: C, 39.42; H, 4.17; N, 7.34. MS (ESI): *m/z* = 607 [M – Br + C_6H_7NO]$^+$.

trans-Dibromido(1,3-diisopropylbenzimidazolin-2-ylidene)(4-methylpyridine)palladium(II) (24).

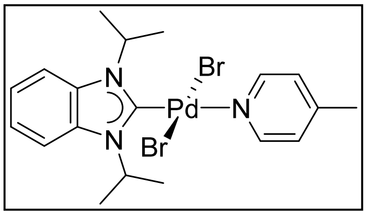

Yield: 60 mg (0.10 mmol, >99%). ^{1}H NMR (300 MHz, $CDCl_3$): δ 8.93 (m, 2 H, py-H), 7.55 (dd, 2 H, Ar−H), 7.20 (dd, 2 H, Ar−H), 7.15 (d, 2 H, py−H), 6.33 (m, ^3J(H,H) = 7.1 Hz, 2 H, NCH), 2.37 (s, 3 H, py−CH_3), 1.78 (d, ^3J(H,H) = 7.1 Hz, 12 H, CH_3). $^{13}C\{^1H\}$ NMR (75 MHz, $CDCl_3$): δ 160.4$_3$ (HEP), 152.7, 150.5, 134.1, 126.0, 122.8, 113.2 (Ar−C), 55.2 (NCH), 21.8 (py−CH_3), 21.2 (CH_3). Anal. Calc. for $C_{19}H_{25}Br_2N_3Pd$: C, 40.63; H, 4.49; N, 7.48. Found: C, 40.90; H, 4.53; N, 7.63. MS (ESI): *m/z* = 575 [M – Br + C_6H_7N]$^+$.

trans-Dibromido(1,3-diisopropylbenzimidazolin-2-ylidene)(4-ethylpyridine)palladium(II) (25).

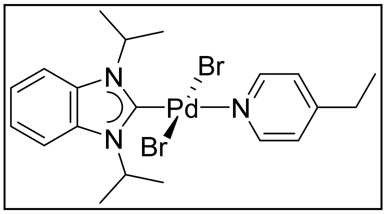

Yield: 60 mg (0.10 mmol, >99%). ^{1}H NMR (300 MHz, $CDCl_3$): δ 8.96 (m, 2 H, py−H), 7.58 (dd, 2 H, Ar−H), 7.19 (m, 4 H, Ar−H and py−H), 6.34 (m, ^3J(H,H) = 7.1 Hz, 2 H, NCH), 2.66 (q, ^3J(H,H) = 7.6 Hz, 2 H, C*H*$_2$$CH_3$), 1.78 (d, ^3J(H,H) = 7.1 Hz, 12 H, CH_3), 1.23 (t, ^3J(H,H) = 7.6 Hz, 3 H, CH_2C*H*$_3$). $^{13}C\{^1H\}$ NMR (75 MHz, $CDCl_3$): δ 160.5$_8$ (HEP), 156.3,

152.9, 134.2, 124.8, 122.9, 113.3 (Ar – C), 55.2 (NCH), 28.9 (*C*H_2CH_3), 21.3 (CH_3), 14.7 (CH_2*C*H_3). Anal. Calc. for $C_{20}H_{27}Br_2N_3Pd$: C, 41.73; H, 4.73; N, 7.30. Found: C, 41.90; H, 4.75; N, 7.42. MS (ESI): *m/z* = 603 $[M – Br + C_7H_9N]^+$.

***trans*-Dibromido(4-aminopyridine)(1,3-diisopropylbenzimidazolin-2-ylidene)palladium(II) (26).**

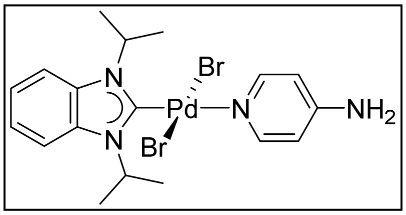

Slow evaporation of a chloroform solution of the crude product afforded the pure product as yellow crystals. Yield: 60 mg (0.10 mmol, >99%). ^{1}H NMR (300 MHz, $CDCl_3$): δ 8.61 (d, 2 H, py–H), 7.57 (dd, 2 H, Ar–H), 7.20 (dd, 2 H, Ar–H), 6.46 (m, 2 H, py–H), 6.34 (m, ^3J(H,H) = 7.1 Hz, 2 H, NCH), 4.44 (br, 2 H, NH_2), 1.78 (d, ^3J(H,H) = 7.1 Hz, 12 H, CH_3). $^{13}C\{^1H\}$ NMR (75 MHz, $CDCl_3$): δ 161.3$_5$ (HEP), 154.3, 153.2, 134.2, 122.8, 113.2, 110.3 (Ar–C), 55.1 (NCH), 21.3 (CH_3). Anal. Calc. for $C_{18}H_{24}Br_2N_4Pd$: C, 38.42; H, 4.30; N, 9.96. Found: C, 38.11; H, 4.15; N, 9.75. MS (ESI): *m/z* = 524 $[M – Br + CH_3CN]^+$.

***trans*-Dibromido(1,3-diisopropylbenzimidazolin-2-ylidene)(4-(dimethylamino)pyridine)-palladium(II) (27).**

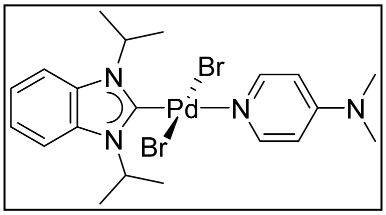

Slow evaporation of a chloroform/dichloromethane solution of the crude product afforded the pure product as yellow crystals. Yield: 60 mg (0.10 mmol, >99%). ^{1}H NMR (300 MHz, $CDCl_3$): δ 8.61 (m, 2H, py–H), 7.57 (dd, 2 H, Ar–H), 7.19 (dd, 2 H, Ar–H), 6.44 (m, 2 H, py–H), 6.36 (m, ^3J(H,H) = 7.1 Hz, 2 H, NCH), 3.02 (s, 6 H, $N(H_3)_2$), 1.78 (d, ^3J(H,H) = 7.1 Hz, 12 H, CH_3). $^{13}C\{^1H\}$ NMR (75 MHz, $CDCl_3$): δ 161.9$_7$ (HEP), 155.1, 152.1, 134.1, 122.7, 113.1, 107.3 (Ar–C), 54.9, (NCH), 39.8 ($N(CH_3)_2$), 21.2 (CH_3). Anal. Calc. for $C_{20}H_{28}Br_2N_4Pd$: C, 40.67; H, 4.78; N, 9.48. Found: C, 38.71; H, 4.33; N, 8.98. MS (ESI): *m/z* = 633 $[M – Br + C_7H_{10}N_2]^+$.

General procedure for preparation of *trans*-[$PdBr_2$(^{i}Pr_2-bimy)(4-py-COO^-M^+)] complexes 28–31, where M = Group I metals.

A mixture of complex **I** (47 mg, 0.05 mmol) and 4-pyridinecarboxylic acid (12 mg, 0.10 mmol) was suspended in dichloromethane (4 mL) and stirred for 2 h at ambient temperature. The appropriate Group I metal hydroxide (0.10 mmol) was added to the resulting yellow solution before stirring for another 1 h at ambient temperature. The resultant mixture was dried *in vacuo* to give the product as a pale yellow solid.

Lithium *trans*-dibromido(1,3-diisopropylbenzimidazolin-2-ylidene)(4-pyridinecarboxylate)-palladate(II) (28).

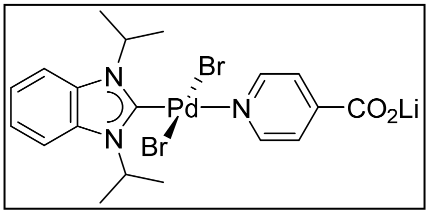

Yield: 60 mg (0.10 mmol, >99%). ^{1}H NMR (300 MHz, $CDCl_3$): δ 9.37 (s, 2 H, py−H), 7.97 (s, 2 H, py−H), 7.62 (s, 2 H, Ar−H), 7.26 (s, 2 H, Ar−H), 6.34 (m, 2 H, NC*H*(CH_3)$_2$), 1.82 (d, 12 H, NCH(C*H*$_3$)$_2$). ^{13}C{^{1}H} NMR (75 MHz, $CDCl_3$): δ 168.5 (COOH), 159.0$_5$ (HEP), 154.4, 139.1, 134.2, 124.8, 123.0, 113.3, (Ar−C), 55.3 (NCH), 21.3 (CH_3).

Sodium *trans*-dibromido(1,3-diisopropylbenzimidazolin-2-ylidene)(4-pyridinecarboxylate)-palladate(II) (29).

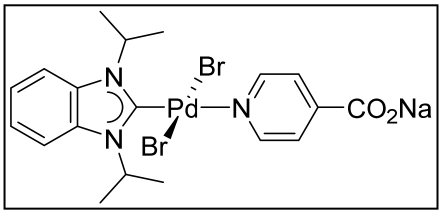

Yield: 60 mg (0.10 mmol, >99%). ^{1}H NMR (300 MHz, $CDCl_3$): δ 8.93 (s, 2 H, py−H), 7.69 (s, 2 H, py−H), 7.54 (s, 2 H, Ar−H), 7.17 (s, 2 H, Ar−H), 6.32 (m, 2 H, NCH), 1.73 (d, 12 H, CH_3). ^{13}C{^{1}H} NMR (75 MHz, $CDCl_3$): δ 171.8 (COOH), 160.8$_4$ (HEP), 153.2, 146.6, 134.2, 124.9, 122.7, 113.3, (Ar−C), 55.3 (NCH), 21.4 (CH_3). MS (ESI): m/z = 590 [M – Na]$^-$.

Potassium *trans*-dibromido(1,3-diisopropylbenzimidazolin-2-ylidene)(4-pyridinecarboxylate)palladate(II) (30).

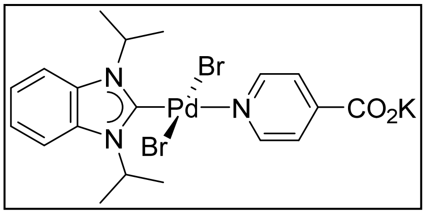

Yield: 60 mg (0.10 mmol, >99%). ^{1}H NMR (300 MHz, $CDCl_3$): δ 8.94 (d, 2 H, py−H), 7.67 (d, 2 H, py−H), 7.52 (dd, 2 H, Ar−H), 7.15 (dd, 2 H, Ar−H), 6.28 (m, ^3J(H,H) = 7.1 Hz, 2 H, NCH), 1.67 (d, ^3J(H,H) = 7.1 Hz, 12 H, CH_3). $^{13}C\{^1H\}$ NMR (75 MHz, $CDCl_3$): δ 160.7$_6$ (HEP), 153.2, 146.9, 134.1, 124.8, 122.8, 113.3, (Ar−C), 55.2 (NCH), 21.3 (CH_3). The COOH signal could not be detected. MS (ESI): *m/z* = 590 $[M - K]^-$.

Rubidium *trans*-dibromido(1,3-diisopropylbenzimidazolin-2-ylidene)(4-pyridinecarboxylate)palladate(II) (31).

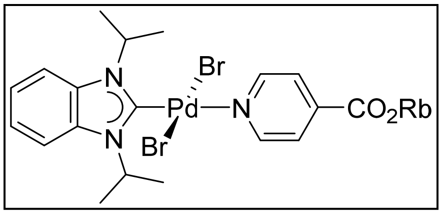

Yield: 60 mg (0.10 mmol, >99%). ^{1}H NMR (300 MHz, $CDCl_3$): δ 9.02 (d, 2 H, py−H), 7.70 (d, 2 H, py−H), 7.55 (dd, 2 H, Ar−H), 7.18 (dd, 2 H, Ar−H), 6.30 (m, ^3J(H,H) = 7.1 Hz, 2H, NCH), 1.72 (d, ^3J(H,H) = 7.1 Hz, 12 H, CH_3). $^{13}C\{^1H\}$ NMR (75 MHz, $CDCl_3$): δ 160.2$_9$ (HEP), 153.4, 134.1, 124.8, 122.9, 113.3, (Ar−C), 55.2 (NCH), 21.3 (CH_3). The COOH signal and one Ar-C signal could not be detected.

***trans*-Dibromido(1,3-diisopropylbenzimidazolin-2-ylidene)(1,5,7-Triazabicyclo[4.4.0]dec-5-ene)-palladium(II)** (**32**).

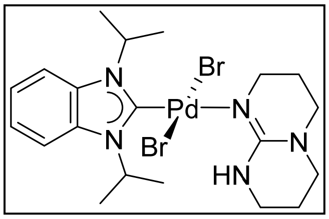

Compound 1,5,7-Triazabicyclo[4.4.0]dec-5-ene (TBD) (14 mg, 0.10 mmol) was added to the suspension of dimer **I** (47 mg, 0.05 mmol) in CH_2Cl_2 (20 mL) and stirred for 1 h. Solvent was removed under reduced pressure. The residue was washed with hexane (3 × 10 mL) and dried in vacuo affording the product as a yellow powder (61 mg, 0.10 mmol, >99%). Single crystals were obtained by slow evaporation of a saturated solution in $CHCl_3$/hexane. ^{1}H NMR (300 MHz, $CDCl_3$): δ 7.50 (dd, 2 H, Ar–H), 7.13 (dd, 2 H, Ar–H), 6.27 (m, ^3J(H,H) = 7.1 Hz, 2 H, NCH), 3.49 (t, ^3J(H,H) = 5.6 Hz, 2 H, TBD–CH_2), 3.26 (t, ^3J(H,H) = 5.8 Hz, 2 H, TBD–CH_2), 3.10–3.05 (m, 4 H, TBD–CH_2), 1.94–1.82 (m, 4 H, TBD–CH_2), 1.74 (d, ^3J(H,H) = 7.1 Hz, 12 H, CH_3). $^{13}C\{^1H\}$ NMR (75 MHz, $CDCl_3$): 165.7$_4$ (HEP), 152.4 (C=N), 134.0, 122.5, 113.0 (Ar–C), 54.8 (NCH), 48.4, 48.0, 46.8, 40.1, 23.9, 22.8 (TBD–C), 21.4 (CH_3). Anal. Calcd for

$C_{20}H_{31}Br_2N_5Pd$: C, 39.53; H, 5.14; N, 11.52. Found: C, 39.86; H, 5.08; N, 11.31. MS (ESI): *m/z* 528 $[M – Br]^+$.

***trans*-Dibromido(1,3-diisopropylbenzimidazolin-2-ylidene)(1,8-diazabicyclo[5.4.0]undec-7-ene)palladium(II) (33).**

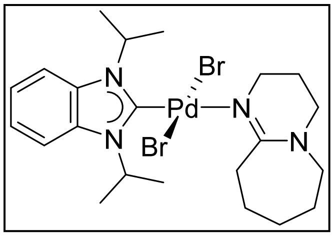

Complex **31** was prepared in analogy to complex **30** from 1,8-diazabicyclo[5.4.0]undec-7-ene (DBU) (15 µL, 0.10 mmol) and dimer **I** (47 mg, 0.05 mmol). Yield: 62 mg (0.10 mmol, >99%). ^{1}H NMR (500 MHz, $CDCl_3$): δ 7.52 (dd, 2 H, Ar–H), 7.16 (dd, 2 H, Ar–H), 6.31 (br–s, 2 H, NCH), 3.56 (t, ^3J(H,H) = 5.5 Hz, 2 H, DBU–CH_2), 3.40–3.38 (m, 2 H, DBU–CH_2), 3.27–3.26 (m, 2 H, DBU–CH_2), 3.23 (t, ^3J(H,H) = 6.0 Hz, 2 H, DBU–CH_2), 1.98 (m, 2 H, DBU–CH_2), 1.89 (m, 2 H, DBU–CH_2), 1.77 (d, ^3J(H,H) = 7.0 Hz, 12 H, CH_3), 1.72 (m, 2 H, DBU–CH_2), 1.60 (br–s, 2 H, DBU–CH_2). $^{13}C\{^1H\}$ NMR (126 MHz, $CDCl_3$): 166.3_2 (HEP), 163.9 (C=N), 134.1, 122.6, 113.0 (Ar–C), 54.9 (NCH), 54.1, 48.5, 47.6, 38.7, 30.0, 28.6, 25.0, 23.1 (DBU–C), 21.4 (CH_3). Anal. Calcd for $C_{22}H_{34}Br_2N_4Pd$: C, 42.57; H, 5.52; N, 9.03. Found: C, 42.26; H, 5.21; N, 8.98. MS (ESI): *m/z* 582 $[M – Br + CH_3CN]^+$.

***trans*-Dibromido(1,3-diisopropylbenzimidazolin-2-ylidene)(triethylamine)palladium(II) (34)**.

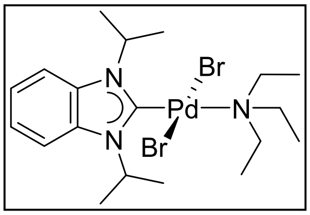

2.5 equiv of triethylamine (TEA) (35 µL, 0.25 mmol) was added to the suspension of **I** (47 mg, 0.05 mmol) in CH_2Cl_2 (20 mL) and stirred overnight. The volatiles were removed in vacuo, affording the product as a yellow powder (57 mg, 0.10 mmol, >99%). ^{1}H NMR (300 MHz, $CDCl_3$): δ 7.52 (dd, 2 H, Ar–H), 7.15 (dd, 2 H, Ar–H), 6.22 (m, ^3J(H,H) = 7.1 Hz, 2 H, NCH), 3.03 (m, ^3J(H,H) = 7.1 Hz, 6 H, NCH_2), 1.74 (d, ^3J(H,H) = 7.1 Hz, 12 H, CH_3), 1.34 (t, ^3J(H,H) = 7.1 Hz, 9 H, CH_2CH_3). $^{13}C\{^1H\}$ NMR (75 MHz, $CDCl_3$): 157.9_6 (HEP), 134.0, 122.6, 113.0 (Ar–C), 54.7 (NCH), 48.9 (NCH_2), 20.9 (CH_3), 10.8 (NCH_2CH_3). Anal. Calcd for $C_{19}H_{33}Br_2N_3Pd$: C, 40.06; H, 5.84; N, 7.38. Found: C, 38.71; H, 5.79; N, 7.06 (the best result obtained). MS (ESI): *m/z* 469 $[M – TEA + H]^+$.

***cis*-Dibromido(1,3-diisopropylbenzimidazolin-2-ylidene)(trimethylphosphite)palladium(II) (*cis*-35).**

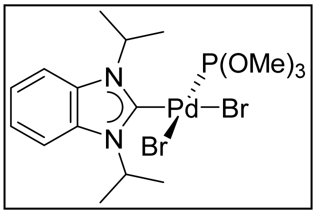

Complex **I** (24 mg, 0.03 mmol) and trimethylphosphite (6 μL, 0.05 mmol) were dissolved in $CDCl_3$ (0.6 mL) for direct NMR analysis. Single crystals were obtained by slow evaporation of a saturated solution in $CHCl_3$/hexane. ^{1}H NMR (300 MHz, $CDCl_3$): δ 7.61 (dd, 2 H, Ar–H), 7.28 (dd, 2 H, Ar–H), 5.79 (m, ^3J(H,H) = 7.0 Hz, 2 H, NCH), 3.83 (d, ^3J(P,H) = 13 Hz, 6 H, OCH_3), 3.77 (d, ^3J(P,H) = 13 Hz, 3 H, OCH_3), 1.74 (d, ^3J(H,H) = 7.0 Hz, 6 H, CH_3), 1.66 (d, ^3J(H,H) = 7.0 Hz, 6 H, CH_3). $^{13}C\{^1H\}$ NMR (75 MHz, $CDCl_3$): 170.7$_9$ (d, ^2J(C,P) = 21.4 Hz, $C_{carbene}$), 133.8, 123.5, 113.7 (Ar–C), 55.2 (br–s, NCH), 55.0 (d, ^2J(P,C) = 5.5 Hz, OCH_3), 73.4 (d, ^2J(P,C) = 4.4 Hz, OCH_3), 21.0, 20.9 (CH_3). $^{31}P\{^1H\}$ NMR (121 MHz, $CDCl_3$): 101.0 (P). Anal. Calcd for $C_{16}H_{27}Br_2N_2O_3PPd$: C, 32.43; H, 4.59; N, 4.73. Found: C, 32.56; H, 5.02; N, 4.45. MS (ESI): *m/z* 545 $[M - Br + MeOH]^+$.

***trans*-Dibromido(1,3-diisopropylbenzimidazolin-2-ylidene)(triisopropylphosphite)palladium(II) (*trans*-36).**

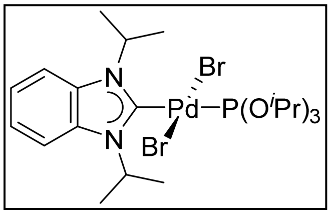

^{13}C-labeled complex **I** (9.4 mg, 0.01 mmol) and triisopropylphosphite (5 μL, 0.02 mmol) were dissolved in $CDCl_3$ (0.6 mL) for direct NMR spectroscopic analysis. ^{1}H NMR (300 MHz, $CDCl_3$): δ 7.56 (dd, 2 H, Ar–H), 7.21 (dd, 2 H, Ar–H), 5.91 (m, ^3J(C,H) = 4.3 Hz, ^3J(H,H) = 7.1 Hz, 2 H, NCH), 5.15 (m, ^3J(P,H) = 8.6 Hz, ^3J(H,H) = 6.1 Hz, 3 H, OCH), 1.77 (d, ^3J(H,H) = 7.1 Hz, 12 H, NCH(C*H*$_3$)$_2$), 1.42 (d, ^3J(H,H) = 6.1 Hz, 18 H, OCH(C*H*$_3$)$_2$). $^{13}C\{^1H\}$ NMR (75 MHz, $CDCl_3$): 175.2$_5$ (d, ^2J(C,P) = 287.1 Hz, HEP). $^{31}P\{^1H\}$ NMR (121 MHz, $CDCl_3$): 113.4 (d, ^2J(C,P) = 287.1 Hz, P).

***cis*-Dibromido(1,3-diisopropylbenzimidazolin-2-ylidene)(triisopropylphosphite)palladium(II) (*cis*-36).**

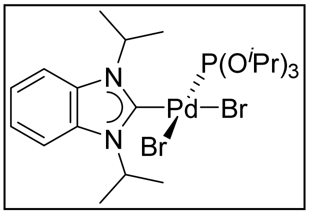

Complex **I** (47 mg, 0.05 mmol) and triisopropylphosphite (25 μL, 0.10 mmol) were dissolved in CH_2Cl_2 (10 mL) and stirred for 0.5 h. The solvent was removed under reduced pressure, and the residue was washed with diethyl ether to give the off-white product (57 mg, 0.08 mmol, 84%). Single crystals were obtained by slow evaporation of a saturated solution in $CHCl_3$/hexane. ^{1}H NMR (500 MHz, $CDCl_3$): δ 7.58 (dd, 2 H, Ar–H), 7.23 (dd, 2 H, Ar–H), 5.82 (m, ^3J(H,H) = 7.0 Hz, 2 H, NCH), 5.15 (br–m, 3 H, OCH), 1.71 (d, ^3J(H,H) = 7.0 Hz, 6 H, NCH(CH_3)$_2$), 1.69 (d, ^3J(H,H) = 7.0 Hz, 6 H, NCH(CH_3)$_2$), 1.19 (d, ^3J(H,H) = 6.3 Hz, 18 H, OCH(CH_3)$_2$). ^{13}C{^{1}H} NMR (126 MHz, $CDCl_3$): 171.9$_8$ (d, ^2J(C,P) = 22.9 Hz, $C_{carbene}$), 133.8, 123.3, 113.4 (Ar–C), 73.4 (d, ^2J(P,C) = 4.6 Hz, OCH), 55.1 (d, ^4J(P,C) = 1.8 Hz, NCH), 24.5 (d, ^3J(P,C) = 4.6 Hz, OCH(CH_3)$_2$), 21.3, 21.1 (NCH(CH_3)$_2$). ^{31}P{^{1}H} NMR (202 MHz, $CDCl_3$): 90.4 (P). Anal. Calcd for $C_{22}H_{39}Br_2N_2O_3PPd$: C, 39.04; H, 5.81; N, 4.14. Found: C, 39.06; H, 5.40; N, 4.08. MS (ESI): *m/z* 628 [M – Br + MeOH]$^+$.

***trans*-Dibromido(1,3-diisopropylbenzimidazolin-2-ylidene)(triphenylphosphite)palladium(II) (*trans*-37).**

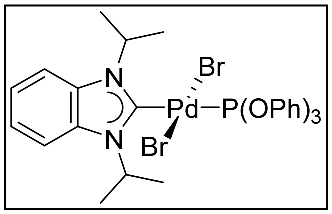

Complex **I** (24 mg, 0.03 mmol) and triphenylphosphite (13 μL, 0.05 mmol) were dissolved in $CDCl_3$ (0.6 mL) for direct NMR analysis. ^{1}H NMR (300 MHz, $CDCl_3$): δ 7.50–7.30 (m, 11 H, Ar–H), 7.25–7.14 (m, 8 H, Ar–H), 5.21 (m, ^3J(H,H) = 7.2 Hz, 2 H, NCH), 1.51 (d, ^3J(H,H) = 7.2 Hz, 12 H, CH_3). ^{13}C{^{1}H} NMR (75 MHz, $CDCl_3$): 171.6$_5$ (d, ^2J(C,P) = 289.8 Hz, HEP), 151.8 (d, ^2J(C,P) = 5.5 Hz, Ar–C), 134.0 (d, ^4J(C,P) = 9.3 Hz, Ar–C), 130.2, 125.7, 123.0 (Ar–C), 122.2 (d, ^3J(C,P) = 6.0 Hz, Ar–C), 113.5 (Ar–C), 54.6 (NCH), 21.3 (CH_3). ^{31}P{^{1}H} NMR (202 MHz, $CDCl_3$): 102.3 (P).

***cis*-Dibromido(1,3-diisopropylbenzimidazolin-2-ylidene)(triphenylphosphite)palladium(II) (*cis*-37).**

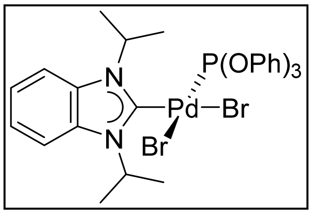

Complex **I** (47 mg, 0.05 mmol) and triphenylphosphite (26 μL, 0.10 mmol) were suspended in MeOH (10 mL) and stirred overnight. The off-white solid product was isolated by filtration and further dried in vacuo. Yield: 72 mg, 0.09 mmol, 93%. Single crystals were obtained by slow evaporation of a saturated solution in $CHCl_3$/hexane. ^1H NMR (500 MHz, $CDCl_3$): *δ* 7.55 (dd, 2 H, Ar–H), 7.34–7.29 (m, 11 H, Ar–H), 7.27–7.22 (m, 6 H, Ar–H), 5.32 (m, ^3J(H,H) = 7.0 Hz, 2 H, NCH), 1.57 (d, ^3J(H,H) = 7.0 Hz, 6 H, CH_3), 1.14 (d, ^3J(H,H) = 7.0 Hz, 6 H, CH_3). $^{13}C\{^1H\}$ NMR (126 MHz, $CDCl_3$): 169.2$_7$ (d, ^2J(C,P) = 22.9 Hz, $C_{carbene}$), 151.1 (d, ^2J(C,P) = 8.3 Hz, Ar–C), 133.9, 130.8, 126.7, 123.6 (Ar–C), 122.0 (d, ^3J(C,P) = 4.6 Hz, Ar–C), 113.8 (Ar–C), 55.6 (NCH), 20.9, 20.8 (CH_3). $^{31}P\{^1H\}$ NMR (202 MHz, $CDCl_3$): 86.3 (P). Anal. Calcd for $C_{31}H_{33}Br_2N_2O_3PPd$: C, 47.81; H, 4.27; N, 3.60. Found: C, 48.13; H, 4.46; N, 3.90. MS (ESI): *m/z* 740 [M – Br + $CH_3CN]^+$.

***trans*-Dibromido(1,3-diisopropylbenzimidazolin-2-ylidene)(triphenylphosphite)palladium(II) (*trans*-38).**

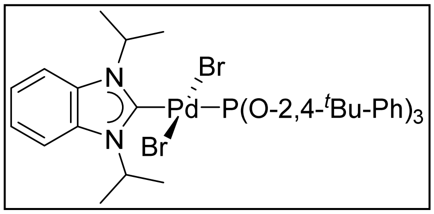

This complex was synthesized by reacting complex **I** (47 mg, 0.05 mmol) and tris(2,4-di-*tert*-butyltriphenyl)phosphite (65 mg, 0.10 mmol) for 30 min in CH_2Cl_2 before dried in vacuum. Yield: 120 mg, 0.01 mmol, >99%. ^1H NMR (500 MHz, $CDCl_3$): *δ* 8.09 (dd, 3 H, Ar–H), 7.53 (dd, 2 H, Ar–H), 7.44 (d, 3 H, Ar–H), 7.22–7.20 (m, 5 H, Ar–H), 5.66 (m, ^3J(H,H) = 7.0 Hz, 2 H, NCH), 1.62 (s, 27 H, $C(CH_3)_3$), 1.59 (d, ^3J(H,H) = 7.0 Hz, 12 H, CH_3), 1.35 (s, 27 H, $C(CH_3)_3$). $^{13}C\{^1H\}$ NMR (126 MHz, $CDCl_3$): 171.4$_8$ (d, ^2J(C,P) = 289.6 Hz, HEP), 149.3 (d, ^2J(C,P) = 3.7 Hz, Ar–C), 146.8 (Ar–C), 139.5 (d, ^3J(C,P) = 5.5 Hz, Ar–C), 134.1 (d, ^4J(C,P) = 9.2 Hz, Ar–C), 125.5, 123.4, 122.9 (Ar–C), 120.1 (d, ^3J(C,P) = 11.9 Hz, Ar–C), 113.4 (Ar–C), 54.7 (NCH), 36.0, 35.2 (*C*$(CH_3)_3$), 32.3, 31.5 (C(*C*$H_3)_3$), 21.2 (CH_3). $^{31}P\{^1H\}$ NMR (202 MHz, $CDCl_3$): 103.6 (P). Anal. Calcd for $C_{55}H_{81}Br_2N_2O_3PPd$: C, 59.22; H, 7.32; N, 2.51. Found: C, 59.48; H, 7.12; N, 2.16. MS (ESI): *m/z* 1075 [M – Br + $CH_3CN]^+$.

***trans*-Dibromido(1,3-diisopropylbenzimidazolin-2-ylidene)(triphenylarsine)palladium(II) (*trans*-39).**

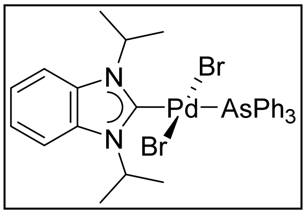

Complex **I** (24 mg, 0.03 mmol) and triphenylarsine (15 mg, 0.05 mmol) were dissolved in $CDCl_3$ (0.6 mL) for direct NMR measurement. ^{1}H NMR (500 MHz, $CDCl_3$): *δ* 7.69−7.68 (m, 6 H, Ar–H), 7.59 (dd, 2 H, Ar–H), 7.44−7.39 (m, 9 H, Ar–H), 7.22 (dd, 2 H, Ar–H), 6.15 (m, ^3J(H,H) = 7.1 Hz, 2 H, NCH), 1.79 (d, ^3J(H,H) = 7.1 Hz, 12 H, CH_3). $^{13}C\{^1H\}$ NMR (126 MHz, $CDCl_3$): 169.1_7 (HEP), 135.2, 134.3, 133.5, 130.5, 129.2, 123.0, 113.5 (Ar–C), 55.1 (NCH), 21.5 (CH_3).

***cis*-Dibromido(1,3-diisopropylbenzimidazolin-2-ylidene)(triphenylarsine)palladium(II) (*cis*-39).**

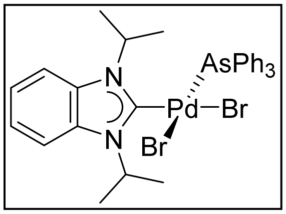

Complex *trans*-**36** was left to isomerize in $CDCl_3$ for 6 d to this complex as major product. Single crystals were obtained by slow evaporation of a saturated solution in $CHCl_3$/hexane. ^{1}H NMR (500 MHz, $CDCl_3$): *δ* 7.47 (dd, 2 H, Ar–H), 7.38 (t, ^3J(H,H) = 7.3 Hz, 4 H, Ar–H), 7.33−7.27 (m, 5 H, Ar–H), 7.24−7.20 (m, 8 H, Ar–H), 5.90 (m, ^3J(H,H) = 7.0 Hz, 2 H, NCH), 1.66 (d, ^3J(H,H) = 7.0 Hz, 6 H, CH_3), 0.89 (d, ^3J(H,H) = 7.0 Hz, 6 H, CH_3). $^{13}C\{^1H\}$ NMR (126 MHz, $CDCl_3$): 169.3_4 ($C_{carbene}$), 135.2, 134.1, 132.7, 131.3, 129.7, 123.4, 113.4 (Ar–C), 55.4 (NCH), 21.6, 19.9 (CH_3). Anal. Calcd for $C_{31}H_{33}AsBr_2N_2Pd$: C, 48.06; H, 4.29; N, 3.62. Found: C, 48.42; H, 4.16; N, 3.80. MS (ESI): *m/z* 736 $[M - Br + CH_3CN]^+$.

***cis*-Dibromido(1,3-diisopropylbenzimidazolin-2-ylidene)(triphenylstibine)palladium(II) (*cis*-40).**

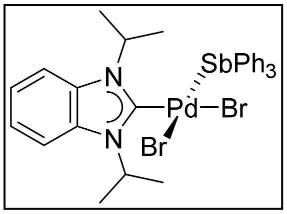

Complex **I** (24 mg, 0.03 mmol) and triphenylstibine (18 mg, 0.05 mmol) were dissolved in $CDCl_3$ (0.6 mL) for direct NMR measurement. Single crystals were obtained by slow evaporation of a saturated solution in $CHCl_3$/hexane. ^{1}H NMR (500 MHz, $CDCl_3$): *δ* 7.49 (dd, 2 H, Ar–H), 7.36 (ps–t, 3 H, Ar–H), 7.31 (ps–d, 6 H, Ar–H), 7.25 (dd, 2 H, Ar–H), 7.20 (ps–t, 6 H, Ar–H), 5.88 (m, ^3J(H,H) = 7.0 Hz, 2 H, NCH), 1.66 (d, ^3J(H,H) = 7.0 Hz, 6 H, CH_3), 1.04 (d, ^3J(H,H) = 7.0 Hz, 6

H, CH_3). $^{13}C\{^1H\}$ NMR (126 MHz, $CDCl_3$): 167.4$_6$ ($C_{carbene}$), 136.1, 134.4, 131.4, 130.1, 129.9, 123.5, 113.3 (Ar–C), 55.3 (NCH), 21.4, 20.2 (CH_3). Anal. Calcd for $C_{31}H_{33}Br_2N_2PdSb$: C, 45.32; H, 4.05; N, 3.41. Found: C, 45.25; H, 3.99; N, 3.44. MS (ESI): *m/z* 782 $[M - Br + CH_3CN]^+$.

***trans*-Dibromido(1,3-diisopropylbenzimidazolin-2-ylidene)(dimethylsulfide)palladium(II) (41).**

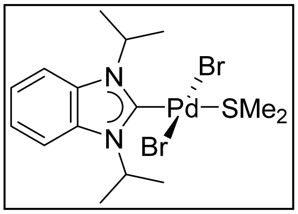

Complex **21** was prepared in analogy to the pyridine complexes as a yellow solid from the reaction of dimethylsulfide (8 μL, 0.10 mmol) and complex **I** (47 mg, 0.05 mmol) in CH_2Cl_2 (10 mL). Yield: 53 mg, 0.10 mmol, >99%. ^1H NMR (500 MHz, $CDCl_3$): δ 7.58 (dd, 2 H, Ar–H), 7.12 (dd, 2 H, Ar–H), 6.04 (m, ^3J(H,H) = 7.0 Hz, 2 H, NCH), 2.47 (s, 6 H, SCH_3), 1.74 (d, ^3J(H,H) = 7.0 Hz, 12 H, CH_3). $^{13}C\{^1H\}$ NMR (126 MHz, $CDCl_3$): 163.4$_6$ (HEP), 134.1, 123.1, 113.4 (Ar–C), 55.2 (NCH), 21.8 (SCH_3), 21.3 (CH_3). Anal. Calcd for $C_{15}H_{24}Br_2N_2PdS$: C, 33.95; H, 4.56; N, 5.28. Found: C, 33.86; H, 4.08; N, 5.66 (the best result obtained). MS (ESI): *m/z* 572 $[M + CH_3CN + H]^+$.

***trans*-Dibromido(1,3-diisopropylbenzimidazolin-2-ylidene)(tetrahydrothiophene)palladium(II) (42).**

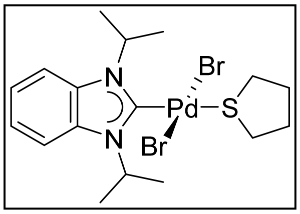

Complex **22** was also prepared in analogy to the pyridine complexes as a yellow solid from the reaction of tetrahydrothiophene (THT) (9 μL, 0.10 mmol) and complex **I** (47 mg, 0.05 mmol) in CH_2Cl_2 (10 mL). Yield: 56 mg, 0.10 mmol, >99%. Single crystals were obtained by slow evaporation of a saturated solution in $CHCl_3$/hexane. ^1H NMR (500 MHz, $CDCl_3$): δ 7.56 (dd, 2 H, Ar–H), 7.20 (dd, 2 H, Ar–H), 6.04 (m, ^3J(H,H) = 6.9 Hz, 2 H, NCH), 3.30 (br–s, 4 H, SCH_2), 2.07 (br–s, 4 H, CH_2), 1.74 (d, ^3J(H,H) = 6.9 Hz, 12 H, CH_3). $^{13}C\{^1H\}$ NMR (126 MHz, $CDCl_3$): 163.6$_3$ (HEP), 134.1, 123.0, 113.3 (Ar–C), 55.1 (NCH), 36.4 (SCH_2), 30.8 (CH_2), 21.3 (CH_3). Anal. Calcd for $C_{17}H_{26}Br_2N_2PdS$: C, 36.68; H, 4.71; N, 5.03. Found: C, 36.66; H, 4.64; N, 5.14. MS (ESI): *m/z* 518 $[M - Br + CH_3CN]^+$.

***trans*-Dibromido(1,3-diisopropylbenzimidazolin-2-ylidene)(pyridine-N-oxide)palladium(II) (43).**

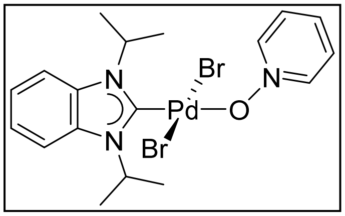

Complex **I** (47 mg, 0.10 mmol) and pyridine N-oxide (24 mg, 0.25 mmol) were heated in $CHCl_3$ (20 mL) at 70 °C overnight. The resulting solution was concentrated to 3 mL before diethylether (20 mL) was added. The orange precipitate was collected and dried as the product (101 mg, 0.18 mmol, 90%). Single crystals were obtained by slow evaporation of a saturated solution in $CHCl_3$/hexane. ^1H NMR (300 MHz, $CDCl_3$): δ 8.59 (br–s, 2 H, Ar–H), 7.51 (dd, 2 H, Ar–H), 7.45 (br–s, 3 H, Ar–H), 7.15 (dd, 2 H, Ar–H), 6.38 (m, ^3J(H,H) = 7.1 Hz, 2 H, NCH), 1.72 (d, ^3J(H,H) = 7.1 Hz, 12 H, CH_3). $^{13}C\{^1H\}$ NMR (75 MHz, $CDCl_3$): 155.7$_4$ (HEP), 142.4, 133.9, 126.5 (2 ×), 122.8, 113.0 (Ar–C), 55.2 (NCH), 21.1 (CH_3). Anal. Calcd for $C_{18}H_{23}Br_2N_3OPd$: C, 38.36; H, 4.11; N, 7.46. Found: C, 38.17; H, 4.16; N, 7.24. MS (ESI): *m/z* 525 $[M - Br + CH_3CN]^+$.

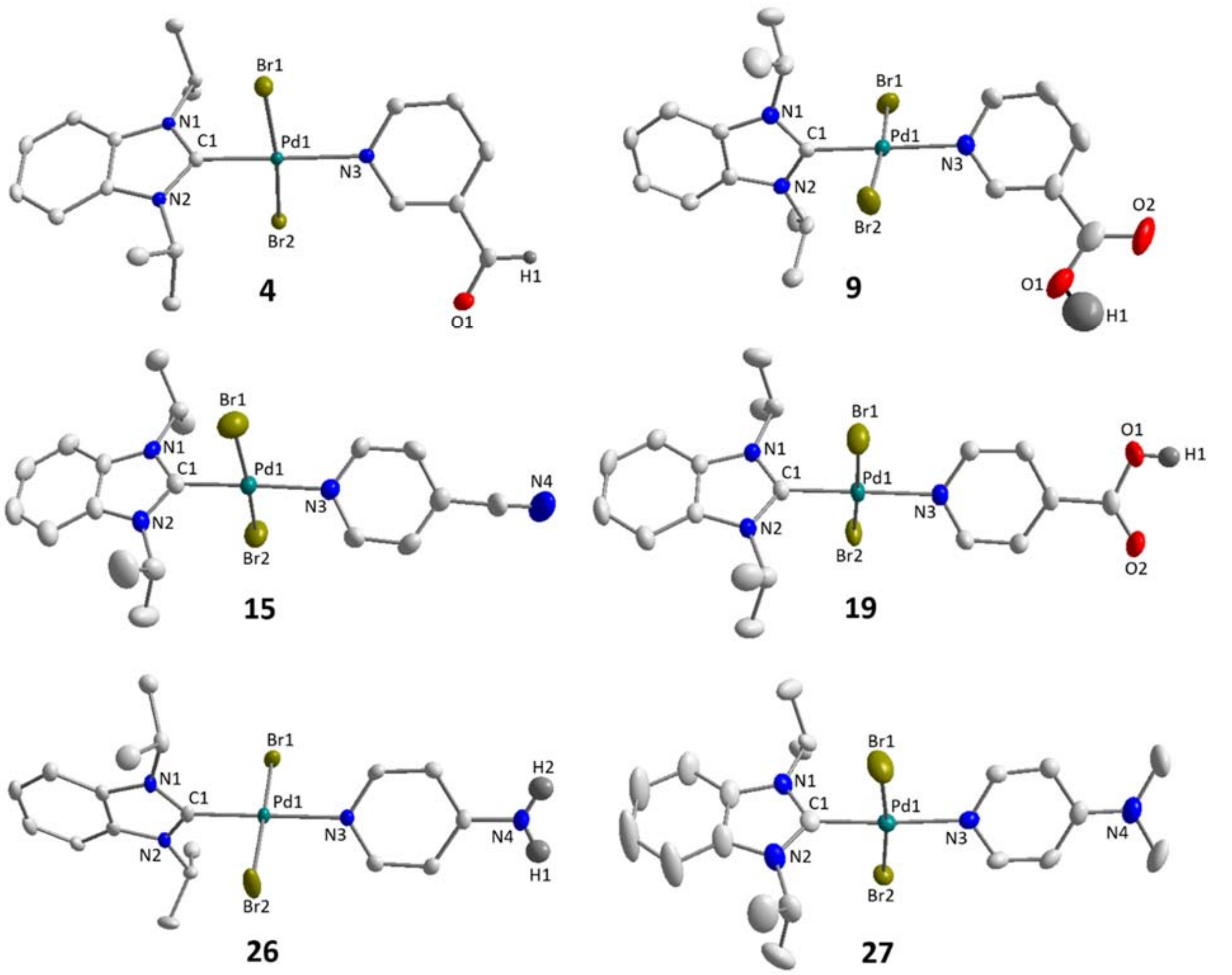

Figure S1. Molecular structures of **4**, **9**, **15**·CH_2Cl_2, **19**·$2CH_2Cl_2$, **26**·$0.5CHCl_3$, **27**·$0.5CHCl_3$·$0.5CH_2Cl_2$ showing 50% probability ellipsoids; hydrogen atoms and solvent molecules are omitted for clarity. Selected bond length [Å] and bond angles [°]: **4**, Pd1–C1 1.953(3), Pd1–Br1 2.4230(4), Pd1–Br2 2.4487(4), Pd1–N3 2.119(2); C1–Pd1–Br1 87.22(8), C1–Pd1–Br2 87.22(8), Br1–Pd1–N3 92.95(6), Br2–Pd1–N3 92.61(6); $PdCBr_2N$/NHC dihedral angle θ 82.24°. **9**, Pd1–C1 1.952(4), Pd1–Br1 2.4183(7), Pd1–Br2 2.4413(7), Pd1–N3 2.110(4); C1–Pd1–Br1 86.9(1), C1–Pd1–Br2 89.1(1), Br1–Pd1–N3 91.2(1), Br2–Pd1–N3 92.8(1); θ 86.51°. **15**, Pd1–C1 1.947(7), Pd1–Br1 2.421(1), Pd1–Br2 2.417(1), Pd1–N3 2.095(6); C1–Pd1–Br1 87.7(2), C1–Pd1–Br2 88.4(2), Br1–Pd1–N3 90.3(2), Br2–Pd1–N3 93.7(2); θ 86.84°. **19**, Pd1–C1 1.961(3), Pd1–Br1 2.4198(4), Pd1–Br2 2.4410(4), Pd1–N3 2.103(3); C1–Pd1–Br1 90.71(9), C1–Pd1–Br2 86.89(9), Br1–Pd1–N3 90.14(7), Br2–Pd1–N3 92.34(7); θ 89.71°. **26**, Pd1–C1 1.973(6), Pd1–Br1 2.407(1), Pd1–Br2 2.4317(9), Pd1–N3 2.088(5); C1–Pd1–Br1 89.7(2), C1–Pd1–Br2 88.8(2), Br1–Pd1–N3 90.9(2), Br2–Pd1–N3 90.6(2); θ 83.09°. **27**, Pd1–C1 1.955(6), Pd1–Br1 2.4311(8), Pd1–Br2 2.4166(8), Pd1–N3 2.095(5); C1–Pd1–Br1 88.2(2), C1–Pd1–Br2 85.9(2), Br1–Pd1–N3 92.6(1), Br2–Pd1–N3 93.5(1); θ 85.79°.

Table S1. Selected Crystallographic data.

Compound	**4**	**9**	**15**·CH_2Cl_2	**19**·$2CH_2Cl_2$	**26**·$0.5CHCl_3$
Lattice	Triclinic	Monoclinic	Monoclinic	Triclinic	Monoclinic
Formula	$C_{19}H_{23}Br_2N_3OPd$	$C_{19}H_{23}Br_2N_3O_2Pd$	$C_{19}H_{22}Br_2N_4Pd\cdot CH_2Cl_2$	$C_{19}H_{23}Br_2N_4O_2Pd\cdot 2CH_2Cl_2$	$C_{18}H_{24}Br_2N_4Pd\cdot 0.5CHCl_3$
Formula weight	575.62	591.62	657.55	761.48	622.32
Space group	*P*-1	$P2_1$/n	*C*2/c	*P*-1	$P2_1$/c
a/Å	9.1210(10)	15.3284(7)	41.587(4)	9.9511(5)	16.159(2)
b/Å	9.4755(10)	9.4384(5)	14.0574(14)	10.8305(5)	9.6149(14)
c/Å	12.7092(13)	16.6434(8)	8.7497(9)	15.1461(7)	30.464(4)
α/°	70.348(2)	90	90	97.3690(10)	90
β/°	85.363(2)	116.238(2)	98.940(2)	100.9540(10)	105.365(3)
γ/°	80.042(2)	90	90	113.1680(10)	90
V/Å^3	1018.54(19)	2159.80(18)	5053.0(9)	1435.64(12)	4563.9(11)
Z	2	4	8	2	8
Temperature (K)	100(2)	223(2)	223(2)	223(2)	100(2)
Radiation (λ,Å)	0.71073	0.71073	0.71073	0.71073	0.71073
ρ (calcd.), g cm^{-3}	1.877	1.819	1.729	1.762	1.811
θ max, deg.	27.49	27.50	25.00	27.49	27.50
No. of data	4647	4945	4466	6559	10473
No. of parameters	239	249	285	303	507
R_1 [I>2sigma(I)]	0.0262	0.0490	0.0625	0.0368	0.0473
wR_2	0.0696	0.0997	0.1530	0.0882	0.1104
GOF	1.076	1.024	1.021	1.038	1.078

Table S1. Continued

Compound	**27**·0.5CHCl$_3$·0.5CH$_2$Cl$_2$	**32**	***cis*-35**·CH$_2$Cl$_2$	***cis*-36**·CHCl$_3$	***cis*-37**·2CHCl$_3$
Lattice	Orthorhombic	Orthorhombic	Triclinic	Monoclinic	Monoclinic
Formula	$C_{20}H_{28}Br_2N_4Pd$·0.5CHCl$_3$·0.5CH$_2$Cl$_2$	$C_{20}H_{31}Br_2N_5Pd$	$C_{16}H_{27}Br_2N_2O_3PPd$·CH$_2Cl_2$	$C_{22}H_{39}Br_2N_2O_3PPd$·CHCl$_3$	$C_{31}H_{33}Br_2N_2O_3PPd$·2CHCl$_3$
Formula weight	692.83	607.72	677.51	796.11	1017.52
Space group	*Pccn*	*Pbca*	*P*-1	*P2$_1$/c*	*P2$_1$/n*
a/Å	20.261(2)	9.5113(4)	10.7930(9)	15.6417(8)	9.5178(8)
b/Å	13.9454(17)	17.3866(7)	11.3295(10)	11.2303(5)	29.221(2)
c/Å	17.297(2)	28.6770(12)	12.8900(10)	19.3568(9)	14.4122(12)
α/°	90	90	65.134(2)	90	90
β/°	90	90	67.128(2)	97.7740(10)	93.881(2).
γ/°	90	90	82.651(2)	90	90
V/Å^3	4887.2(10)	4742.3(3)	1316.43(19)	3369.0(3)	3999.2(6)
Z	8	8	2	4	4
Temperature (K)	100(2)	223(2)	223(2)	223(2)	100(2)
Radiation (λ,Å)	0.71073	0.71073	0.71073	0.71073	0.71073
ρ (calcd.), g cm^{-3}	1.883	1.702	1.709	1.570	1.690
θ max, deg.	25.00	27.49	25.00	27.50	27.50
No. of data	4308	5448	4638	7718	9180
No. of parameters	313	284	325	326	468
R_1 [I>2sigma(I)]	0.0475	0.0497	0.0551	0.0391	0.0453
wR_2	0.1541	0.1062	0.1401	0.0933	0.1059
GOF	1.140	1.062	1.018	1.038	1.042

Table S1. Continued

Compound	***cis*-39**	***cis*-40**	**42**	**43**·$CHCl_3$
Lattice	Monoclinic	Monoclinic	Monoclinic	Monoclinic
Formula	$C_{31}H_{33}AsBr_2N_2Pd$	$C_{31}H_{33}Br_2N_2PdSb$	$C_{17}H_{26}Br_2N_2PdS$	$C_{18}H_{23}Br_2N_3OPd \cdot CHCl_3$
Formula weight	774.73	821.56	556.68	682.98
Space group	$P2_1$/n	$P2_1$/c	$P2_1$/c	$P2_1$/n
a/Å	10.5639(5)	10.3243(4)	12.7531(7)	10.2308(6)
b/Å	17.5659(11)	14.7594(5)	9.9648(6)	17.7001(9)
c/Å	16.5458(10)	20.3602(6)	16.0437(9)	13.5874(7)
α/°	90	90	90	90
β/°	93.981(2)	97.7730(12)	105.1420(10)	93.122(2)
γ/°	90	90	90	90
V/Å^3	3062.9(3)	3073.99(18)	1968.08(19)	2456.8(2)
Z	4	4	4	4
Temperature (K)	100(2)	100(2)	100(2)	100(2)
Radiation (λ,Å)	0.71073	0.71073	0.71073	0.71073
ρ (calcd.), g cm^{-3}	1.680	1.775	1.879	1.846
θ max, deg.	27.500	27.498	27.49	27.500
No. of data	7045	7067	4507	5637
No. of parameters	338	338	227	266
R_1 [I>2sigma(I)]	0.0228	0.0299	0.0292	0.0240
wR_2	0.0468	0.0694	0.0682	0.0545
GOF	1.039	1.056	1.036	1.034

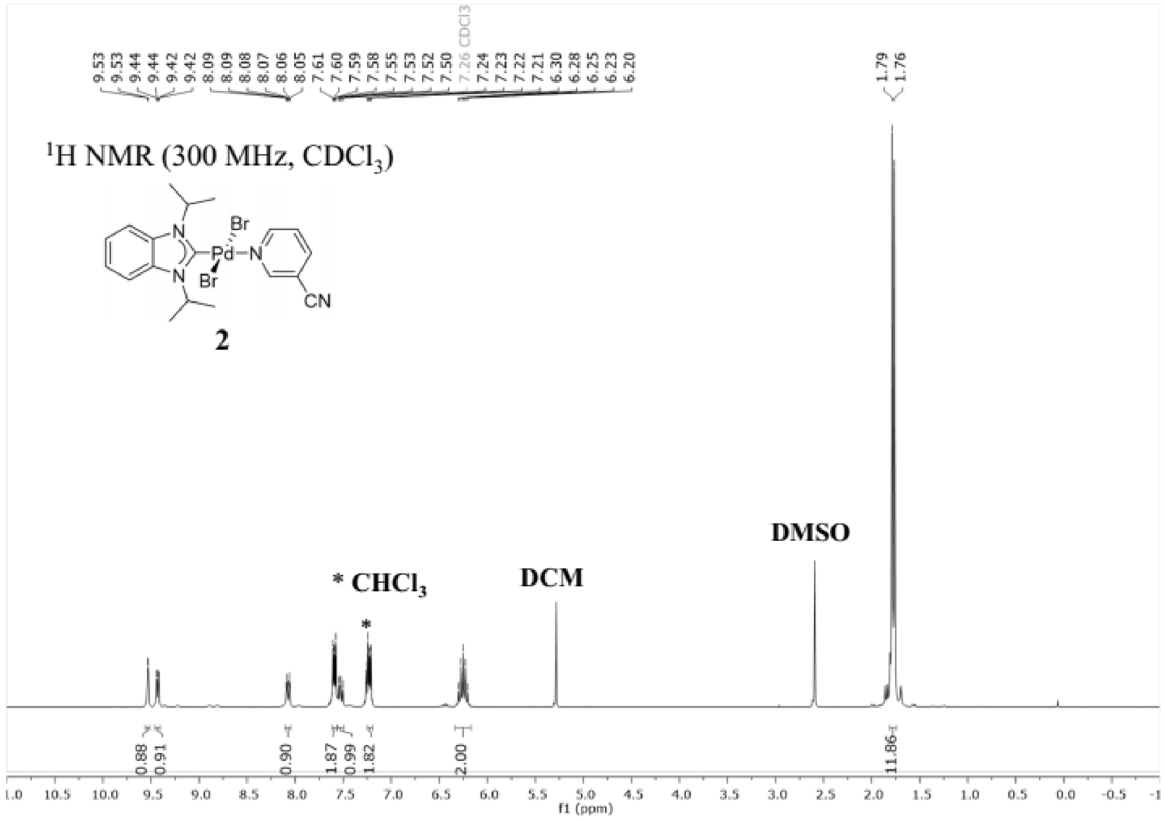
1H NMR (300 MHz, CDCl3)
2
DMSO
* CHCl3
DCM
f1 (ppm)

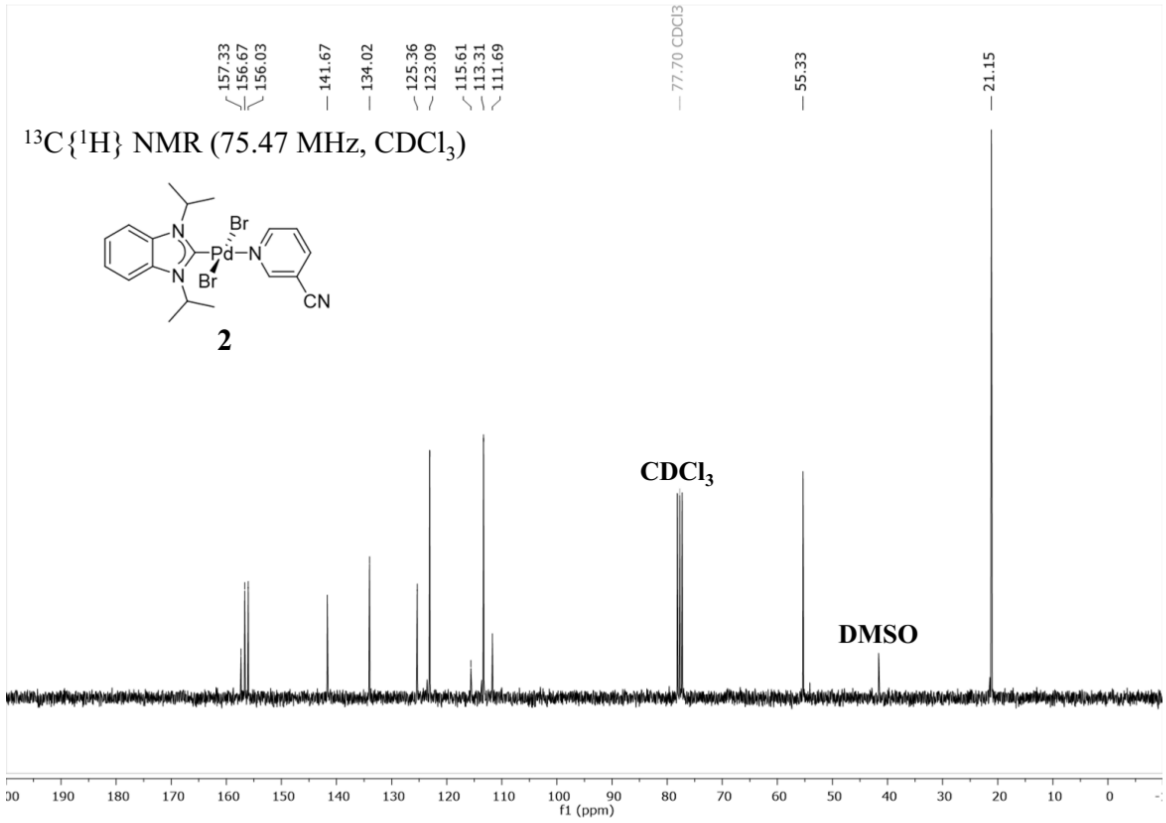
13C{1H} NMR (75.47 MHz, CDCl3)
2
CDCl3
DMSO
f1 (ppm)

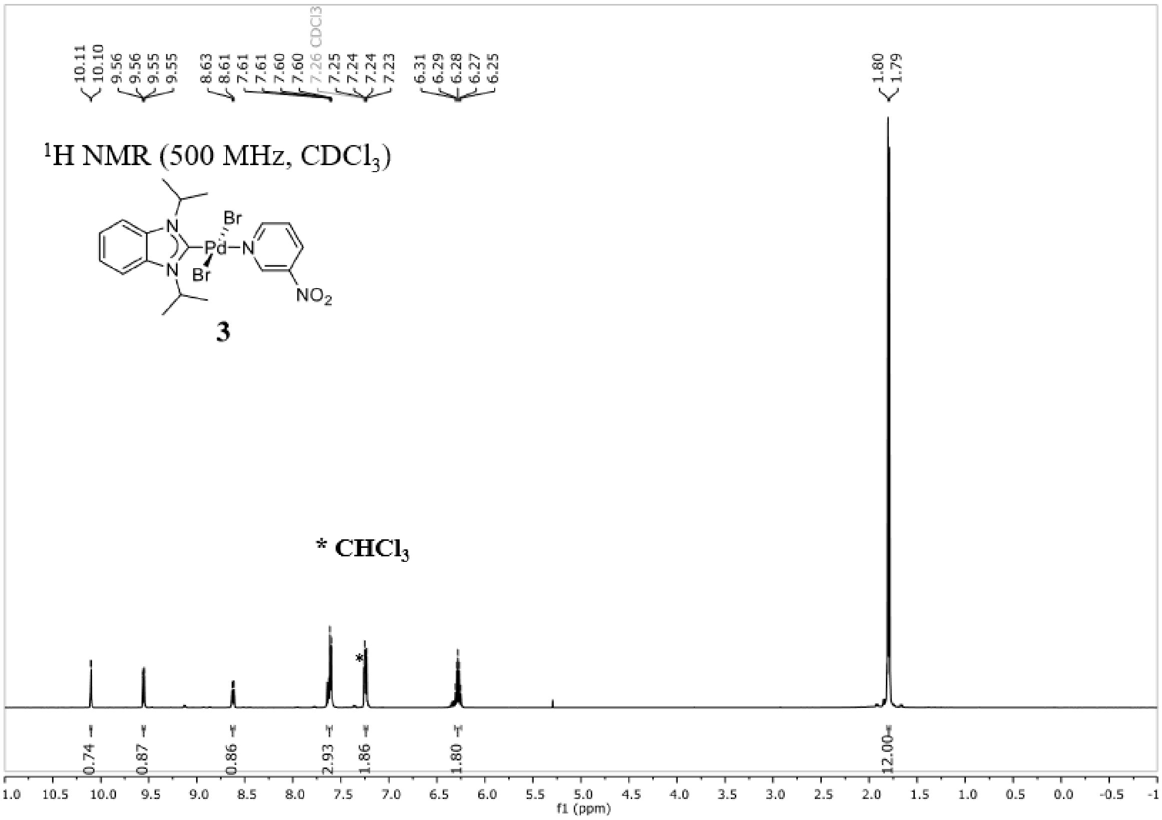
10.11
10.10
9.56
9.56
9.55
9.55
8.63
8.61
7.61
7.61
7.60
7.60
7.26 CDCl3
7.25
7.24
7.24
7.23
6.31
6.29
6.28
6.27
6.25
1.80
1.79
1H NMR (500 MHz, CDCl3)
Br
N
Pd
N
Br
N
NO2
3
* CHCl3
*
0.74
0.87
0.86
2.93
1.86
1.80
12.00
f1 (ppm)

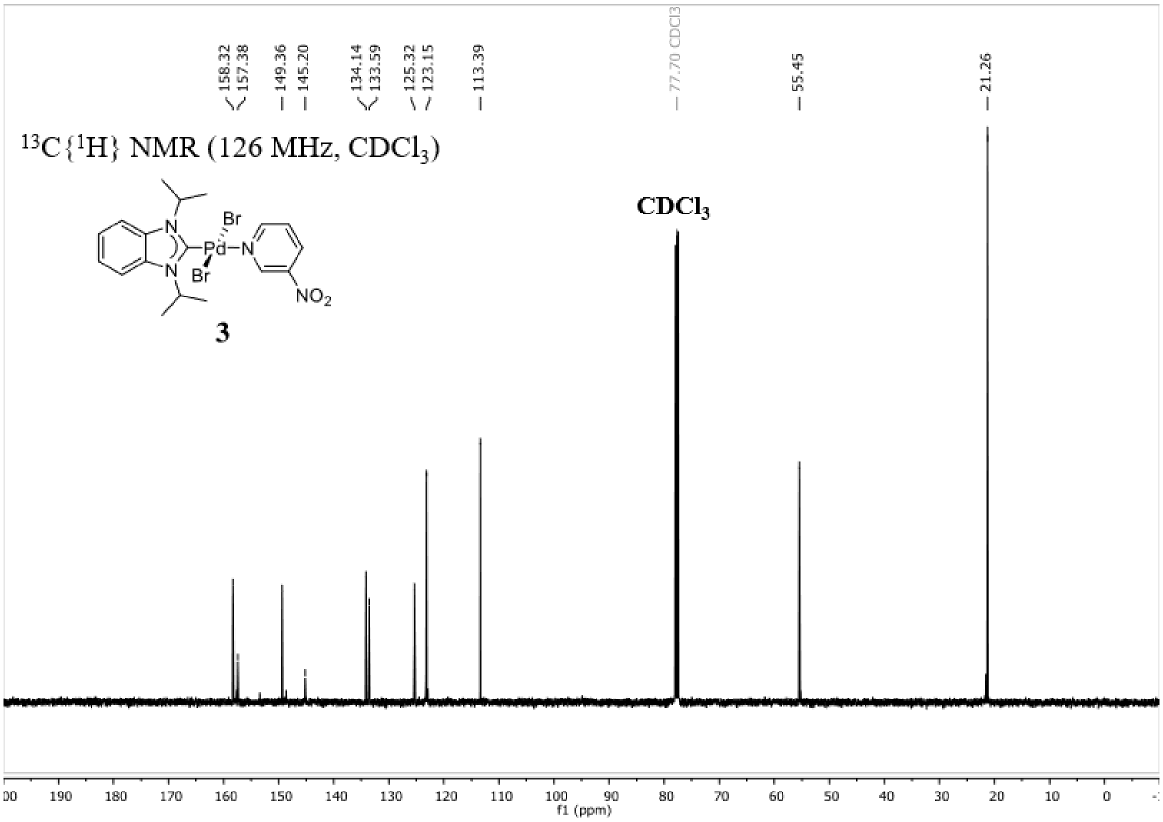
158.32
157.38
149.36
145.20
134.14
133.59
125.32
123.15
113.39
77.70 CDCl3
55.45
21.26
13C{1H} NMR (126 MHz, CDCl3)
Br
N
Pd
N
Br
N
NO2
3
CDCl3
f1 (ppm)

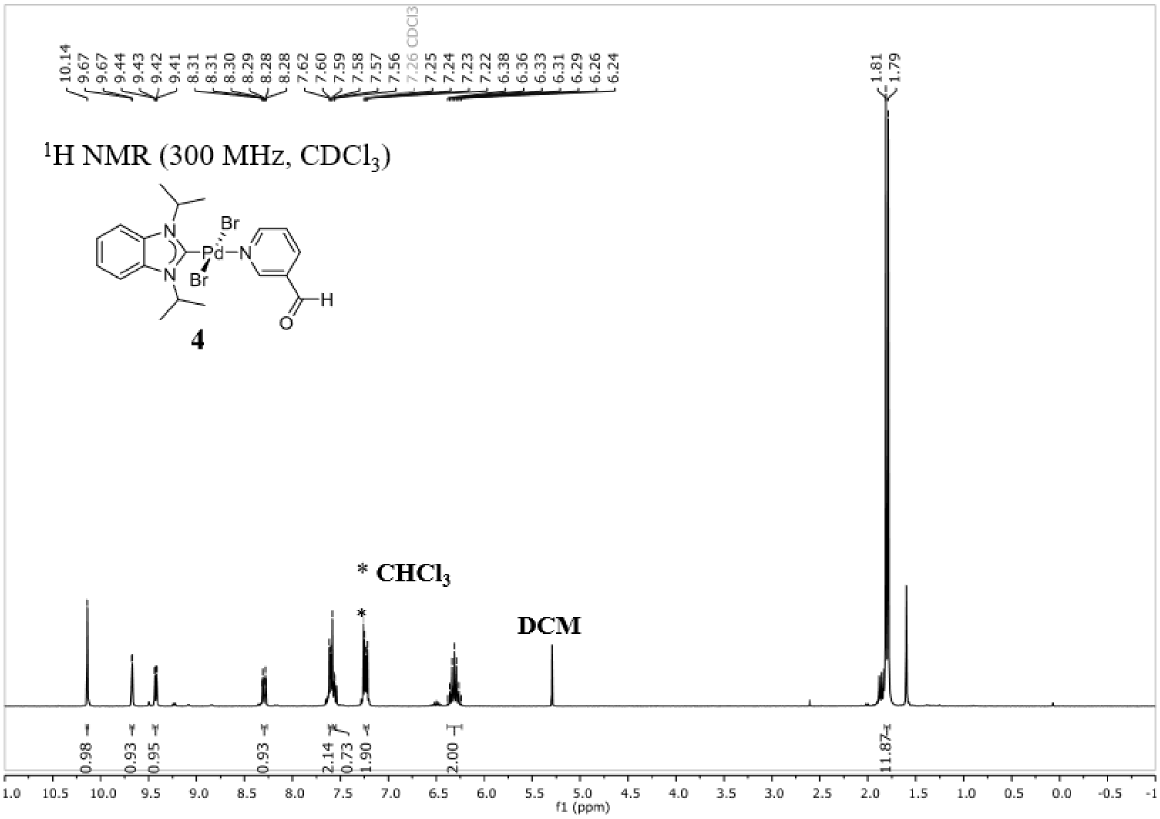

10.14 9.67 9.67 9.44 9.43 9.42 9.41 8.31 8.31 8.30 8.29 8.28 8.28 7.62 7.60 7.59 7.58 7.57 7.56 7.26 CDCl3 7.25 7.24 7.23 7.22 6.38 6.36 6.33 6.31 6.29 6.26 6.24
1.81 1.79
1H NMR (300 MHz, CDCl3)
Br
Pd
N
Br
H
O
4
* CHCl3
*
DCM
0.98 0.93 0.95 0.93 2.14 0.73 1.90 2.00 11.87
f1 (ppm)

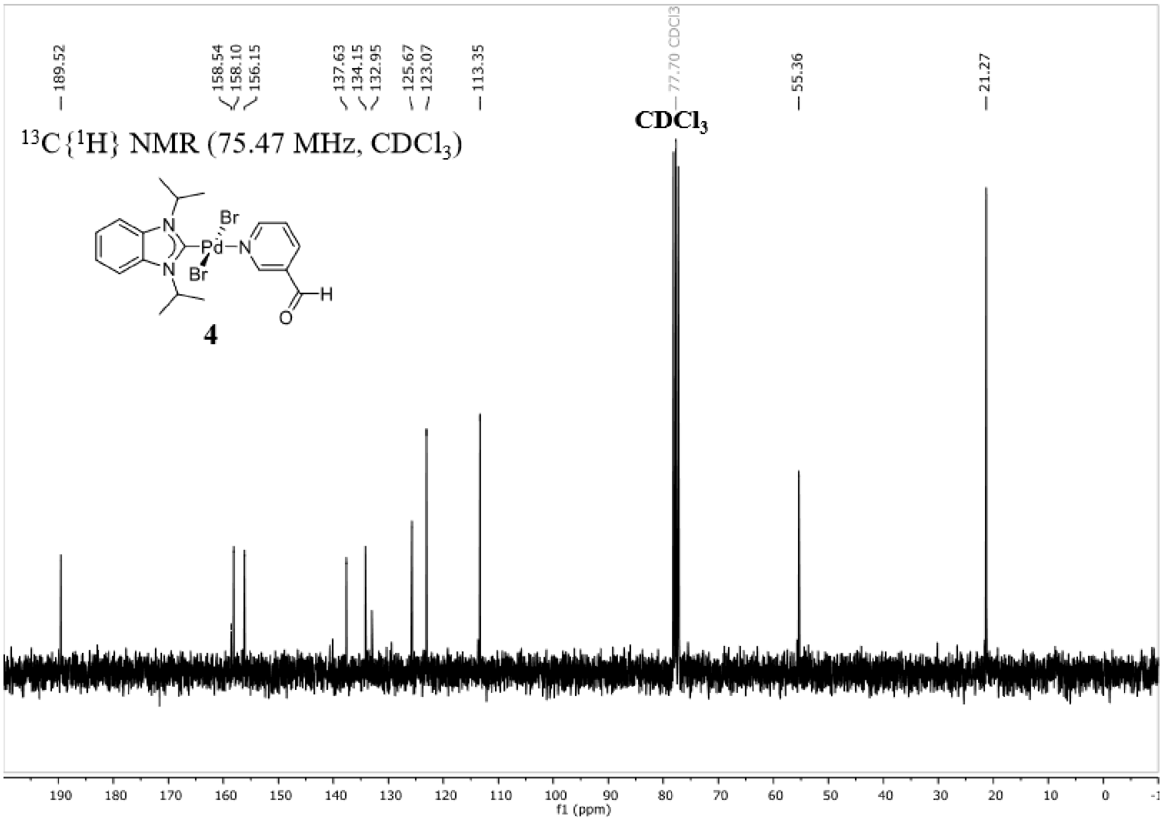

189.52
158.54 158.10 156.15
137.63 134.15 132.95 125.67 123.07 113.35
77.70 CDCl3
55.36
21.27
CDCl3
13C{1H} NMR (75.47 MHz, CDCl3)
Br
Pd
N
Br
H
O
4
f1 (ppm)

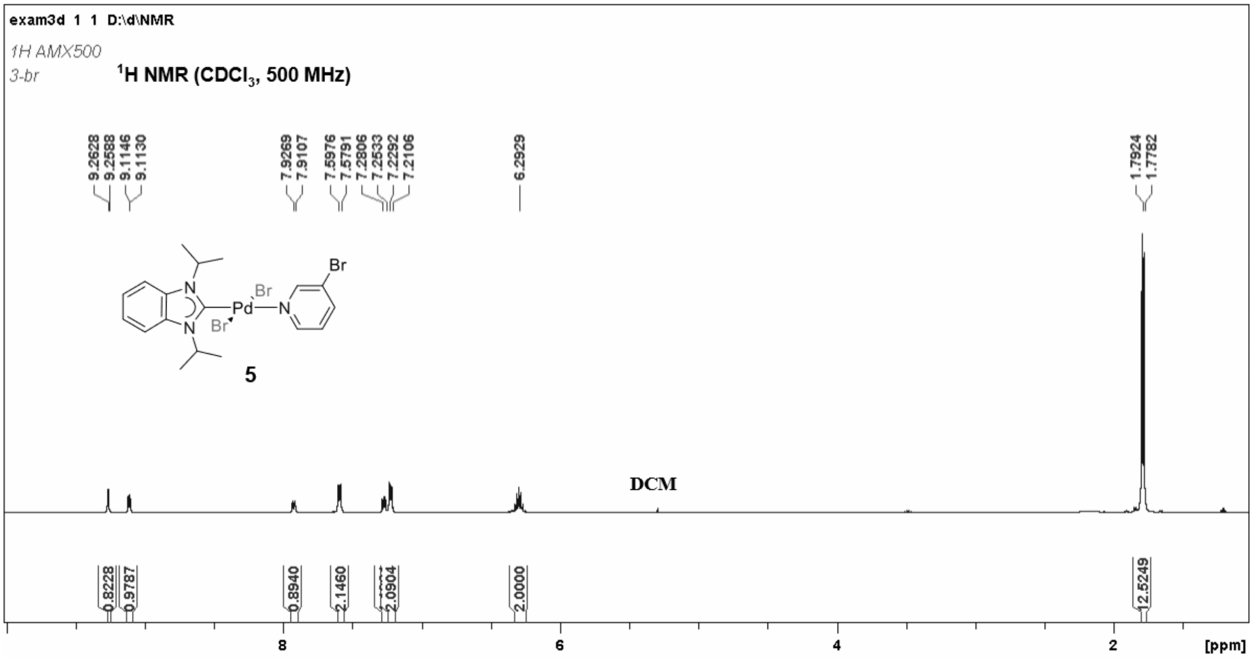
exam3d 1 1 D:\d\NMR
1H AMX500
3-br
1H NMR (CDCl3, 500 MHz)
9.2628
9.2588
9.1146
9.1130
7.9269
7.9107
7.5976
7.5791
7.2806
7.2533
7.2292
7.2106
6.2929
1.7924
1.7782
Br
N
Pd
N
Br
Br
N
5
DCM
0.8228
0.9787
0.8940
2.1460
2.0904
2.0000
12.5249
8
6
4
2
[ppm]

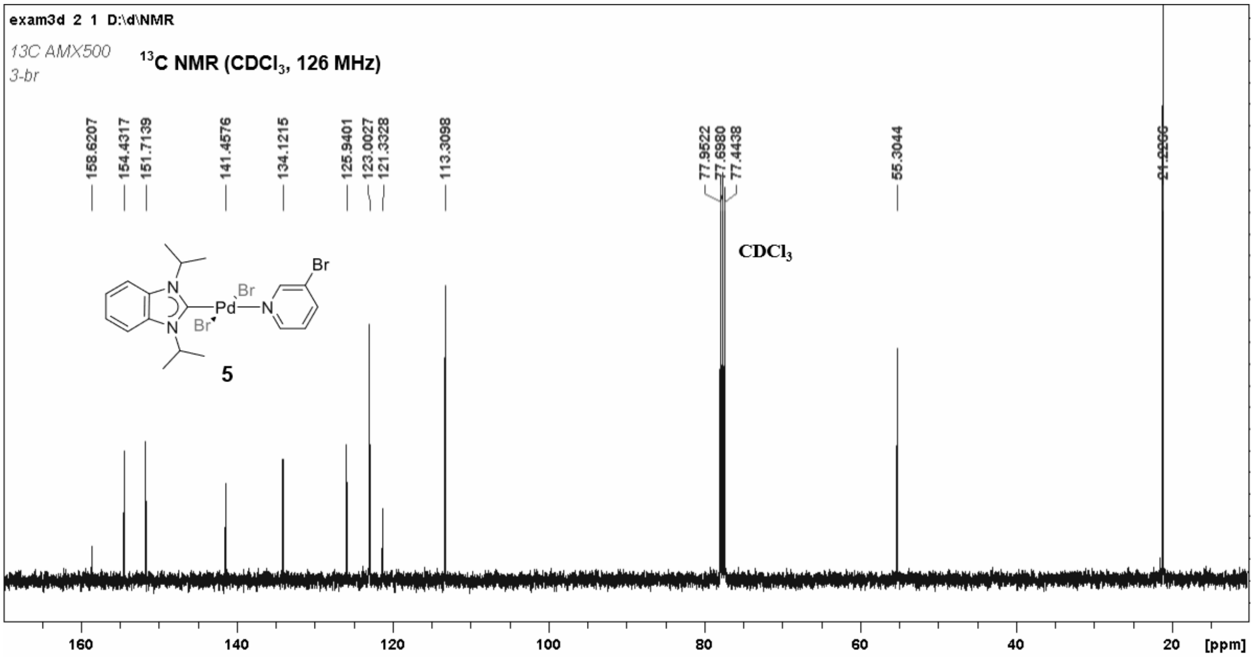
exam3d 2 1 D:\d\NMR
13C AMX500
3-br
13C NMR (CDCl3, 126 MHz)
158.6207
154.4317
151.7139
141.4576
134.1215
125.9401
123.0027
121.3328
113.3098
77.9522
77.6980
77.4438
55.3044
21.2266
Br
N
Pd
N
Br
Br
N
5
CDCl3
160
140
120
100
80
60
40
20
[ppm]

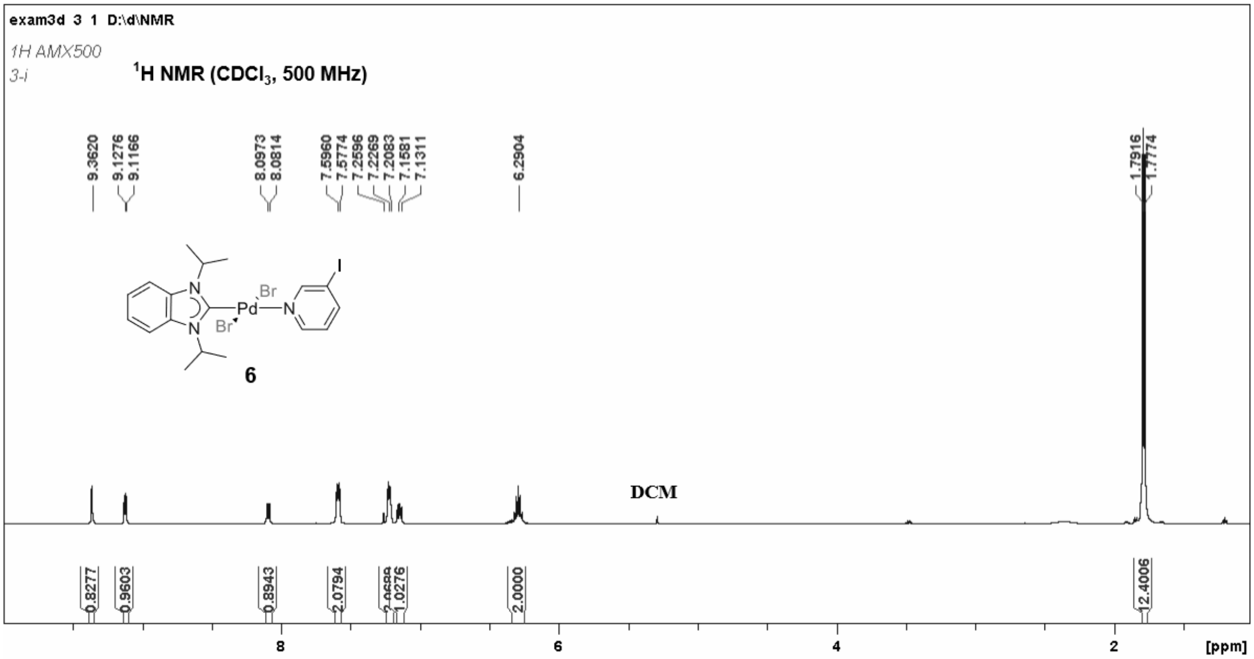
exam3d 3 1 D:\d\NMR
1H AMX500
3-i
^{1}H NMR ($CDCl_3$, 500 MHz)
9.3620 9.1276 9.1166 8.0973 8.0814 7.5960 7.5774 7.2596 7.2269 7.2083 7.1581 7.1311 6.2904 1.7916 1.7774
DCM
6
0.8277 0.9603 0.8943 2.0794 1.0276 2.0000 12.4006
8 6 4 2 [ppm]

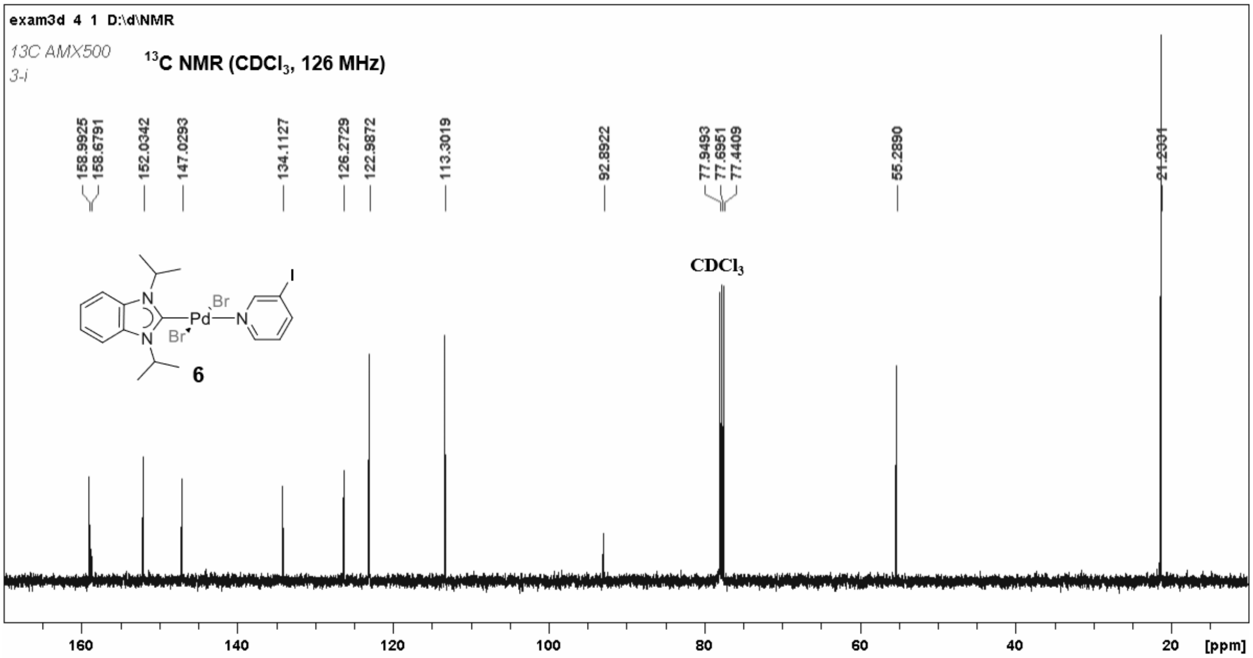
exam3d 4 1 D:\d\NMR
13C AMX500
3-i
^{13}C NMR ($CDCl_3$, 126 MHz)
158.9925 158.6791 152.0342 147.0293 134.1127 126.2729 122.9872 113.3019 92.8922 77.9493 77.6951 77.4409 55.2890 21.2331
$CDCl_3$
6
160 140 120 100 80 60 40 20 [ppm]

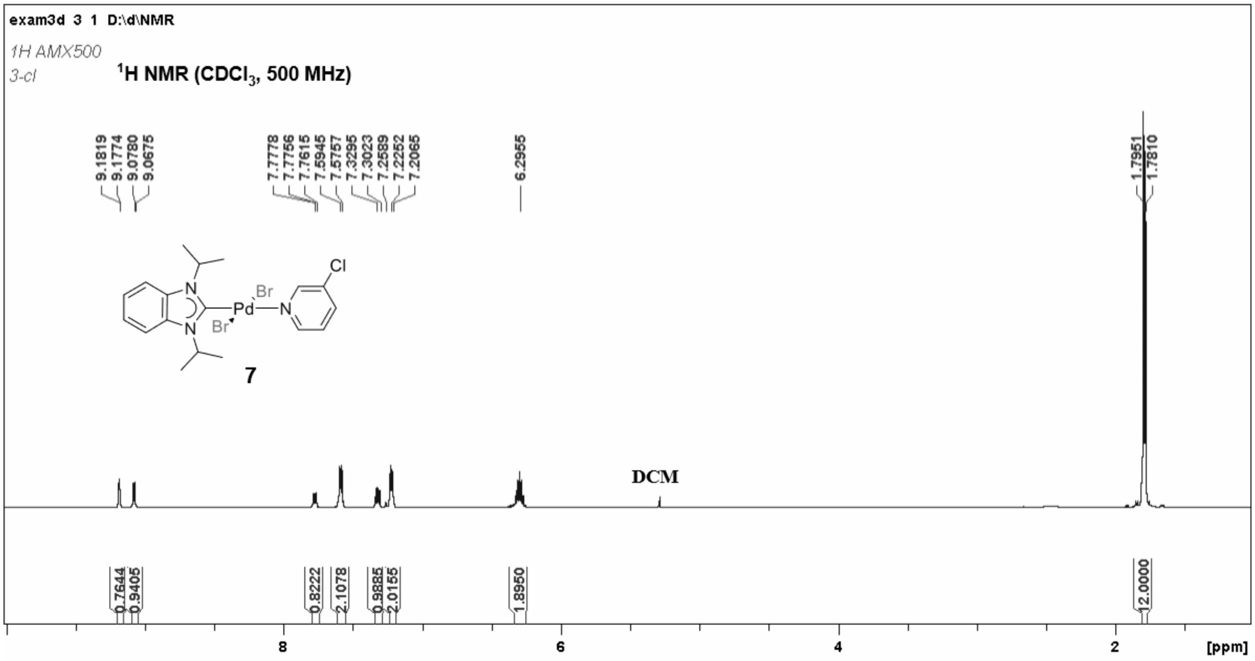
exam3d 3 1 D:\d\NMR
1H AMX500
3-cl
^{1}H NMR ($CDCl_3$, 500 MHz)
9.1819
9.1774
9.0780
9.0675
7.7778
7.7756
7.7615
7.5945
7.5757
7.3295
7.3023
7.2589
7.2252
7.2065
6.2955
1.7951
1.7810
Cl
Br
Pd
N
Br
7
DCM
0.7644
0.9405
0.8222
2.1078
0.9885
2.0155
1.8950
12.0000
8
6
4
2
[ppm]

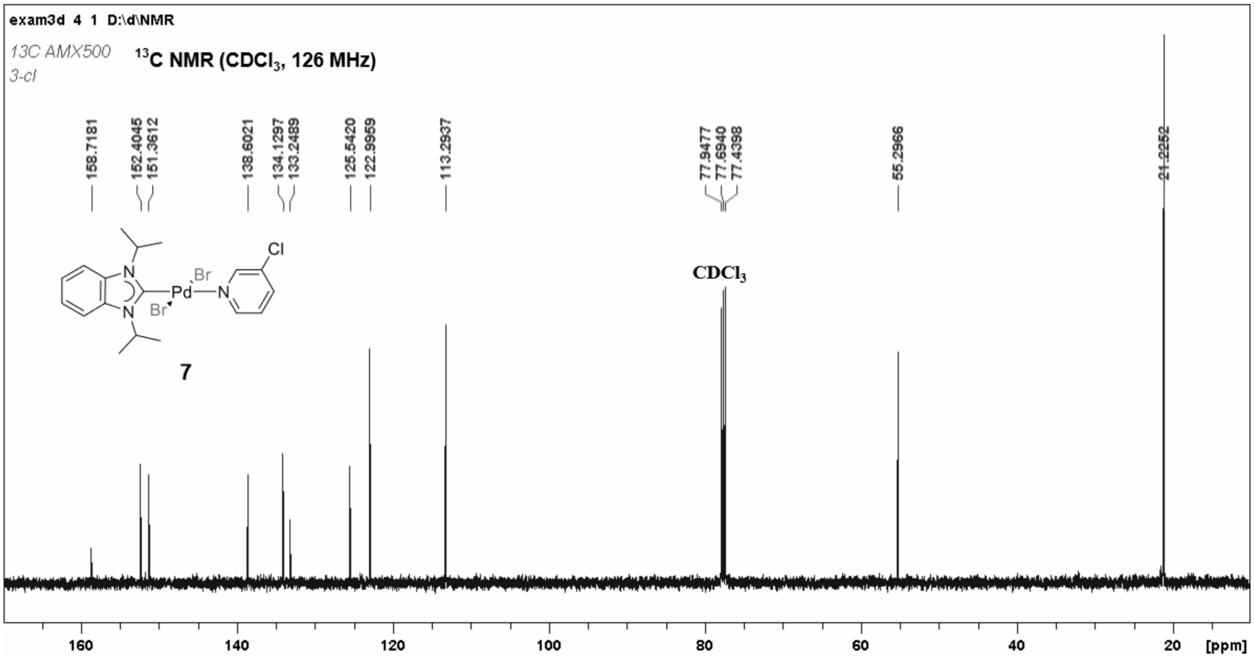
exam3d 4 1 D:\d\NMR
13C AMX500
3-cl
^{13}C NMR ($CDCl_3$, 126 MHz)
158.7181
152.4045
151.3612
138.6021
134.1297
133.2489
125.5420
122.9959
113.2937
77.9477
77.6940
77.4398
55.2966
21.2252
Cl
Br
Pd
N
Br
7
$CDCl_3$
160
140
120
100
80
60
40
20
[ppm]

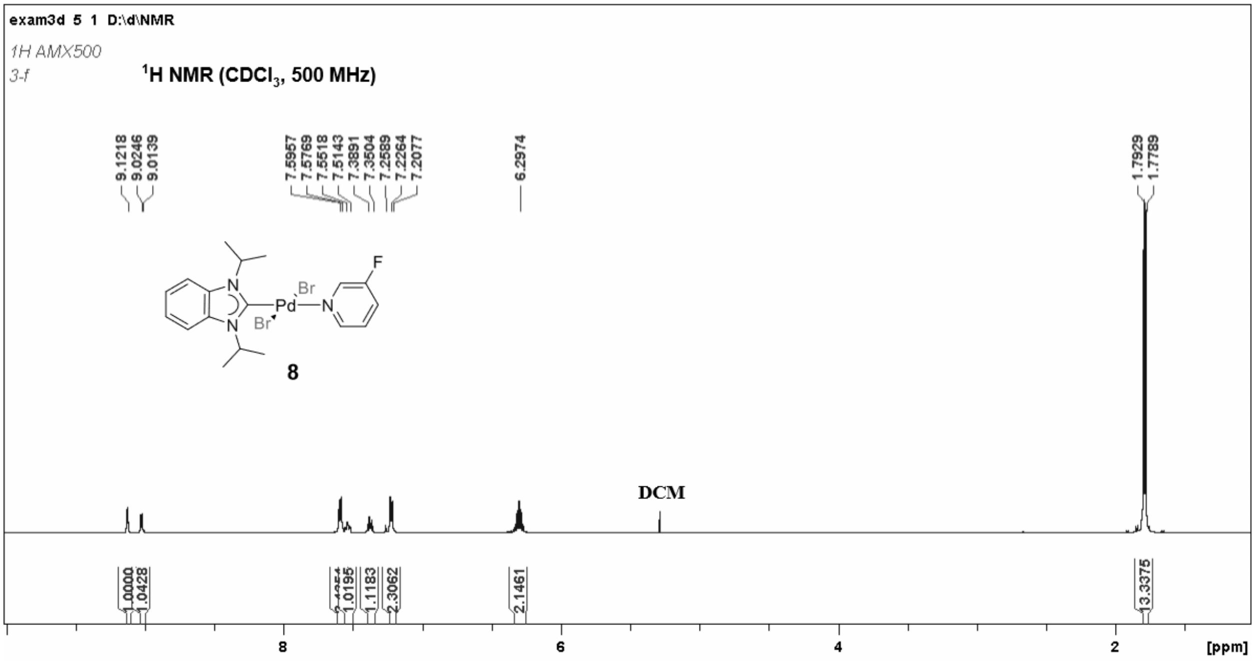

exam3d 5 1 D:\d\NMR
1H AMX500
3-f
^{1}H NMR ($CDCl_3$, 500 MHz)
9.1218
9.0246
9.0139
7.5957
7.5769
7.5518
7.5143
7.3891
7.3504
7.2589
7.2264
7.2077
6.2974
1.7929
1.7789
F
Br
N
Pd
Br
N
N
8
DCM
1.0000
1.0428
1.0195
1.1183
2.3062
2.1461
13.3375
8
6
4
2
[ppm]

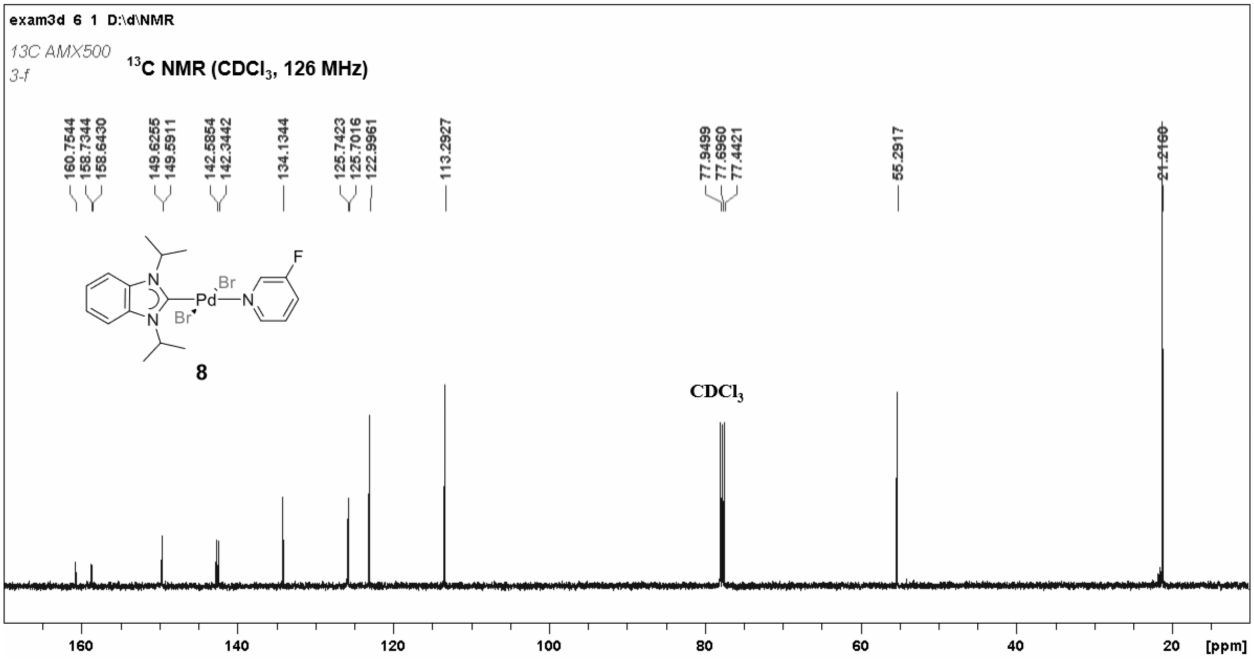

exam3d 6 1 D:\d\NMR
13C AMX500
3-f
^{13}C NMR ($CDCl_3$, 126 MHz)
160.7544
158.7344
158.6430
149.6255
149.5911
142.5854
142.3442
134.1344
125.7423
125.7016
122.9961
113.2927
77.9499
77.6960
77.4421
55.2917
21.2160
F
Br
N
Pd
Br
N
N
8
CDCl3
160
140
120
100
80
60
40
20
[ppm]

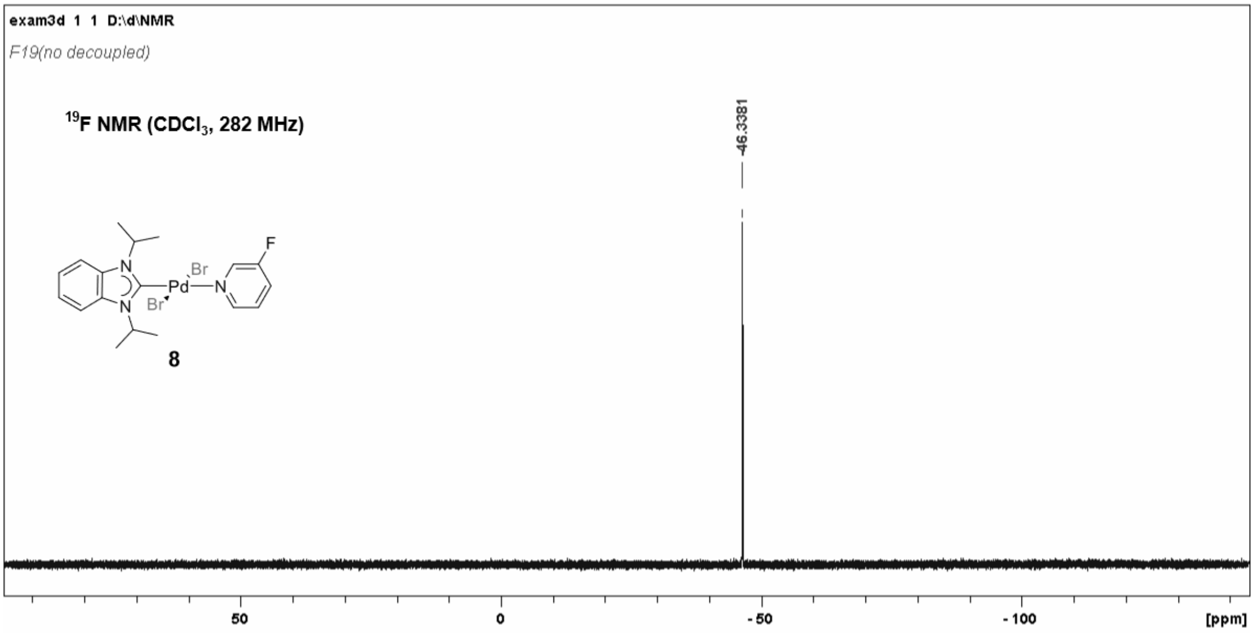
exam3d 1 1 D:\d\NMR
F19(no decoupled)
19F NMR (CDCl3, 282 MHz)
-46.3381
8
50
0
- 50
- 100
[ppm]

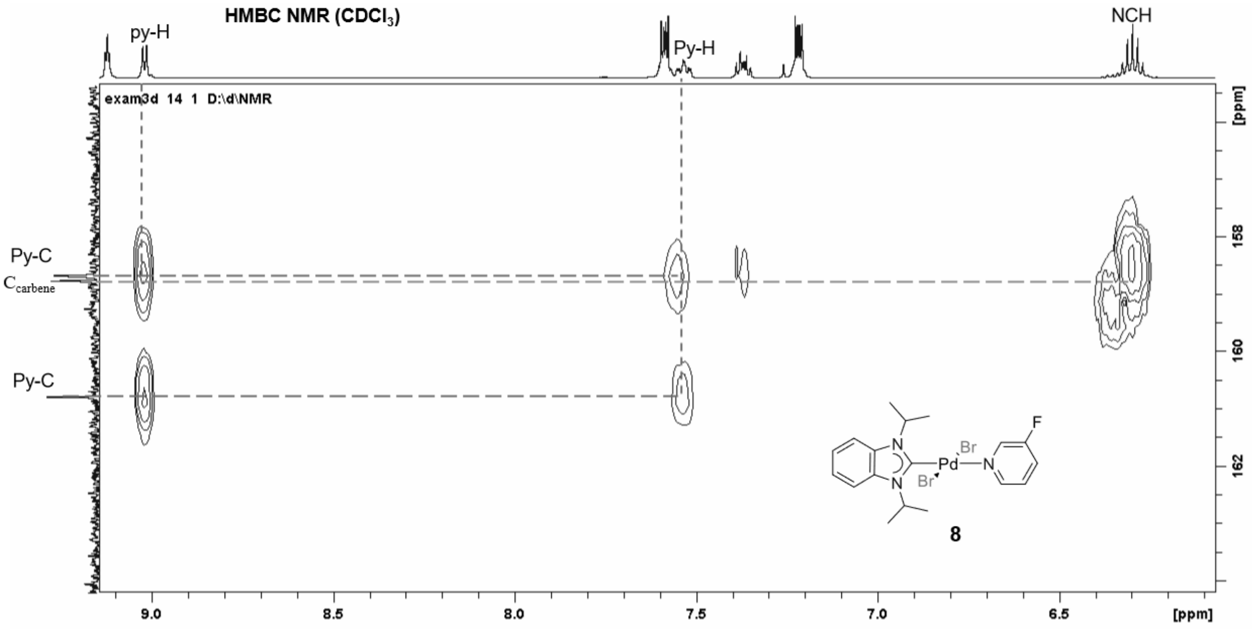
HMBC NMR (CDCl3)
py-H
Py-H
NCH
exam3d 14 1 D:\d\NMR
Py-C
Ccarbene
Py-C
8
158
160
162
[ppm]
9.0
8.5
8.0
7.5
7.0
6.5
[ppm]

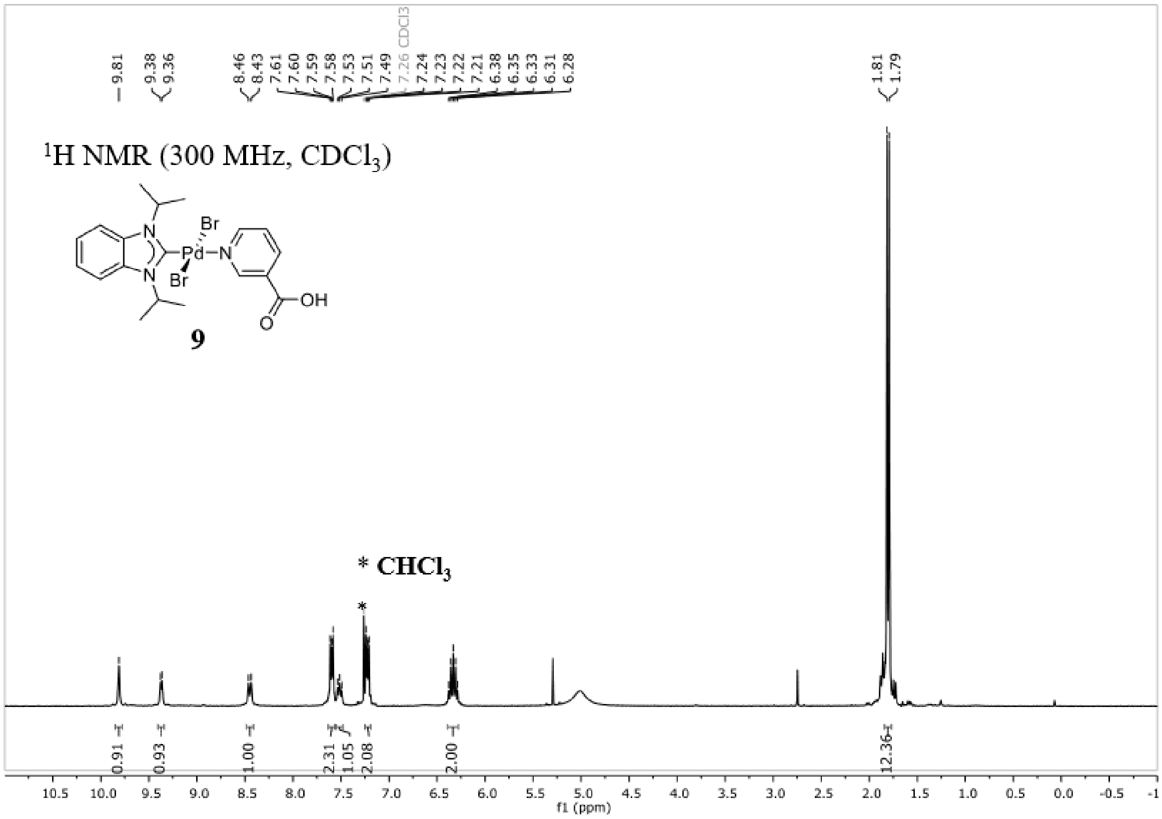

1H NMR (300 MHz, CDCl3)
9
* CHCl3
f1 (ppm)

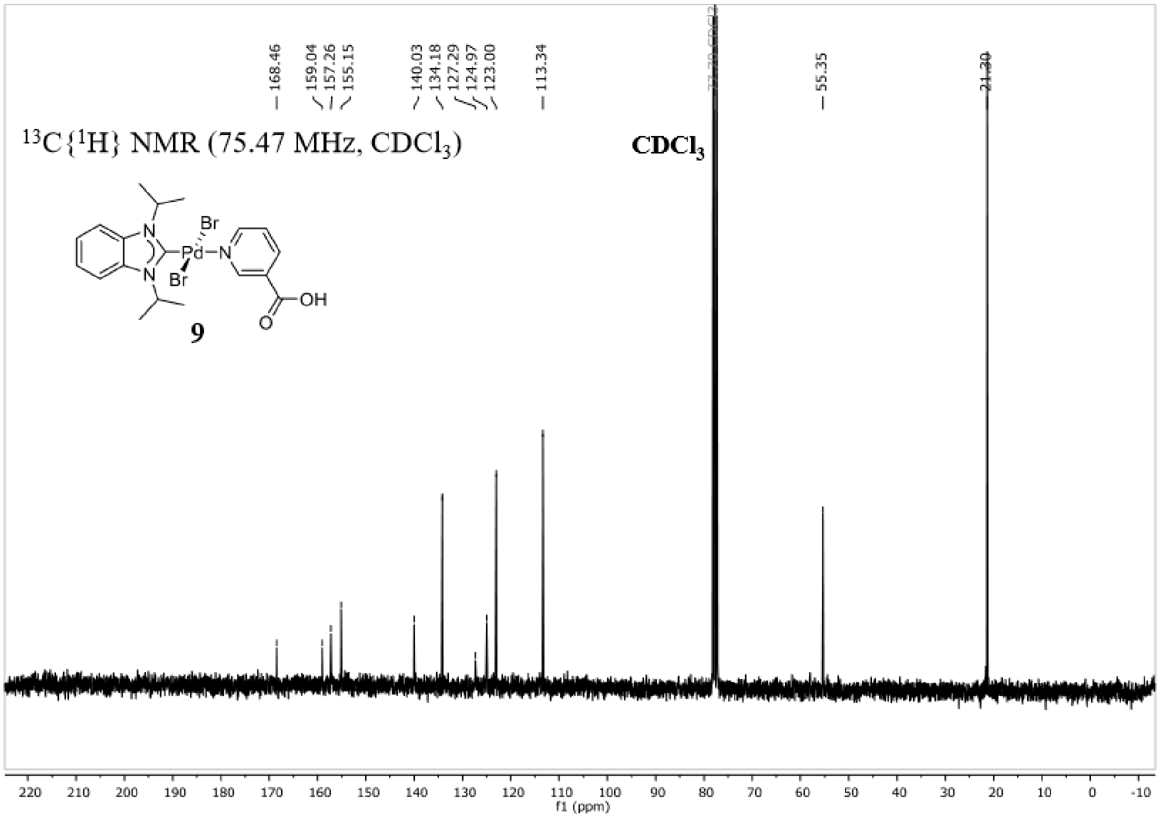

13C{1H} NMR (75.47 MHz, CDCl3)
9
CDCl3
f1 (ppm)

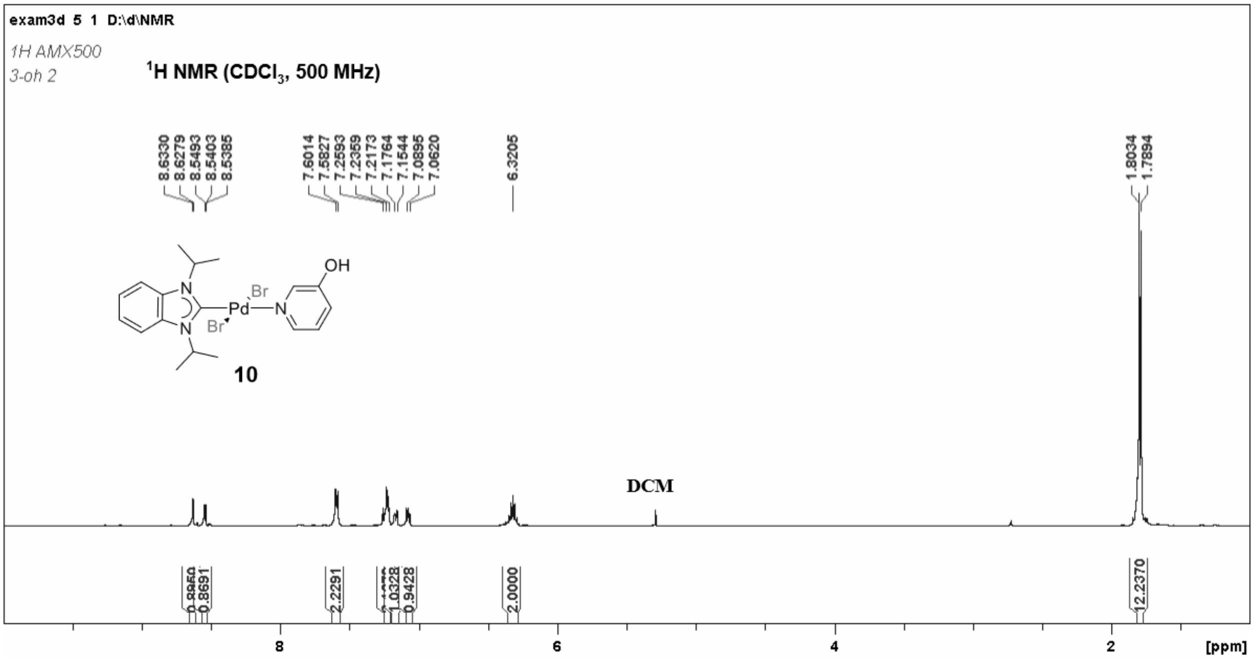
exam3d 5 1 D:\d\NMR
1H AMX500
3-oh 2
1H NMR (CDCl3, 500 MHz)
8.6330
8.6279
8.5493
8.5403
8.5385
7.6014
7.5827
7.2593
7.2359
7.2173
7.1764
7.1544
7.0895
7.0620
6.3205
1.8034
1.7894
OH
Br
Pd
N
Br
10
DCM
0.8691
2.2291
1.0328
0.9428
2.0000
12.2370
8
6
4
2
[ppm]

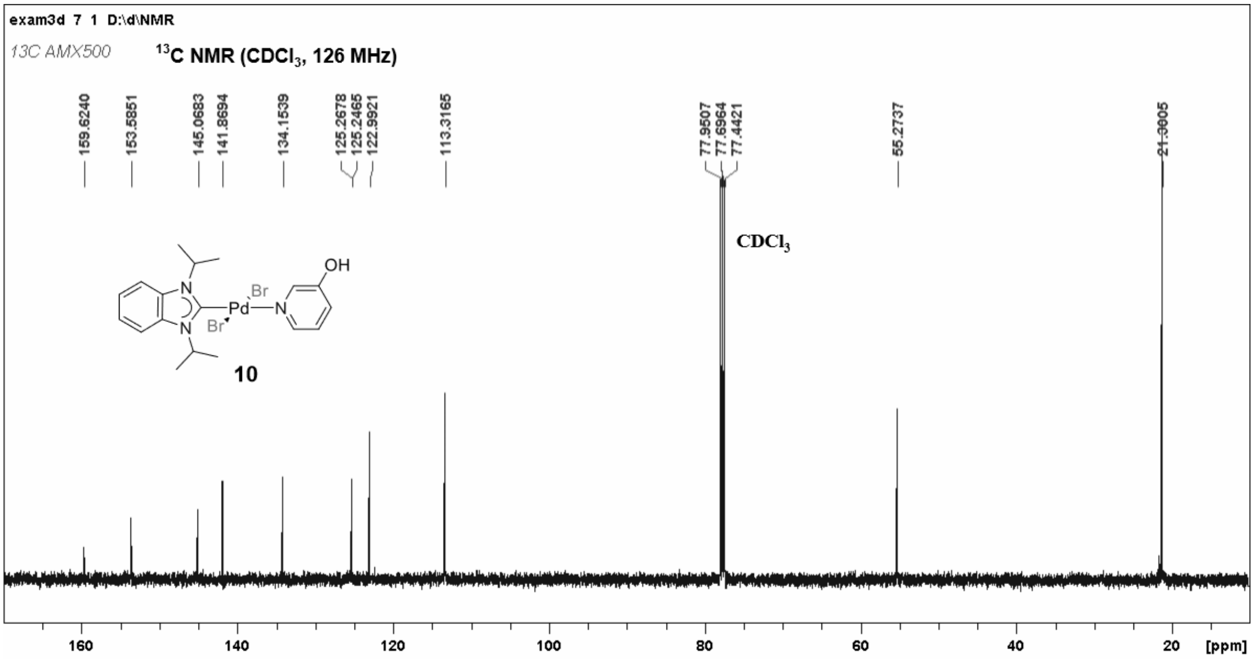
exam3d 7 1 D:\d\NMR
13C AMX500
13C NMR (CDCl3, 126 MHz)
159.6240
153.5851
145.0683
141.8694
134.1539
125.2678
125.2465
122.9921
113.3165
77.9507
77.6964
77.4421
55.2737
OH
Br
Pd
N
Br
10
CDCl3
160
140
120
100
80
60
40
20
[ppm]

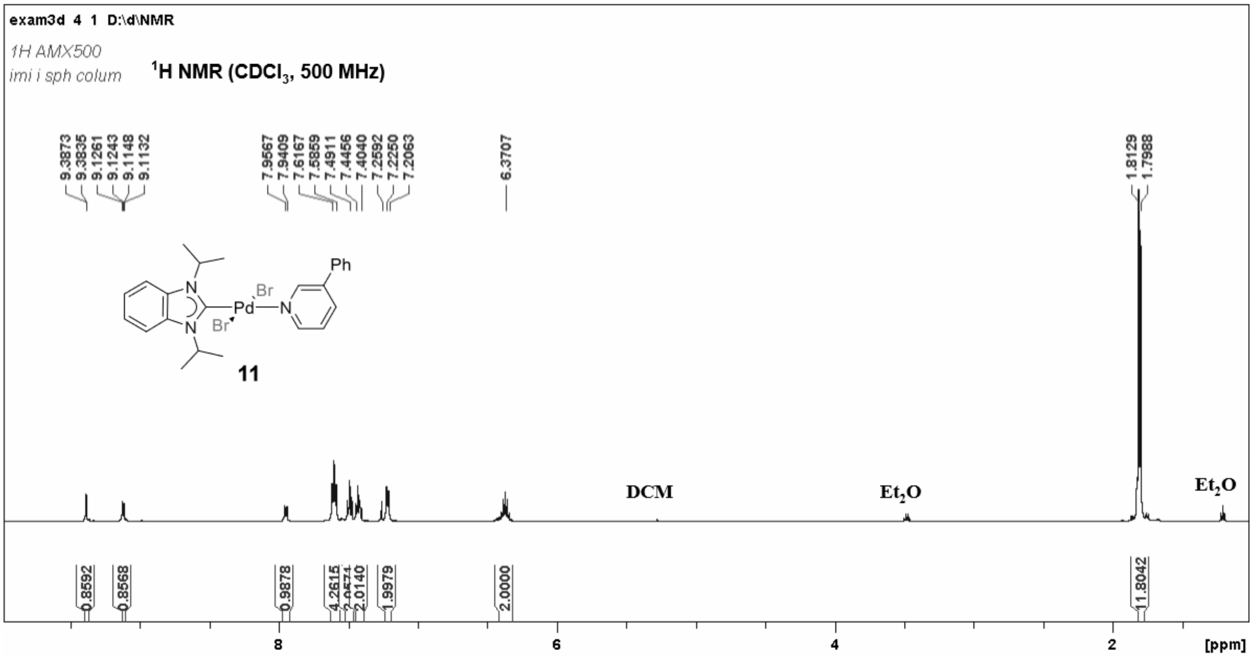

exam3d 4 1 D:\d\NMR
1H AMX500
imi i sph colum
^{1}H NMR ($CDCl_3$, 500 MHz)
9.3873
9.3835
9.1261
9.1243
9.1148
9.1132
7.9567
7.9409
7.6167
7.5859
7.4911
7.4456
7.4040
7.2592
7.2250
7.2063
6.3707
1.8129
1.7988
Ph
Br
Pd
N
11
DCM
Et_2O
Et_2O
0.8592
0.8568
0.9878
4.2615
2.0140
1.9979
2.0000
11.8042
8
6
4
2
[ppm]

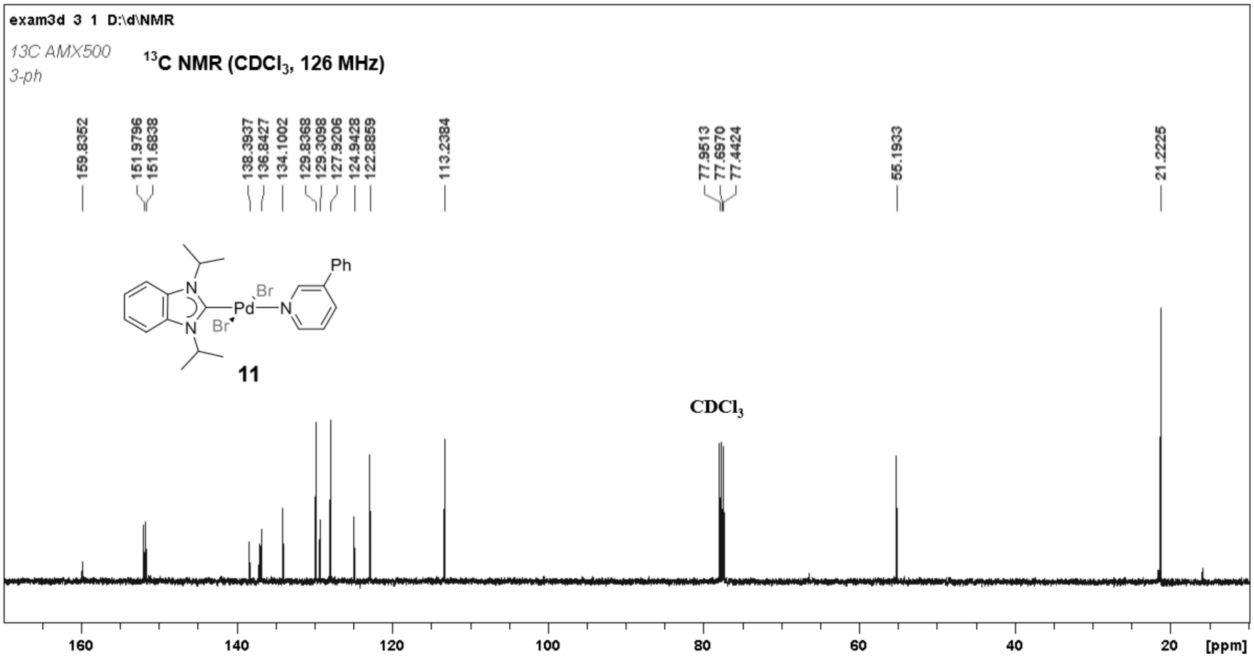

exam3d 3 1 D:\d\NMR
13C AMX500
3-ph
^{13}C NMR ($CDCl_3$, 126 MHz)
159.8352
151.9796
151.6838
138.3937
136.8427
134.1002
129.8368
129.3098
127.9206
124.9428
122.8859
113.2384
77.9513
77.6970
77.4424
55.1933
21.2225
Ph
Br
Pd
N
11
$CDCl_3$
160
140
120
100
80
60
40
20
[ppm]

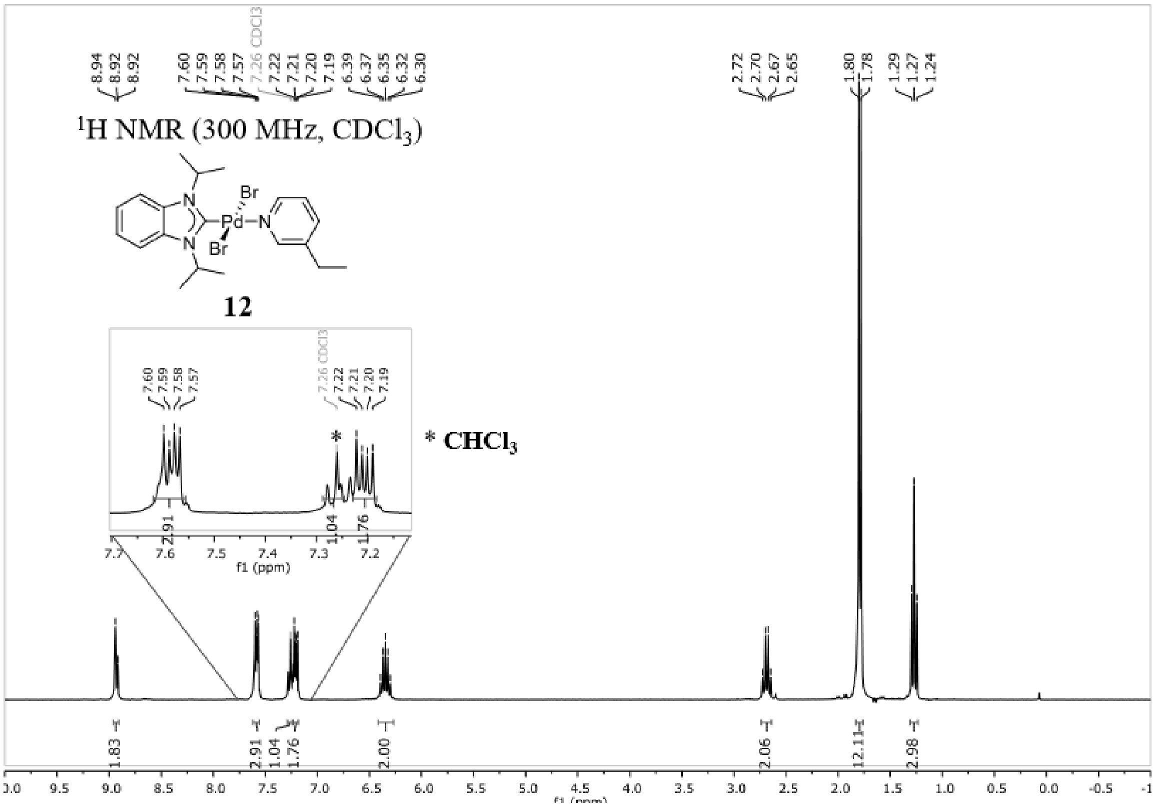

8.94
8.92
8.92
7.60
7.59
7.58
7.57
7.26 CDCl3
7.22
7.21
7.20
7.19
6.39
6.37
6.35
6.32
6.30
2.72
2.70
2.67
2.65
1.80
1.78
1.29
1.27
1.24
¹H NMR (300 MHz, CDCl₃)
Br
Pd
N
Br
12
* CHCl₃
2.91
1.04
1.76
f1 (ppm)
1.83
2.91
1.04
1.76
2.00
2.06
12.11
2.98

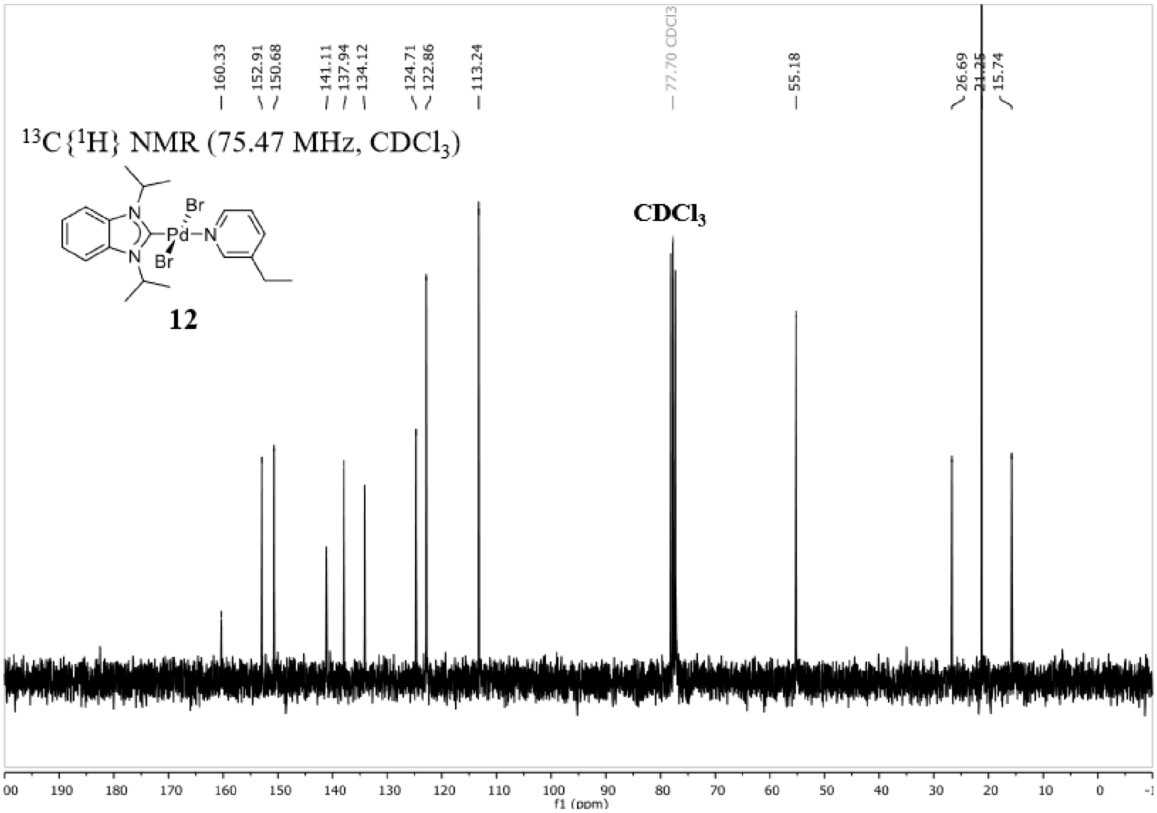

160.33
152.91
150.68
141.11
137.94
134.12
124.71
122.86
113.24
77.70 CDCl3
55.18
26.69
21.25
15.74
¹³C{¹H} NMR (75.47 MHz, CDCl₃)
Br
Pd
N
Br
12
CDCl₃
f1 (ppm)

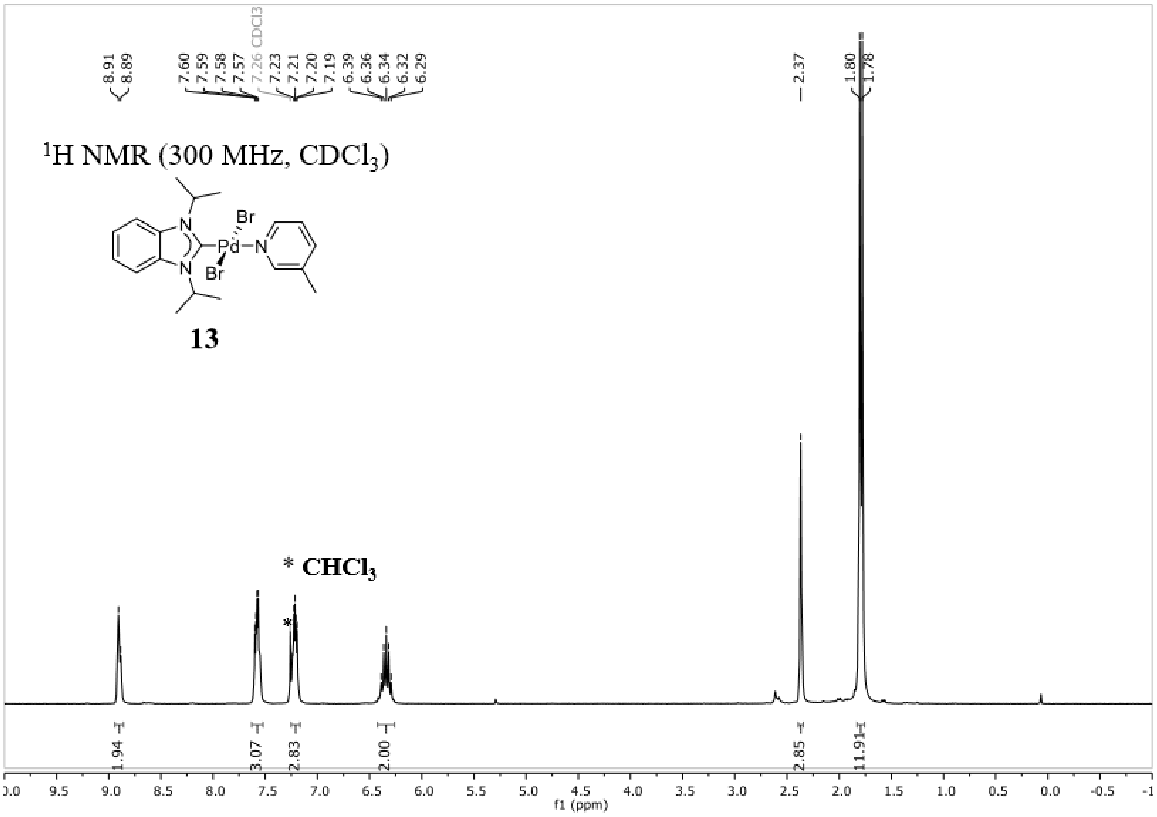
8.91
8.89
7.60
7.59
7.58
7.57
7.26 CDCl3
7.23
7.21
7.20
7.19
6.39
6.36
6.34
6.32
6.29
2.37
1.80
1.78
1H NMR (300 MHz, CDCl3)
Br
Pd
N
Br
13
* CHCl3
*
1.94
3.07
2.83
2.00
2.85
11.91
f1 (ppm)

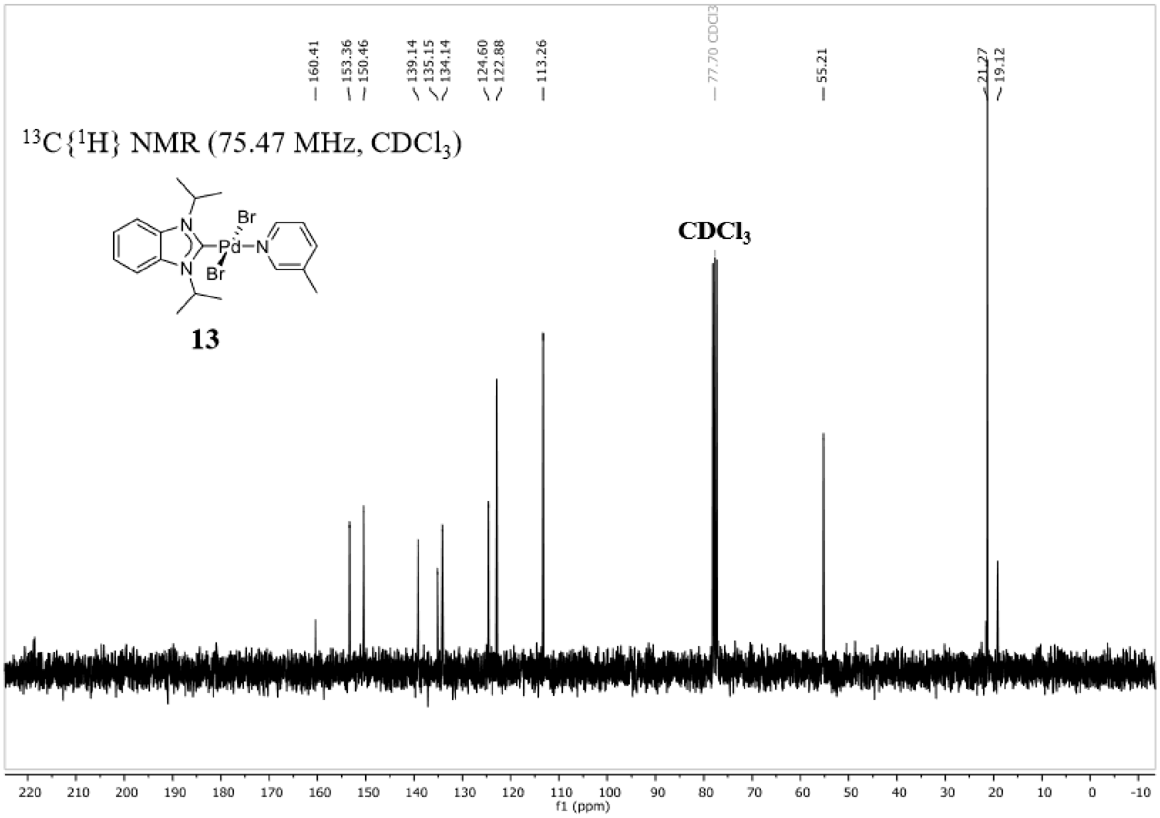
160.41
153.36
150.46
139.14
135.15
134.14
124.60
122.88
113.26
77.70 CDCl3
55.21
21.27
19.12
13C{1H} NMR (75.47 MHz, CDCl3)
Br
Pd
N
Br
13
CDCl3
f1 (ppm)

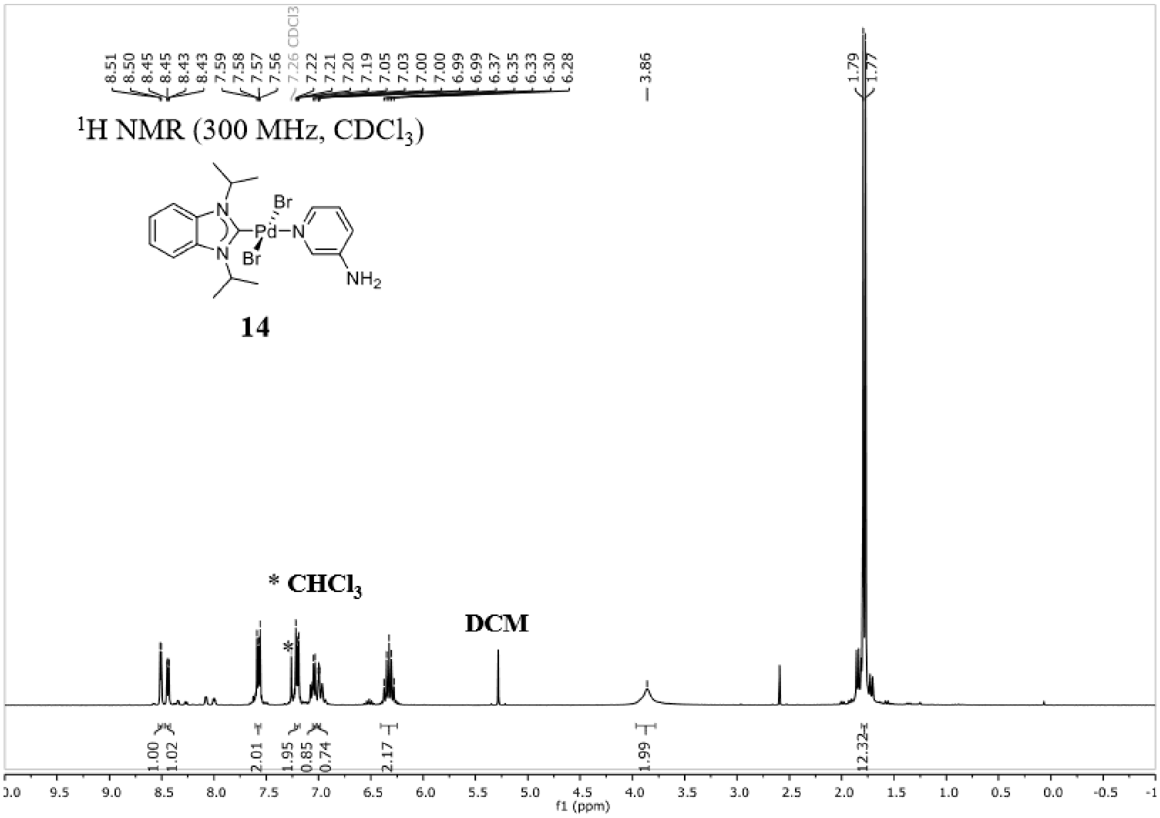

8.51
8.50
8.45
8.45
8.43
8.43
7.59
7.58
7.57
7.56
7.26 CDCl3
7.22
7.21
7.20
7.19
7.05
7.03
7.00
7.00
6.99
6.99
6.97
6.37
6.35
6.33
6.30
6.28
3.86
1.79
1.77
¹H NMR (300 MHz, CDCl3)
Br
N
Pd
N
Br
N
NH2
14
* CHCl3
*
DCM
1.00
1.02
2.01
1.95
0.85
0.74
2.17
1.99
12.32
9.5
9.0
8.5
8.0
7.5
7.0
6.5
6.0
5.5
5.0
4.5
4.0
3.5
3.0
2.5
2.0
1.5
1.0
0.5
0.0
-0.5
f1 (ppm)

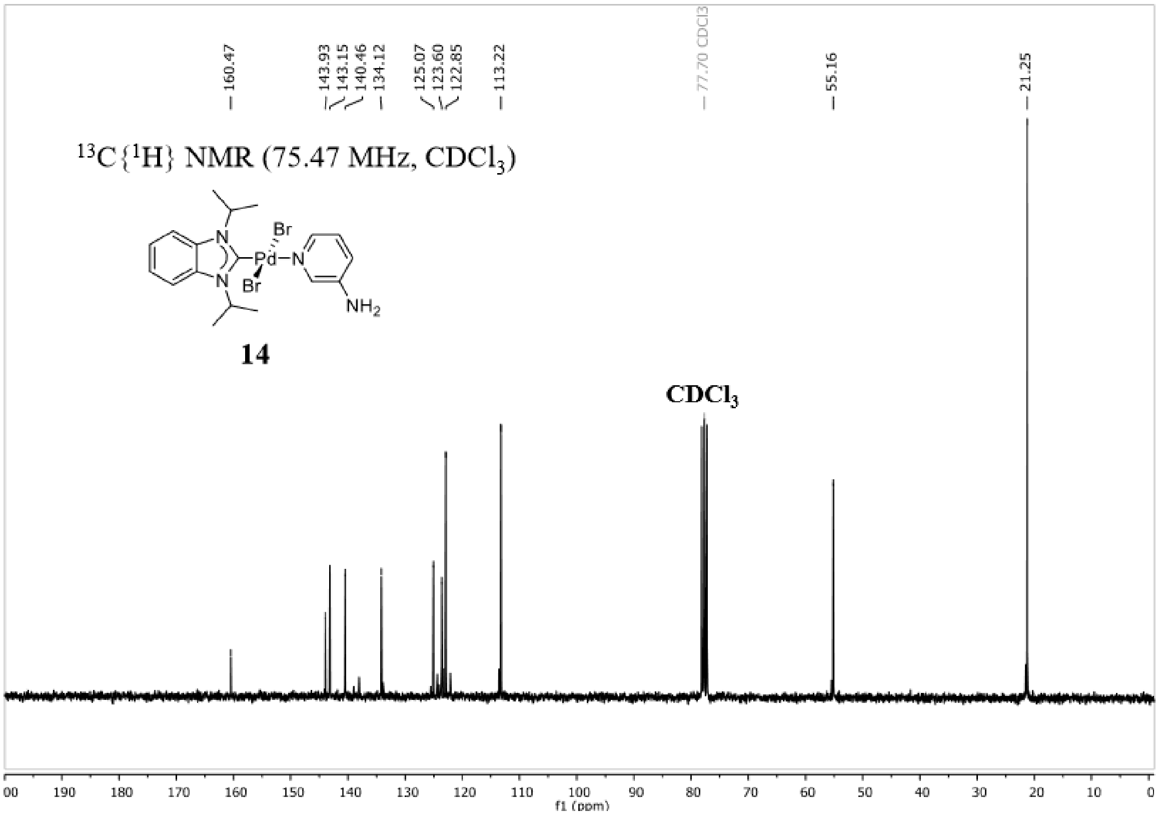

160.47
143.93
143.15
140.46
134.12
125.07
123.60
122.85
113.22
77.70 CDCl3
55.16
21.25
¹³C{¹H} NMR (75.47 MHz, CDCl3)
Br
N
Pd
N
Br
N
NH2
14
CDCl3
190
180
170
160
150
140
130
120
110
100
90
80
70
60
50
40
30
20
10
0
f1 (ppm)

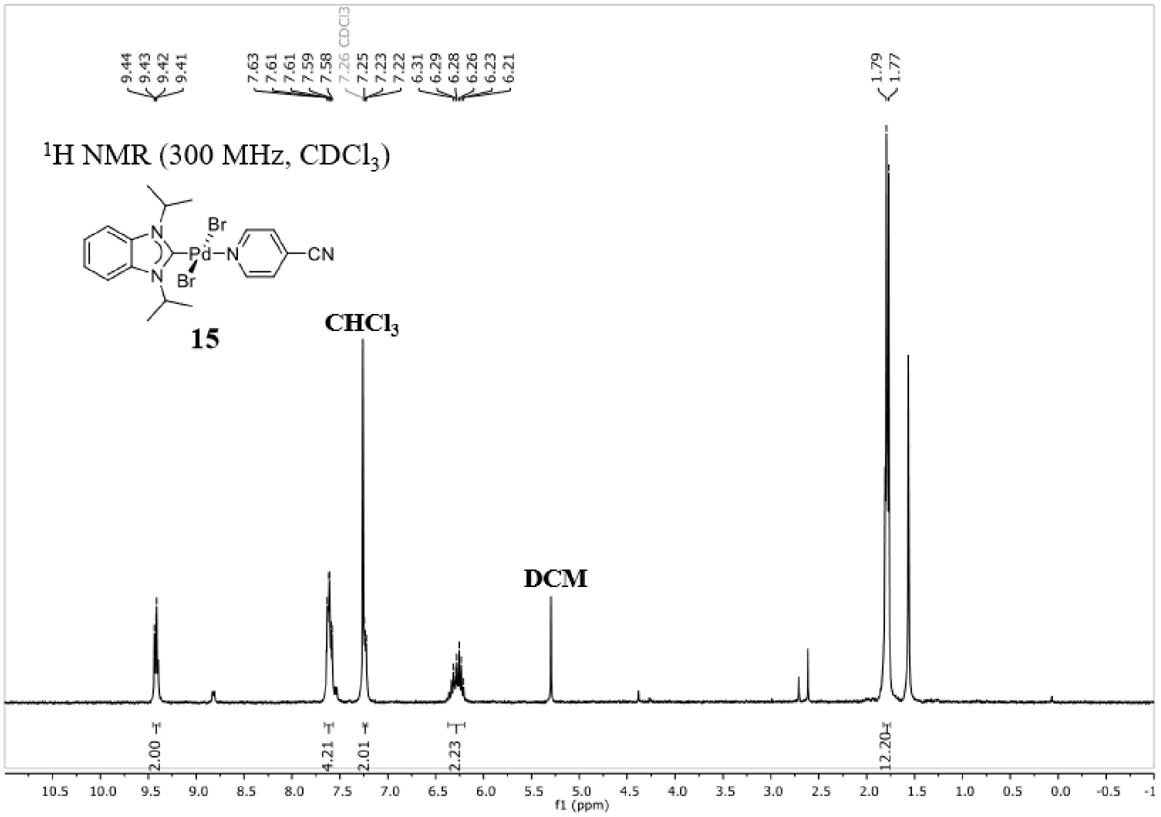
9.44
9.43
9.42
9.41
7.63
7.61
7.61
7.59
7.58
7.26 CDCl3
7.25
7.23
7.22
6.31
6.29
6.28
6.26
6.23
6.21
1.79
1.77
1H NMR (300 MHz, CDCl3)
Br
Pd
N
CN
Br
15
CHCl3
DCM
2.00
4.21
2.01
2.23
12.20
f1 (ppm)

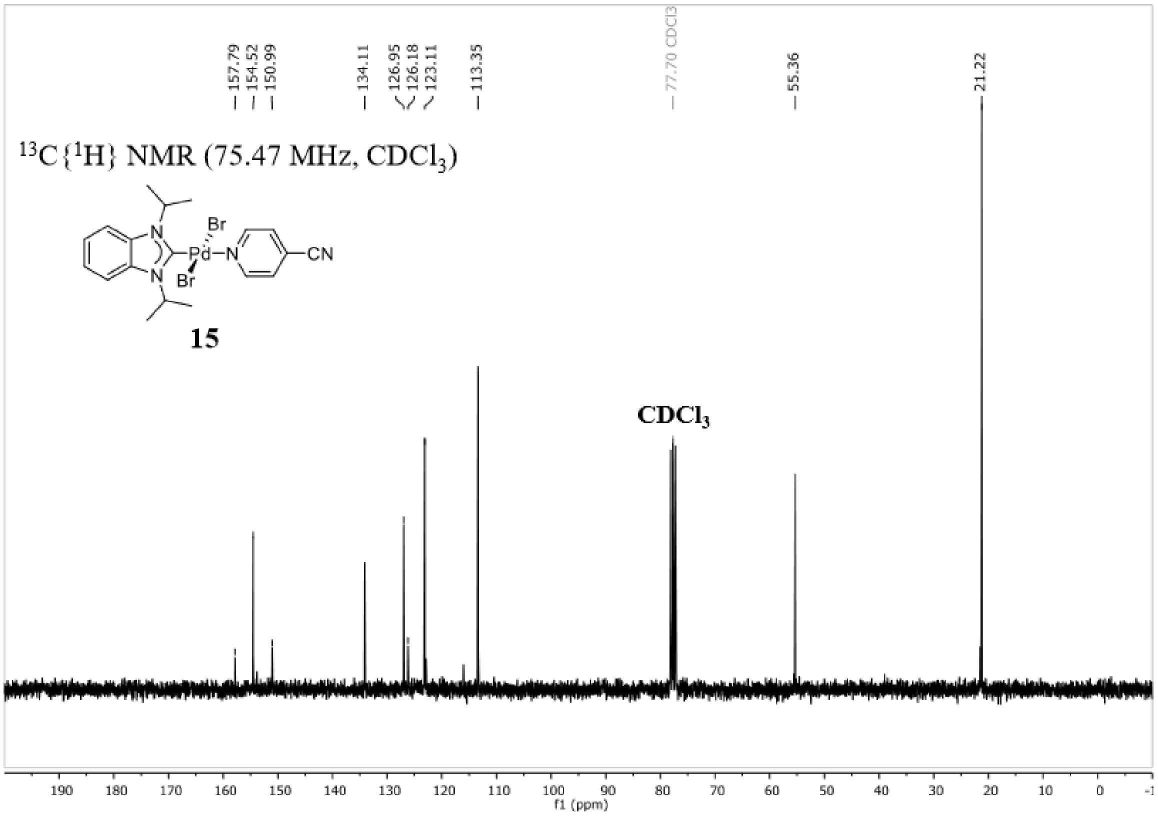
157.79
154.52
150.99
134.11
126.95
126.18
123.11
113.35
77.70 CDCl3
55.36
21.22
13C{1H} NMR (75.47 MHz, CDCl3)
Br
Pd
N
CN
Br
15
CDCl3
f1 (ppm)

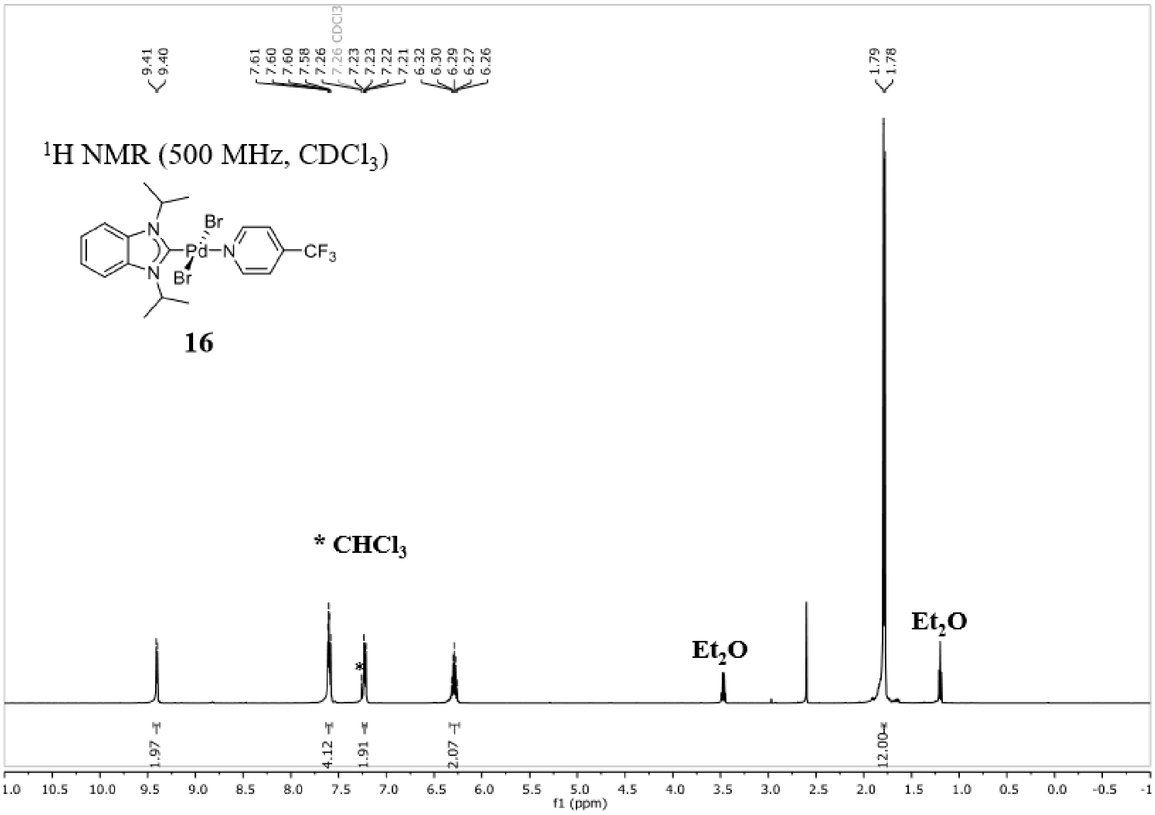
1H NMR (500 MHz, CDCl3)
16
* CHCl3
Et2O
Et2O
f1 (ppm)

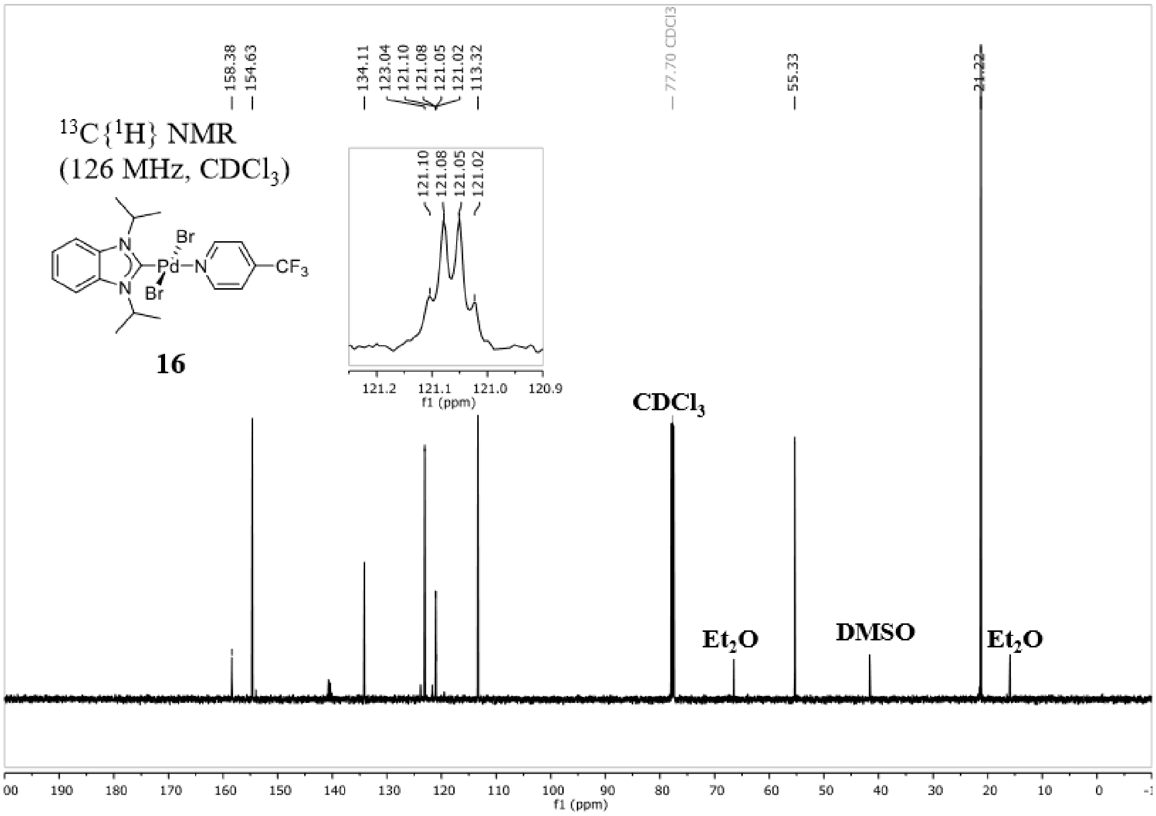
13C{1H} NMR
(126 MHz, CDCl3)
16
CDCl3
Et2O
DMSO
Et2O
f1 (ppm)

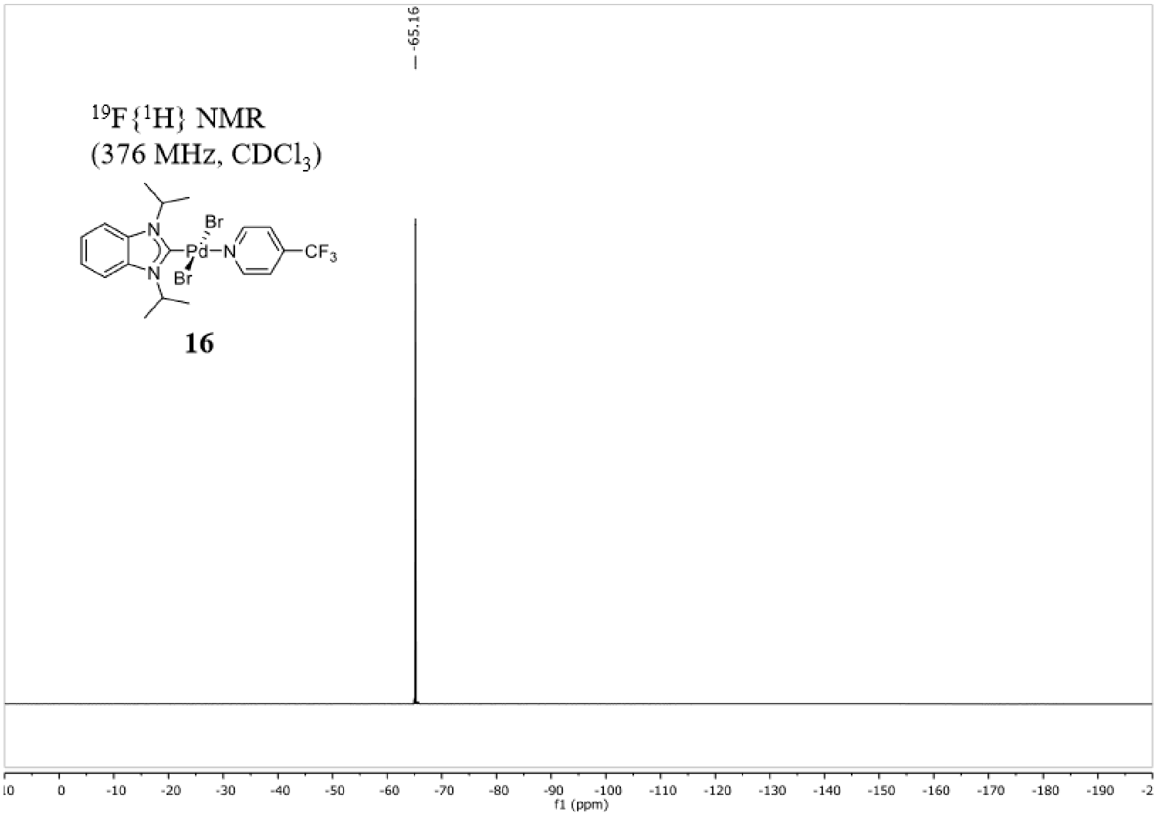

-65.16
19F{1H} NMR
(376 MHz, CDCl3)
Br
Pd
N
CF3
Br
16
f1 (ppm)

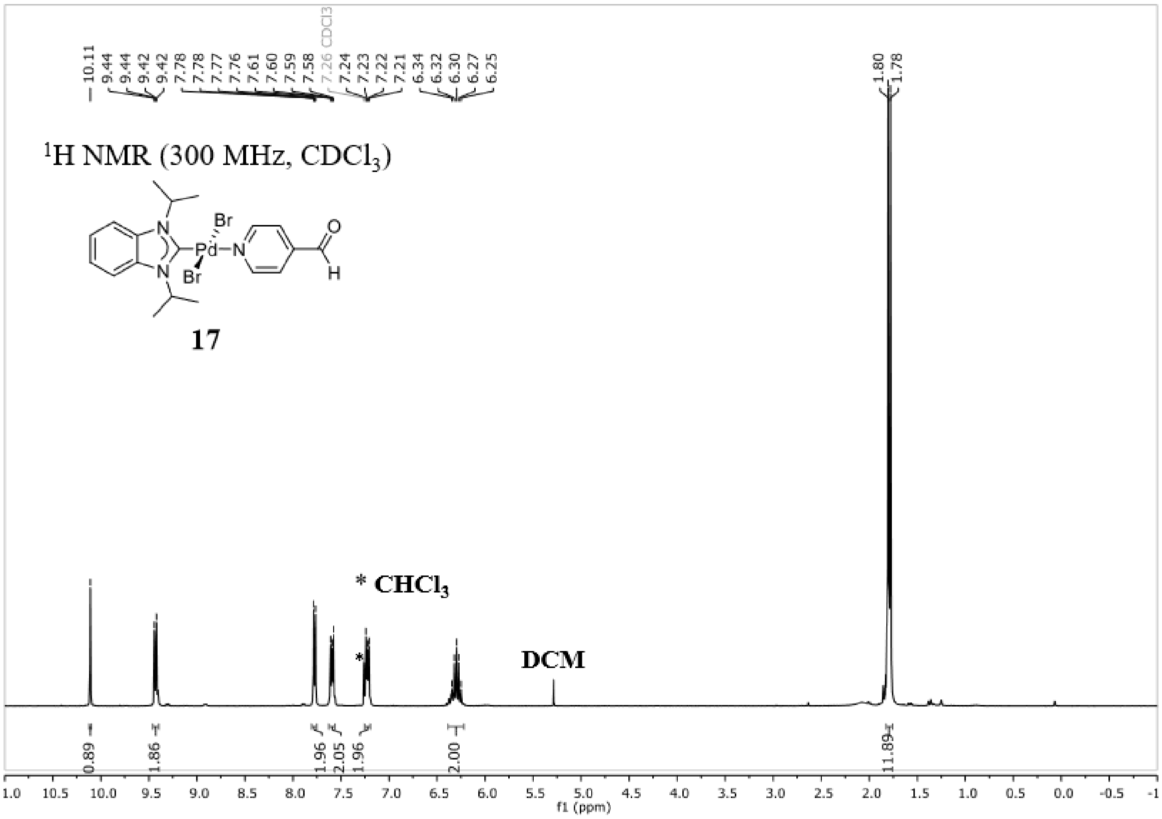

10.11
9.44
9.44
9.42
9.42
7.78
7.78
7.77
7.76
7.61
7.60
7.59
7.58
7.26 CDCl3
7.24
7.23
7.22
7.21
6.34
6.32
6.30
6.27
6.25
1.80
1.78
1H NMR (300 MHz, CDCl3)
Br
Pd
N
Br
O
H
17
* CHCl3
DCM
0.89
1.86
1.96
2.05
1.96
2.00
11.89
f1 (ppm)

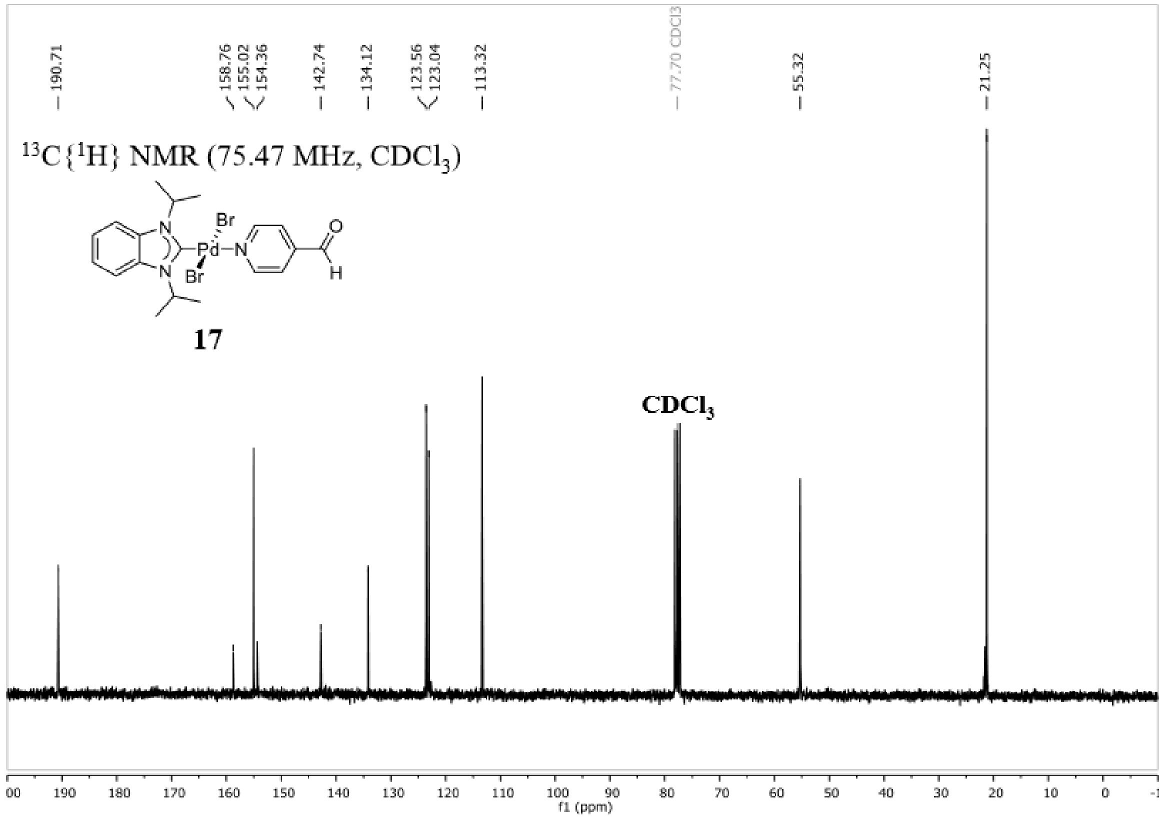
190.71
158.76
155.02
154.36
142.74
134.12
123.56
123.04
113.32
77.70 CDCl3
55.32
21.25
$^{13}C\{^1H\}$ NMR (75.47 MHz, $CDCl_3$)
Br
N
Pd
N
O
H
Br
N
17
$CDCl_3$
f1 (ppm)

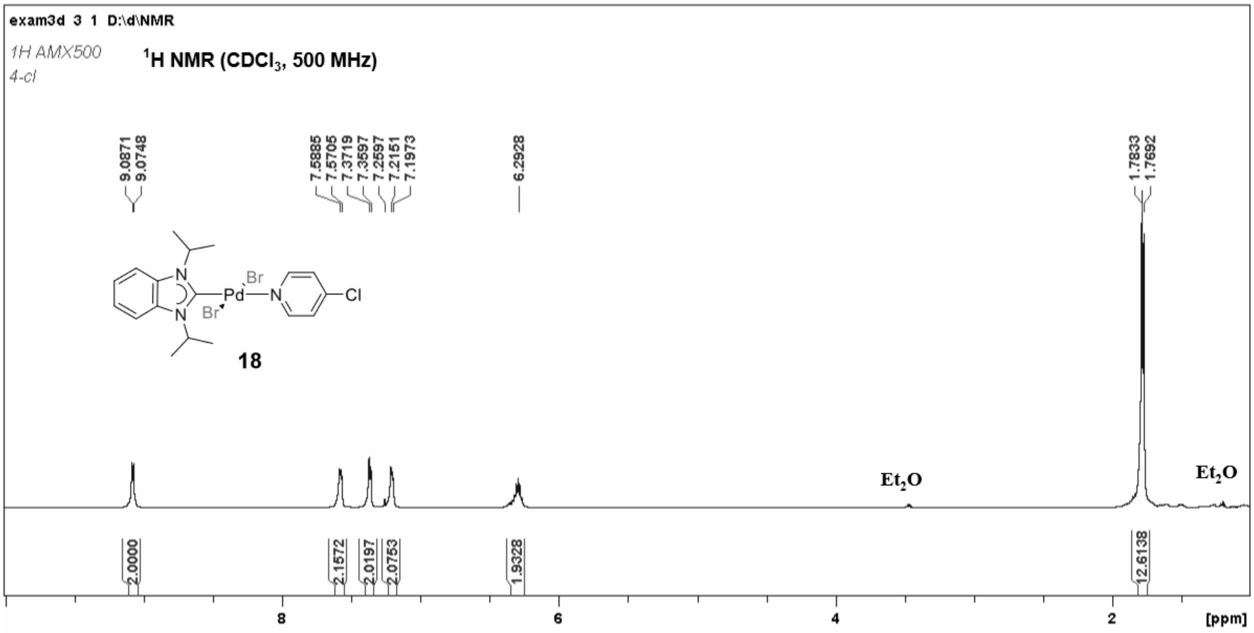
exam3d 3 1 D:\d\NMR
1H AMX500
4-cl
^1H NMR ($CDCl_3$, 500 MHz)
9.0871
9.0748
7.5885
7.5705
7.3719
7.3597
7.2597
7.2151
7.1973
6.2928
1.7833
1.7692
Br
N
Pd
N
Cl
Br
N
18
Et_2O
Et_2O
2.0000
2.1572
2.0197
2.0753
1.9328
12.6138
[ppm]

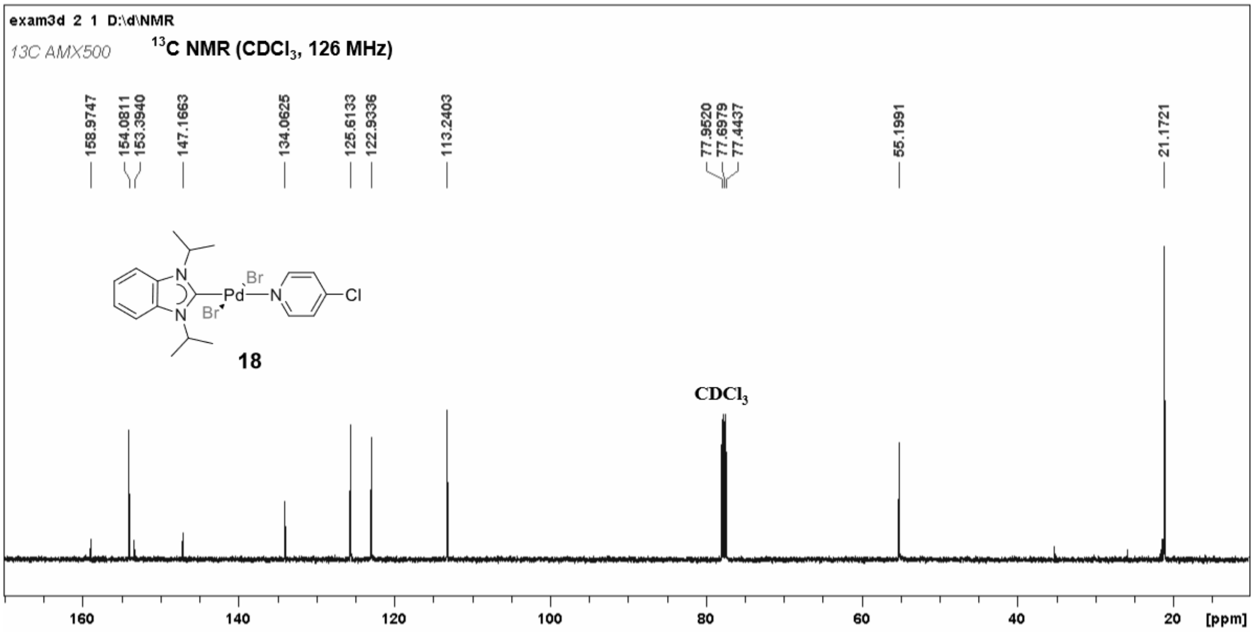
exam3d 2 1 D:\d\NMR
13C AMX500
^{13}C NMR ($CDCl_3$, 126 MHz)
158.9747
154.0811
153.3940
147.1663
134.0625
125.6133
122.9336
113.2403
77.9520
77.6979
77.4437
55.1991
21.1721
Br
Pd
N
Cl
18
$CDCl_3$
160
140
120
100
80
60
40
20
[ppm]

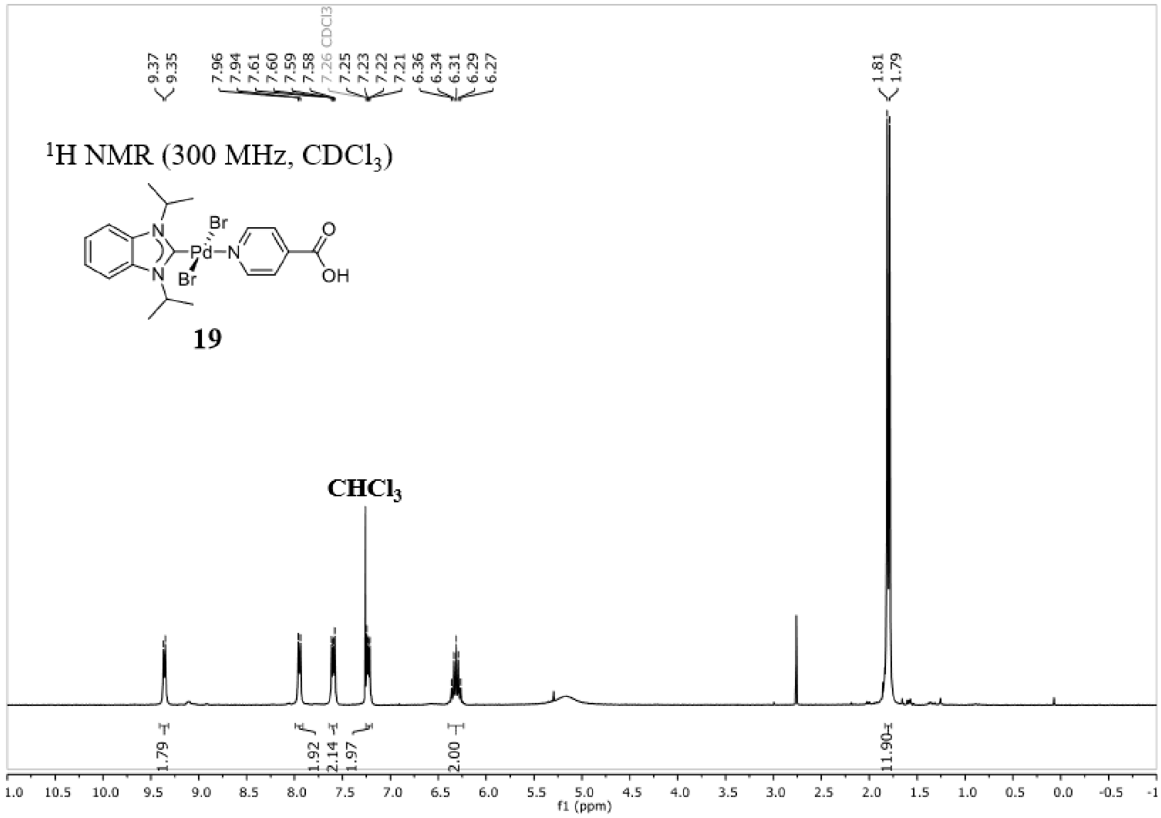
9.37
9.35
7.96
7.94
7.61
7.60
7.59
7.58
7.26 CDCl3
7.25
7.23
7.22
7.21
6.36
6.34
6.31
6.29
6.27
1.81
1.79
^{1}H NMR (300 MHz, $CDCl_3$)
Br
Pd
N
O
OH
19
$CHCl_3$
1.79
1.92
2.14
1.97
2.00
11.90
f1 (ppm)

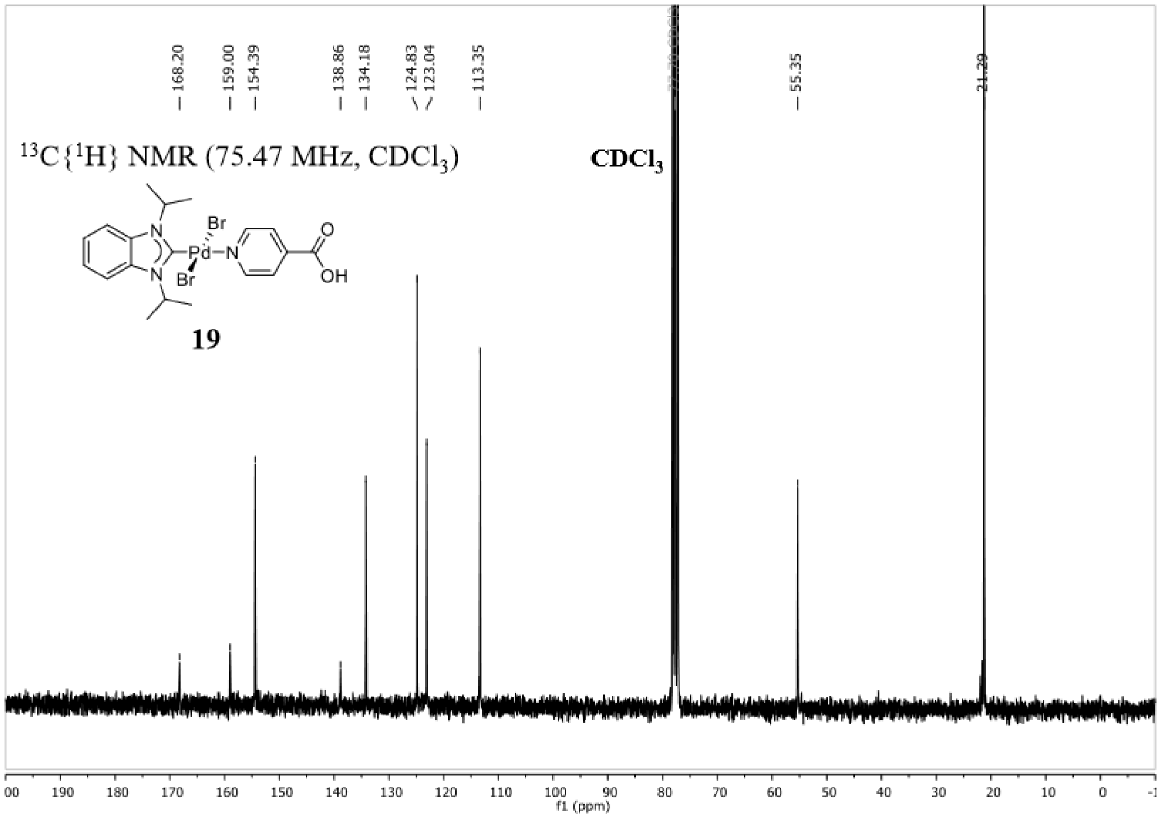
168.20
159.00
154.39
138.86
134.18
124.83
123.04
113.35
55.35
21.29
$^{13}C\{^{1}H\}$ NMR (75.47 MHz, $CDCl_3$)
$CDCl_3$
Br
Pd
N
O
OH
Br
N
N
19
00 190 180 170 160 150 140 130 120 110 100 90 80 70 60 50 40 30 20 10 0
f1 (ppm)

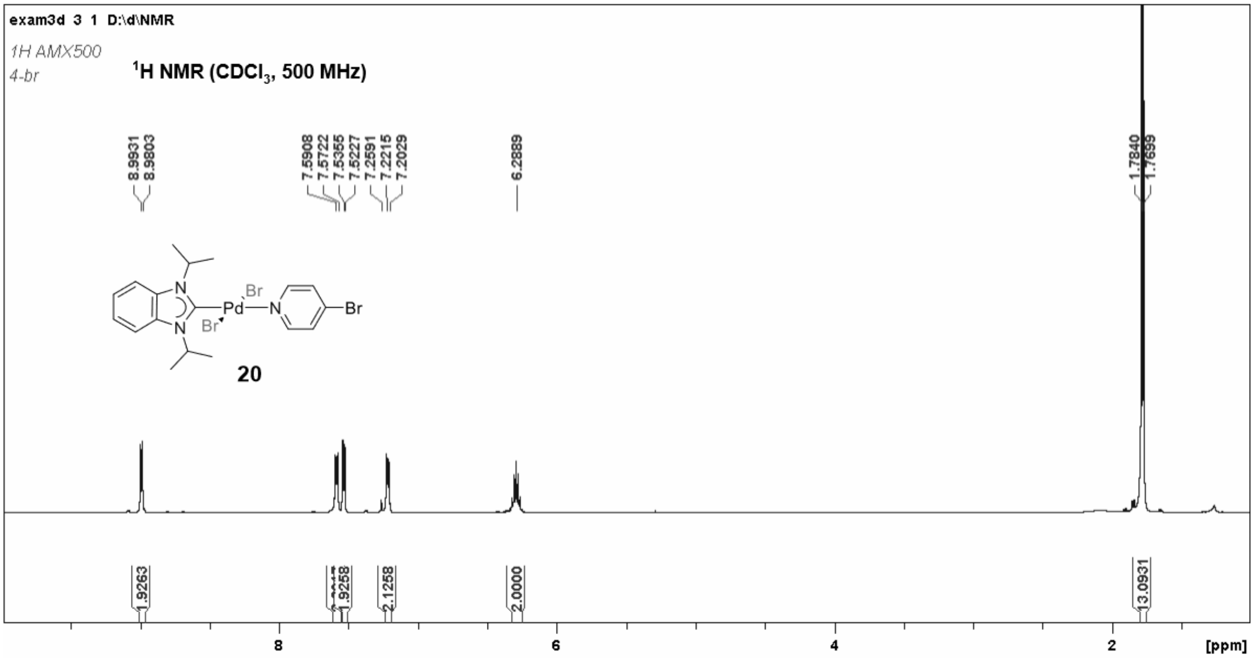
exam3d 3 1 D:\d\NMR
1H AMX500
4-br
^{1}H NMR ($CDCl_3$, 500 MHz)
8.9931
8.9803
7.5908
7.5722
7.5355
7.5227
7.2591
7.2215
7.2029
6.2889
1.7840
1.7699
Br
Pd
N
Br
Br
N
N
20
1.9263
1.9258
2.1258
2.0000
13.0931
8
6
4
2
[ppm]

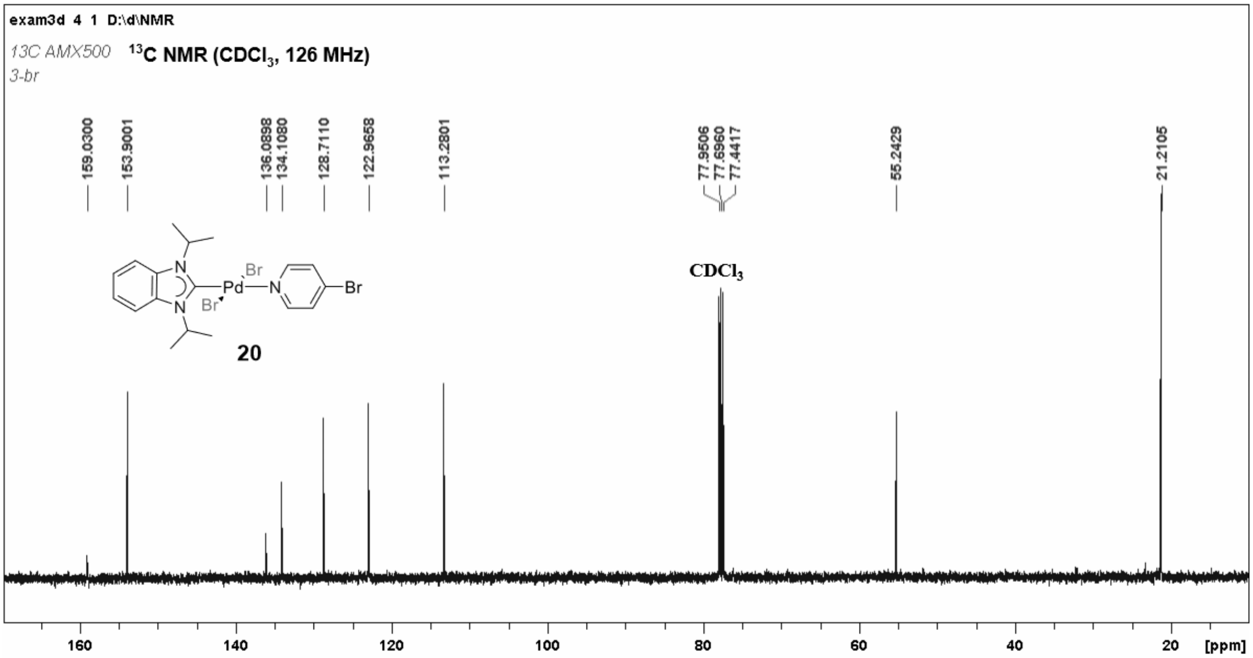
exam3d 4 1 D:\d\NMR
13C AMX500
3-br
^{13}C NMR (CDCl$_3$, 126 MHz)
159.0300
153.9001
136.0898
134.1080
128.7110
122.9658
113.2801
77.9506
77.6960
77.4417
55.2429
21.2105
CDCl$_3$
Br
Pd
N
Br
20
160
140
120
100
80
60
40
20
[ppm]

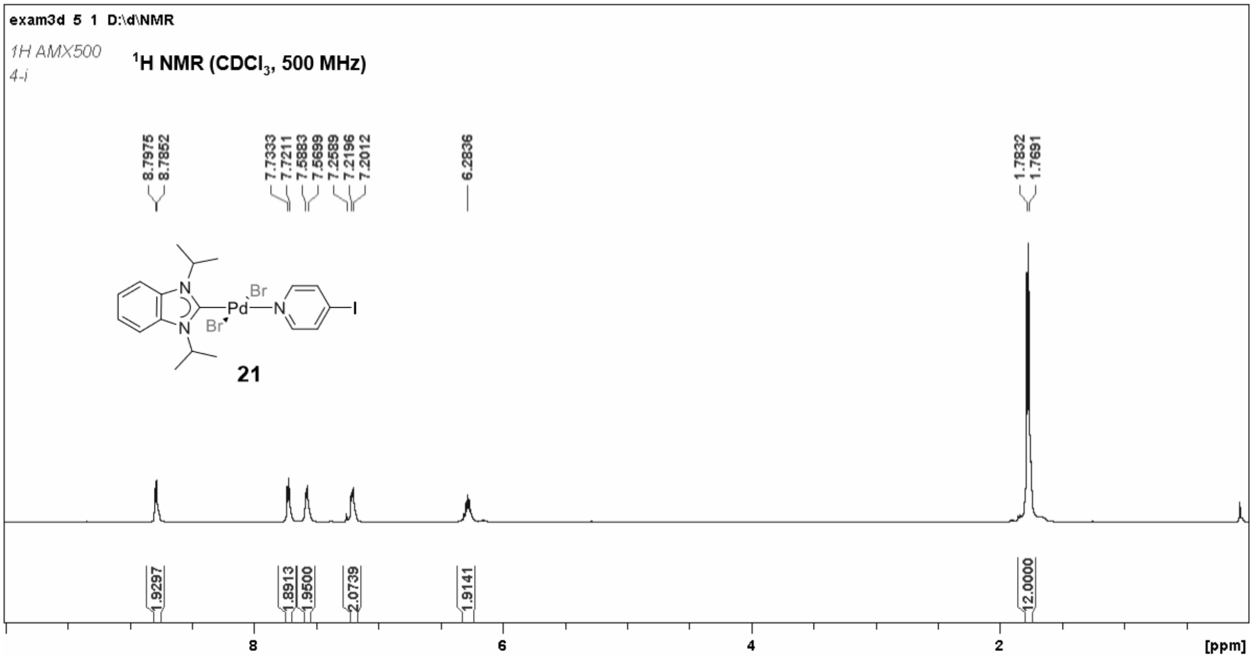
exam3d 5 1 D:\d\NMR
1H AMX500
4-i
^{1}H NMR (CDCl$_3$, 500 MHz)
8.7975
8.7852
7.7333
7.7211
7.5883
7.5699
7.2589
7.2196
7.2012
6.2836
1.7832
1.7691
Br
Pd
N
I
21
1.9297
1.8913
1.9500
2.0739
1.9141
12.0000
8
6
4
2
[ppm]

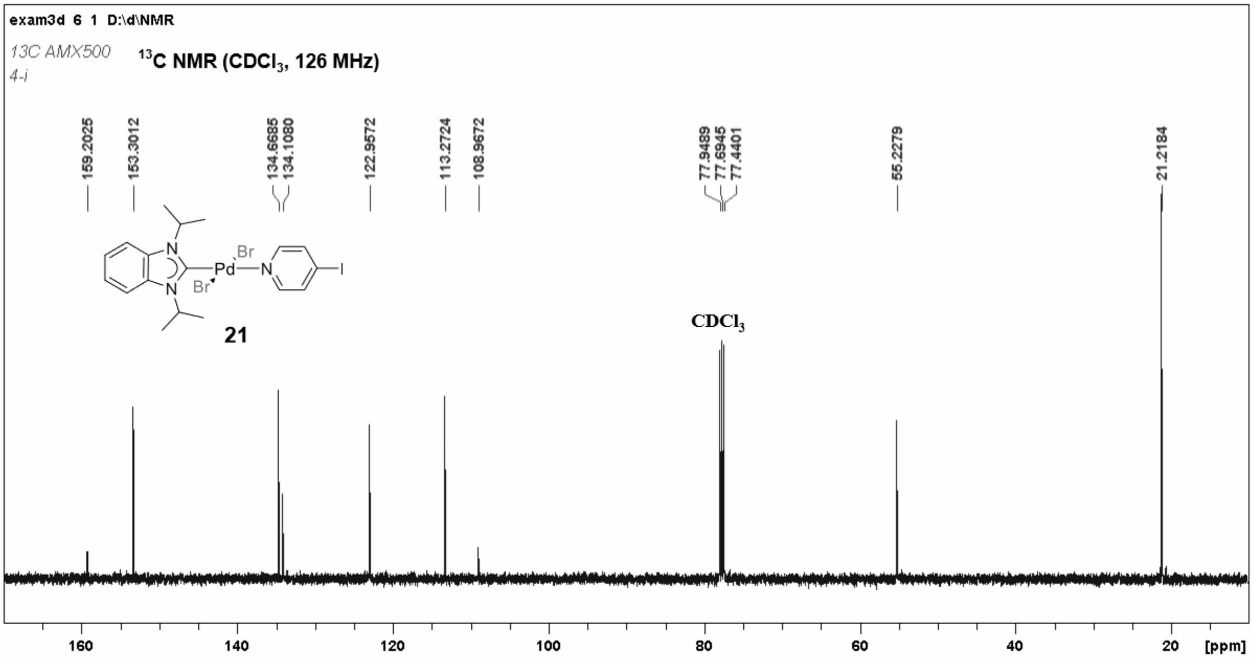
exam3d 6 1 D:\d\NMR
13C AMX500
4-i
^{13}C NMR (CDCl$_3$, 126 MHz)
159.2025
153.3012
134.6685
134.1080
122.9572
113.2724
108.9672
77.9489
77.6945
77.4401
55.2279
21.2184
21
CDCl$_3$
160
140
120
100
80
60
40
20
[ppm]

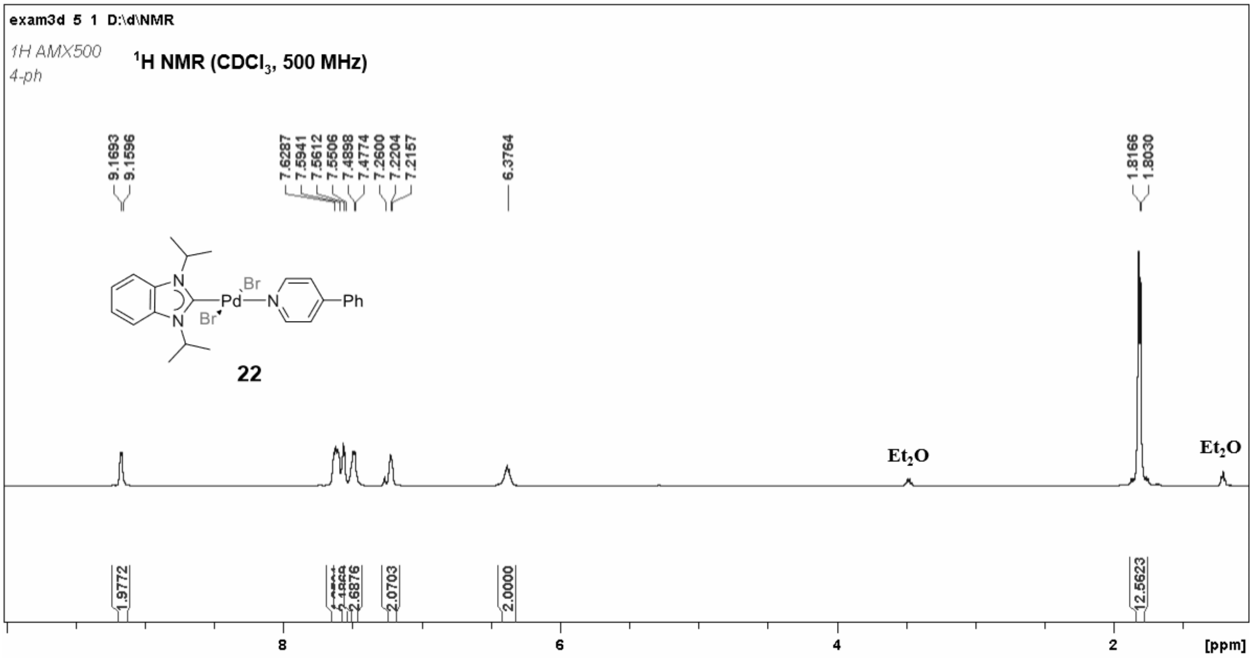
exam3d 5 1 D:\d\NMR
1H AMX500
4-ph
^{1}H NMR (CDCl$_3$, 500 MHz)
9.1693
9.1596
7.6287
7.5941
7.5612
7.5506
7.4898
7.4774
7.2600
7.2204
7.2157
6.3764
1.8166
1.8030
Ph
22
Et$_2$O
Et$_2$O
1.9772
2.6876
2.0703
2.0000
12.5623
8
6
4
2
[ppm]

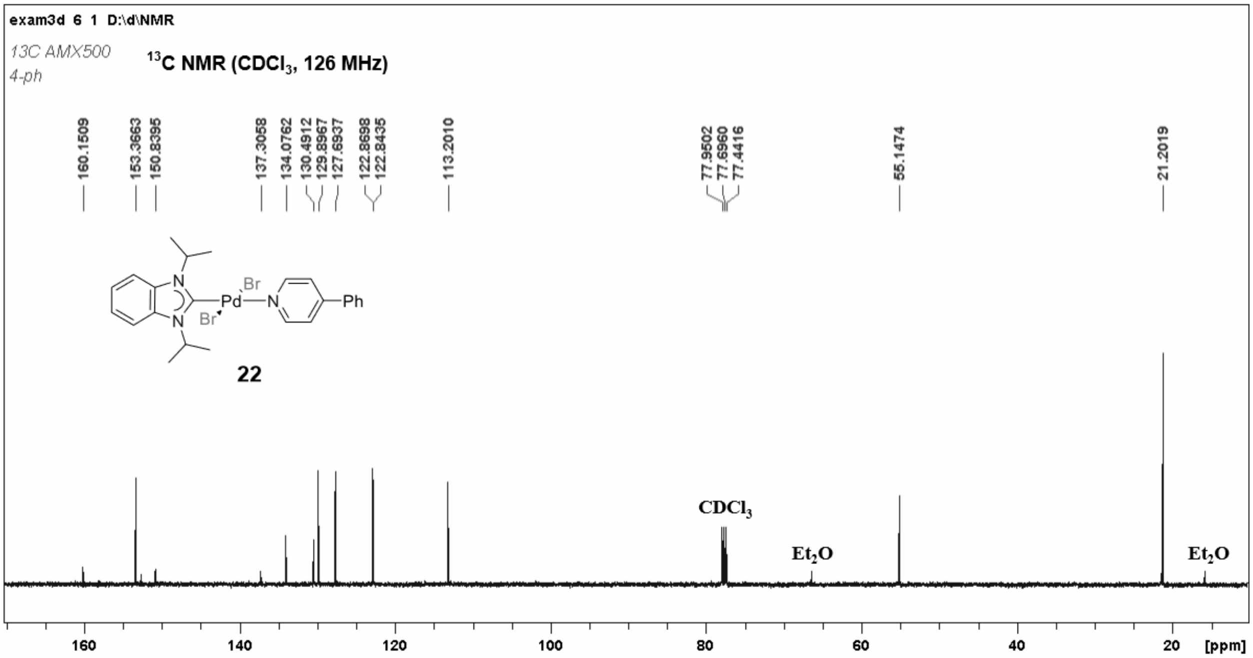
exam3d 6 1 D:\d\NMR
13C AMX500
4-ph
^{13}C NMR (CDCl$_3$, 126 MHz)
160.1509
153.3663
150.8395
137.3058
134.0762
130.4912
129.8967
127.6937
122.8698
122.8435
113.2010
77.9502
77.6960
77.4416
55.1474
21.2019
Br
Pd
N
Ph
22
CDCl$_3$
Et$_2$O
Et$_2$O
[ppm]

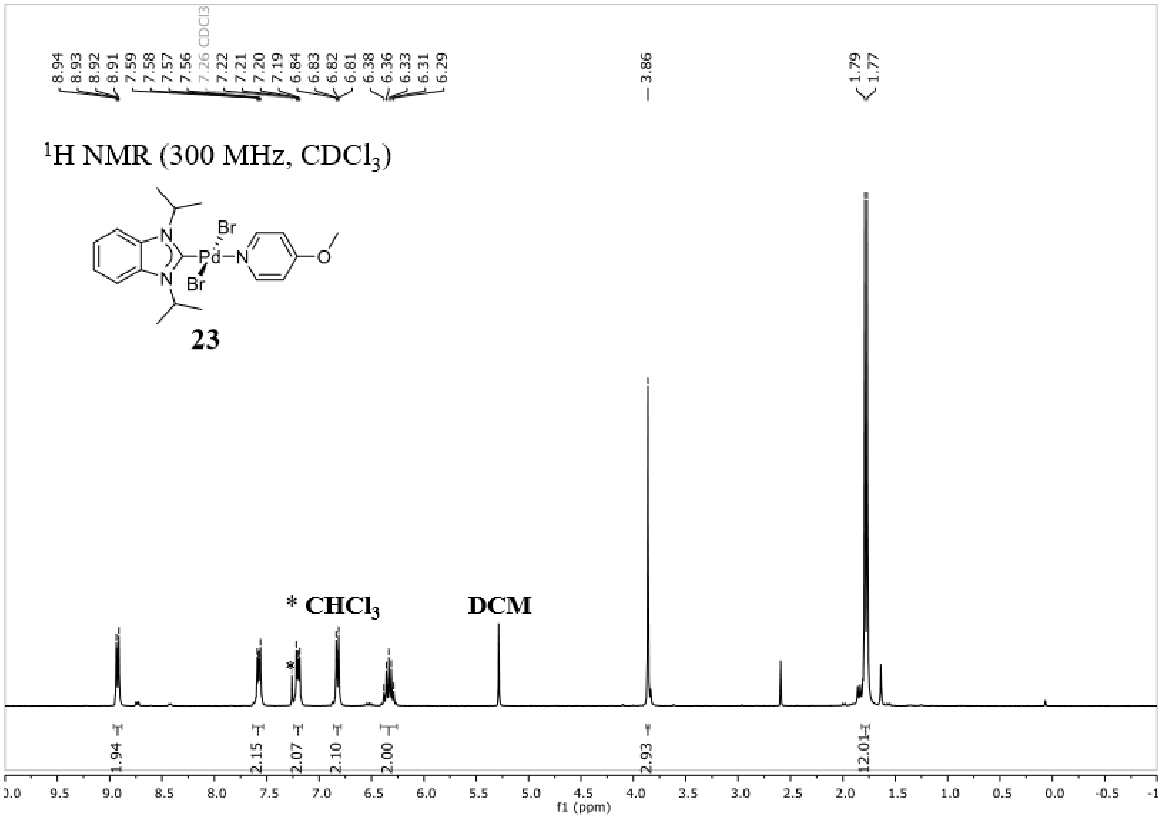
8.94
8.93
8.92
8.91
7.59
7.58
7.57
7.56
7.26 CDCl3
7.22
7.21
7.20
7.19
6.84
6.83
6.82
6.81
6.38
6.36
6.33
6.31
6.29
3.86
1.79
1.77
^{1}H NMR (300 MHz, CDCl$_3$)
23
* CHCl$_3$
DCM
1.94
2.15
2.07
2.10
2.00
2.93
12.01
f1 (ppm)

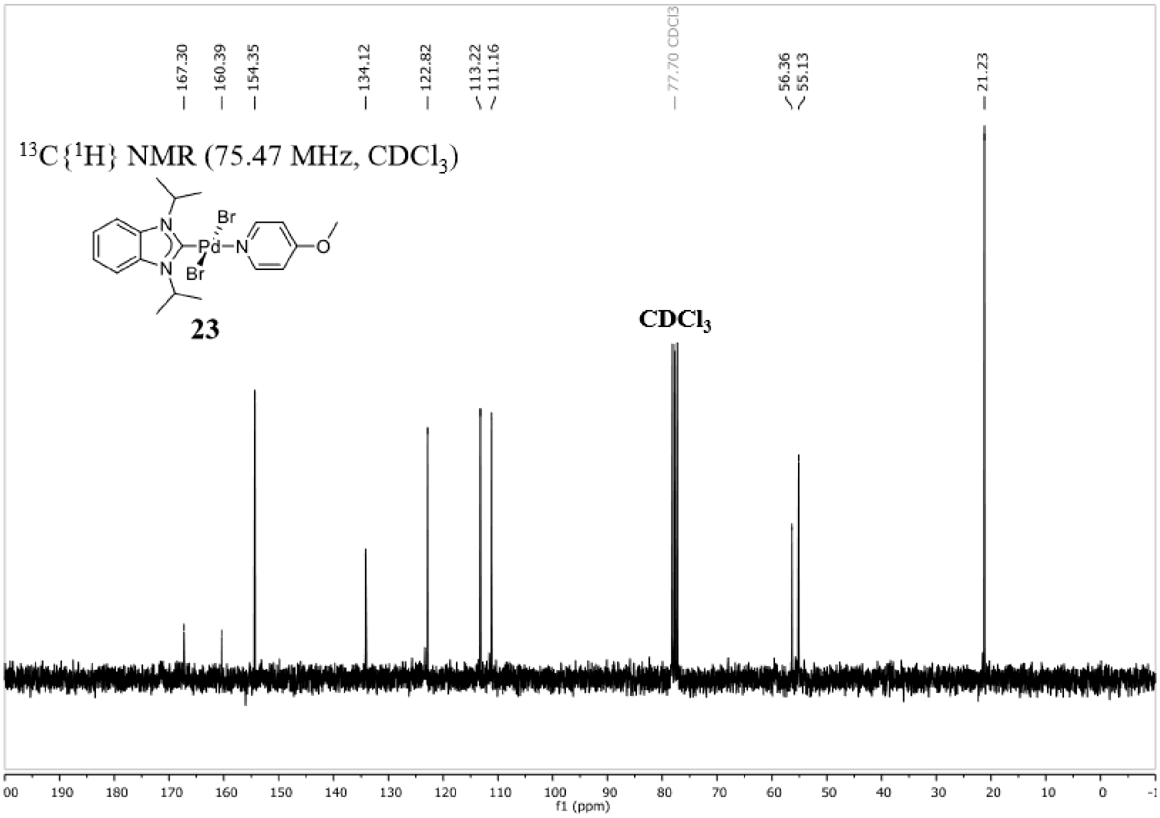
167.30
160.39
154.35
134.12
122.82
113.22
111.16
77.70 CDCl3
56.36
55.13
21.23
13C{1H} NMR (75.47 MHz, CDCl3)
Br
N
Pd
O
23
CDCl3
f1 (ppm)

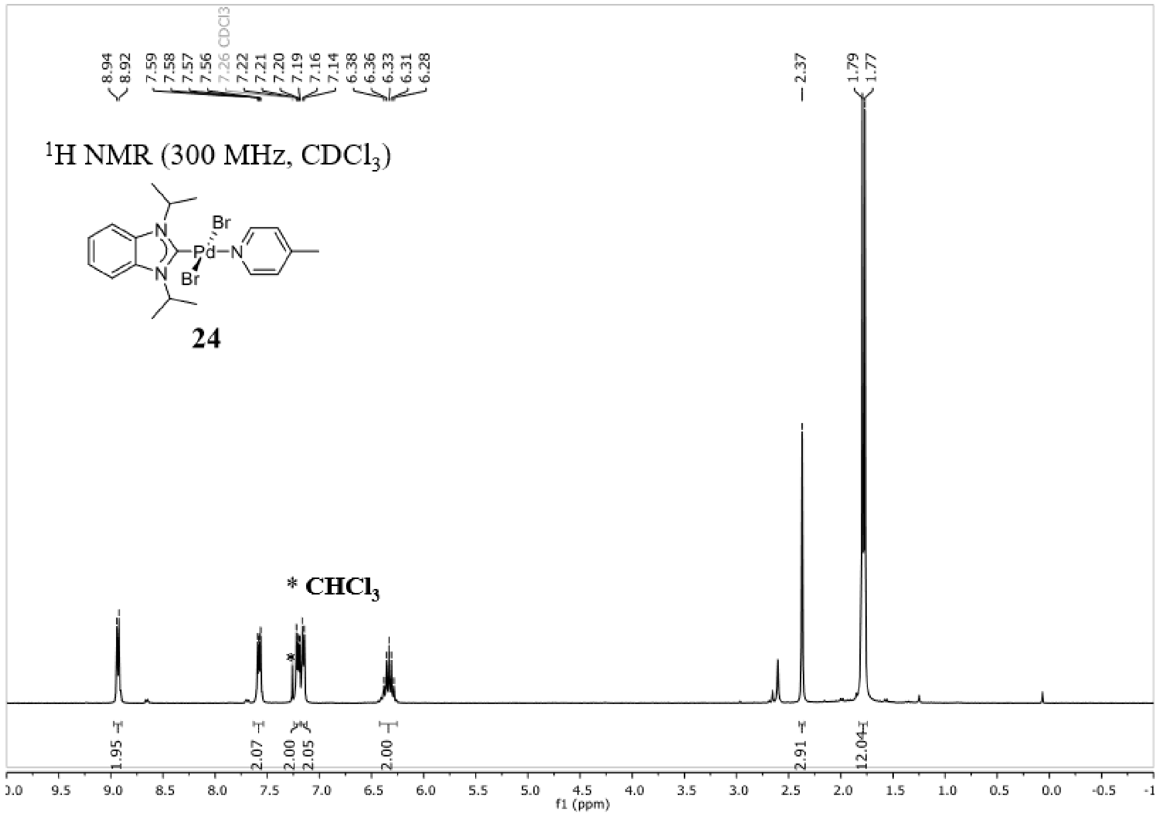
8.94
8.92
7.59
7.58
7.57
7.56
7.26 CDCl3
7.22
7.21
7.20
7.19
7.16
7.14
6.38
6.36
6.33
6.31
6.28
2.37
1.79
1.77
1H NMR (300 MHz, CDCl3)
Br
N
Pd
24
* CHCl3
1.95
2.07
2.00
2.05
2.00
2.91
12.04
f1 (ppm)

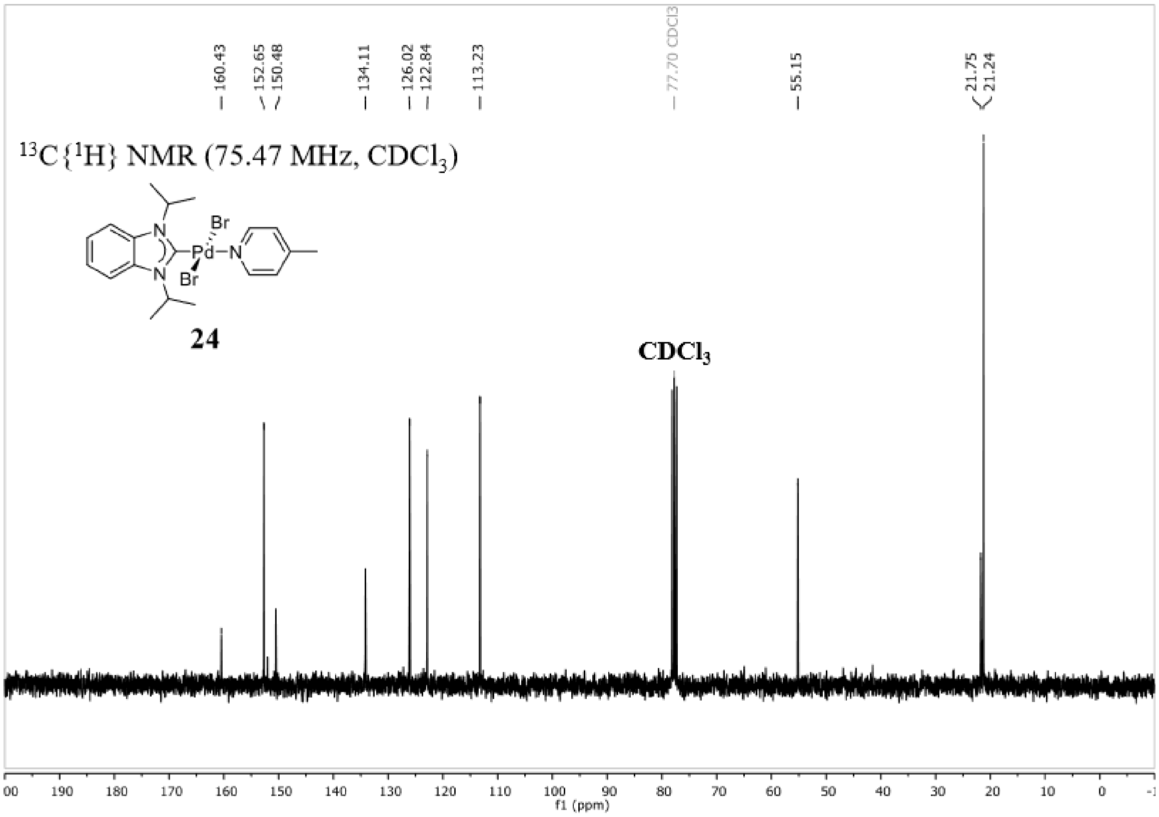
160.43
152.65
150.48
134.11
126.02
122.84
113.23
77.70 CDCl3
55.15
21.75
21.24
$^{13}C\{^1H\}$ NMR (75.47 MHz, $CDCl_3$)
Br
Pd
N
Br
24
$CDCl_3$
f1 (ppm)

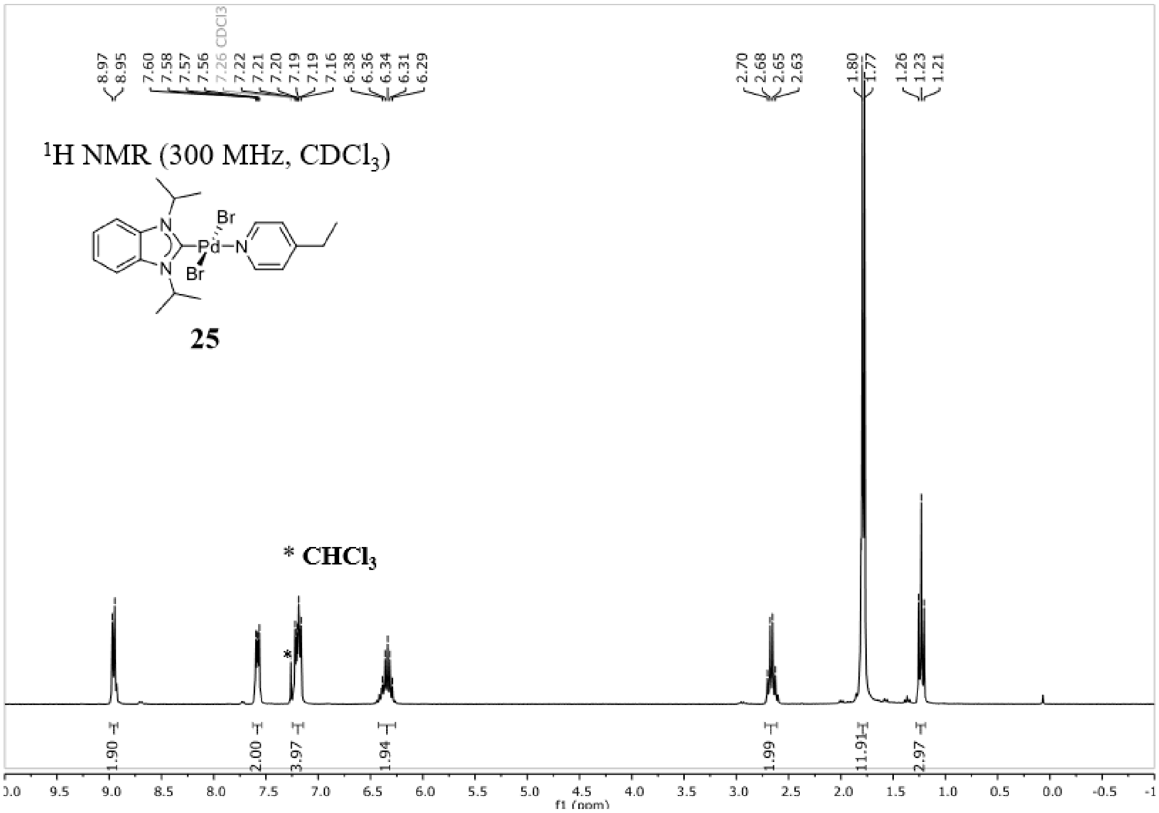
8.97
8.95
7.60
7.58
7.57
7.56
7.26 CDCl3
7.22
7.21
7.20
7.19
7.19
7.16
6.38
6.36
6.34
6.31
6.29
2.70
2.68
2.65
2.63
1.80
1.77
1.26
1.23
1.21
^1H NMR (300 MHz, $CDCl_3$)
Br
Pd
N
Br
25
* $CHCl_3$
1.90
2.00
3.97
1.94
1.99
11.91
2.97
f1 (ppm)

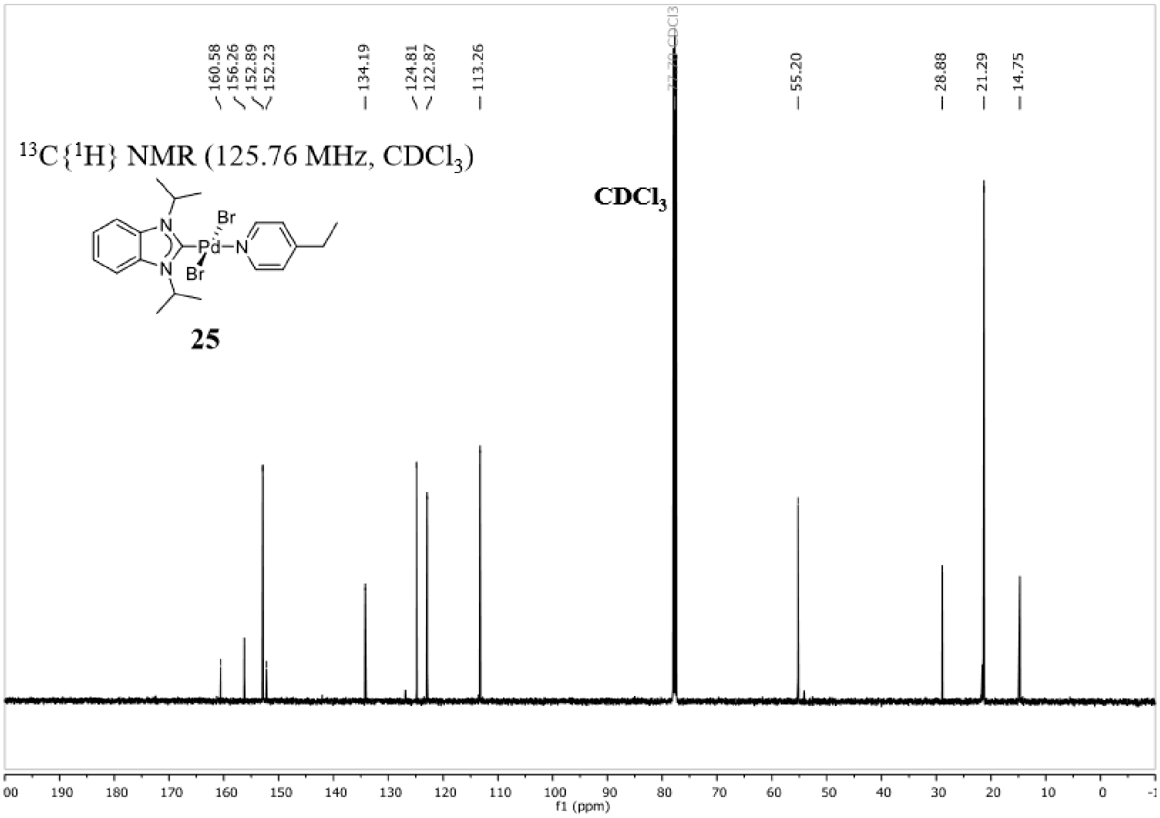
160.58
156.26
152.89
152.23
134.19
124.81
122.87
113.26
55.20
28.88
21.29
14.75
$^{13}C\{^{1}H\}$ NMR (125.76 MHz, $CDCl_3$)
Br
Pd
N
Br
25
$CDCl_3$
f1 (ppm)

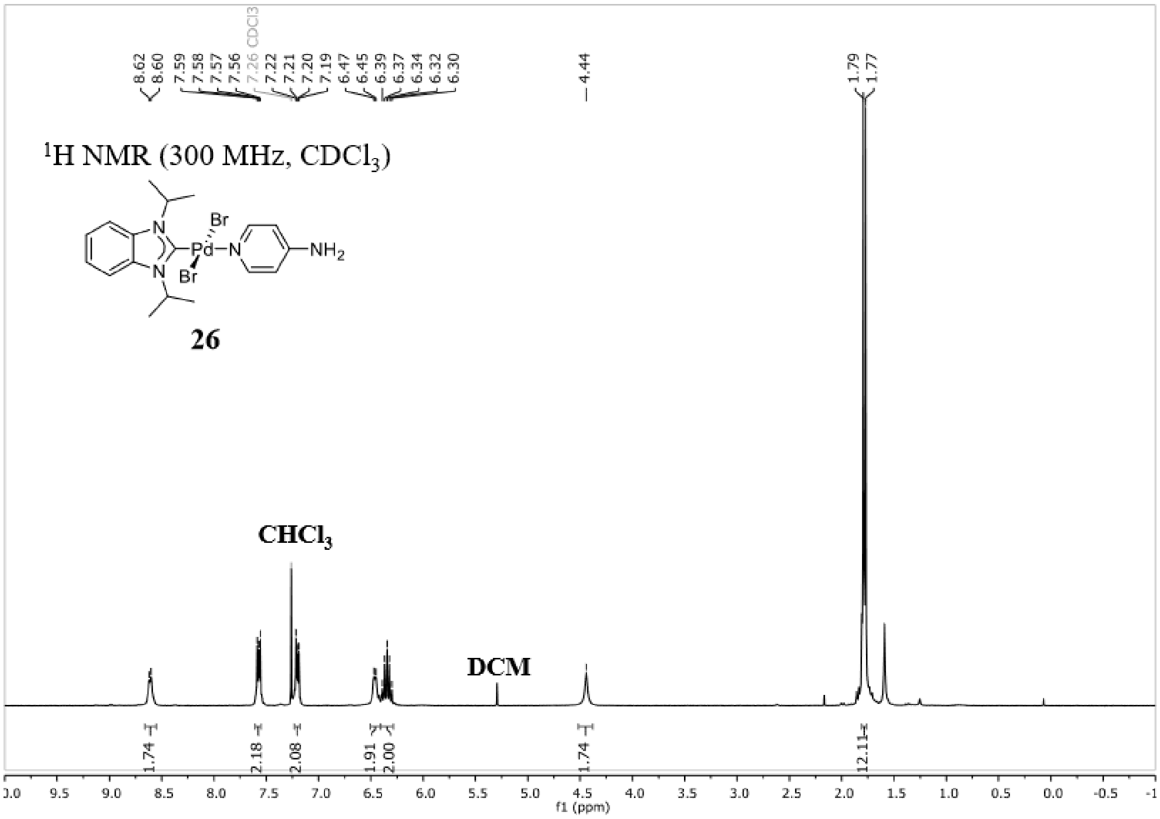
8.62
8.60
7.59
7.58
7.57
7.56
7.26 CDCl3
7.22
7.21
7.20
7.19
6.47
6.45
6.39
6.37
6.34
6.32
6.30
4.44
1.79
1.77
^{1}H NMR (300 MHz, $CDCl_3$)
Br
Pd
N
NH_2
Br
26
$CHCl_3$
DCM
1.74
2.18
2.08
1.91
2.00
1.74
12.11
f1 (ppm)

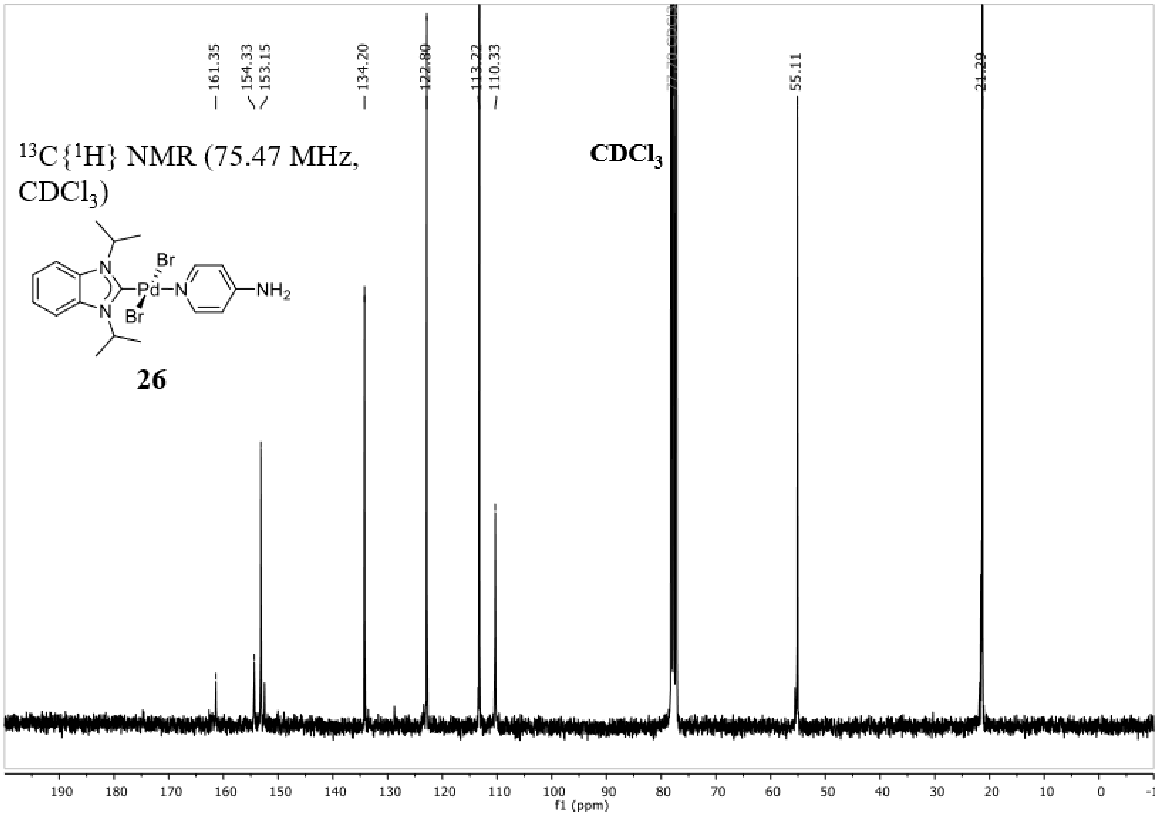
161.35
154.33
153.15
134.20
122.80
113.22
110.33
55.11
21.29
13C{1H} NMR (75.47 MHz, CDCl3)
Br
N
Pd
N
Br
NH2
26
CDCl3
190 180 170 160 150 140 130 120 110 100 90 80 70 60 50 40 30 20 10 0
f1 (ppm)

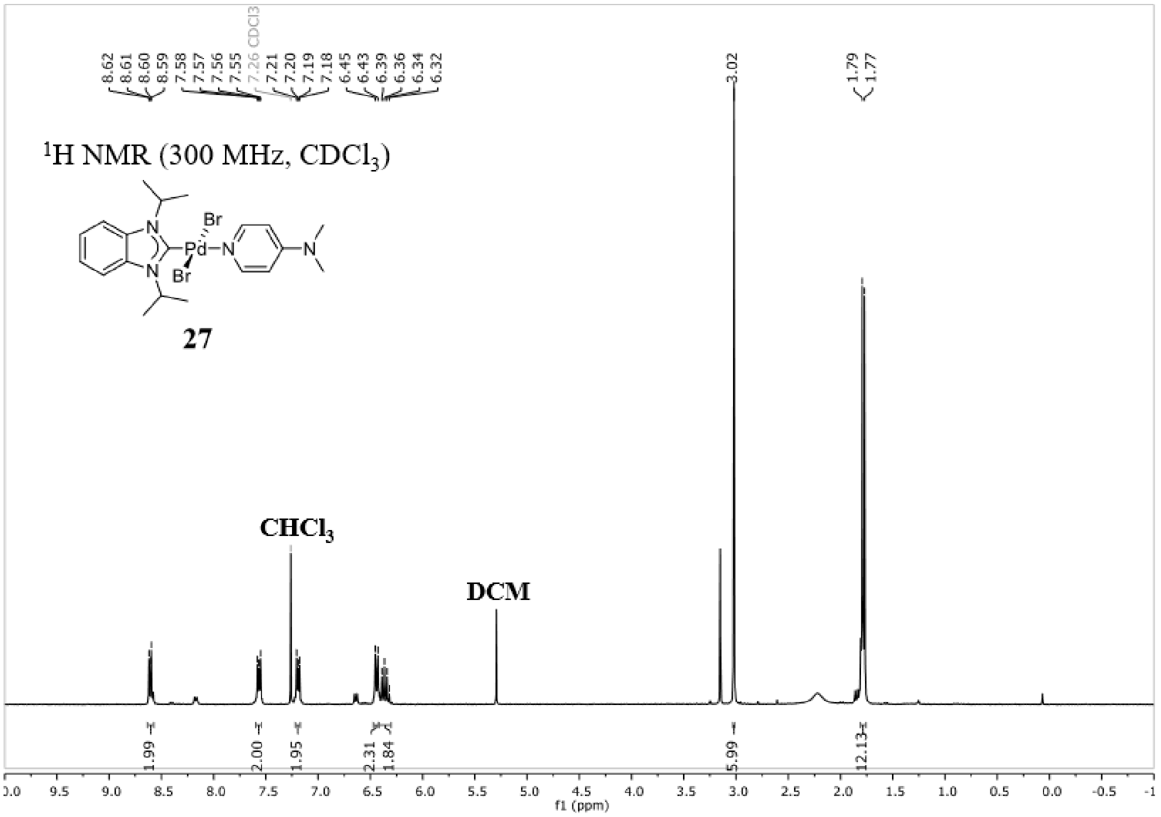
8.62
8.61
8.60
8.59
7.58
7.57
7.56
7.55
7.26 CDCl3
7.21
7.20
7.19
7.18
6.45
6.43
6.39
6.36
6.34
6.32
3.02
1.79
1.77
1H NMR (300 MHz, CDCl3)
Br
N
Pd
N
Br
27
CHCl3
DCM
1.99
2.00
1.95
2.31
1.84
5.99
12.13
9.5 9.0 8.5 8.0 7.5 7.0 6.5 6.0 5.5 5.0 4.5 4.0 3.5 3.0 2.5 2.0 1.5 1.0 0.5 0.0 -0.5
f1 (ppm)

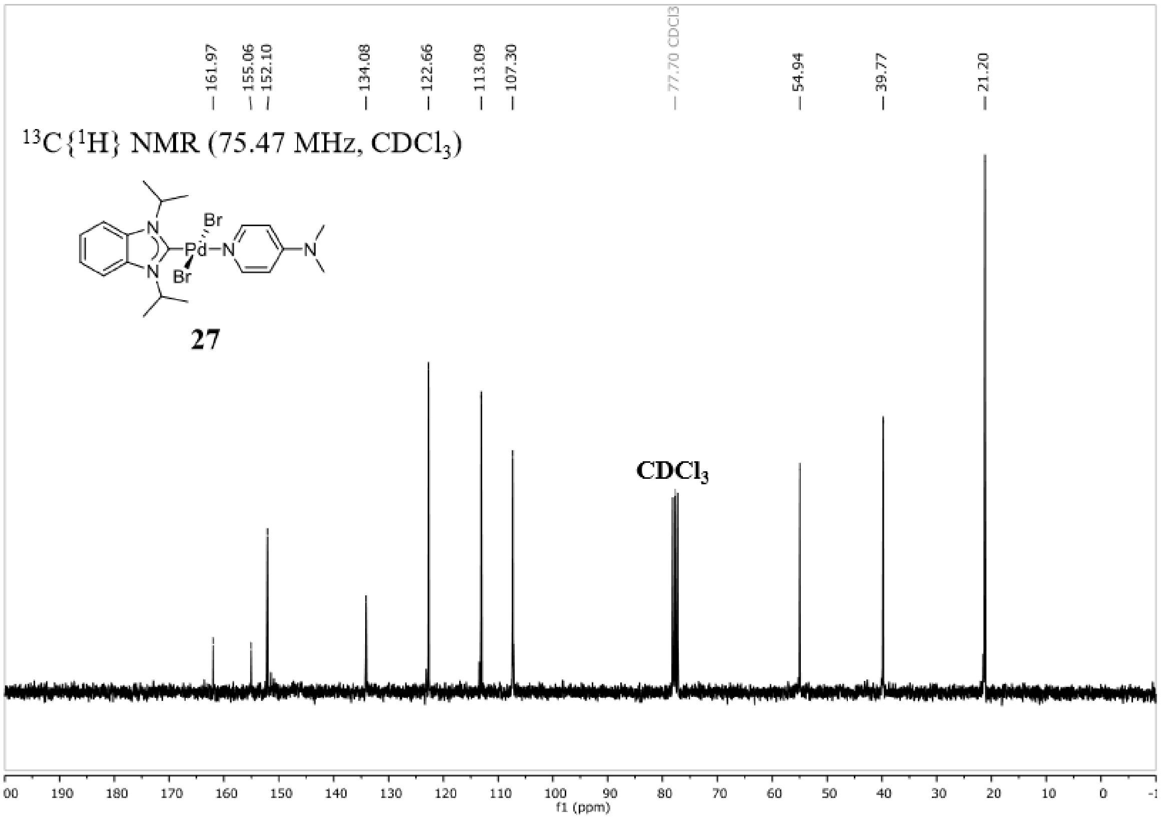
161.97
155.06
152.10
134.08
122.66
113.09
107.30
77.70 CDCl3
54.94
39.77
21.20
$^{13}C\{^1H\}$ NMR (75.47 MHz, $CDCl_3$)
Br
N
Pd
Br
N
27
$CDCl_3$
f1 (ppm)

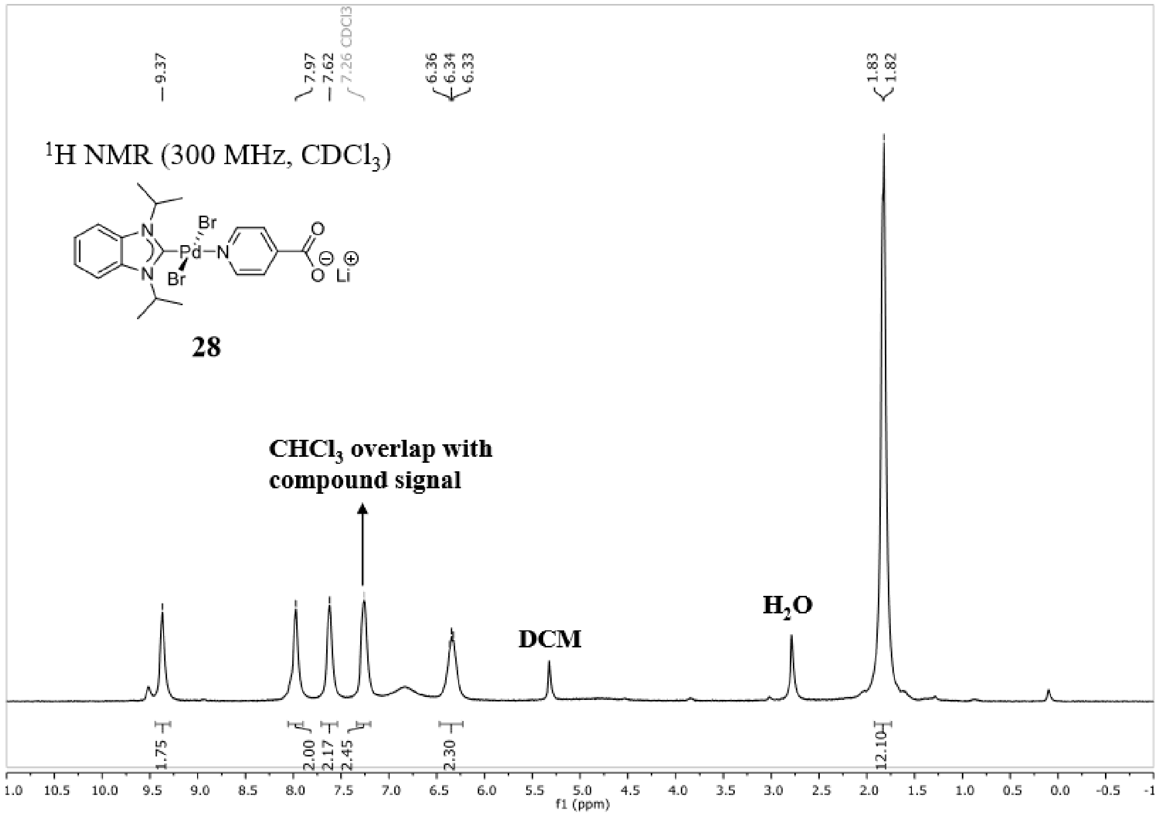
9.37
7.97
7.62
7.26 CDCl3
6.36
6.34
6.33
1.83
1.82
^1H NMR (300 MHz, $CDCl_3$)
Br
Pd
N
O
Li
28
$CHCl_3$ overlap with compound signal
DCM
H_2O
1.75
2.00
2.17
2.45
2.30
12.10
f1 (ppm)

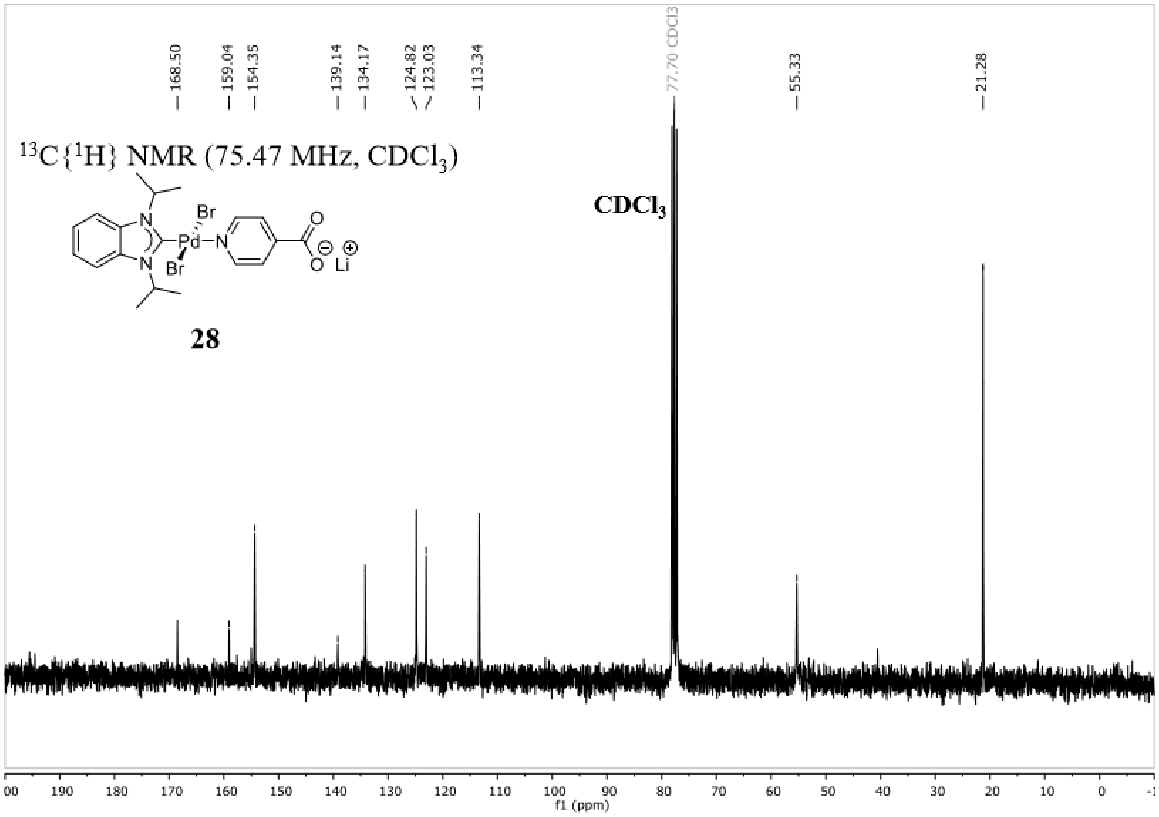
168.50
159.04
154.35
139.14
134.17
124.82
123.03
113.34
77.70 CDCl3
55.33
21.28
$^{13}C\{^1H\}$ NMR (75.47 MHz, $CDCl_3$)
N
Br
Pd
N
O
Br
O
Li
28
$CDCl_3$
f1 (ppm)

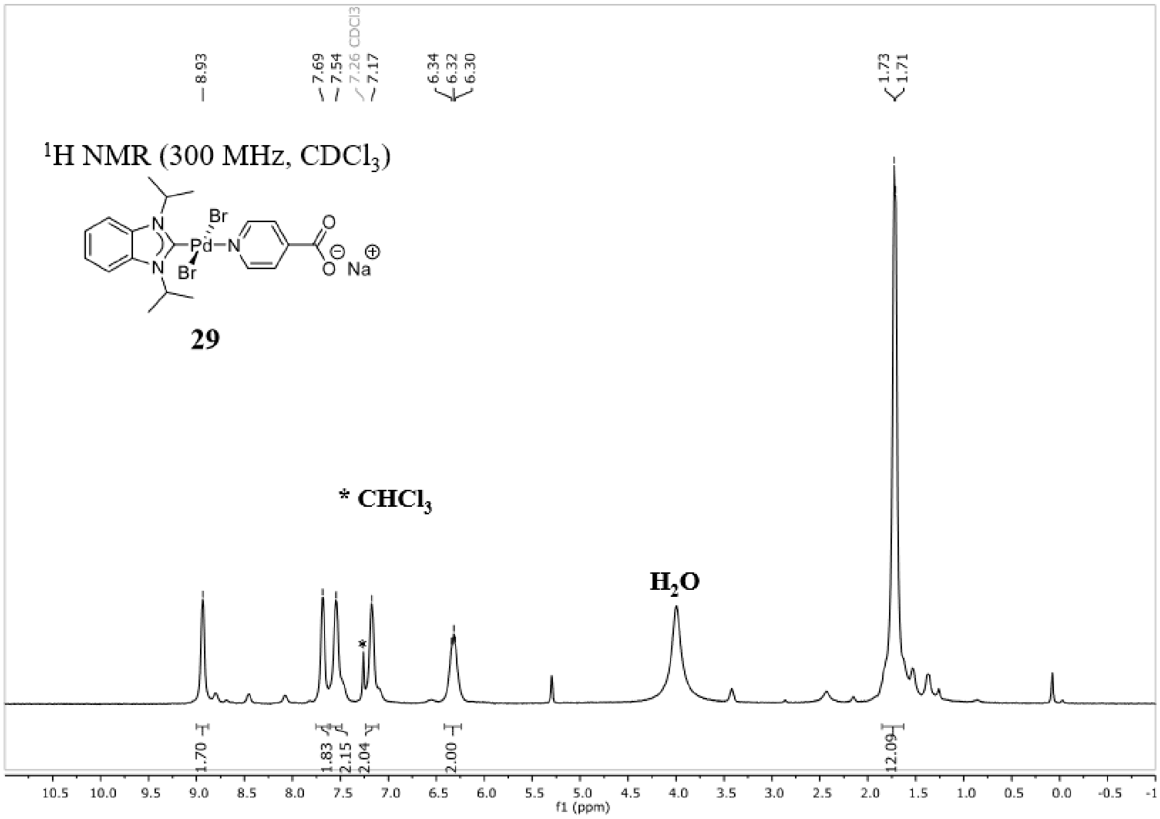
8.93
7.69
7.54
7.26 CDCl3
7.17
6.34
6.32
6.30
1.73
1.71
^1H NMR (300 MHz, $CDCl_3$)
N
Br
Pd
N
O
Br
O
Na
29
* $CHCl_3$
H_2O
1.70
1.83
2.15
2.04
2.00
12.09
f1 (ppm)

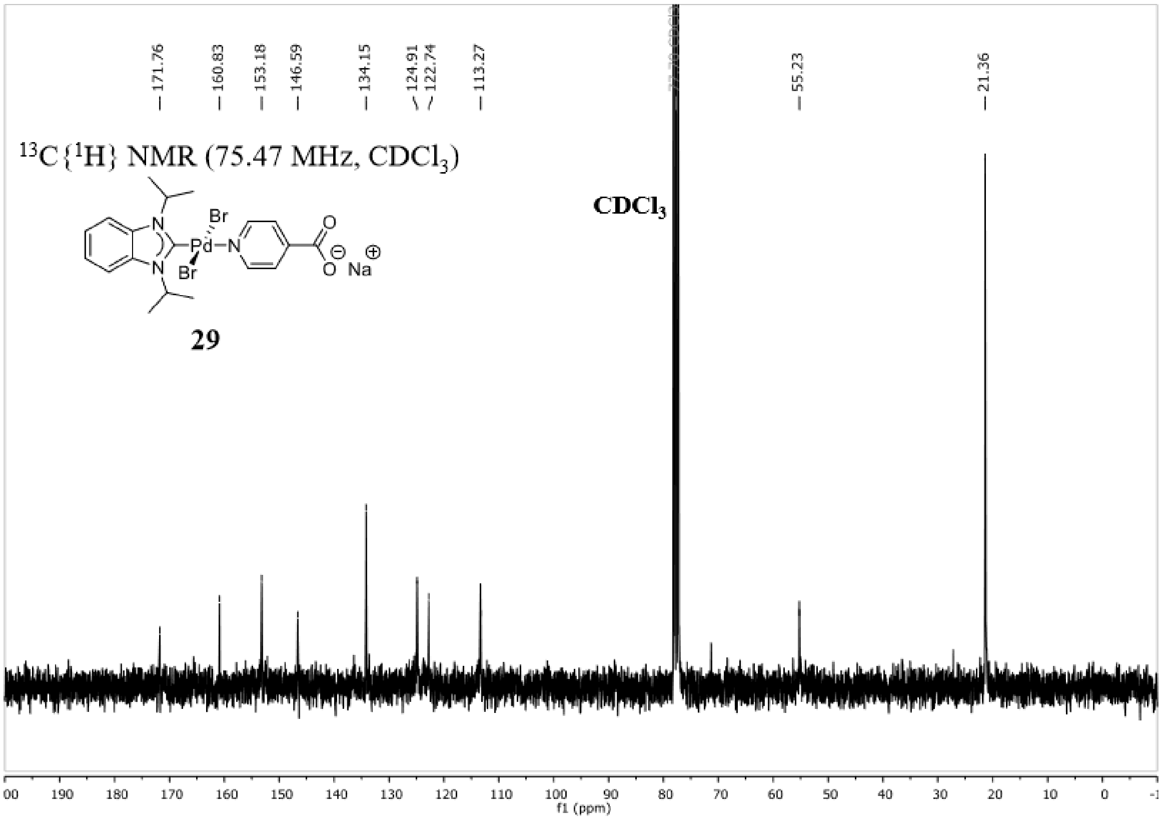
171.76
160.83
153.18
146.59
134.15
124.91
122.74
113.27
55.23
21.36
$^{13}C\{^1H\}$ NMR (75.47 MHz, $CDCl_3$)
Br
Pd
N
Br
O
Na
CDCl3
f1 (ppm)

29

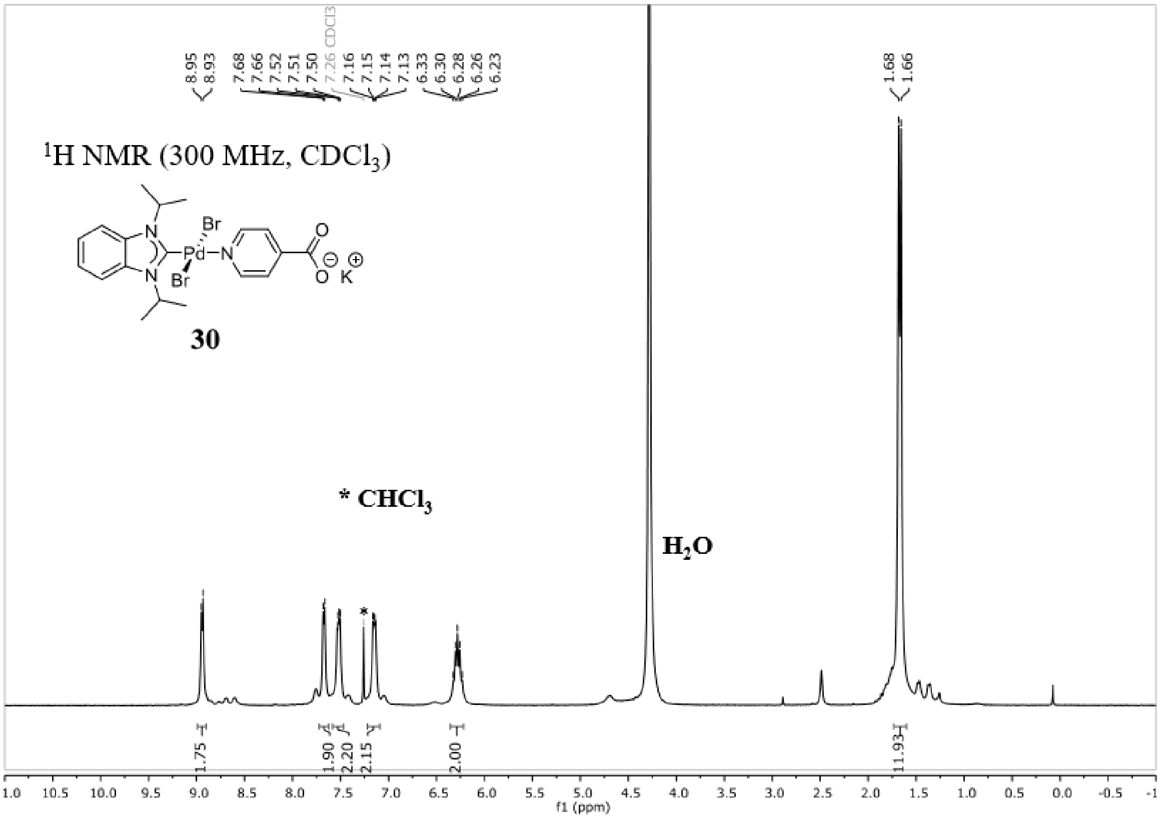
8.95
8.93
7.68
7.66
7.52
7.51
7.50
7.26 CDCl3
7.16
7.15
7.14
7.13
6.33
6.30
6.28
6.26
6.23
1.68
1.66
^1H NMR (300 MHz, $CDCl_3$)
Br
Pd
N
Br
O
K
* CHCl3
H2O
1.75
1.90
2.20
2.15
2.00
11.93
f1 (ppm)

30

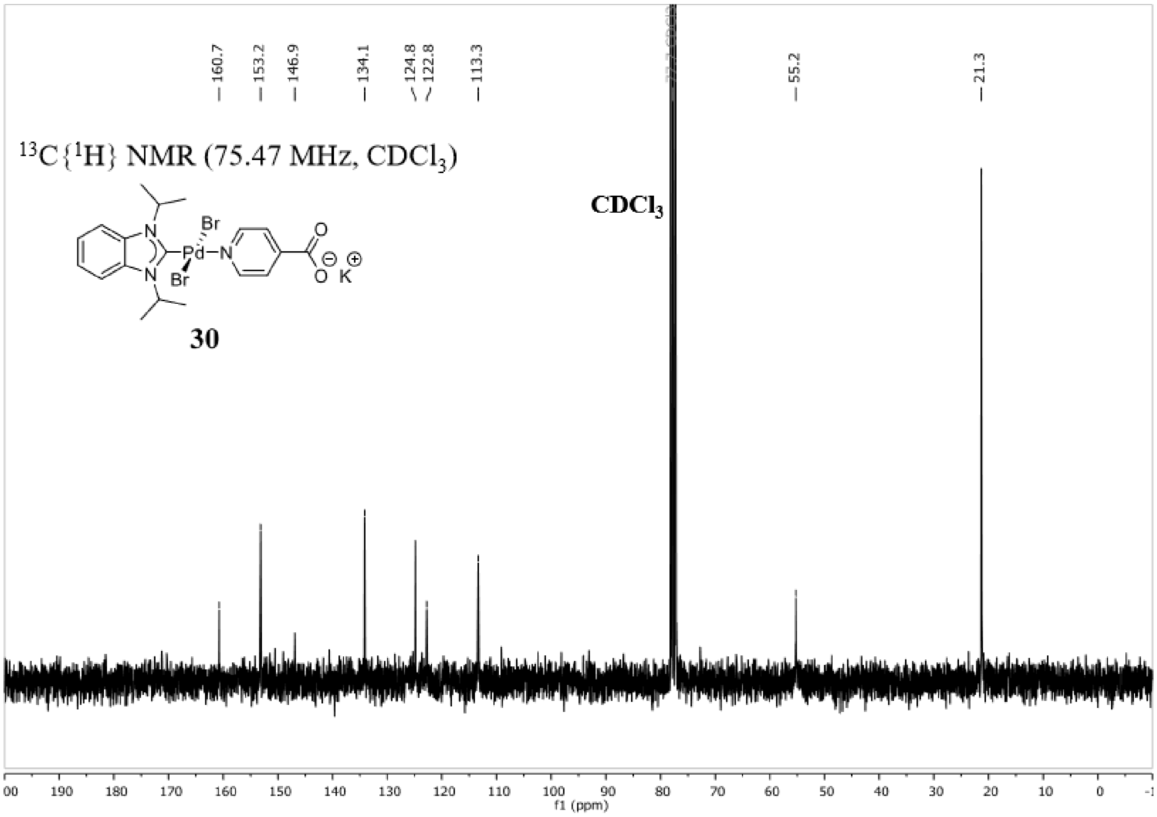

160.7
153.2
146.9
134.1
124.8
122.8
113.3
55.2
21.3
$^{13}C\{^1H\}$ NMR (75.47 MHz, $CDCl_3$)
$CDCl_3$
30
f1 (ppm)

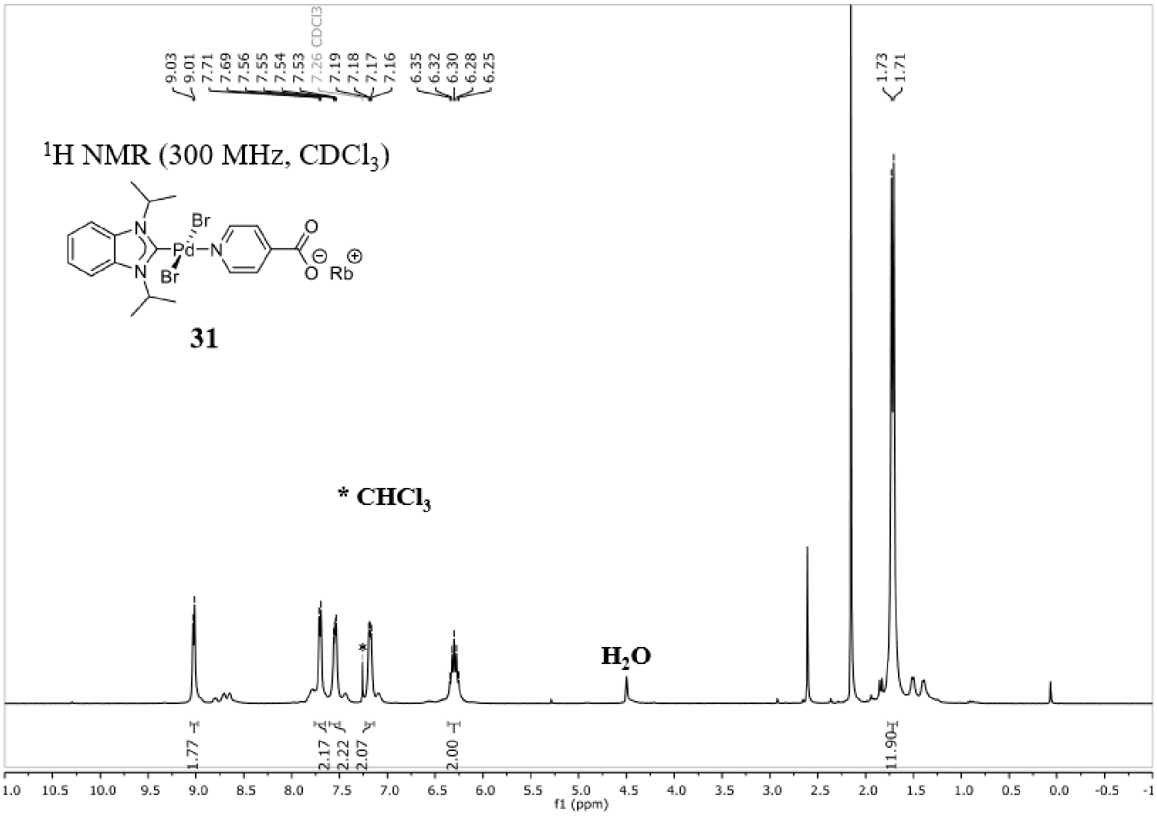

9.03
9.01
7.71
7.69
7.56
7.55
7.54
7.53
7.26 CDCl3
7.19
7.18
7.17
7.16
6.35
6.32
6.30
6.28
6.25
1.73
1.71
^1H NMR (300 MHz, $CDCl_3$)
31
* $CHCl_3$
H_2O
1.77
2.17
2.22
2.07
2.00
11.90
f1 (ppm)

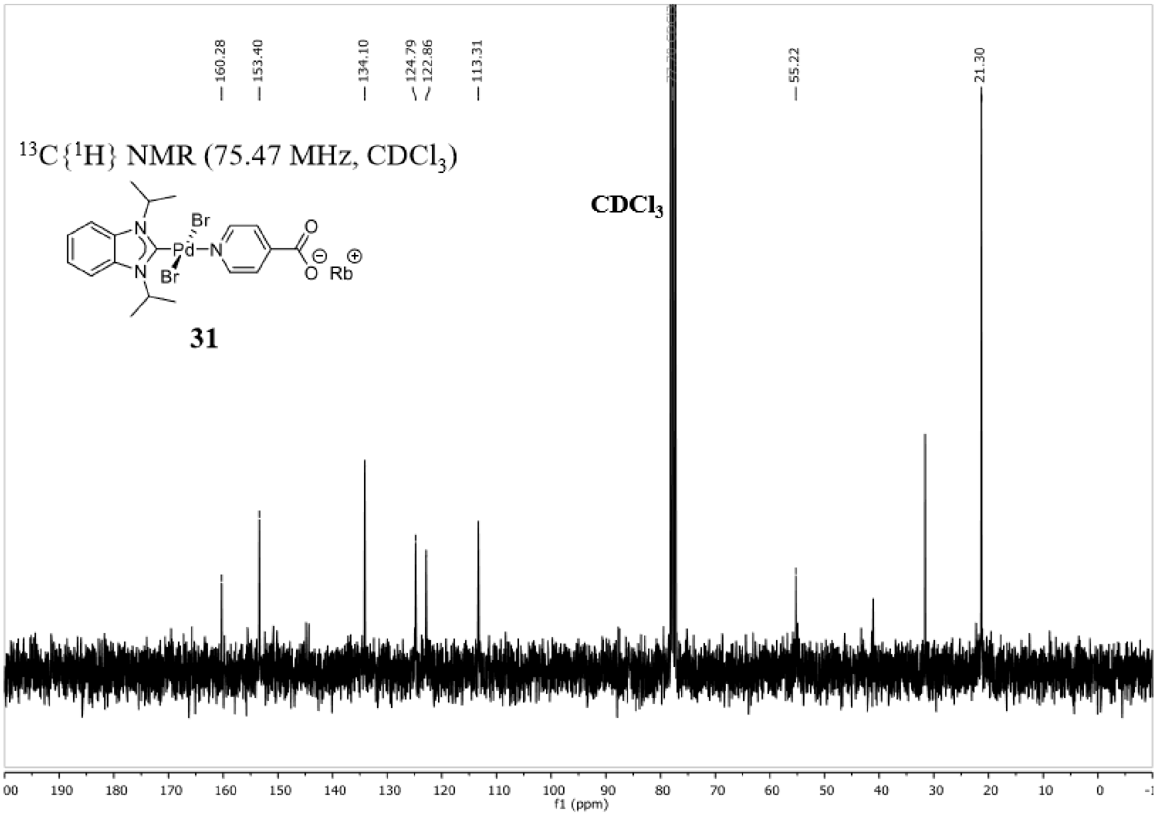

160.28
153.40
134.10
124.79
122.86
113.31
55.22
21.30
^{13}C{^{1}H} NMR (75.47 MHz, CDCl$_3$)
Br
Pd
N
Br
O
Rb
31
CDCl$_3$
f1 (ppm)

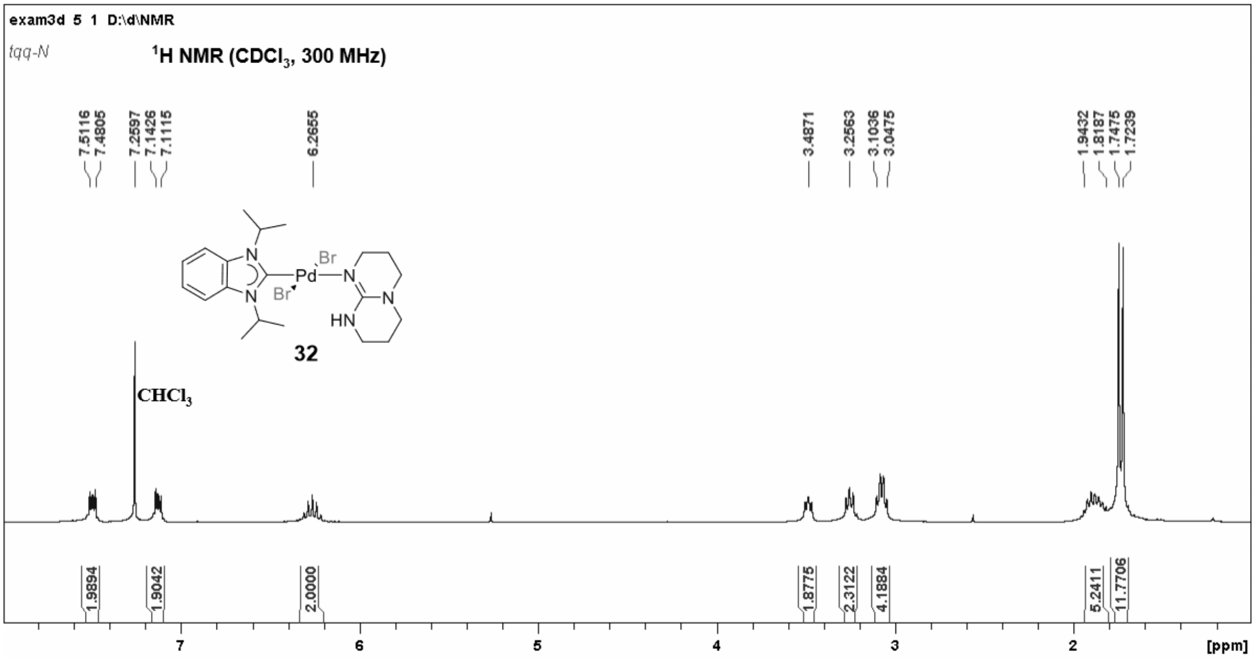

exam3d 5 1 D:\d\NMR
tqq-N
^{1}H NMR (CDCl$_3$, 300 MHz)
7.5116
7.4805
7.2597
7.1426
7.1115
6.2655
3.4871
3.2563
3.1036
3.0475
1.9432
1.8187
1.7475
1.7239
Br
Pd
HN
32
CHCl$_3$
1.9894
1.9042
2.0000
1.8775
2.3122
4.1884
5.2411
11.7706
[ppm]

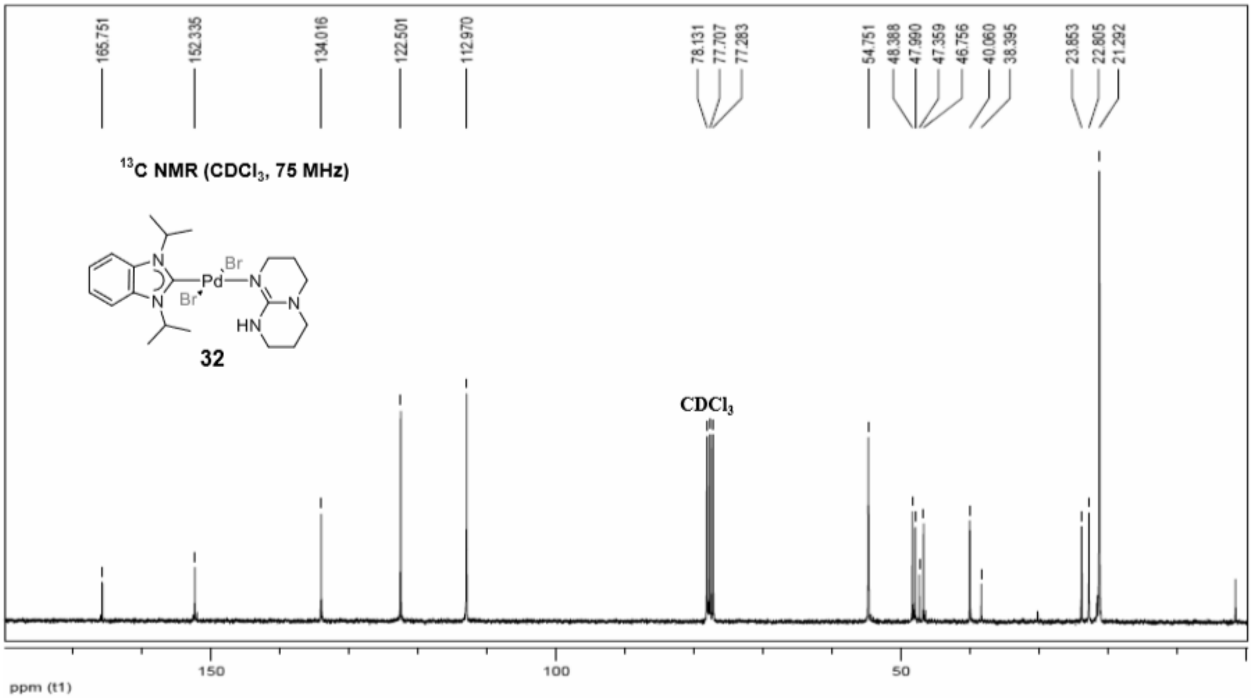
165.751
152.335
134.016
122.501
112.970
78.131
77.707
77.283
54.751
48.388
47.990
47.359
46.756
40.060
38.395
23.853
22.805
21.292
13C NMR (CDCl3, 75 MHz)
Br
Pd
N
Br
HN
32
CDCl3
150
100
50
ppm (t1)

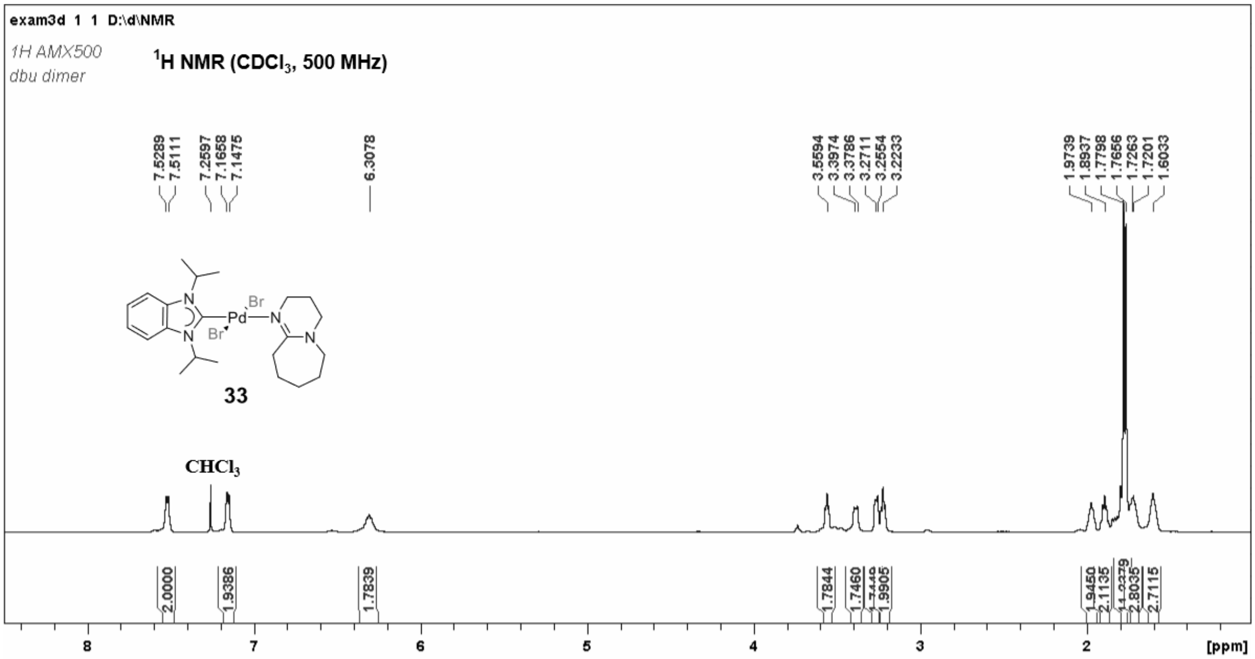
exam3d 1 1 D:\d\NMR
1H AMX500
dbu dimer
1H NMR (CDCl3, 500 MHz)
7.5289
7.5111
7.2597
7.1658
7.1475
6.3078
3.5594
3.3974
3.3786
3.2711
3.2554
3.2233
1.9739
1.8937
1.7798
1.7656
1.7263
1.7201
1.6033
Pd
Br
Br
33
CHCl3
2.0000
1.9386
1.7839
1.7844
1.7460
1.9905
1.9450
2.1135
2.8035
2.7115
8
7
6
5
4
3
2
[ppm]

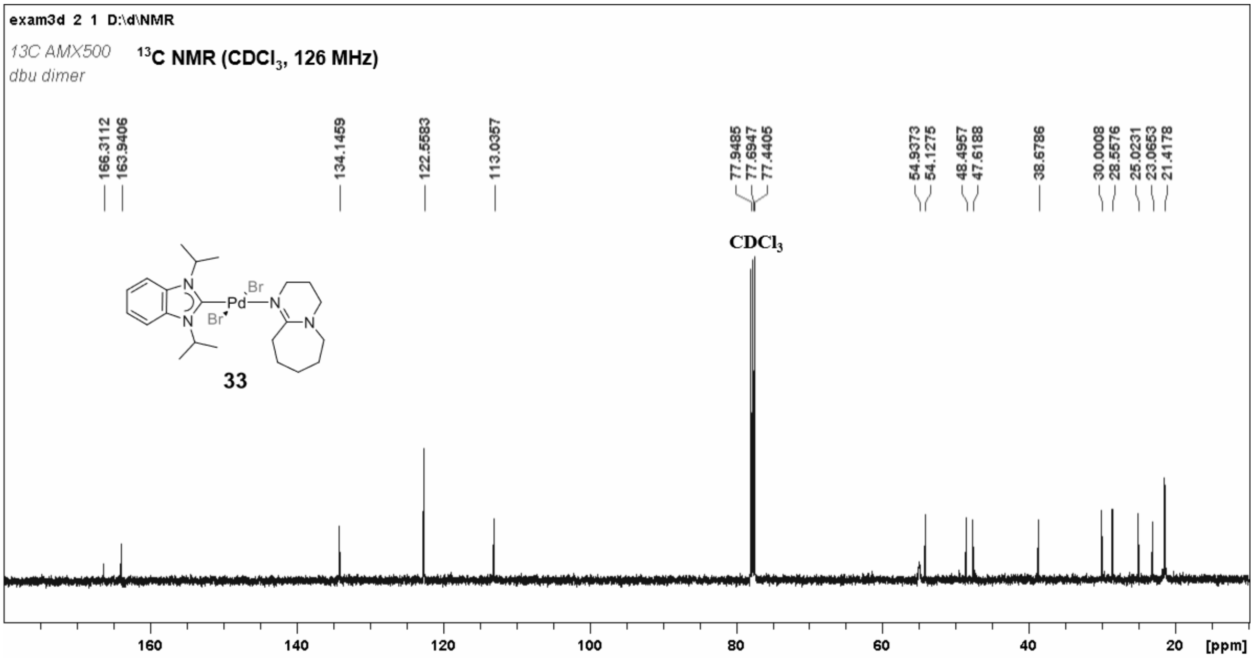
exam3d 2 1 D:\d\NMR
13C AMX500
dbu dimer
13C NMR (CDCl3, 126 MHz)
166.3112
163.9406
134.1459
122.5583
113.0357
77.9485
77.6947
77.4405
54.9373
54.1275
48.4957
47.6188
38.6786
30.0008
28.5576
25.0231
23.0653
21.4178
CDCl3
33
160
140
120
100
80
60
40
20
[ppm]

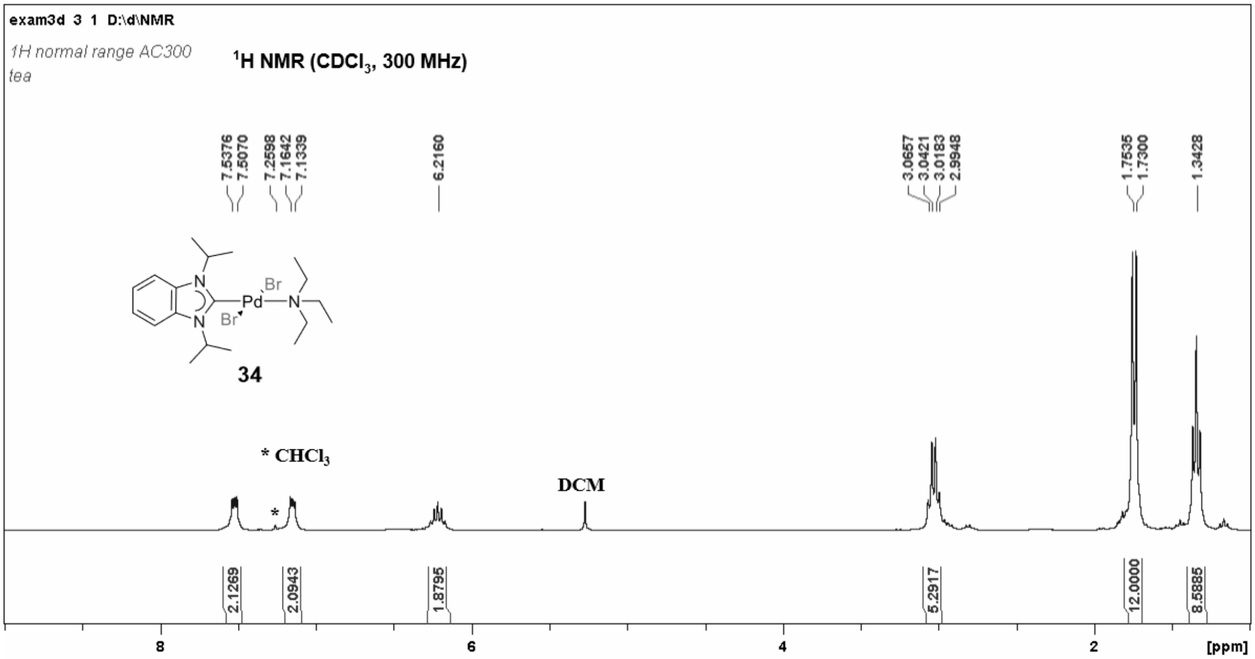
exam3d 3 1 D:\d\NMR
1H normal range AC300
tea
1H NMR (CDCl3, 300 MHz)
7.5376
7.5070
7.2598
7.1642
7.1339
6.2160
3.0657
3.0421
3.0183
2.9948
1.7535
1.7300
1.3428
34
* CHCl3
DCM
2.1269
2.0943
1.8795
5.2917
12.0000
8.5885
8
6
4
2
[ppm]

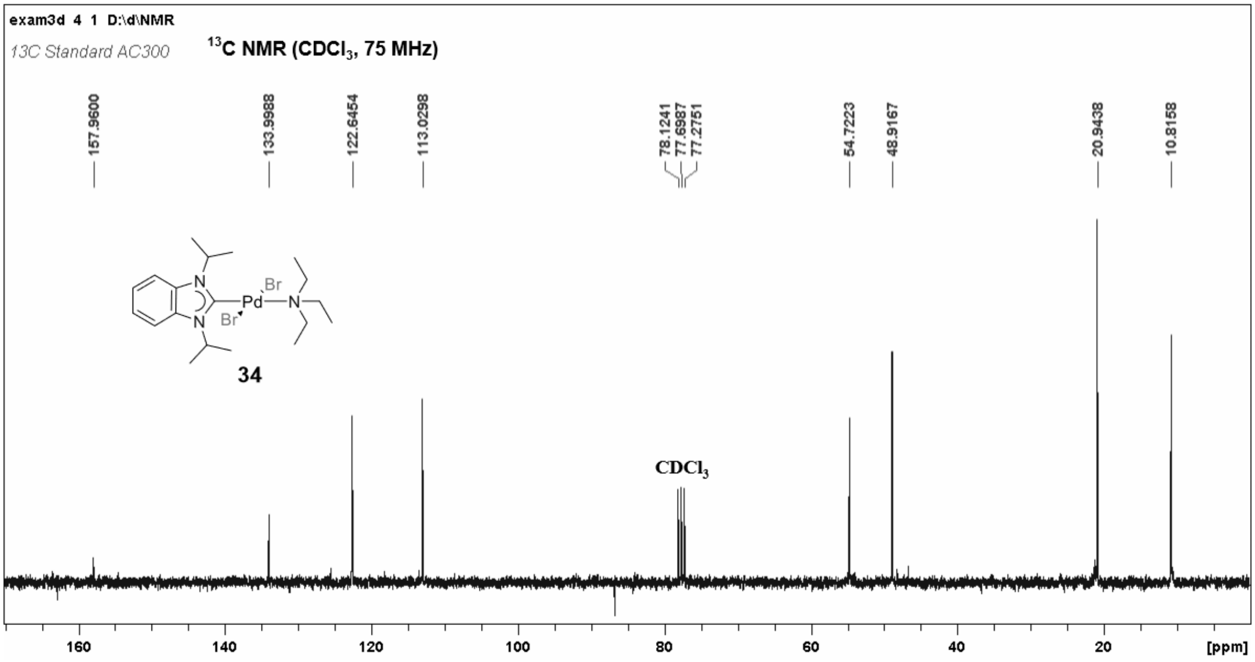
exam3d 4 1 D:\d\NMR
13C Standard AC300
^{13}C NMR (CDCl$_3$, 75 MHz)
157.9600
133.9988
122.6454
113.0298
78.1241
77.6987
77.2751
54.7223
48.9167
20.9438
10.8158
34
CDCl$_3$
160
140
120
100
80
60
40
20
[ppm]

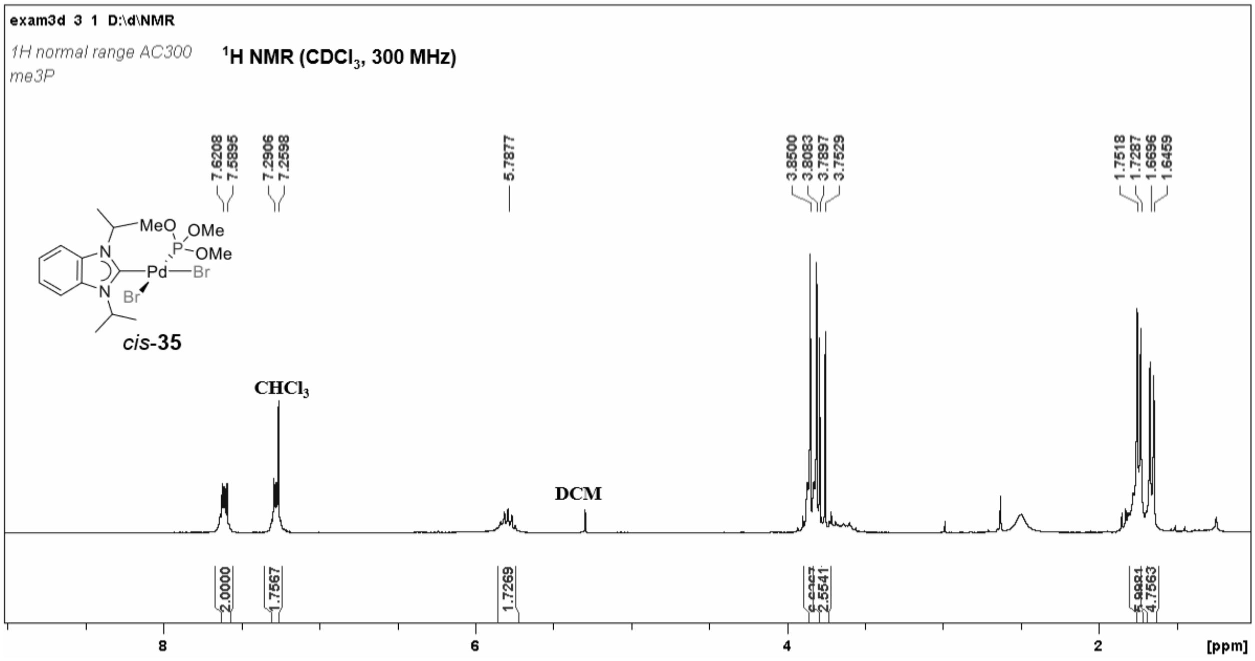
exam3d 3 1 D:\d\NMR
1H normal range AC300
me3P
^{1}H NMR (CDCl$_3$, 300 MHz)
7.6208
7.5895
7.2906
7.2598
5.7877
3.8500
3.8083
3.7897
3.7529
1.7518
1.7287
1.6696
1.6459
cis-35
CHCl$_3$
DCM
2.0000
1.7567
1.7269
2.5541
4.7563
8
6
4
2
[ppm]

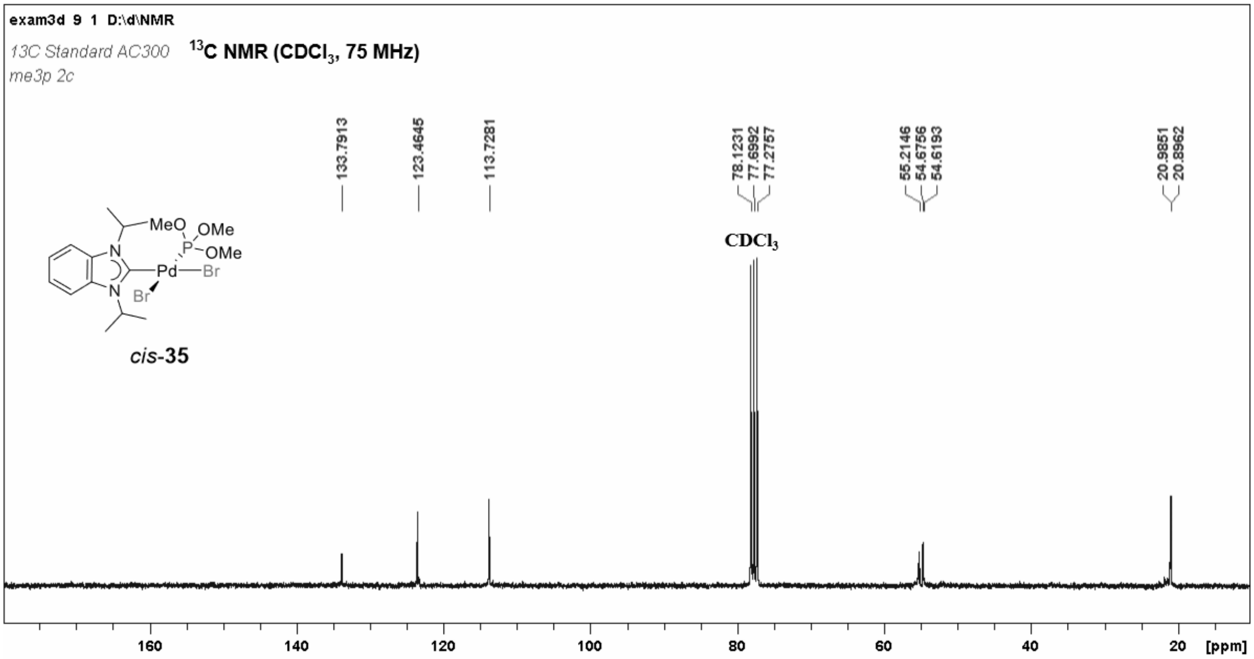
exam3d 9 1 D:\d\NMR
13C Standard AC300
me3p 2c
^{13}C NMR (CDCl$_3$, 75 MHz)
133.7913
123.4645
113.7281
78.1231
77.6992
77.2757
55.2146
54.6756
54.6193
20.9851
20.8962
CDCl$_3$
MeO
OMe
OMe
N
N
Pd
Br
Br
P
cis-35
160
140
120
100
80
60
40
20
[ppm]

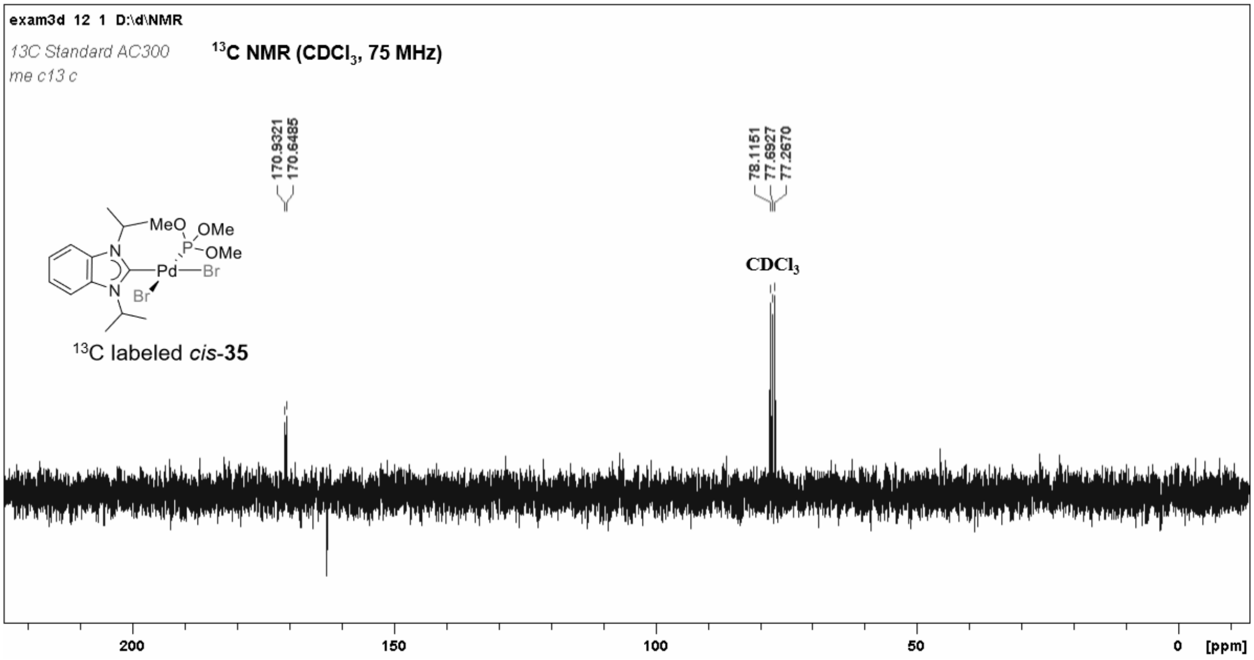
exam3d 12 1 D:\d\NMR
13C Standard AC300
me c13 c
^{13}C NMR (CDCl$_3$, 75 MHz)
170.9321
170.6485
78.1151
77.6927
77.2670
CDCl$_3$
MeO
OMe
OMe
N
N
Pd
Br
Br
P
^{13}C labeled cis-35
200
150
100
50
0
[ppm]

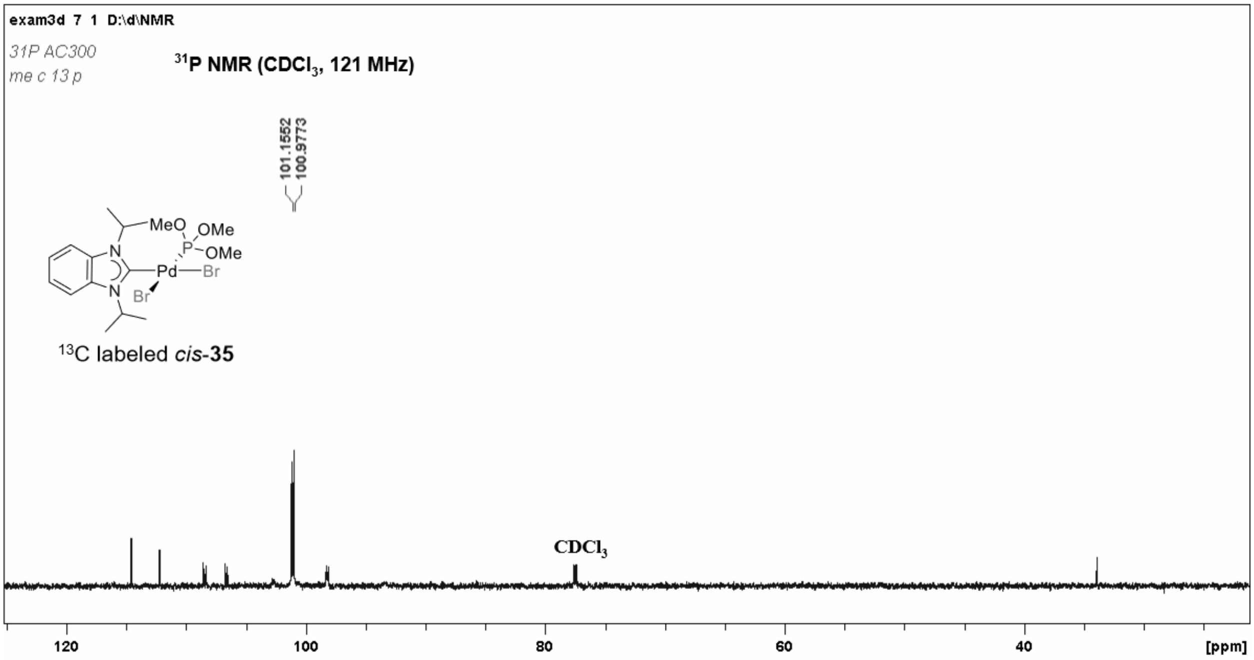
exam3d 7 1 D:\d\NMR
31P AC300
me c 13 p
31P NMR (CDCl3, 121 MHz)
101.1552
100.9773
MeO
OMe
OMe
Pd
Br
Br
13C labeled cis-35
CDCl3
120
100
80
60
40
[ppm]

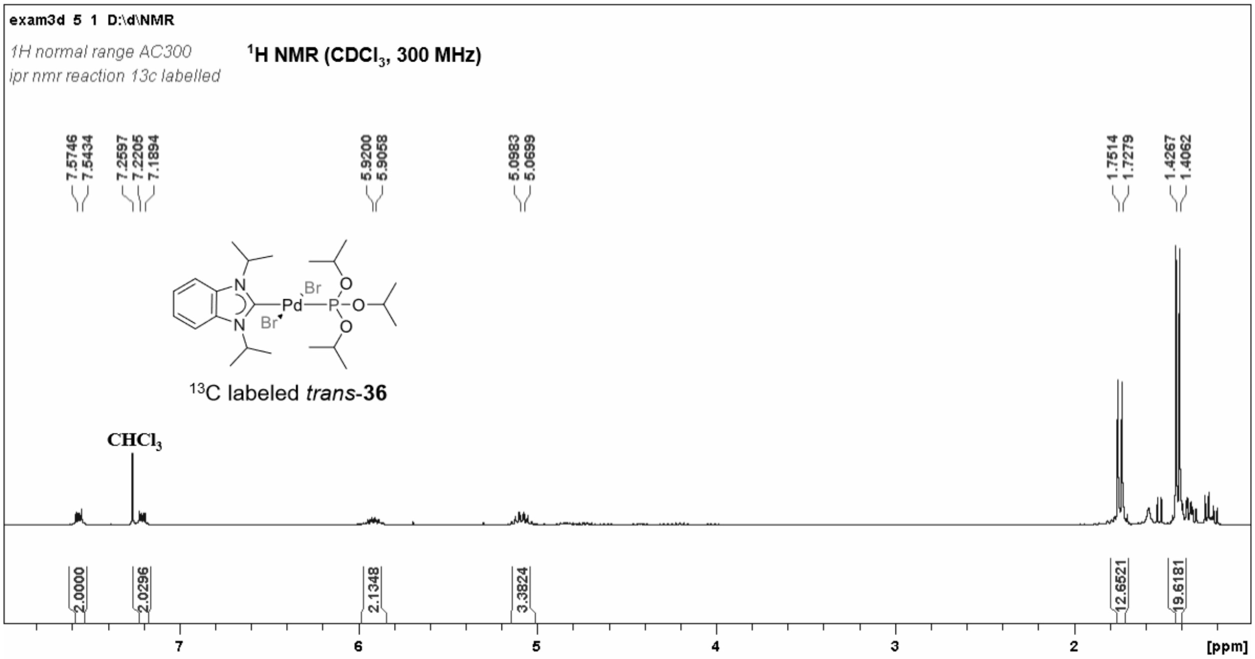
exam3d 5 1 D:\d\NMR
1H normal range AC300
ipr nmr reaction 13c labelled
1H NMR (CDCl3, 300 MHz)
7.5746
7.5434
7.2597
7.2205
7.1894
5.9200
5.9058
5.0983
5.0699
1.7514
1.7279
1.4267
1.4062
Pd
Br
Br
13C labeled trans-36
CHCl3
2.0000
2.0296
2.1348
3.3824
12.6521
19.6181
7
6
5
4
3
2
[ppm]

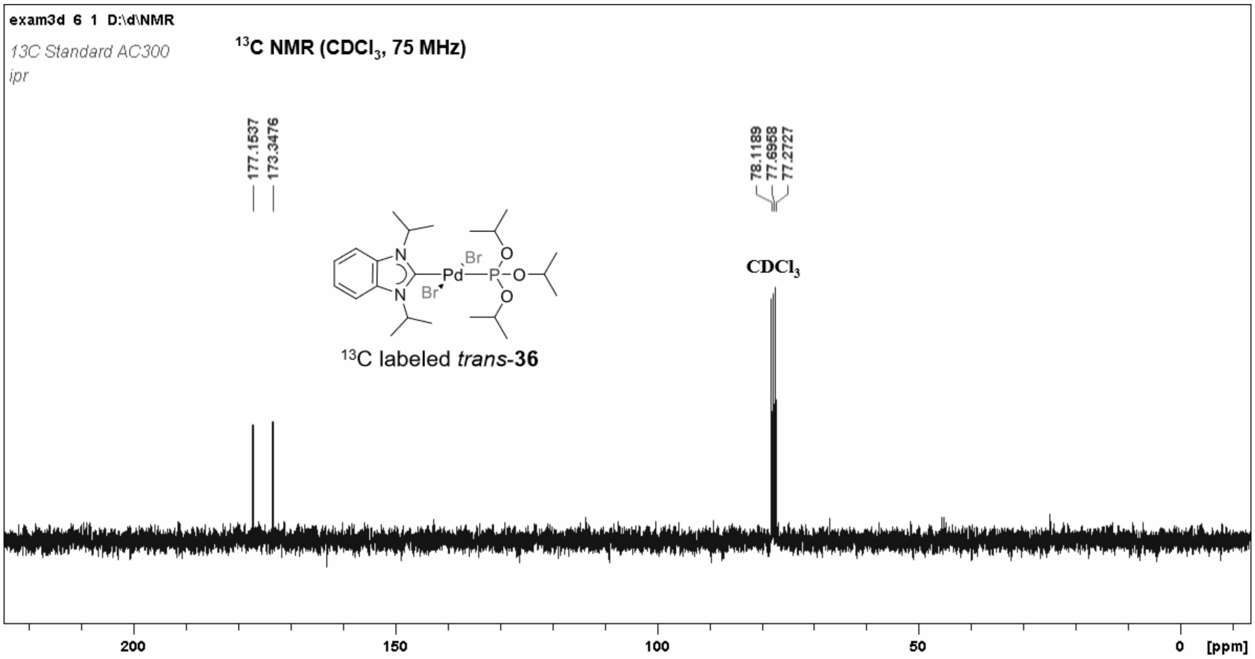
exam3d 6 1 D:\d\NMR
13C Standard AC300
ipr
^{13}C NMR ($CDCl_3$, 75 MHz)
177.1537
173.3476
78.1189
77.6958
77.2727
CDCl$_3$
^{13}C labeled *trans*-**36**
200
150
100
50
0
[ppm]

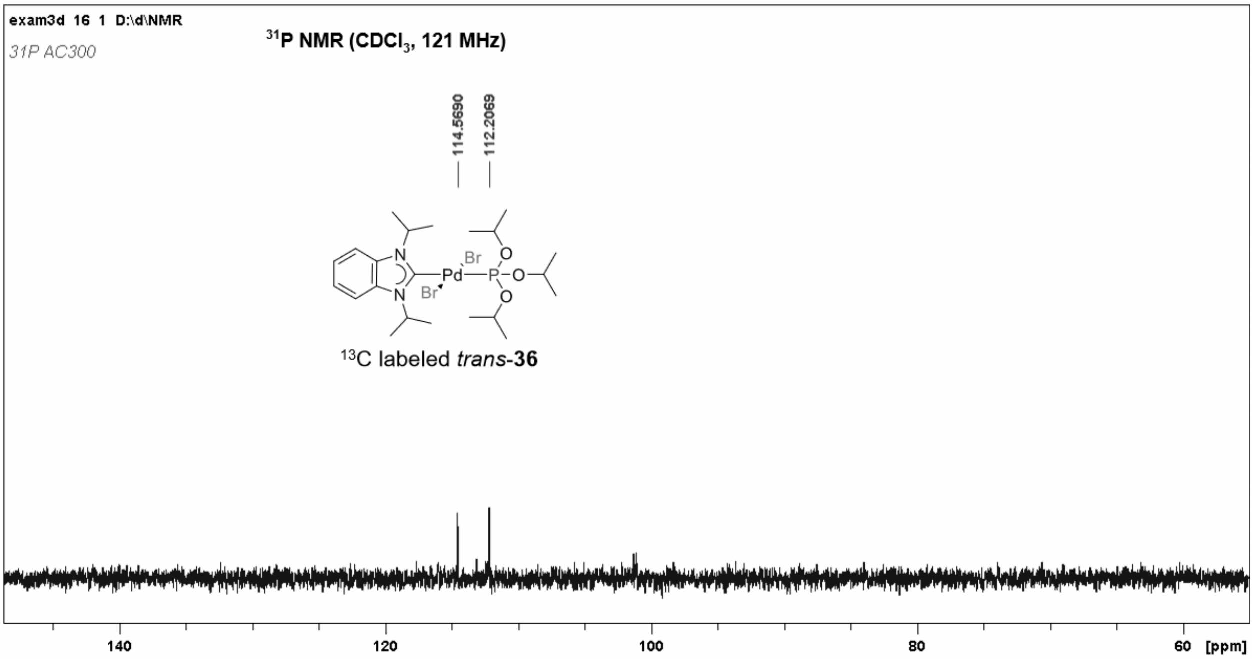
exam3d 16 1 D:\d\NMR
31P AC300
^{31}P NMR ($CDCl_3$, 121 MHz)
114.5690
112.2069
^{13}C labeled *trans*-**36**
140
120
100
80
60
[ppm]

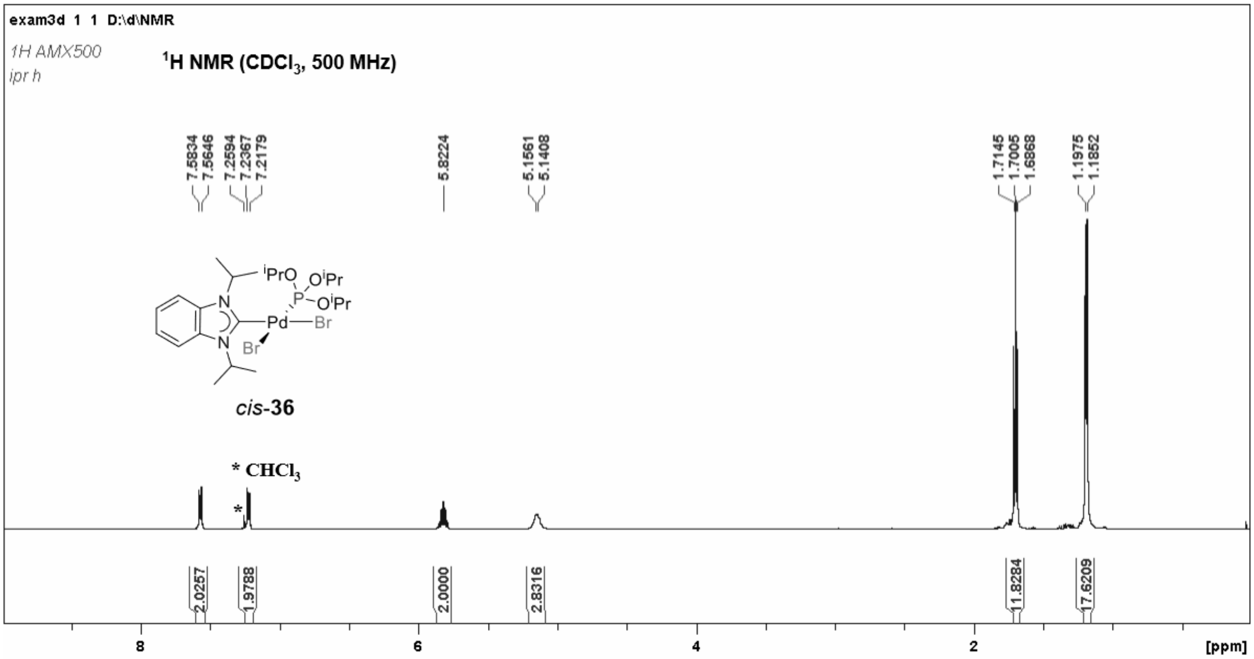
exam3d 1 1 D:\d\NMR
1H AMX500
ipr h
^{1}H NMR (CDCl$_3$, 500 MHz)
7.5834
7.5646
7.2594
7.2367
7.2179
5.8224
5.1561
5.1408
1.7145
1.7005
1.6868
1.1975
1.1852
cis-36
* CHCl$_3$
2.0257
1.9788
2.0000
2.8316
11.8284
17.6209
8
6
4
2
[ppm]

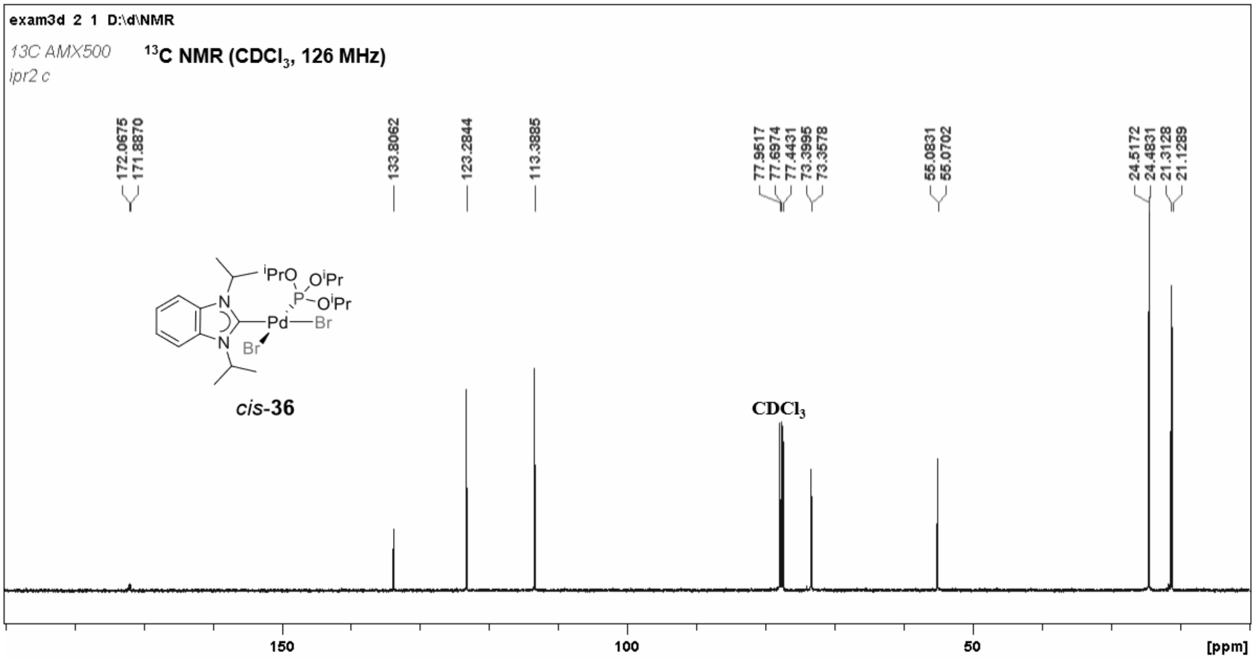
exam3d 2 1 D:\d\NMR
13C AMX500
ipr2 c
^{13}C NMR (CDCl$_3$, 126 MHz)
172.0675
171.8870
133.8062
123.2844
113.3885
77.9517
77.6974
77.4431
73.3995
73.3578
55.0831
55.0702
24.5172
24.4831
21.3128
21.1289
cis-36
CDCl$_3$
150
100
50
[ppm]

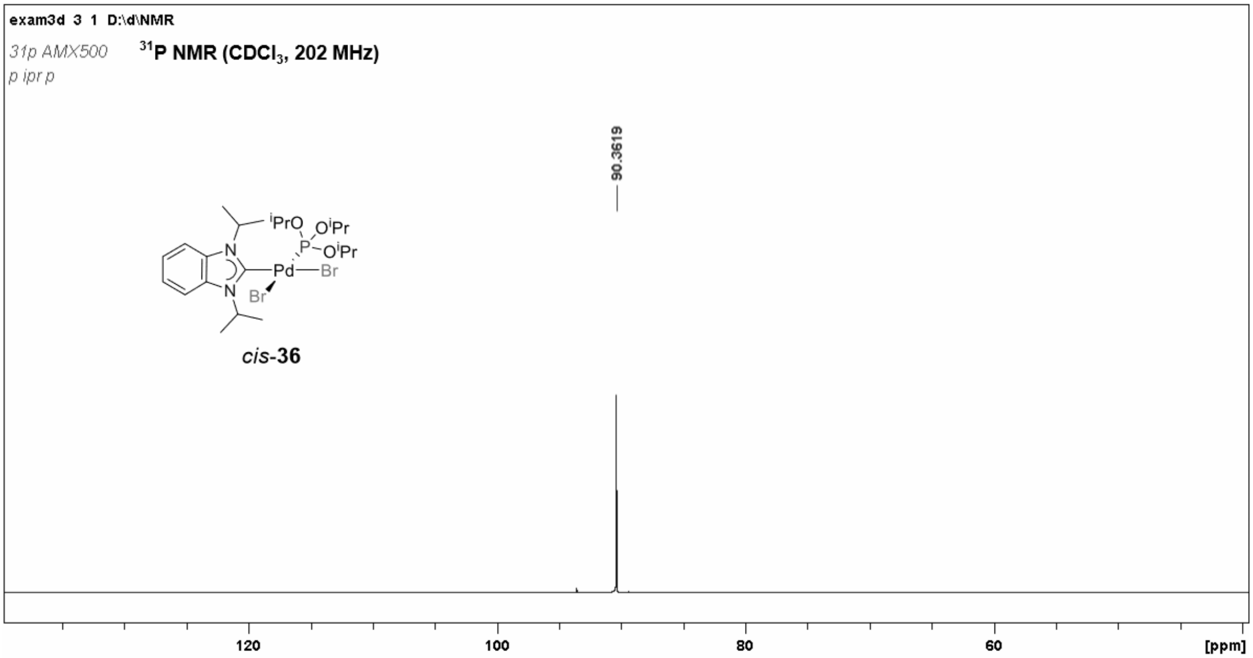
exam3d 3 1 D:\d\NMR
31p AMX500
p ipr p
^{31}P NMR ($CDCl_3$, 202 MHz)
90.3619
^{i}PrO
O^{i}Pr
O^{i}Pr
P
N
N
Pd
Br
Br
cis-36
120
100
80
60
[ppm]

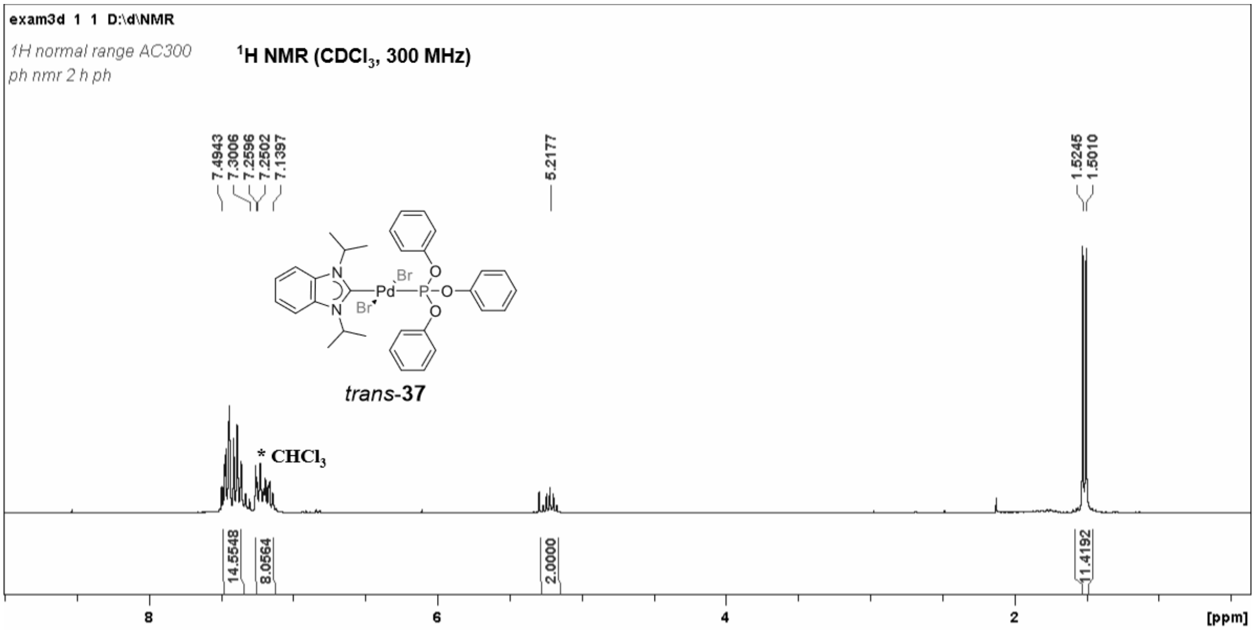
exam3d 1 1 D:\d\NMR
1H normal range AC300
ph nmr 2 h ph
^{1}H NMR ($CDCl_3$, 300 MHz)
7.4943
7.3006
7.2596
7.2502
7.1397
5.2177
1.5245
1.5010
N
N
Pd
Br
Br
P
O
O
O
trans-37
* $CHCl_3$
14.5548
8.0564
2.0000
11.4192
8
6
4
2
[ppm]

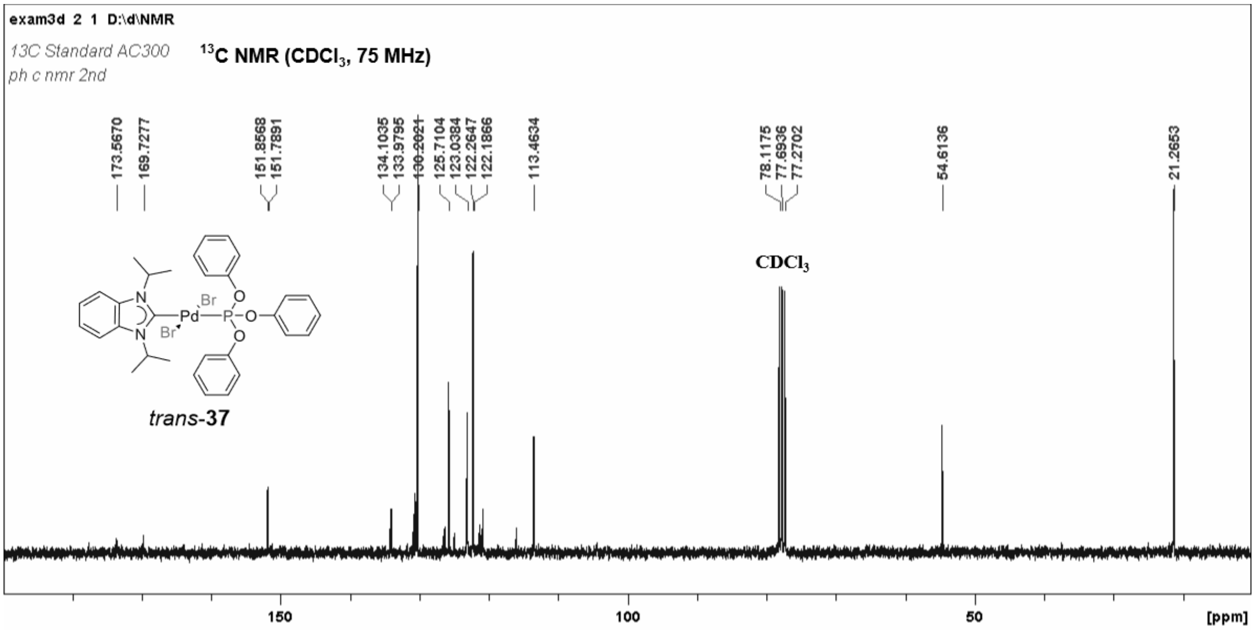
exam3d 2 1 D:\d\NMR
13C Standard AC300
ph c nmr 2nd
^{13}C NMR ($CDCl_3$, 75 MHz)
173.5670
169.7277
151.8568
151.7891
134.1035
133.9795
130.2021
125.7104
123.0384
122.2647
122.1866
113.4634
78.1175
77.6936
77.2702
54.6136
21.2653
$CDCl_3$
Br
Pd
P
O
N
trans-37
150
100
50
[ppm]

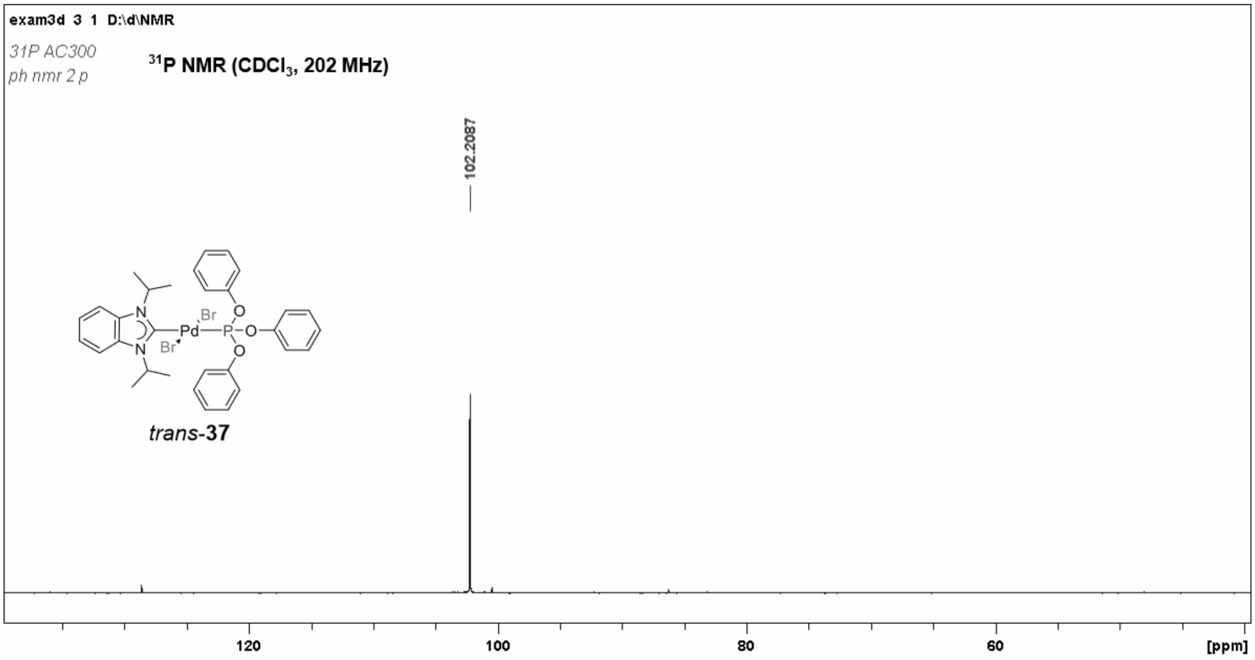
exam3d 3 1 D:\d\NMR
31P AC300
ph nmr 2 p
^{31}P NMR ($CDCl_3$, 202 MHz)
102.2087
Br
Pd
P
O
N
trans-37
120
100
80
60
[ppm]

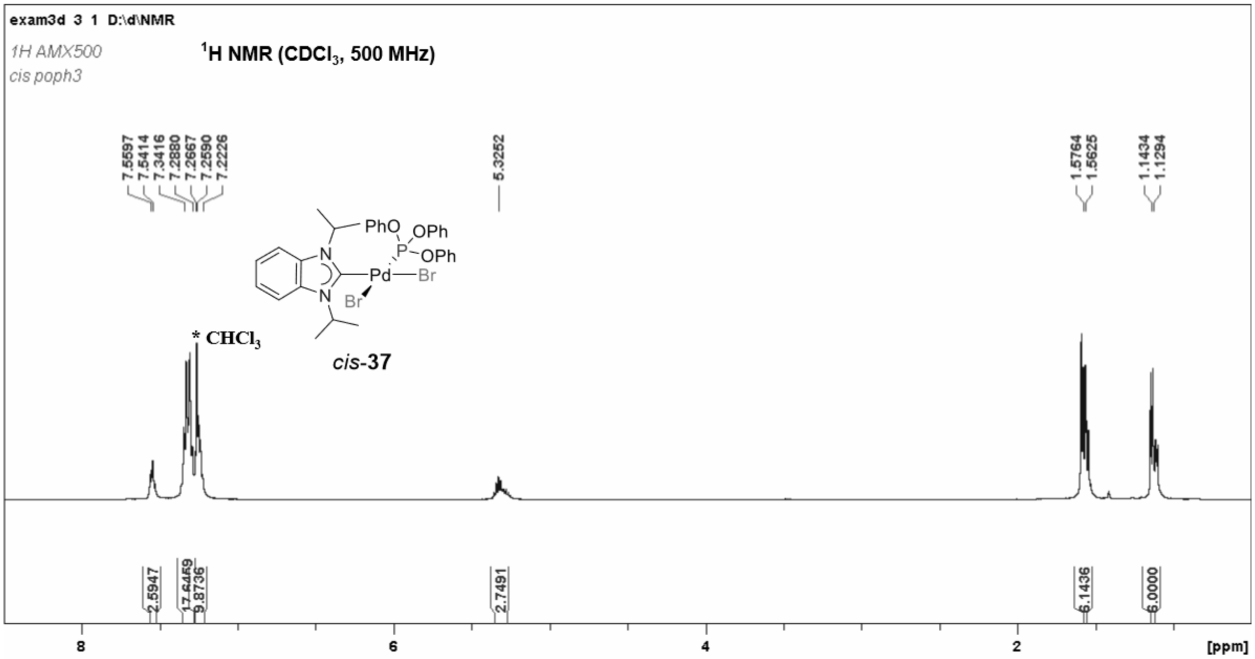
exam3d 3 1 D:\d\NMR
1H AMX500
cis poph3
^1H NMR ($CDCl_3$, 500 MHz)
7.5597
7.5414
7.3416
7.2880
7.2667
7.2590
7.2226
5.3252
1.5764
1.5625
1.1434
1.1294
PhO
OPh
P
OPh
N
Pd
Br
Br
N
* $CHCl_3$
cis-37
2.5947
17.6459
9.8736
2.7491
6.1436
6.0000
8
6
4
2
[ppm]

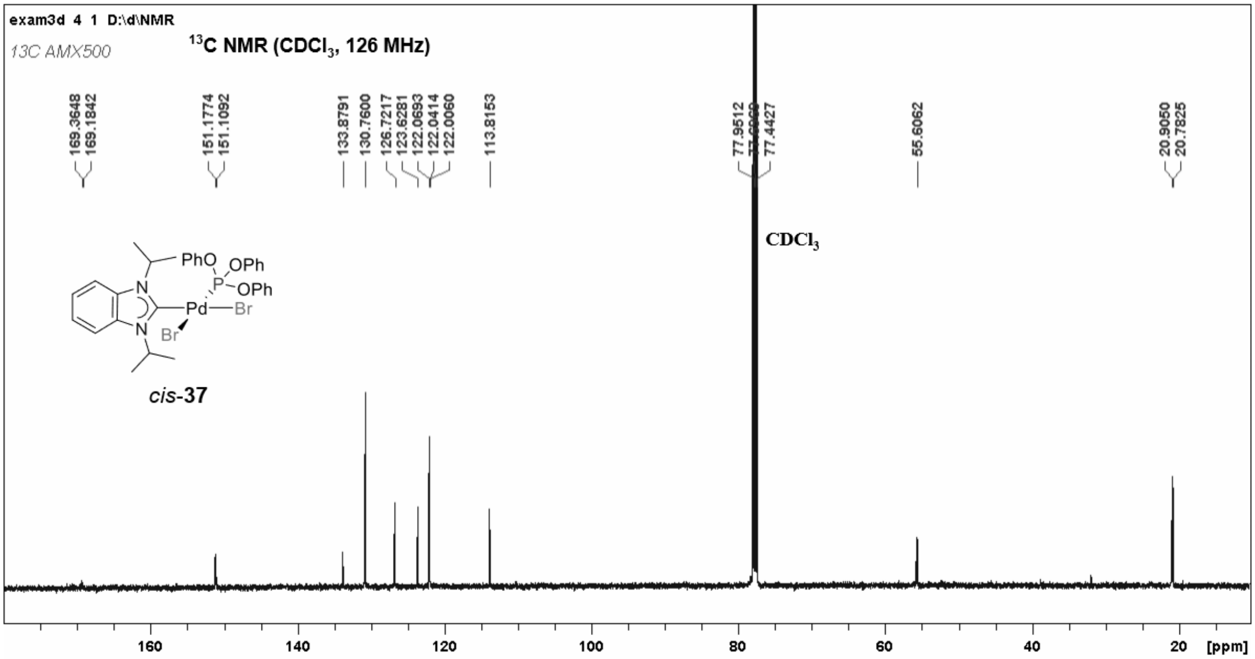
exam3d 4 1 D:\d\NMR
13C AMX500
^{13}C NMR ($CDCl_3$, 126 MHz)
169.3648
169.1842
151.1774
151.1092
133.8791
130.7600
126.7217
123.6281
122.0693
122.0414
122.0060
113.8153
77.9512
77.4427
55.6062
20.9050
20.7825
$CDCl_3$
PhO
OPh
P
OPh
N
Pd
Br
Br
N
cis-37
160
140
120
100
80
60
40
20
[ppm]

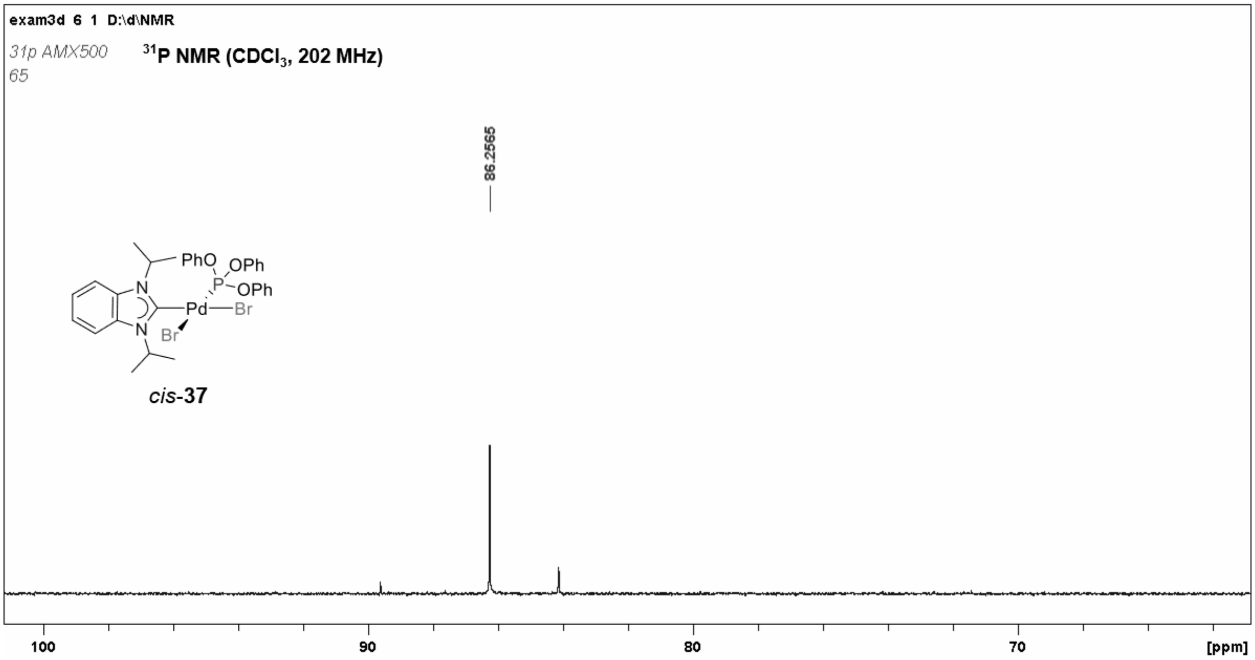
exam3d 6 1 D:\d\NMR
31p AMX500
65
^{31}P NMR ($CDCl_3$, 202 MHz)
86.2565
PhO
OPh
OPh
P
N
N
Pd
Br
Br
cis-37
100
90
80
70
[ppm]

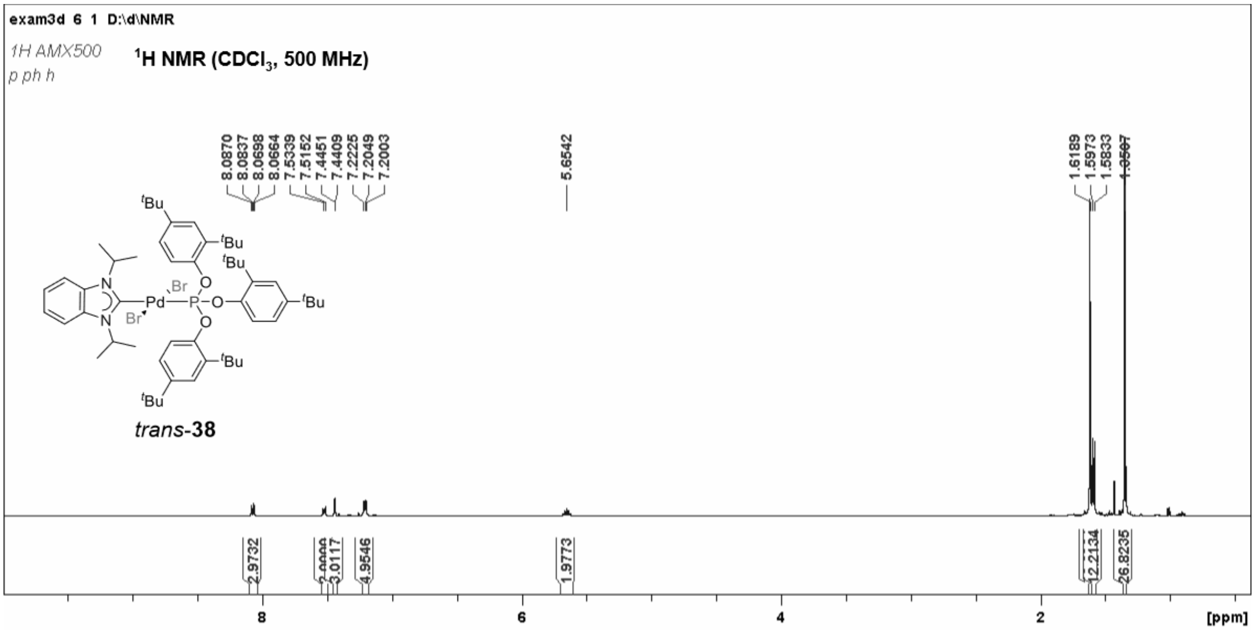
exam3d 6 1 D:\d\NMR
1H AMX500
p ph h
^1H NMR ($CDCl_3$, 500 MHz)
8.0870
8.0837
8.0698
8.0664
7.5339
7.5152
7.4451
7.4409
7.2225
7.2049
7.2003
5.6542
1.6189
1.5973
1.5833
1.3567
tBu
Br
Pd
P
O
N
N
trans-38
2.9732
3.0117
4.9546
1.9773
12.2134
26.8235
8
6
4
2
[ppm]

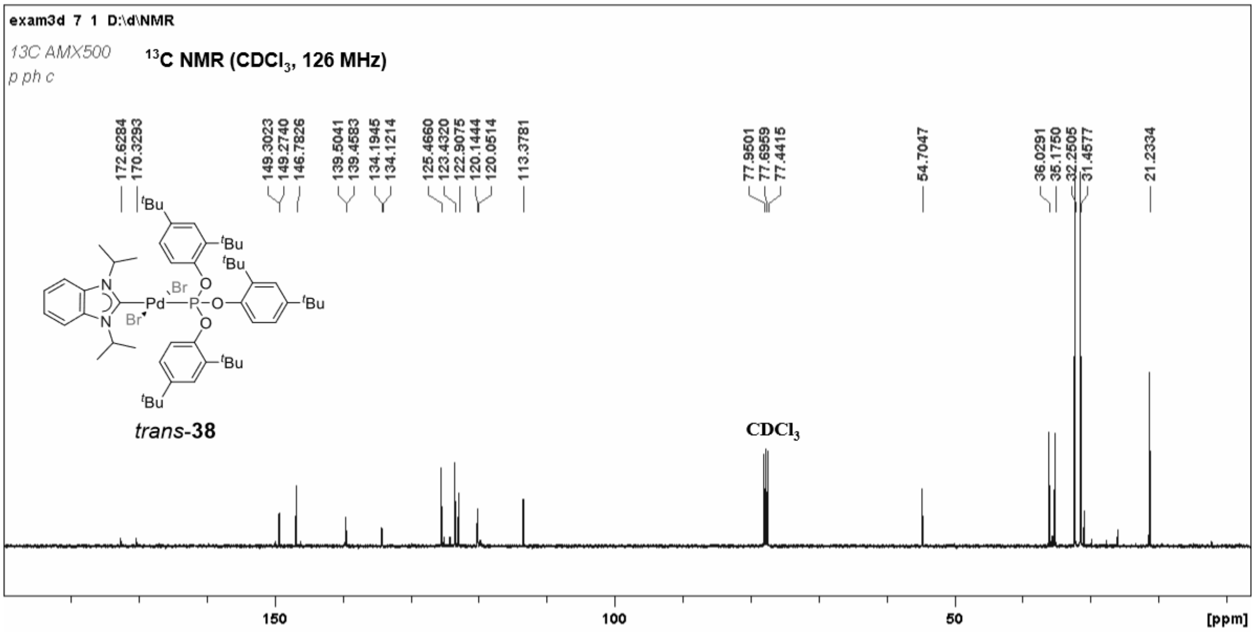
exam3d 7 1 D:\d\NMR
13C AMX500
p ph c
^{13}C NMR ($CDCl_3$, 126 MHz)
172.6284
170.3293
149.3023
149.2740
146.7826
139.5041
139.4583
134.1945
134.1214
125.4660
123.4320
122.9075
120.1444
120.0514
113.3781
77.9501
77.6959
77.4415
54.7047
36.0291
35.1750
32.2505
31.4577
21.2334
trans-38
$CDCl_3$
150
100
50
[ppm]

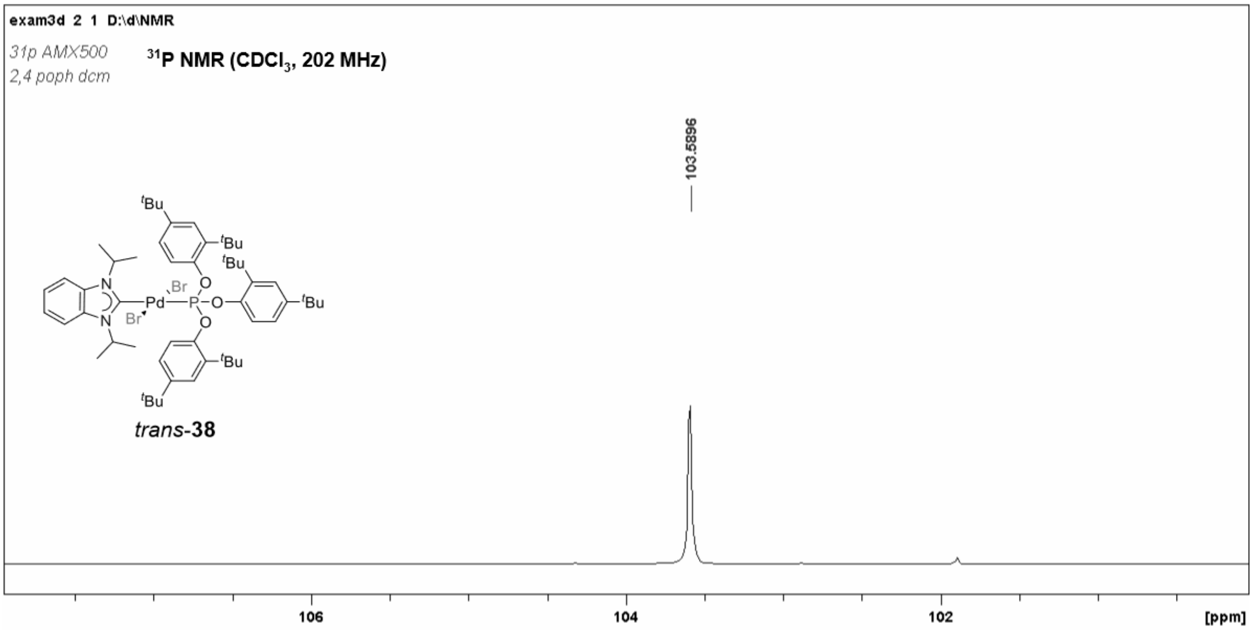
exam3d 2 1 D:\d\NMR
31p AMX500
2,4 poph dcm
^{31}P NMR ($CDCl_3$, 202 MHz)
103.5896
trans-38
106
104
102
[ppm]

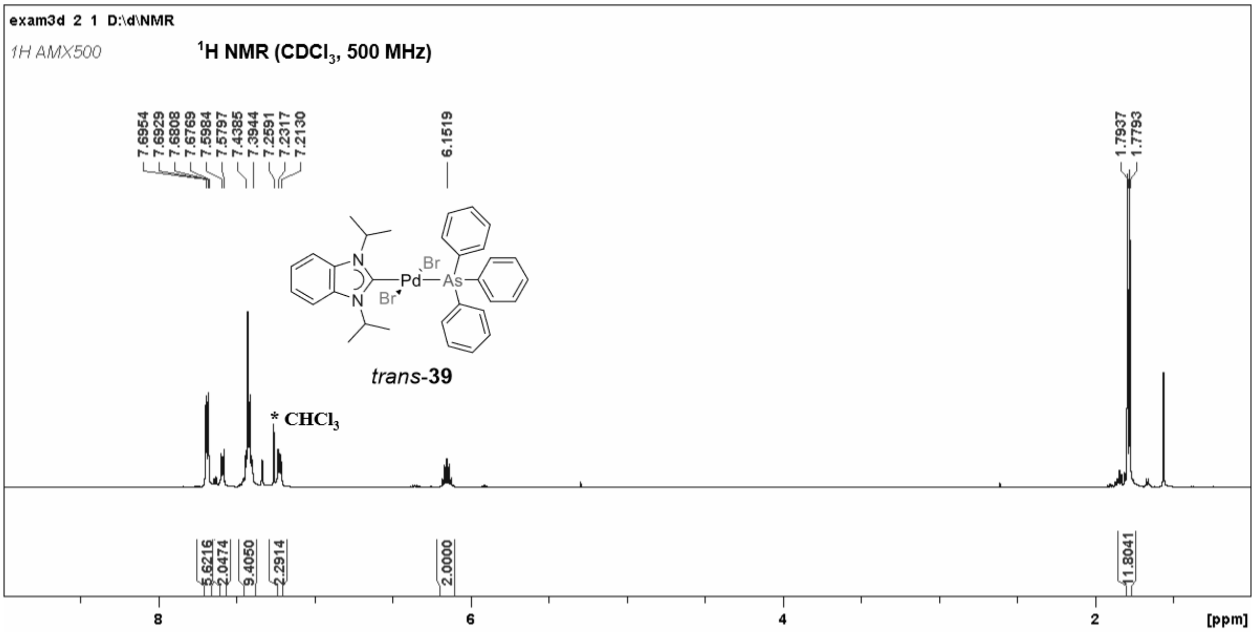
exam3d 2 1 D:\d\NMR
1H AMX500
^{1}H NMR (CDCl$_3$, 500 MHz)
7.6954 7.6929 7.6808 7.6769 7.5984 7.5797 7.4385 7.3944 7.2591 7.2317 7.2130
6.1519
1.7937 1.7793
Br
Pd
As
Br
N
N
trans-39
* CHCl$_3$
5.6216 2.0474 9.4050 2.2914
2.0000
11.8041
8
6
4
2
[ppm]

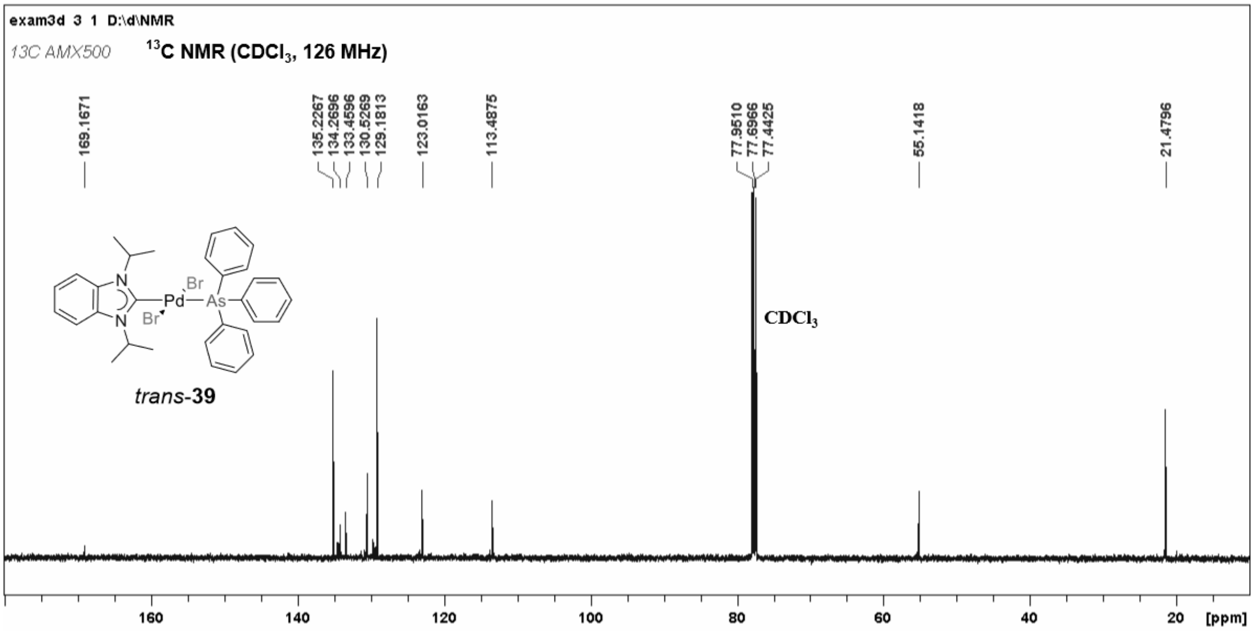
exam3d 3 1 D:\d\NMR
13C AMX500
^{13}C NMR (CDCl$_3$, 126 MHz)
169.1671
135.2267 134.2696 133.4596 130.5269 129.1813
123.0163
113.4875
77.9510 77.6966 77.4425
55.1418
21.4796
Br
Pd
As
Br
N
N
trans-39
CDCl$_3$
160
140
120
100
80
60
40
20
[ppm]

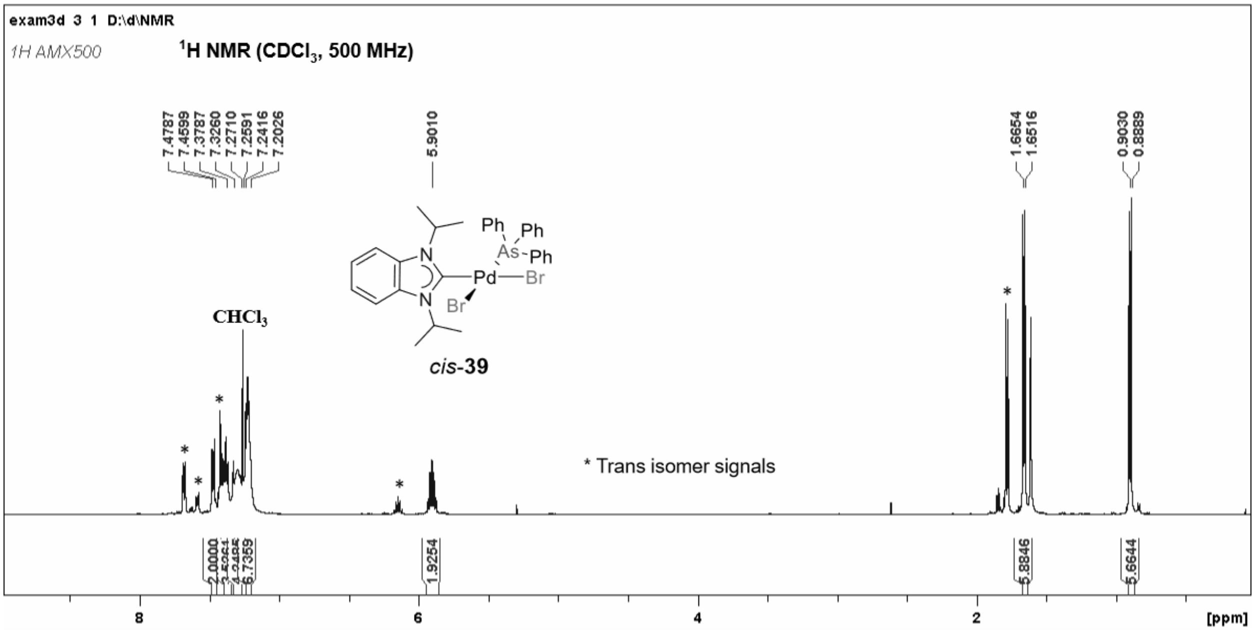

exam3d 3 1 D:\d\NMR
1H AMX500
1H NMR (CDCl3, 500 MHz)
7.4787
7.4599
7.3787
7.3260
7.2710
7.2591
7.2416
7.2026
5.9010
1.6654
1.6516
0.9030
0.8889
Ph
Ph
As
Ph
N
N
Pd
Br
Br
CHCl3
cis-39
* Trans isomer signals
2.0000
1.9254
5.8846
5.6644
8
6
4
2
[ppm]

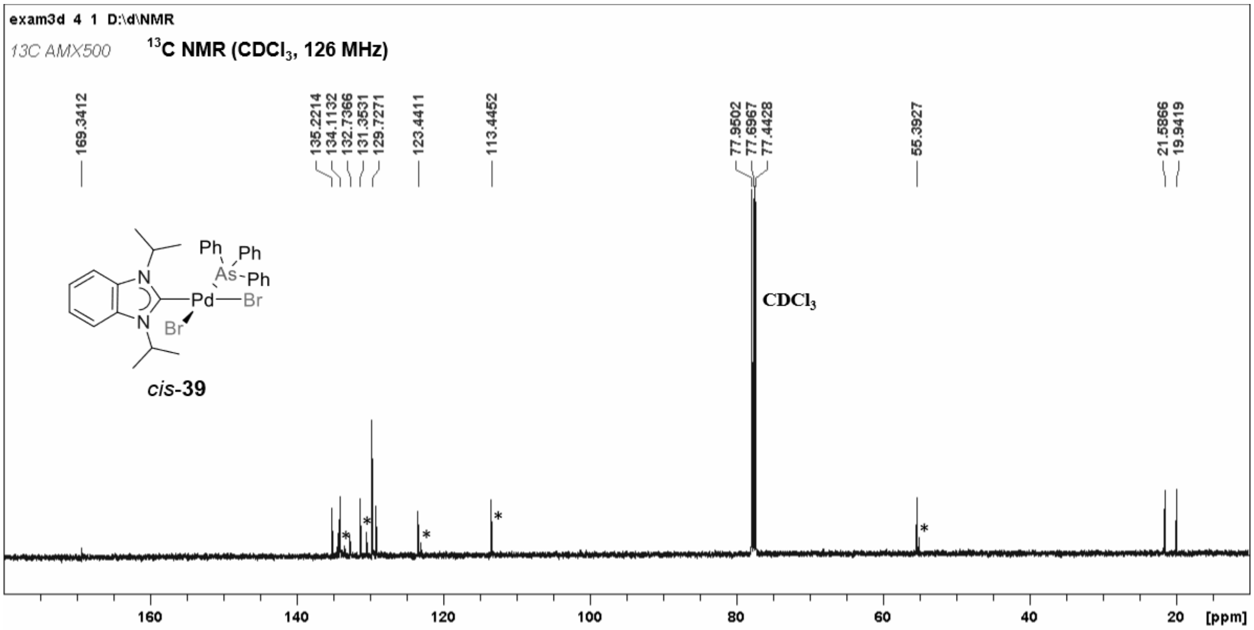

exam3d 4 1 D:\d\NMR
13C AMX500
13C NMR (CDCl3, 126 MHz)
169.3412
135.2214
134.1132
132.7366
131.3531
129.7271
123.4411
113.4452
77.9502
77.6967
77.4428
55.3927
21.5866
19.9419
Ph
Ph
As
Ph
N
N
Pd
Br
Br
cis-39
CDCl3
160
140
120
100
80
60
40
20
[ppm]

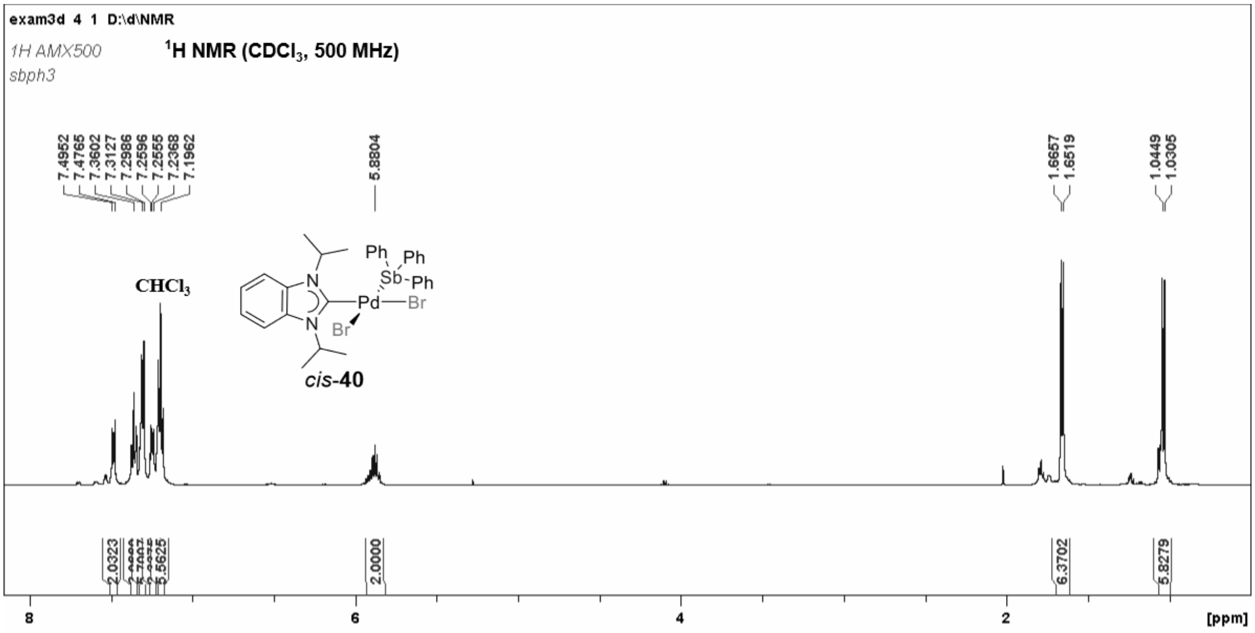
exam3d 4 1 D:\d\NMR
1H AMX500
sbph3
^{1}H NMR (CDCl$_3$, 500 MHz)
7.4952
7.4765
7.3602
7.3127
7.2986
7.2596
7.2555
7.2368
7.1962
5.8804
1.6657
1.6519
1.0449
1.0305
CHCl$_3$
Ph
Ph
Sb
Ph
N
N
Pd
Br
Br
cis-**40**
2.0323
2.0000
6.3702
5.8279
8
6
4
2
[ppm]

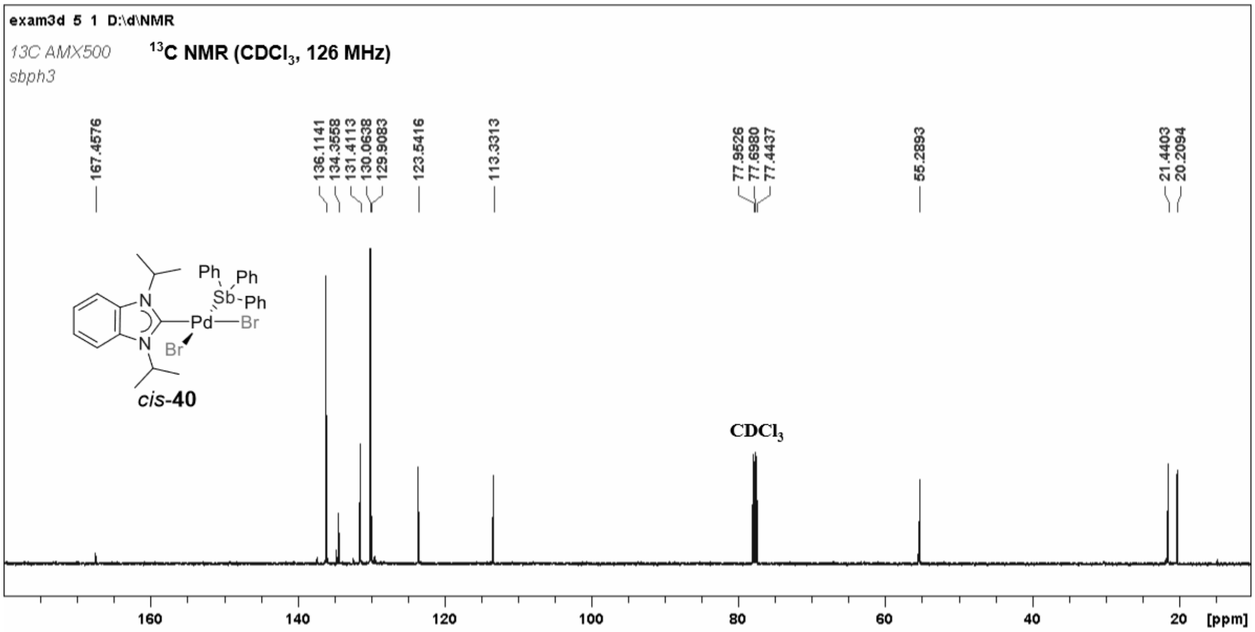
exam3d 5 1 D:\d\NMR
13C AMX500
sbph3
^{13}C NMR (CDCl$_3$, 126 MHz)
167.4576
136.1141
134.3558
131.4113
130.0638
129.9083
123.5416
113.3313
77.9526
77.6980
77.4437
55.2893
21.4403
20.2094
Ph
Ph
Sb
Ph
N
N
Pd
Br
Br
cis-**40**
CDCl$_3$
160
140
120
100
80
60
40
20
[ppm]

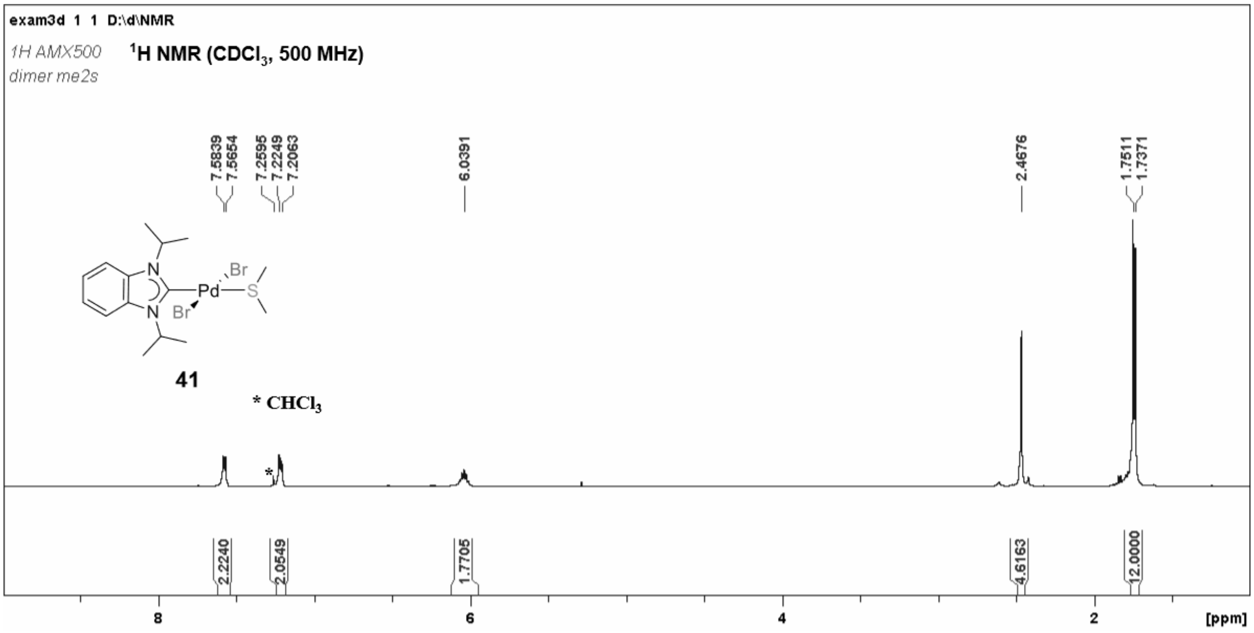
exam3d 1 1 D:\d\NMR
1H AMX500
dimer me2s
^{1}H NMR ($CDCl_3$, 500 MHz)
7.5839
7.5654
7.2595
7.2249
7.2063
6.0391
2.4676
1.7511
1.7371
Br
Pd
S
N
N
Br
41
* $CHCl_3$
*
2.2240
2.0549
1.7705
4.6163
12.0000
8
6
4
2
[ppm]

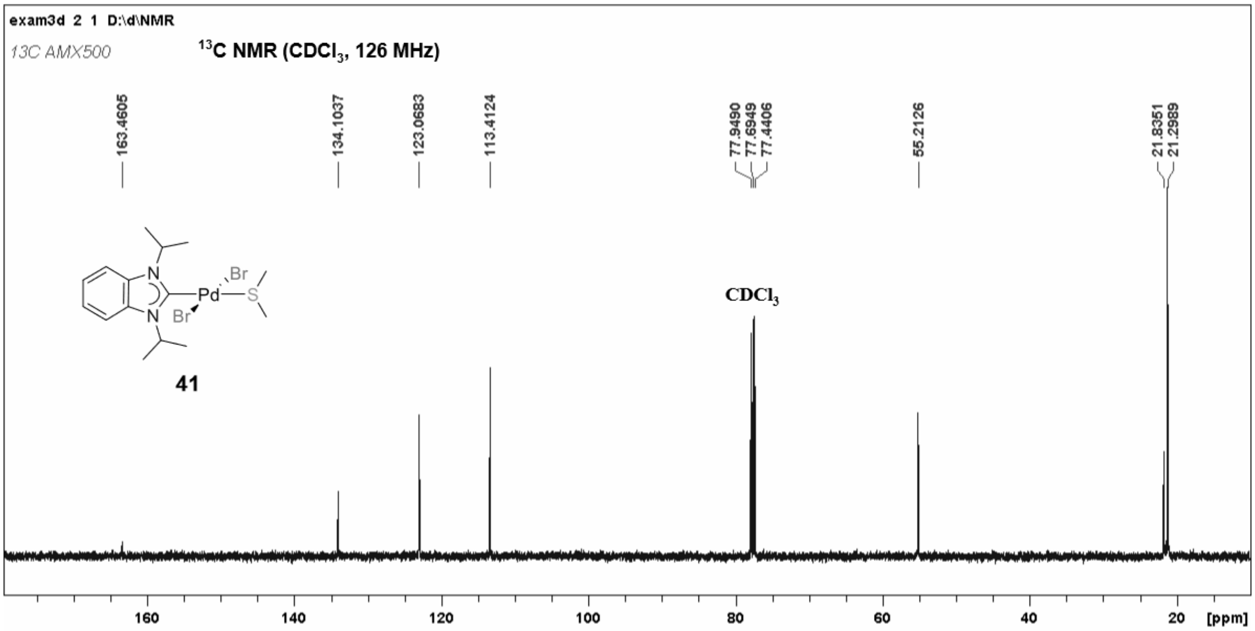
exam3d 2 1 D:\d\NMR
13C AMX500
^{13}C NMR ($CDCl_3$, 126 MHz)
163.4605
134.1037
123.0683
113.4124
77.9490
77.6949
77.4406
55.2126
21.8351
21.2989
Br
Pd
S
N
N
Br
41
$CDCl_3$
160
140
120
100
80
60
40
20
[ppm]

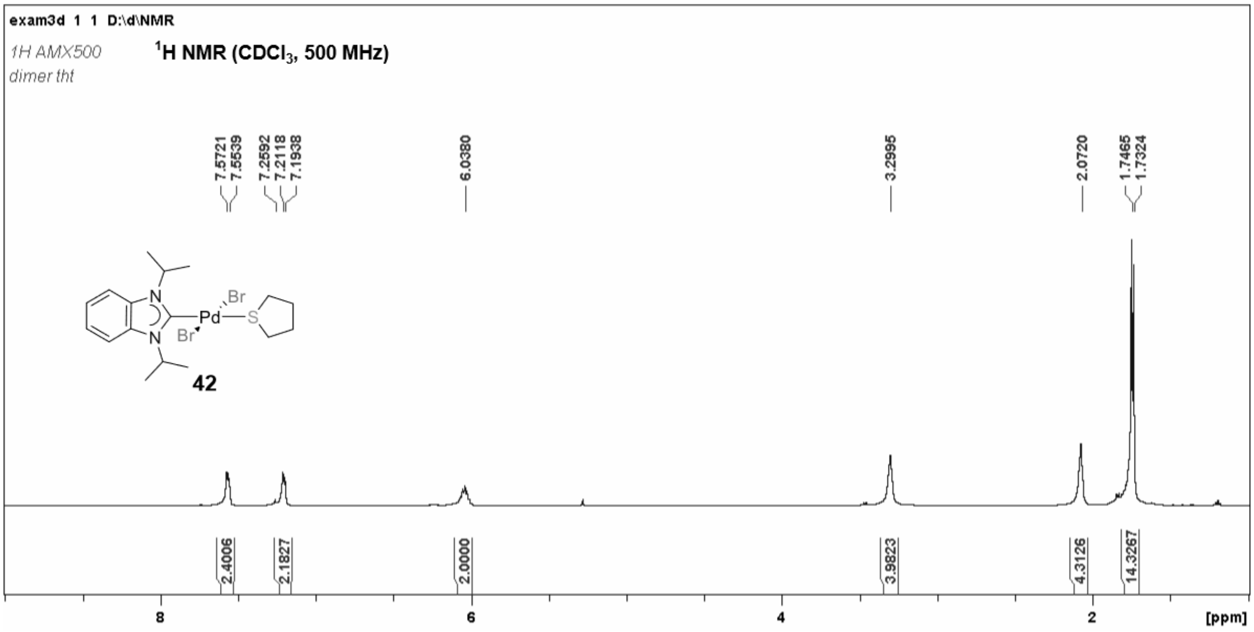
exam3d 1 1 D:\d\NMR
1H AMX500
dimer tht
1H NMR (CDCl3, 500 MHz)
7.5721
7.5539
7.2592
7.2118
7.1938
6.0380
3.2995
2.0720
1.7465
1.7324
42
2.4006
2.1827
2.0000
3.9823
4.3126
14.3267
8
6
4
2
[ppm]

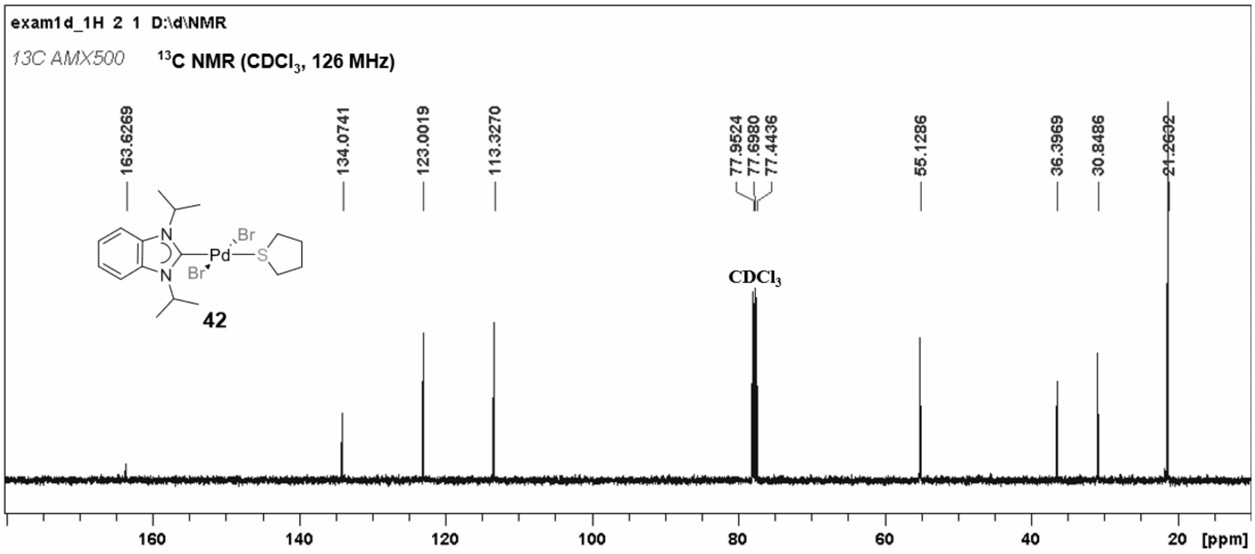
exam1d_1H 2 1 D:\d\NMR
13C AMX500
13C NMR (CDCl3, 126 MHz)
163.6269
134.0741
123.0019
113.3270
77.9524
77.6980
77.4436
55.1286
36.3969
30.8486
21.2602
CDCl3
42
160
140
120
100
80
60
40
20
[ppm]

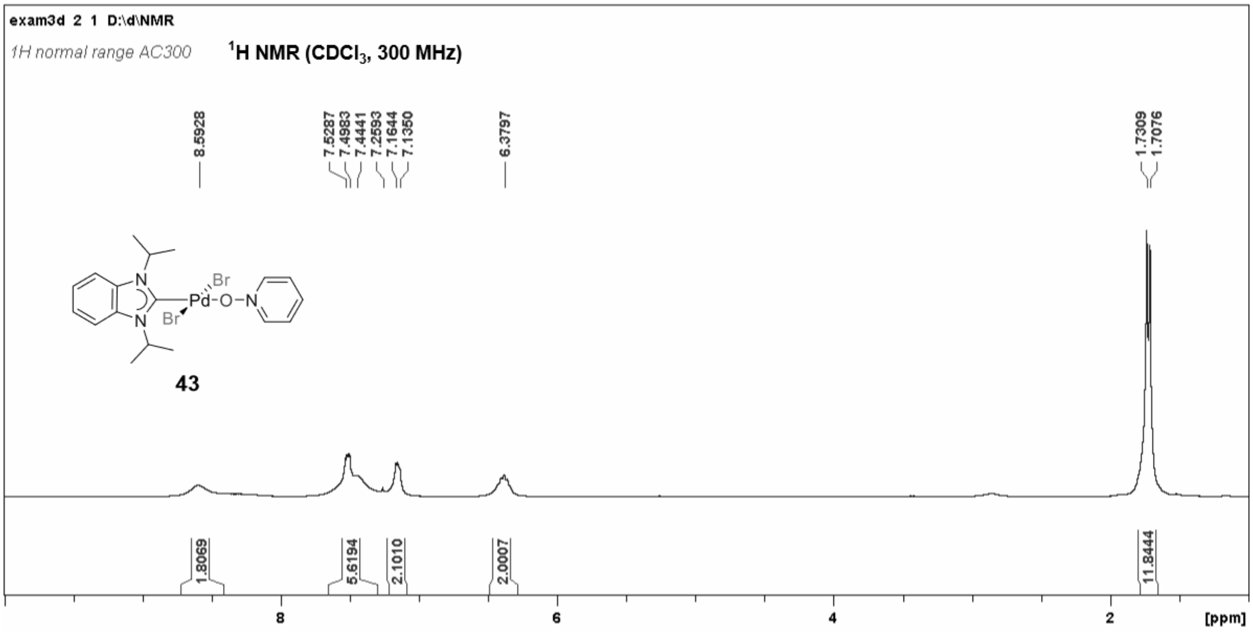
exam3d 2 1 D:\d\NMR
1H normal range AC300
1H NMR (CDCl3, 300 MHz)
8.5928
7.5287
7.4983
7.4441
7.2593
7.1644
7.1350
6.3797
1.7309
1.7076
Br
Pd
O
N
Br
N
N
43
1.8069
5.6194
2.1010
2.0007
11.8444
8
6
4
2
[ppm]

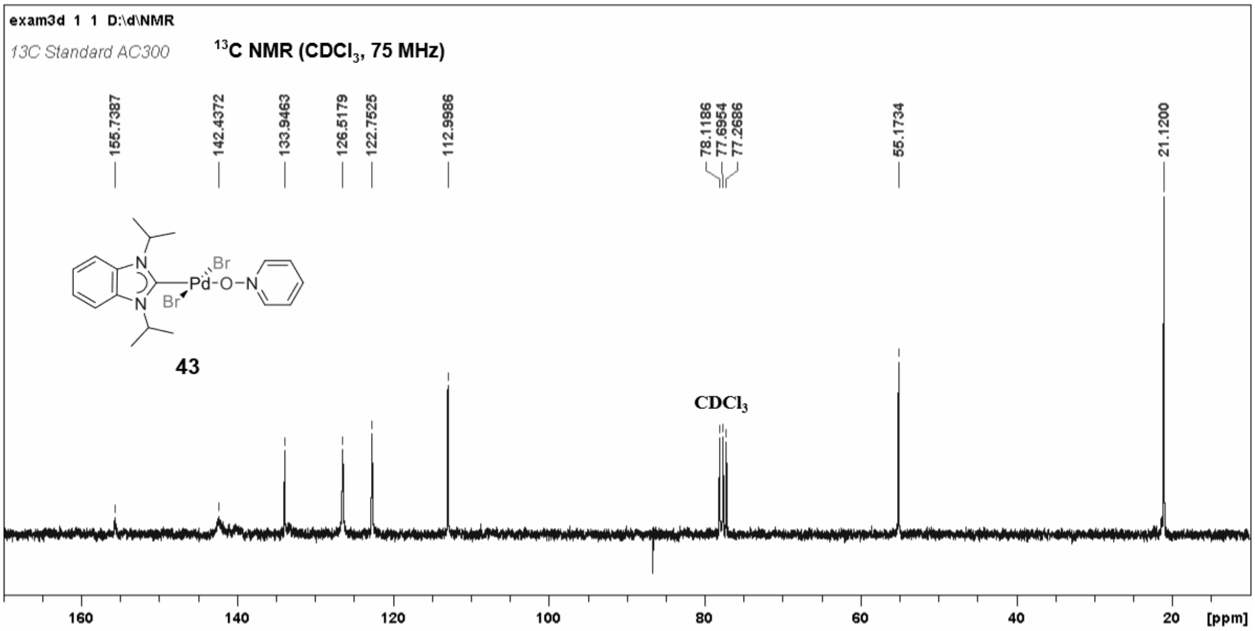
exam3d 1 1 D:\d\NMR
13C Standard AC300
13C NMR (CDCl3, 75 MHz)
155.7387
142.4372
133.9463
126.5179
122.7525
112.9986
78.1186
77.6954
77.2686
55.1734
21.1200
Br
Pd
O
N
Br
N
N
43
CDCl3
160
140
120
100
80
60
40
20
[ppm]
